# High-Throughput Profiling of Prenylelongases Enables the Assembly of Modified Prenoids

**DOI:** 10.1021/acschembio.6c00250

**Published:** 2026-06-15

**Authors:** Hui Li, Anjali Sital, Clemens Mayer, Felix Kaspar

**Affiliations:** † Biomolecular Chemistry and Catalysis, 3647 Stratingh Institute for Chemistry, University of Groningen, Nijenborgh 4, 9747 AG Groningen, The Netherlands; ‡ Organic Chemistry, 9379 Saarland University, 66123 Saarbrücken, Germany; § Department of Natural Product Biotechnology, Helmholtz Institute for Pharmaceutical Research Saarland (HIPS), Helmholtz Centre for Infection Research (HZI) and Department of Pharmacy, PharmaScienceHub (PSH), Saarland University, Campus E8.1, 66123 Saarbrücken, Germany

## Abstract

Modified (non-natural) prenoids are useful as chemical biology tools, designer fragrances, and building blocks of bioactive compounds. However, their laborious synthesis generally limits their widespread application. Although prenylelongases offer potential biocatalytic access to modified long-chain prenoids, these enzymes have remained underexplored since (i) they are difficult to assay in high throughput and (ii) their pyrophosphate substrates are challenging and expensive to synthesize. To address these gaps, we herein report how prenylelongases can be interrogated with continuous high-throughput assays using readily accessible monophosphates of their native and modified substrates, bypassing the need for laborious pyrophosphate synthesis. We demonstrate the utility of this assay platform for the biochemical characterization of a panel of diverse prenylelongases, their profiling with non-native substrates, and two exploratory engineering campaigns. Lastly, we demonstrate that wild-type PEs and their variants can be used to assemble modified prenoids selectively at the (semi)­preparative scale. The methods and biochemical data herein will simplify the engineering of PEs toward expanded substrate scopes and facilitate biocatalytic access to modified prenoids and derivatives thereof.

## Graphical abstract

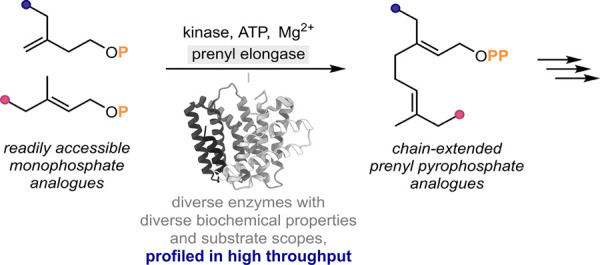

## Introduction

Naturally occurring prenols and their esters are ubiquitous as flavors and fragrances across the food and cosmetic industries.^1,2^ Modified (non-natural) prenoids have found applications as chemical biology tools (e.g., as taggable protein anchors^3–6^ or photochemical handles^7,8^ ), synthetic intermediates (e.g., for the preparation of diverse natural products,^9^ including vitamin K derivatives), designer fragrances^10^ and may find future applications as emulsifiers^11^ or in plastics^12,13^ (Figure 1a).

**Figure 1:**
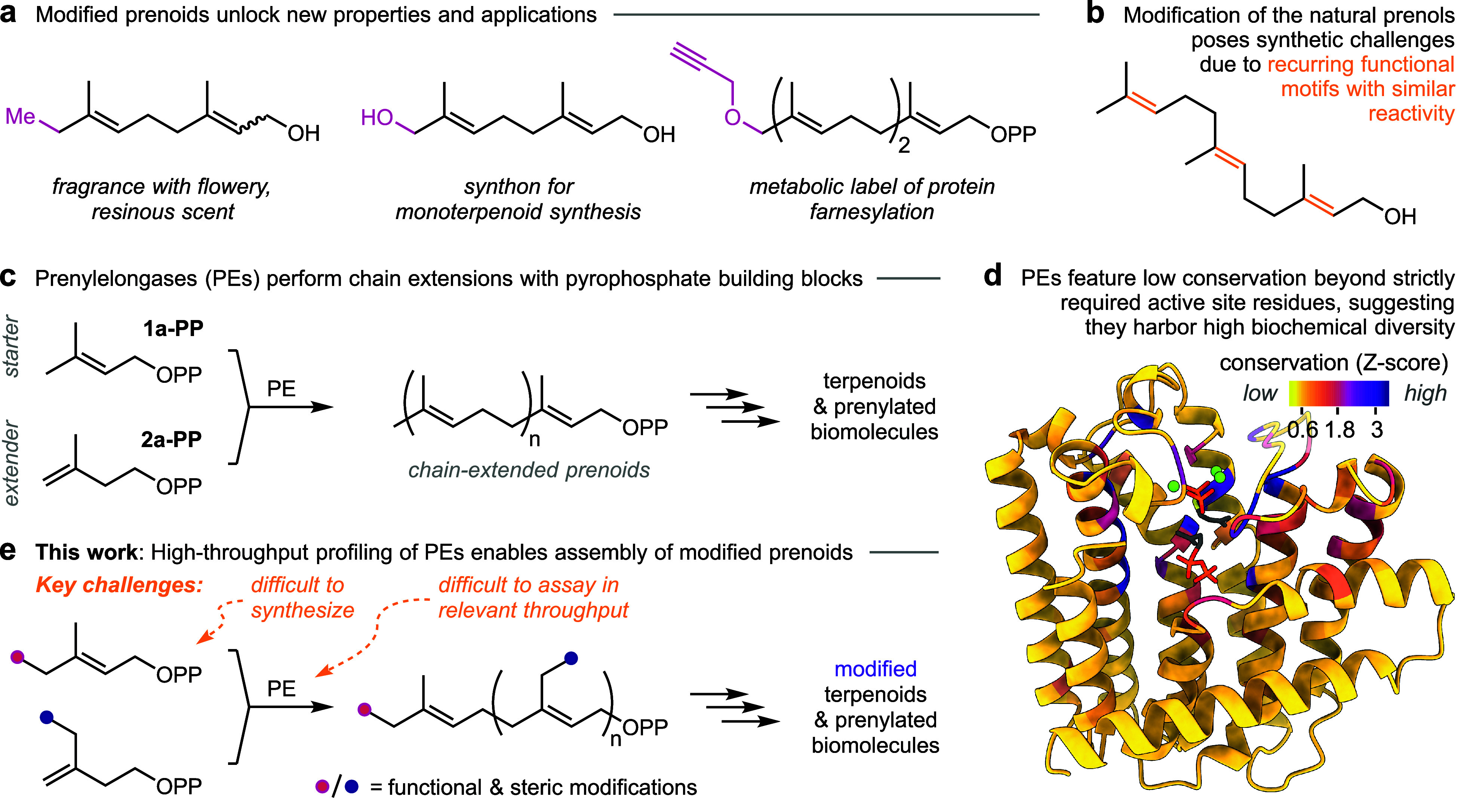
Utility and biosynthesis of modified prenoids. (a) Exemplary applications of modified prenoids.^3,9,10^ (b) Sparse prefunctionalization of natural prenols (e.g., farnesol) with recurring motifs complicates their selective modification. (c) Biosynthetic logic of natural prenoid and terpenoid assembly. (d) PEs across different species exhibit low sequence identities as generally only the metal- or pyrophosphate-coordinating residues are conserved. These conservation data were generated from a representative set of 166 PE entries from the Interpro database (IPR008949, listed in the SI and available for download from the externally hosted Supporting Information)^54^ and mapped onto an AlphaFold2 model^55^ of the FPPS from *G. stearothermophilus* overlaid with the cocrystallized ligands of the *E. coli* homologue (PDB 1rqi).^56^ Z-scores express standard deviations from the mean, with a low (or negative) score indicating that a position is not conserved and a high score (e.g., >2) suggesting relevant conservation of a residue at that position.^57^ (e) Challenges associated with the profiling of PEs with modified substrates. The abbreviation PP indicates a pyrophosphate unit.

Despite their utility and versatility, the routine application of modified prenoids is currently hampered by their challenging synthesis. Lack of synthetic handles and recurring functional motifs (Figure 1b) complicate regioselective modification^14–17^ and analogues of varying chain length typically require individual de novo synthesis.^4,18^ As a result, biocatalytic diversification of prenoids following modular biosynthetic logic presents an intriguing approach to facilitate the access to these compounds and would provide a valuable addition to the synthetic toolbox.

Prenylelongases (PEs, also known as oligoprenyl diphosphate synthases) offer a promising biocatalytic strategy to the assembly of diversely modified prenoids. Natively, PEs such as farnesyl pyrophosphate synthase (FPPS) perform iterative chain extensions of prenyl pyrophosphate (**1a-PP**, a starter unit) with isoprenyl pyrophosphate (**2a-PP**, an extender unit) to generate long-chain prenyl pyrophosphates (Figure 1c).^19,20^ PEs are spread across all kingdoms of life and exhibit a highly conserved overall structure and mechanism. The low degree of sequence identity across this family (including regions of and near the active site, Figure 1d) suggests that PEs may exhibit significant biochemical diversity or different levels of substrate promiscuity. Indeed, some wild-type (wt) PEs have been shown to accept conservatively modified substrates.^21–29^ For example, Ogura and co-workers demonstrated that pumpkin^30–32^ or pig liver^33^ FPPS perform chain extensions with alkylated substrates while the groups of Allemann^34^ and Dickschat^15,35^ showed that bacterial FPPSs can accept alkylated extenders or hydroxylated starters. However, it has previously remained unclear how widespread promiscuity toward modified starters and/or extenders is among PEs or if that trait can be engineered for. Similarly, the kinetics of PEs with modified substrates and the structural determinants governing these transformations have remained unexplored.

Previously, PEs have suffered from limited experimental tractability due to two key challenges (Figure 1e). First, like many enzymes processing phosphorylated intermediates,^36^ PEs and other prenyltransferases are challenging to assay in high throughput. Established methods for the interrogation of these enzymes are generally discontinuous and labor-intensive,^37,38^ expensive,^39–41^ limited to a small substrate pool,^42–45^ or rely on radioactive substrate analogues^46–51^ (also see the literature examples cited above for various chromatographic approaches with dephosphorylated products of PEs). Second, their substrates, (iso-)­prenyl pyrophosphates, are generally difficult to synthesize since established methods are unreliable, low-yielding, poorly scalable, and incompatible with many functional motifs.^34,52,53^ In combination, these two challenges preclude the efficient engineering or directed evolution of PEs. Therefore, methods for the streamlined access to PE substrates and for the rapid interrogation of PEs would facilitate the tuning of these enzymes for desired properties and substrate scopes.

To address these gaps, we herein report on the profiling of PEs with a modular continuous high-throughput assay platform. This platform permits the facile and inexpensive interrogation of PEs with their native and modified substrates, starting from readily accessible prenyl monophosphates. We demonstrate its utility for the biochemical characterization of a panel of diverse PEs, their kinetic profiling with non-native substrates, and an exploratory engineering campaign of two distantly related PEs. Lastly, we demonstrate that wild-type PEs and their variants can be used to assemble modified prenoids at the (semi)­preparative scale.

## Biochemical Characterization of Diverse PEs

We began our study with a curated panel of 12 PEs. This panel included previously (partially) characterized and crystallized FPPSs^51,56,58–60^ as well as uncharacterized enzymes selected (i) for sequence diversity and (ii) to represent all kingdoms of life (Figure 2a). Despite the low sequence identity across the panel (mean <31%), these PEs all share the same overall protein structure and AlphaFold2 models indicate that they all possess the typical D-rich sequence motifs required for the binding of catalytically essential Mg^2+^.^19,20^ All of them could be produced in acceptable to good yields as soluble proteins in *Escherichia coli* (see the SI for further details and Tables S1 and S2 and Figure S1). Four PEs from our initial panel showed limited stability in our assay and storage buffers and were thus excluded from further analysis.

**Figure 2:**
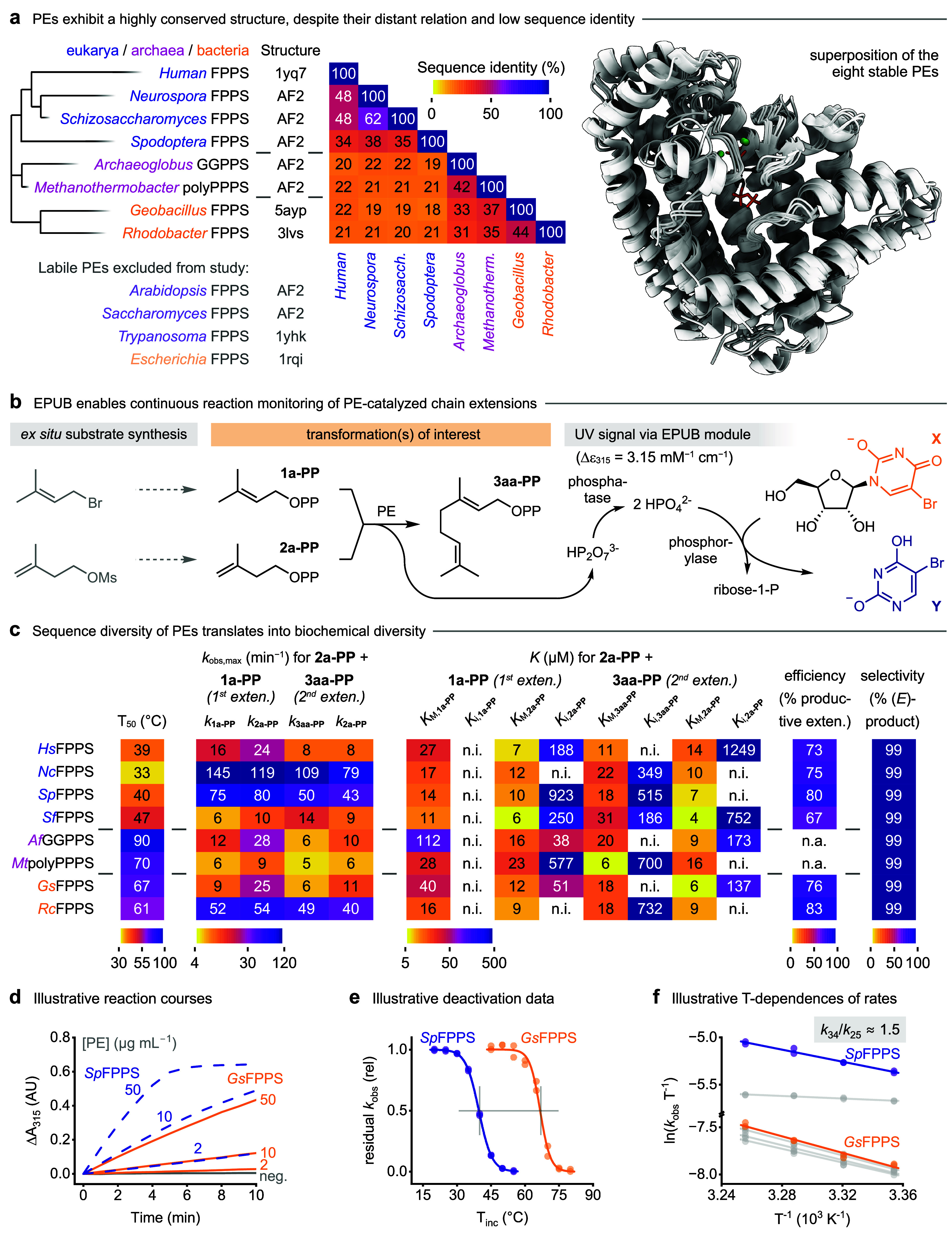
Biochemical characterization of diverse PEs with EPUB. (a) Sequence identities and structures of the PEs studied here. The structure column lists if a crystal structure is available in the PDB or if an AlphaFold2 model was used. For clarity, the superimposed structures were overlaid with the cocrystallized substrates and Mg^2+^ ions from the *E. coli* homologue (1rqi). (b) General reaction sequence for EPUB. (c) Stability, kinetics, efficiency, and selectivity of PEs. The kinetic experiments were performed under EPUB conditions (3 mM 5-bromouridine, 2 mM MgCl_2_, 3 mM (2-hydroxypropyl)-β-cyclodextrin, 1 mM TBA hydroxide, 20 μg mL^–1^ *Gt*PyNP, 0.5 μg mL^–1^ *Gt*IPP, in 150 mM taurine buffer with 1% Tween20, 4% glycerol, pH 9, 25 °C) with the excess substrate at 120 μM. Please see the SI for details on the enzymes (page 14 and following), characterization data for the EPUB assay enzymes (page 36 and following), and notes on the use of purified enzymes or lysates (page 114 and following). Efficiency and selectivity were determined using reactions with 0.6 mM **1a-P**, 0.6 mM **2a-P**, 3 mM glycerol-1-P, 3 mM ATP, 8 mM MgCl_2_, 2.4 μM (0.4 mol %) PE, 0.09 μM (1.5 μg mL^–1^) *Gt*IPP, and 4.8 μM (200 μg mL^–1^) *Af*G_3_PS in 50 mM taurine buffer, pH 9, 25 °C for 18 h. Then, *E. coli* phosphatase (as lysate) was added, the mixture was shaken for 24 h, extracted with CDCl_3_, and subjected to analysis as described in the SI (page 58 and following). Efficiency data for *Af*GGPPS and *Mt*polyPPPS could not be obtained (n.a.) since geranylgeranyl and polyprenyl glycerol ethers are not tractable with this method. (d–f) Illustrative data. The reactions in (d) were run under EPUB conditions with 200 μM **1a-PP** and 400 μM **2a-PP** with 2, 10, or 50 μg mL^–1^ PE. The incubations in (e) were performed in 10 mM taurine buffer pH 9 with 10% glycerol in intervals of 5 min. The reactions in (f) were performed under EPUB conditions at different temperatures. The data for all stable PEs are shown in (d) but only *Sp*FPPS and *Gs*FPPS are highlighted. Please see the SI page 53 and following for further details and the externally hosted Supporting Information for all raw data.^54^ Values exceeding the color scales are colored according to the nearest value within the color scale. FPPS = farnesyl pyrophosphate synthase, GGPPS = geranylgeranyl pyrophosphate synthase, polyPPPS = polyprenyl pyrophosphate synthase, n.i. = not inhibited, n.a. = not available.

For the initial kinetic characterization of the eight stable PEs, we synthesized the native substrates **1a-PP** and **2a-PP** as their tetrabutylammonium (TBA) salts^52,61^ and enlisted our previously developed extended module for phosphate detection by UV-spectroscopic monitoring of bromouridine phosphorolysis (EPUB) for reaction monitoring (Figure 2b).^36,61,62^ EPUB continuously tracks the release of inorganic pyrophosphate, the byproduct of every substrate turnover by PEs. EPUB employs an enzymatic hydrolysis of pyrophosphate to orthophosphate (using a pyrophosphatase) and subsequent enzymatic phosphorolysis of an acidic UV-active nucleoside analogue (using a nucleoside phosphorylase) whose conversion can be monitored at 315 nm.^63^

EPUB permits continuous high-throughput reaction monitoring of pyrophosphate-releasing transformations with any substrate(s). Since the pyrophosphatase and nucleoside phosphorylase we employed exhibit high activity and high affinity for their phosphorylated substrates, EPUB features no detectable equilibration phase and immediately reports on the rate of the transformation of interest (Figures S2 and S3). However, it should be noted that EPUB needs to be run in concentrated taurine buffer at pH 9 for maximum robustness and to ensure sufficient deprotonation of the chromophore, bromouracil (**Y**). Most PEs in our panel readily tolerated these conditions. Even the PEs which ultimately proved labile in taurine buffer displayed activity under EPUB conditions (Figure S2). Since EPUB is an indirect method and cannot differentiate between productive chain extension and hydrolysis, orthogonal experiments are required to exclude undesired side reactions. Therefore, we confirmed the efficiency and selectivity of our PE panel with a ^1^H NMR-based assay (described below) to ensure that EPUB reports accurately on the rates of PE-catalyzed chain extensions.

Using EPUB, we found that all PEs from our panel were active enzymes with striking differences in their biochemical properties (Figure 2c–f). Their stability varied greatly, with the eukaryotic PEs being more labile (*T*_50_ = 33–47 °C) than the bacterial and archaeal ones (*T*_50_ > 61 °C, Figure 2e). A detailed kinetic analysis of our PEs with the native substrates revealed marked differences in their kinetic parameters. The maximum observed rate constants spanned over an order of magnitude (*k*_obs,max_ = 6–145 min^–1^), with the two fungal enzymes (from *Neurospora crassa*, *Nc*FPPS, and *Schizosaccharomyces pombe*, *Sp*FPPS) standing out as the most active ones. Although one may assume that the differences in rates might be due to different temperature preferences of the thermo- and mesophilic enzymes in our panel, we found that these rate constants showed only a minor temperature-dependence (Figure 2f and Table S5). As such, the differences in rate largely seem to reflect overall catalytic proficiency rather than temperature preferences. We also found that some PEs showed strong inhibition by the extender unit **2a-PP**, while others lacked inhibition and instead followed regular Michael-Menten kinetics. For example, *Nc*FPPS showed the highest rate constants in our panel (*k*_obs,max_ ca. 130 min^–1^ for the chain extension of **1a-PP** with **2a-PP**) and was only mildly inhibited by the extender **2a-PP**. In contrast, the geranylgeranyl pyrophosphate synthase from *Archaeoglobus fulgidus* (*Af*GGPPS) acted much slower (*k*_obs,ma_x ca. 20 min^–1^ for the chain extension of **1a-PP** with **2a-PP**) and was strongly inhibited by **2a-PP**, resulting in reduced rates at substrate concentrations above 40 μM (see Figure S3 for representative kinetic plots). Five of the eight PEs in our panel also displayed product inhibition by the product of the first chain extension (**3aa-PP**), indicating that inhibitory phenomena are common among these enzymes. Although substrate and product inhibition of PEs has been reported previously,^64,65^ its prevalence across this enzyme family has previously remained elusive.

Overall, our kinetic data for the characterized PEs are comparable with literature data, although direct comparisons are difficult since previous reports used different purification workflows, reaction conditions, and analytical methods. Our kinetic data (*K*_M_ = 18 μM for **3aa-PP** and 6 μM for **2a-PP**, *k*_obs,max_ = 5 min^–1^) for the FPPS from *Geobacillus stearothermophilus* (*Gs*FPPS) are similar to the values reported by Koyama and colleagues (*K*_M_ = 12 μM for **3aa-PP** and 10 μM for **2a-PP**, *k*_obs,max_ = 2 min^–1^).^66^ In contrast, our rate constants for the human FPPS (*Hs*FPPS, *k*_obs,max_ = 16 min^–1^ for **1a-PP** and 8 min^–1^ for **3aa-PP**) differ from those reported by Poulter and colleagues (*k*_obs,max_ = 0.5 min^–1^ for the net reaction from **1a-PP** to **4aaa-PP**).^67^ The affinity values for this enzyme are not comparable since Poulter and colleagues only used substrate concentrations ≤ 2 μM for their kinetic studies. The discrepancies between our values and these prior reports likely arise from differences in the reaction conditions. For example, the studies by the Koyama and Poulter groups used Tris buffer at pH 8.5 or 7.6 while our assay system requires taurine buffer at pH 9. In addition, neither prior study on *Gs*FPPS or *Hs*FPPS reported substrate inhibition, while we found both enzymes to be inhibited by the extender **2a-PP**. To the best of our knowledge, the FPPS from *Rhodobacter capsulatus* (*Rc*FPPS) has been crystallized but not kinetically characterized while the other five PEs in our panel have not been characterized previously.

Since EPUB is not able to differentiate between successful chain elongation and hydrolysis, we used an NMR-based assay to assess the selectivity and efficiency of our PEs. To this end, we coupled chain extension by a PE to prenyl transfer mediated by the archaeal ether synthase *Af*G_3_PS^61^ and subsequent global dephosphorylation by the *E. coli* alkaline phosphatase, which nonspecifically hydrolyses organophosphates. This yielded prenyl ethers which can be easily identified by ^1^H NMR. This approach relies on the selectivity of *Af*G_3_PS for chain-extended prenyl pyrophosphates. *Af*G_3_PS is inactive on the starter units (Figure S4) but transfers both (*E*)- and (*Z*)-configured prenyl pyrophosphates to a glycerol unit while retaining the configuration of the double bond at the headgroup. This in situ enzymatic derivatization allowed a discrimination between unsuccessful chain extensions (yielding prenols) and successful ones (yielded glycerol ethers) while reporting on the configuration of the double bond(s) formed during chain extension. Using this method, we found that all of our PEs exhibited (*E*)-selectivity with the native substrates (Figures 2c and S5, Table S8). Compared to other prenyltransferases like archeal ether synthases which convert their substrates with >98% efficiency,^61^ our PEs proved comparably inefficient under the chosen reaction conditions with only 70–80% of all substrate turnovers resulting in productive chain extension and the rest leading to futile hydrolysis (Table S8). Since our PEs do not measurably hydrolyze their substrates but do slowly hydrolyze their chain-extended products, the hydrolytic side reaction likely occurs during an unsuccessful additional chain extension prior to product release or after rebinding of the product. In line with these hypotheses, the inhibition of many PEs by their reaction products (Figure 2c) suggests that product release is a slow process and can be rate-limiting for these enzymes.

## Profiling of PEs with Modified Substrates

Next, we sought to assess the promiscuity of our PEs by profiling them with a panel of modified analogues of the starter and extender unit. We selected these analogues to be sterically diverse and electronically distinct from the native substrates by including various alkoxy tails on the methyl groups of **1a-PP** and **2a-PP** (Figure 3b). To access these analogues, we prepared their corresponding alcohol precursors in two consecutive telescoped reaction sequences inspired by procedures disclosed by the groups of Alexandrov^68^ and Distefano and co-workers.^18^ Since established alcohol activation strategies proved impractical for the direct synthesis of the pyrophosphates (see Scheme S2), we employed an alternative route via monophosphates.

**Figure 3:**
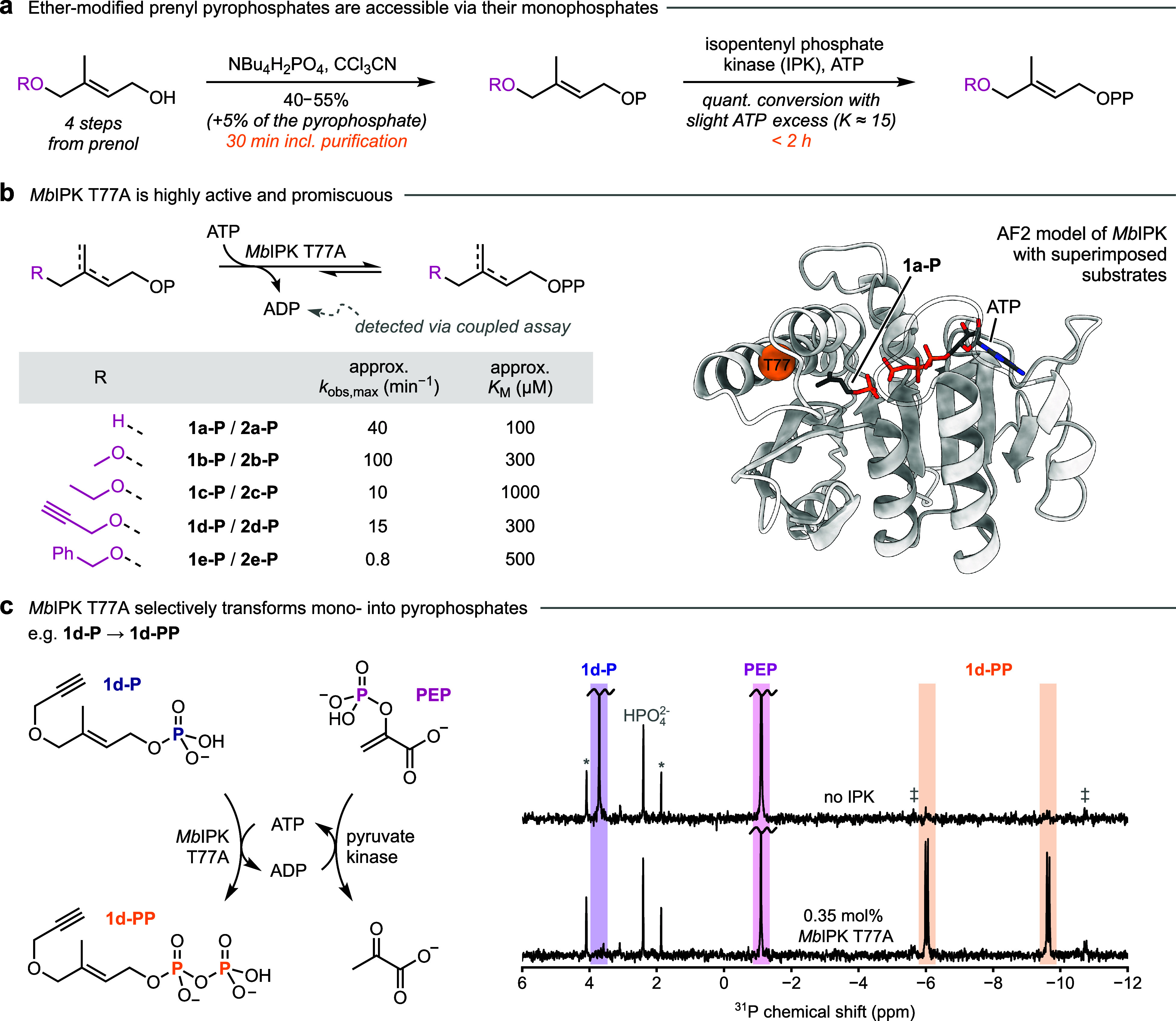
Chemoenzymatic synthesis of modified prenyl pyrophosphates. (a) Synthesis of pyrophosphates via easily accessible monophosphates. (b) Substrate scope of the rationally engineered T77A variant of *Mb*IPK. The rates were obtained from reactions with 0.8 mM PEP, 0.2 mM NADH, 0.4 mM ATP, 10 mM MgCl_2_, 50 mM KCl, 100 μg mL^–1^ *Gs*LDH, and 50 μg mL^–1^ *Gs*PK in 50 mM taurine buffer with 4% glycerol, pH 9, 25 °C. Note that this coupled assay used to measure IPK rates by ADP detection generally underestimates rate constants. Under the conditions we used, this assay employing a pyruvate kinase and a lactate dehydrogenase features lag times due to intermediate accumulation resulting from unfavorable kinetic properties at pH 9 (see the SI page 39 and following). Hence all kinetic parameters listed here are only estimates and given as approximate values. The rates for the prenyl and isoprenyl analogues are essentially identical. The AF2 model of *Mb*IPK is shown as a superposition with the cocrystallized substrates from a homologue (PDB ID 3lkk). The unstructured C-terminus of *Mb*IPK was omitted for clarity and the flexible loop covering the ATP binding site is shown with 80% transparency. (c) Selectivity of *Mb*IPK T77A. This reaction is a representative example with the others shown in Figures S8 and S9. This example was performed with 1 mM **1d-P**, 2 mM PEP, 0.1 mM ATP, 5 mM MgCl_2_, 50 mM KCl, and 100 μg mL^–1^ *Gs*PK in 50 mM taurine buffer with 10% D_2_O and 4% glycerol, pH 9, 25 °C. The asterisks indicate an unknown impurity present in our commercial preparation of PEP (ca. 4 ppm) and the phosphodiester of **1d-P** which is a typical impurity during its synthesis by condensation (ca. 2 ppm). The double daggers indicate the α- and γ-phosphorus of ATP (ca. −11 and −6 ppm). Please see the SI for further details and the externally hosted Supporting Information for all raw data.^54^ The abbreviation P indicates a monophosphate unit.

Motivated by reports from the Noel,^69^ Poulter,^70–72^ Williams^73,74^ and Singh^75,76^ groups on the relaxed substrate scopes of isopentenyl phosphate kinases (IPKs), we accessed modified (iso-)­prenyl pyrophosphates by chemical synthesis of the monophosphates, followed by enzymatic phosphorylation (Figure 3a). As such, we prepared the monophosphates of the analogue panel by kinetically controlled condensation^61,77^ and used a rationally engineered IPK variant for their phosphorylation.

We found that monophosphates could be readily obtained by condensing TBA phosphate and an alcohol in the presence of trichloroacetonitrile, followed by a filtration of the crude product through a silica plug. These syntheses generally took around 30 min, including setup, reaction, workup, and purification (see the SI page 85 and following). Although pyrophosphates can be obtained by the same strategy with longer reaction times, this condensation is not selective and gives pyrophosphates only in a mixture with the mono- and triphosphate, along other condensation products (see the SI page 90 for a discussion on byproducts and their identification by ^31^P NMR).

Our rationally engineered IPK variant was based on prior reports from the Singh lab. They showed that the IPK from *Methanosarcina barkeri* 3 (*Mb*IPK) has trace activity with the propargyl analogue **1d-P**.^76^ In addition, they demonstrated that active-site variants of other IPK homologues can have expanded promiscuity.^75^ As such, we introduced an analogous active-site substitution in *Mb*IPK to improve its activity with **1d-P** and related substrates. The resulting variant *Mb*IPK T77A features a moderately enlarged active site pocket and could readily be produced and purified in high yield (Table S1).

Using a coupled enzymatic assay detecting ADP production (Figures S4–S6),^69,75,76^ we found that *Mb*IPK T77A accepted all substrate analogues in our panel (Figure 3b). For example, it retained activity with its native substrate **2a-P** (*k*_obs,max_ ≈ 40 min^–1^) and showed useful activity with larger substrates such as **1d-P** (15 min^–1^) or **1e-P** (0.8 min^–1^). Although similar substrates have previously been shown to be accepted by IPKs, only **1d-P** constitutes a known analogue.^75,76^ It is worth noting that **1e-P** was accepted by *Mb*IPK T77A even though this analogue is almost isosteric to geranyl monophosphate which is generally not accepted by IPKs. Next, we analyzed reaction mixtures by ^31^P NMR, confirming that *Mb*IPK T77A is selective for the desired phosphorylation (Figures 3c, S8, and S9) with all substrates analogues we tested. Potential side reactions such as overphosphorylation were not observed. Further analysis of equilibrium states showed that the ATP-dependent monophosphate→pyrophosphate transformation is moderately exergonic (*K* ≈ 15, Figure S6), enabling essentially quantitative conversion with a small excess of ATP. *Mb*IPK T77A also readily tolerated the previously optimized EPUB conditions and converted (iso-)­prenyl monophosphates in these complex reaction mixtures (Figure S7). As such, a short preincubation of typical EPUB reaction mixtures containing monophosphate substrate, ATP, and *Mb*IPK T77A provided quantitative conversion to the pyrophosphate substrates in situ. Subsequent addition of PE then enabled continuous monitoring of PE-mediated chain extensions with modified starter or extender units.

Using IPK-mediated in situ phosphorylation in combination with EPUB (Figure 4a), we found that our PEs exhibit pronounced tolerance to substitutions on the starter unit and a narrow substrate scope for the extender unit. Our PEs only exhibited trace activity with the methoxy-modified extender **2b-PP** and were inactive with the other extenders. In contrast, all our PEs converted at least one modified starter (Figure 4b and Table S8). While the bacterial and archaeal PEs in our panel generally only converted the benzoxy starter **1e-PP**, the eukaryotic PEs proved more promiscuous and enabled extension of all modified starters. Although the observed rate constants were typically at least 1 order of magnitude lower than with the native substrates, many of the PEs showed rate constants of >0.2 min^–1^ with these analogues. As a general trend, increased steric bulk and rigidity of the ether substituent correlated with tighter binding but had comparably little effect on the rate constant.

**Figure 4:**
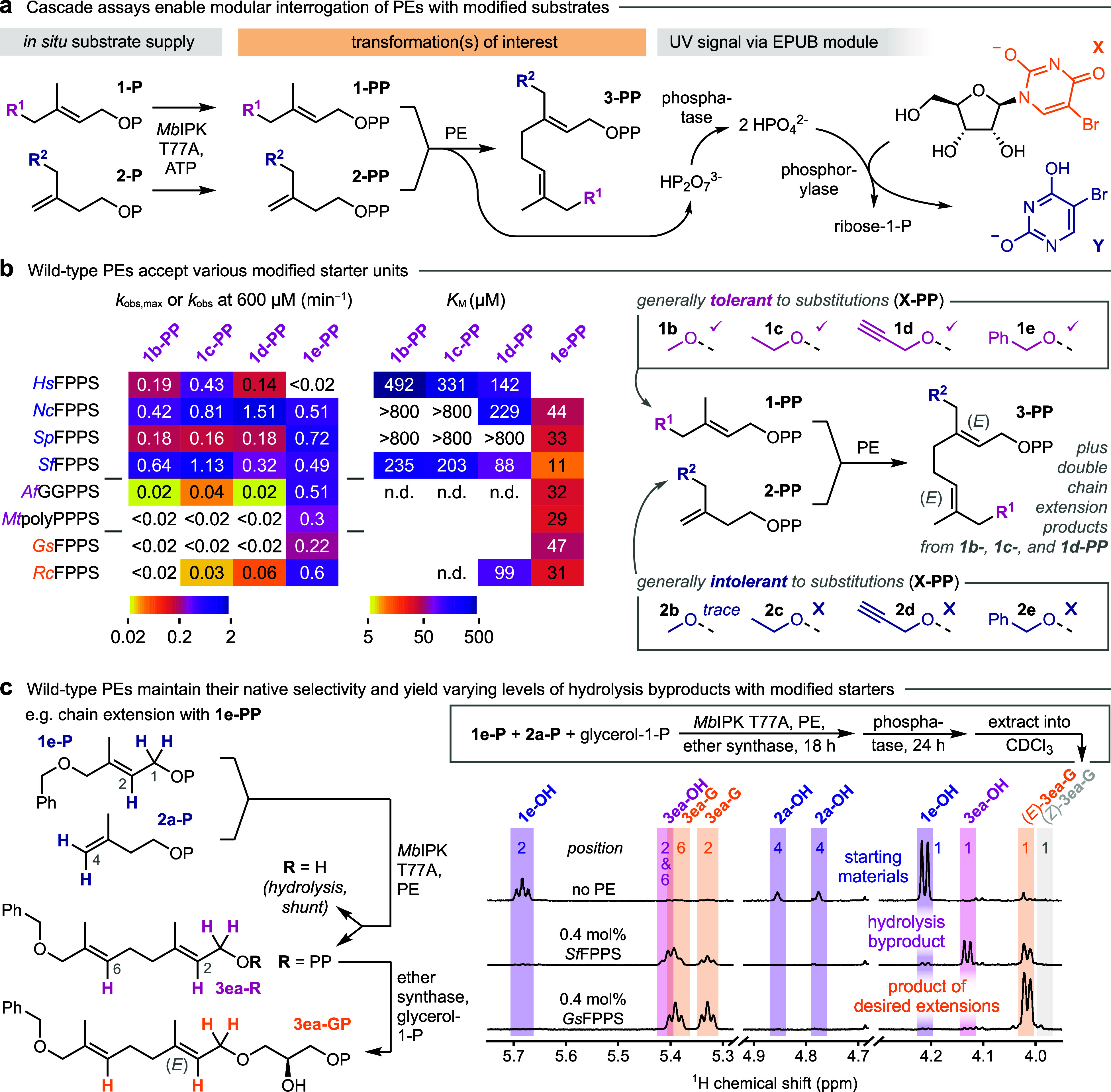
Profiling of PEs with modified substrates. (a) In situ substrate supply coupled to the transformation of interest and EPUB enables continuous high-throughput kinetic experimentation with modified (iso-)­prenyl monophosphates. (b) Performance of wt PEs with modified substrates. The monophosphate precursors were quantitatively converted to their pyrophosphates prior to the PE-catalyzed transformation. To ensure complete pyrophosphorylation, parallel experiments with different *Mb*IPK T77A concentrations were performed (see SI page 52). Most PEs were active with modified starters and inactive with modified extenders. All kinetic experiments shown here were carried out under EPUB conditions (see caption of Figure 2 and SI page 43 and following) with [**2a-PP**] = 150 μM. For *K*_M_ < 800 μM, *k*_obs,max_ values are given, for *K*_M_ > 800 μM, *k*_obs_ values at 600 μM starter are given (also see Table S6). The data in the heatmap were qualitatively confirmed by ^1^H NMR analysis of derivatized products (see SI page 56 and following). Values exceeding the color scales are colored according to the nearest value within the color scale. (c) Illustrative ^1^H NMR data of dephosphorylized reaction products of two FPPSs following in situ etherification with *Af*G_3_PS. Additional examples are shown in Figure S5 and summarized in Table S8. The example shown here was performed with 0.6 mM **1e-P**, 0.6 mM **2a-P**, 3 mM glycerol-1-P, 3 mM ATP, 8 mM MgCl_2_, 2.4 μM (0.4 mol %) PE, 0.09 μM (1.5 μg mL^–1^) *Gt*IPP, and 4.8 μM (200 μg mL^–1^) *Af*G_3_PS in 50 mM taurine buffer, pH 9, 25 °C for 18 h. Then, *E. coli* phosphatase (as lysate) was added, the mixture was shaken for 24 h and then extracted with CDCl_3_ (see SI page 58 for additional details). *Gs*FPPS selectively and quantitatively produces the intact chain-extended product **3ea-PP** which undergoes derivatization to the glycerol ether. *Sf*FPPS also produces a hydrolysis byproduct after the chain extension, yielding a dephosphorylated shunt product **3ea–OH** which does not undergo derivatization. Glycerol-1-phosphate was used as racemic mixture but only the *R*-enantiomer is used by the ether synthase *Af*G_3_PS. This ether synthase is selective for chain-extended prenyl pyrophosphates and does not convert any of the ether-modified building blocks of the type **1-PP** used in this study (Figure S4). Dephosphorylated glycerol ethers such as **3ea-G** have characteristic chemical shifts for H1 for the (*E*)- and (*Z*)-isomer (Table S7). Please see the SI for further details and the externally hosted Supporting Information for all raw data.^54^ n.d. = not determined.

We again orthogonally interrogated the activity, efficiency and selectivity of these PEs with the starter analogues by ^1^H NMR analysis of their glycerol ethers arising from in situ enzymatic derivatization (Figure S5, Tables S7 and S8). To this end, all glycerol ethers of the chain-extended prenyl pyrophosphate analogues were first produced on a semipreparative scale and fully characterized (SI page 109 and following). Using the ^1^H NMR assay, we found that all PEs retained their native (*E*)-selectivity with the modified starter units, although they showed drastic differences in their efficiency. For example (Figure 4c), with the benzoxy-analogue **1e-PP**, the FPPS from *Spodoptera frugiperda* (*Sf*FPPS) gave nearly equal amounts of hydrolysis product (arising from unsuccessful chain extension) and desired pyrophosphate **3ea-PP** (which yielded the glycerol ether **3ea-G** after etherification and dephosphorylation). In contrast, *Gs*FPPS performed the same chain extension with 98% efficiency. This detailed profiling confirms the widespread promiscuity of PEs, which accept heteroatom-bearing substrates of various size while maintaining their native selectivity.

## Tuning PEs by Semirational Engineering

Next, we examined if the promiscuity and biochemical characteristics of PEs could be tuned by protein engineering. As such an endeavor also presented an ideal bench test for the utility of our assay platform, we pursued an exploratory engineering campaign of two distantly related PEs. To the best of our knowledge, the engineering of PEs for the acceptance of modified substrates is an unmet challenge, likely due to the difficulties concerning synthesis and high-throughput experimentation described above.

Based on our initial biochemical characterization, we selected the fungal *Sp*FPPS and the bacterial *Gs*FPPS. Although the two enzymes perform the same net transformation with the same selectivity, they share only 19% sequence identity and differ in every biochemical and kinetic characteristic we examined (Figure 2c) and their substrate scope (Figure 4b). They also differ in the identity of four nonconserved residues (NCRs) in the otherwise highly conserved active site (NCR**I**–**IV**, Figure 5a). As cocrystal structures indicate,^56^ NCR**I** points away from the bound substrates and positions a helix bearing catalytically essential residues, NCR**II** and NCR**III** form a sterically balanced pair (generally two medium-sized residues or one smaller and one larger residue) that modulates the volume of the active site, and NCR**IV** forms part of the active site floor and points toward the extender unit in the catalytic ensemble. In wt PEs, these four NCRs are generally either medium-sized polar (S, T, N) or nonpolar amino acids (L, I, F, Figure 5a, Table S17). We thus hypothesized that NCR substitutions in either *Sp*FPPS or *Gs*FPPS may yield variants with altered biochemical properties, substrate scopes and/or selectivity. As such, we diversified NCR**I**–**IV** individually in both PEs using degenerate primers and assembled a library of defined (sequenced) but randomly chosen variants of both enzymes featuring diverse substitutions (see SI page 62 and following for additional details on the PE variants).

**Figure 5:**
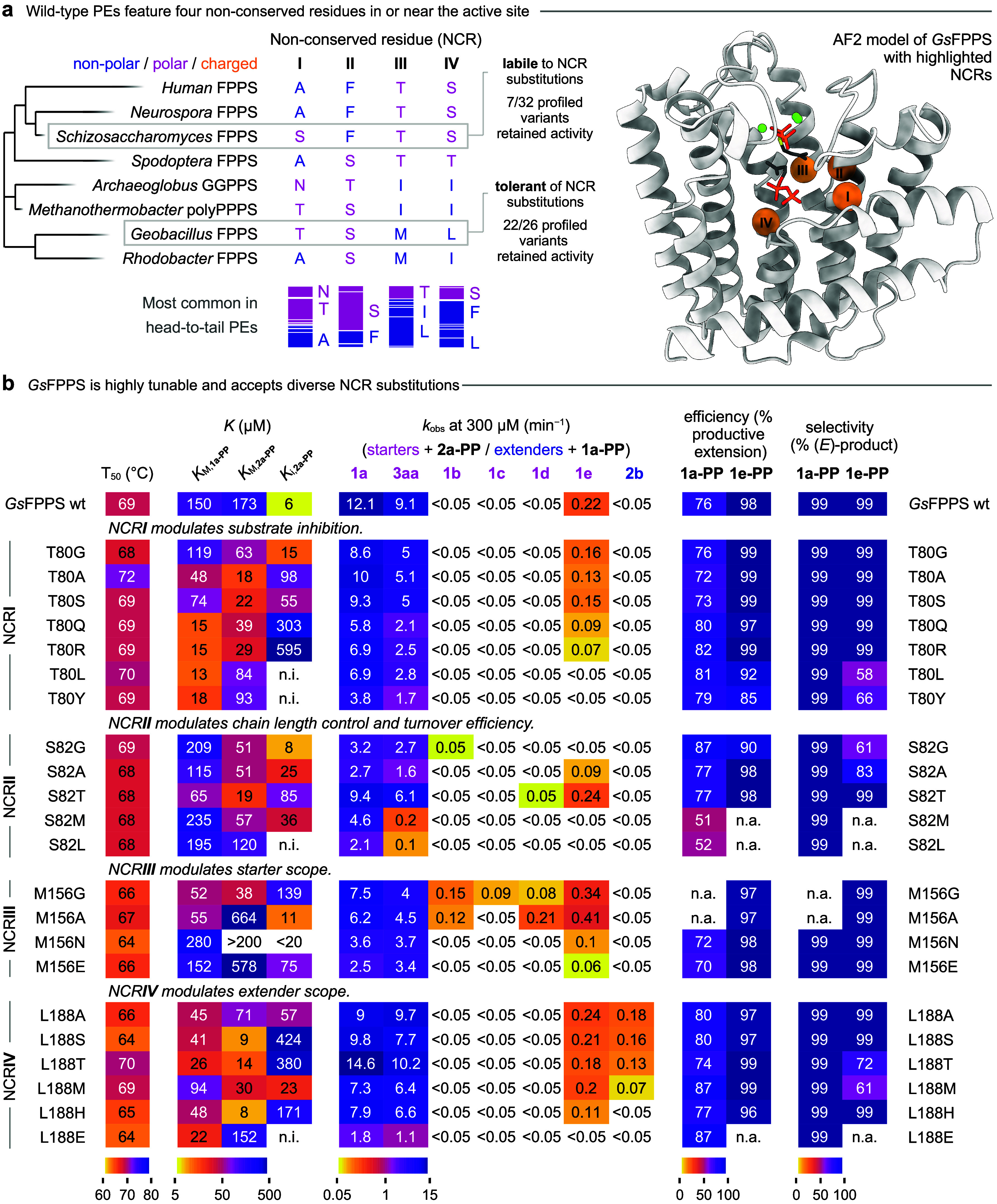
Diversification of PEs and profiling of variants. **a** Diversity of nonconserved active site residues across our PE panel and their distribution in 166 representative head-to-tail PEs from the Interpro database (listed in the SI and available for download from the externally hosted Supporting Information).^54^ In *Gs*FPPS, NCR**I** is T80, NCR**II** is S82, NCR**III** is M156 and NCR**IV** is L188. **b** Profiling of *Gs*FPPS variants (also see Tables S12–S15). The thermal stability was assessed by incubating each variant in 10 mM taurine buffer with 10% glycerol, pH 9, in intervals of 5 min and measuring residual activity. The kinetic parameters with the native substrates were obtained under EPUB conditions (see caption of Figure 1) with the excess substrate at 120 μM. The rates with substrate analogues were obtained from reactions under EPUB conditions with 300 μM starter precursor and 150 μM **2a-P** or with 300 μM **2b-P** and 150 μM **1a-P**. The efficiency and selectivity were determined under identical conditions as those as in Figure 4c. Also see SI page 64 and following for further experimental details. The *k*_obs_ values represent median values of three kinetic experiments with different PE concentrations. Values exceeding the color scales are colored according to the nearest value within the color scale. Note that the stability and kinetic parameters of wt *Gs*FPPS shown here differ slightly from those shown in Figure 2c because these data were obtained under different reaction conditions. *Gs*FPPS M156G and M156A likely perform three chain extensions with the native substrates, yielding geranylgeranyl pyrophosphate, whose corresponding glycerol ether is not tractable by this method. The S82 M variant also features a D29E substitution at the protein surface due to a serendipitous mutation. Fits of the kinetic data for the M156N variant did not converge due to parameter codependency. Please see the SI for further details and the externally hosted Supporting Information for all raw data.^54^ n.i. = not inhibited, n.a. = not available.

Next, we tested the resulting 32 *Sp*FPPS variants and 26 *Gs*FPPS variants for activity with the native substrates **1a-PP** and **2a-PP** and subjected all active variants to a detailed characterization. The active variants were (i) kinetically characterized with the native substrates, (ii) profiled with the panel of substrate analogues by EPUB, and (iii) assessed for the retention of selectivity by ^1^H NMR. We found that the two PEs responded starkly differently to NCR substitutions, with *Gs*FPPS being tolerant and tunable and *Sp*FPPS being generally intolerant. Out of 32 *Sp*FPPS variants, only seven retained measurable activity. Those seven largely featured conservative substitutions (T→S, S→T, F→I) with only NCR**I** (S89 in *Sp*FPPS) allowing some flexibility (Table S10). The active *Sp*FPPS variants generally behaved similarly to the parent enzyme by retaining comparable biochemical characteristics, substrate scope and activity levels (Tables S12–S15). In contrast, out of 26 *Gs*FPPS variants, 22 retained activity. Contrary to our expectation, these active variants featured several examples of substitutions to bulky and/or charged residues (e.g., T→R, M→E, L→H, Figure 5b), which are rare or absent from these positions in wt PEs (Figure 5a, Table S17).

Profiling the active *Gs*FPPS variants elucidated possible roles of each NCR in catalysis (Figure 5b, Tables S12–S15): (i) In *Gs*FPPS, NCR**I** (T80) influences substrate inhibition. Several diverse substitutions were tolerated at this position and caused only minor losses of activity in the native transformation. Substitutions to larger polar or charged residues (T80Q or T80R) led to improved substrate binding and reduced inhibition, albeit at the cost of overall rate. Combined with the observation that some PEs do not display substrate inhibition, this suggests that substrate inhibition of *Gs*FPPS and similar PEs has some biological relevance,^78^ potentially in controlling metabolic flux in terpenoid secondary metabolism. (ii) NCR**II** (S82) influences the chain length selectivity by affecting overall turnover efficiency past the first chain extension. Substitutions to bulky residues (S82L or S82M) led to a complete loss of activity with geranyl pyrophosphate (**3aa-PP**), terminating chain extension after one elongation event. The S82 variants also generally exhibited low activity. For example, the S82G and S82A variants were ca. 5-fold less active than the parent, indicating the need for a fine steric balance at this position and/or hydrogen bonding interactions. (iii) NCR**III** (M156) influences the starter substrate scope. Substitutions to smaller residues (M156G or M156A) unlocked activity with the full panel of starter analogues, including **1b-**, **1c-**, and **1d-PP** which wt *Gs*FPPS failed to convert. (iv) NCR**IV** (L188) influences the extender scope. Substitution to smaller residues (L188A or L188S) enabled the conversion of the methoxy-modified extender **2b-PP** which almost all wt PEs in our panel failed to use as an extender.

This profiling campaign also highlighted that PEs need to orchestrate productive chain extensions with a high degree of steric precision. Several *Gs*FPPS variants illustrate possible outcomes of perturbations to this balance. For example, the T80L and T80Y variants were active but showed poor rates, efficiency, and selectivity, likely by reducing space and flexibility in the active site. Similarly, substitutions of S82 to much smaller (S82G) or larger residues (S82L) generally impaired efficiency and selectivity, potentially by allowing nonproductive substrate binding poses.

Collectively, these data show that almost all biochemical properties of PEs can be tuned by targeted single substitutions, although some PEs appear to be more tolerant of substitutions than others. One may expect that *Sp*FPPS’s general intolerance to substitutions was a result of its lower thermostability. However, we generally observed only small changes to T_50_ across the active *Sp*FPPS and *Gs*FPPS variants (Figure 5b, Table S12), although almost all variants either retained or lost stability. As such, our data suggest that even seemingly conservative substitutions in a PE can effect large changes in its behavior and characteristics, which can be profiled rapidly with coupled assays as demonstrated here. However, it should be noted that our analysis only covered a small section of the possible sequence space of all possible combinations of the NCRs and that the different response of *Gs*FPPS and *Sp*FPPS to NCR substitutions suggests a high degree of context dependency of the observed effects.

## Biocatalytic Assembly of Modified Prenoids

Lastly, we sought to probe the synthetic utility of (engineered) PEs for the assembly of modified prenoids. Toward this end, we prepared derivatives of the modified long-chain prenyl pyrophosphates accessible by our PEs in a one-pot fashion from their monophosphate building blocks. For example, under nonoptimized conditions and with <2 mol % loading of all enzymes, the modified starter **1e-P** and the native extender **2a-P** could be phosphorylated by *Mb*IPK T77A, assembled by wt *Sp*FPPS, and then transferred onto a glycerol unit by the archaeal ether synthase *Af*G_3_PS.^61^ Subsequent dephosphorylation by *E. coli* alkaline phosphatase yielded the benzoxy-protected archaeal membrane lipid **3ea-G** as a single isomer (Figure 6). This compound and all other analogues were also isolated and characterized to confirm their structure (see SI page 109 and following). To test the scalability of this transformation, we made **3ea-G** from **1e-P** (47 mg, 0.07 mmol) as the limiting reagent, giving 13 mg (56% isolated yield) of the prenyl ether **3ea-G**. Under comparable conditions at the semipreparative scale, the native starter **1a-P** and the modified extender **2b-P** could be phosphorylated, assembled by the *Gs*FPPS L188A variant and then dephosphorylated to yield the methoxy-modified geraniol **3ab–OH** in excellent selectivity and good purity after extraction. This analogue represents a methylated analogue of the bioactive monoterpenoid α-acaridiol.^81^ Similar analogues are accessible from bioprocesses^82,83^ or through de novo synthesis,^84,85^ but often only as mixture of isomers. These cascades relied on the excellent selectivity and cross-compatibility of IPKs, PEs and other prenyltransferases and highlight the great potential for modular prenoid assembly to provide otherwise inaccessible products as well as activated intermediates primed for further elaboration. As such, we anticipate that a range of other prenoids and prenylated biomolecules (and their analogues) will be accessible via the enzymes and cascades outlined here.

**Figure 6:**
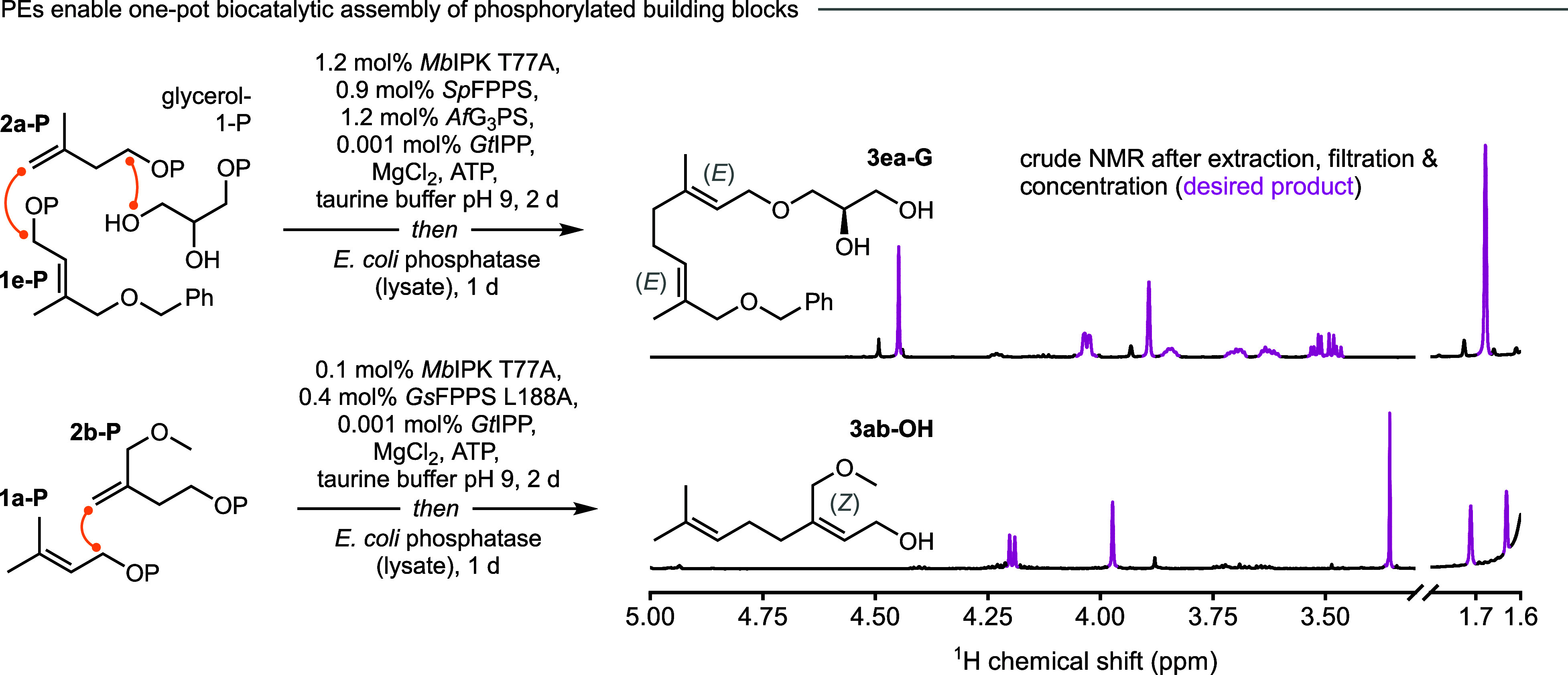
Biocatalytic assembly of modified prenoids and illustrative crude NMR spectra. These reactions were performed with 0.6 mM (iso-)­prenyl monophosphates, 3 mM ATP, 8 mM MgCl_2_, in 50 mM taurine buffer pH 9 with enzymes as indicated in a total volume of 5 or 20 mL. The reaction toward **3ea-G** additionally contained 2 mM *rac*-glycerol-1-phosphate. Please see the SI for further details and the externally hosted Supporting Information for all raw data.^54^

## Conclusions

We have demonstrated the modular profiling of PEs to enable a biocatalytic assembly of modified prenoids. PEs have previously remained underexplored with respect to their biochemical properties, kinetics, substrate scopes, and potential for protein engineering. Using EPUB in combination with in situ access to pyrophosphate substrates through a promiscuous IPK enabled a rapid interrogation of a diverse spectrum of PEs and their variants. Our work demonstrates that some wt PEs already provide access to modified prenoids while the methods and biochemical data herein will facilitate their engineering toward expanded substrate scopes. We expect that these advances will facilitate biocatalytic access to modified prenoids, prenylated biomolecules and terpenoids.^86,87^

## Methods

Experimental details, synthetic procedures, compound characterization data, and additional biochemical data are available from the Supporting Information.

## Supplementary Material



## Notes

Experimental details and methods, additional data for all enzymes reported herein, synthetic procedures, and characterization data for all compounds prepared for this study (PDF)
