# Supplementary material for: High-Throughput Profiling of Prenylelongases Enables the Assembly of Modified Prenoids
Source: ACS Chem Biol. 2026 Jun 15;21(7):1705–17. doi: 10.1021/acschembio.6c00250 (PMC13386471; doi:10.1021/acschembio.6c00250)
Supplement: Supplementary file 1 [file cb6c00250_si_001.pdf]

High-Throughput Profiling of Prenylelongases Enables the Assembly of Modified Prenoids

Supporting Information

Hui Li,[a] Anjali Sital,[a] Clemens Mayer,[a] Felix Kaspar [a,b,c,*]

[a] Biomolecular Chemistry & Catalysis, Stratingh Institute for Chemistry, University of Groningen, Nijenborgh 4, 9747 AG Groningen, The Netherlands.
[b] Organic Chemistry, Saarland University, 66123 Saarbrücken, Germany; felix.kaspar@uni-saarland.de
[c] Department of Natural Product Biotechnology, Helmholtz Institute for Pharmaceutical Research Saarland (HIPS), Helmholtz Centre for Infection Research (HZI) and Department of Pharmacy at Saarland University, PharmaScienceHub (PSH), Campus E8.1, 66123 Saarbrücken, Germany.

Table of Contents

Supplementary figures referenced in the main text 3
Author contributions 10
Data availability 10
Accessibility statement 10
Safety and sustainability considerations 10
Biochemistry 10
General remarks (biochemistry) 10
Cloning 11
Enzymes 14
Routine protein production and purification 33
Characterization of the EPUB enzymes 36
Characterization of the ARK enzymes 39
Characterization of *Mb*IPK T77A 42
Equilibrium states of (iso-)prenyl mono-to-phosphorylation 48
Characterization of PEs – Kinetic experiments with EPUB and initial activity tests 49
Thermal stability of PEs 50
Michaelis-Menten kinetics with PEs and their native substrates 52
Temperature-dependence of rates of PE-mediated chain extensions 53
Screening of PEs with modified substrates 54
Kinetic characterization of PEs with modified substrates 56
Interrogation of the selectivity and efficiency of PEs by NMR 58
Selectivity of *Af*G$_3$PS for chain-extended prenyl pyrophosphates 61
Mutagenesis and initial screening of *Gs*FPPS and *Sp*FPPS variants 62
Production, purification, and characterization of the GsFPPS and SpFPPS variants 64
Bioinformatic analyses 73

Synthetic procedures	74
General remark (synthetic chemistry)	74
Numbering of compounds	74
Notes on failed routes	76
Synthesis of the native pyrophosphate substrates	79
Synthesis of key branchpoint intermediates	83
Synthesis of monophosphate substrates	85
Semi-preparative biotransformations	109
Additional discussion	114
Supplementary references	116

Supplementary figures referenced in the main text

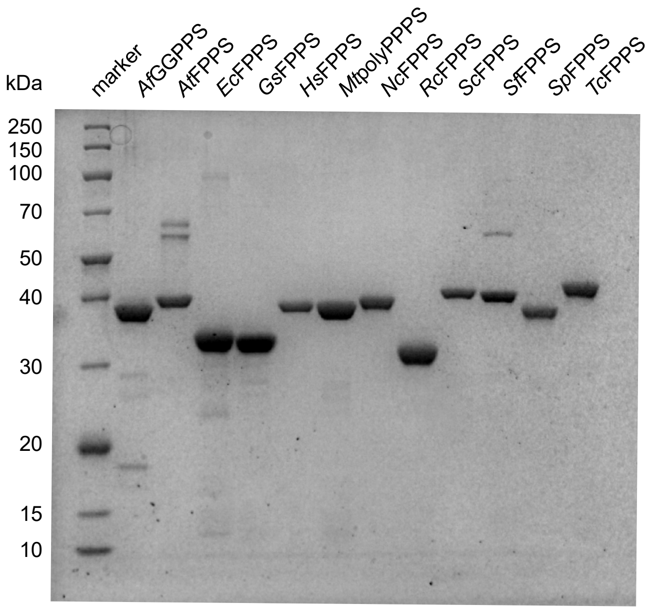

Figure S1. SDS PAGE analysis of purified PEs, using precast SDS PAGE gels (SurePAGE™, Bis-Tris, 10x8, 12%, 15 wells) and Tris-MOPS-SDS Running Buffer from GenScript (USA).

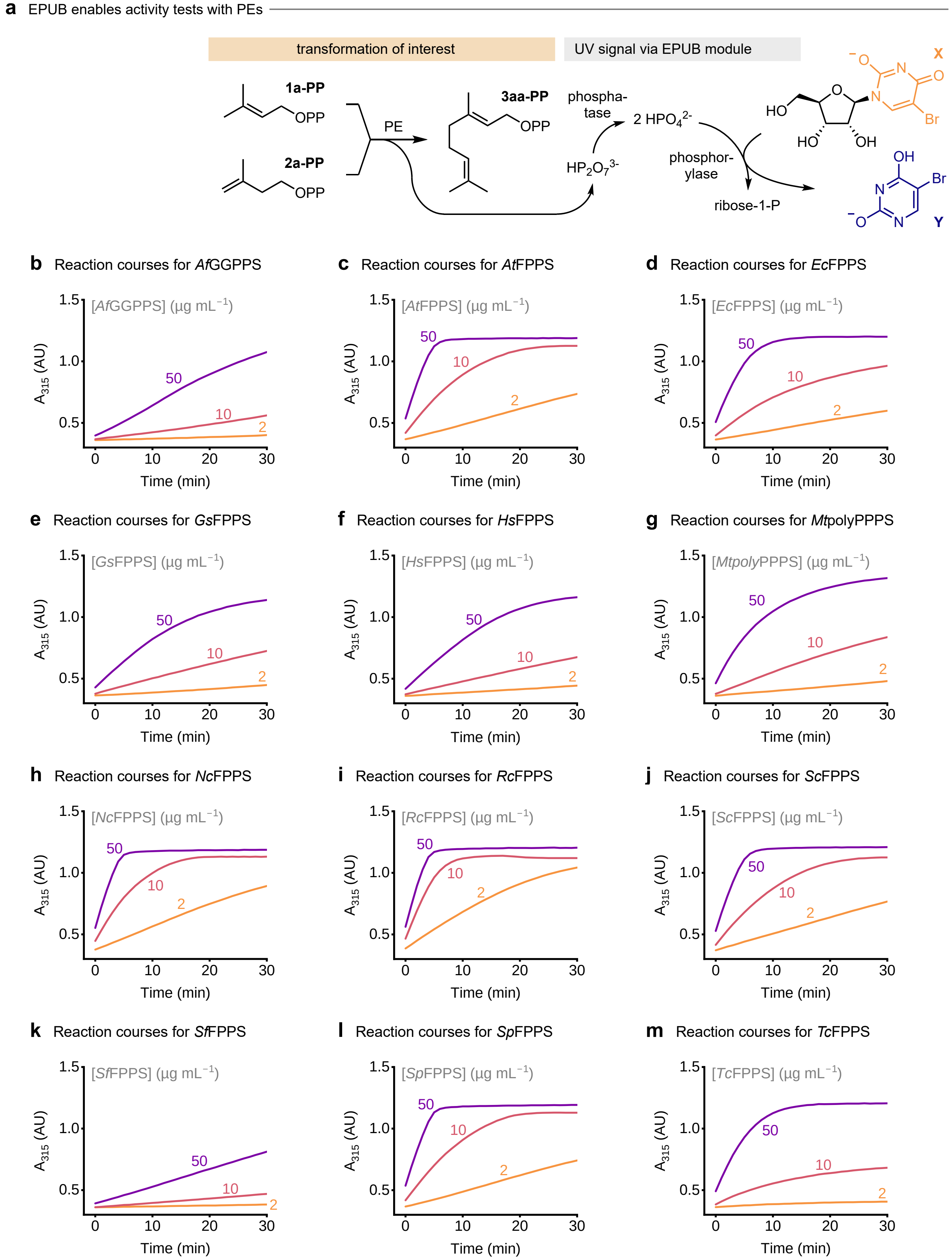

Figure S2. Initial activity tests with the full panel of PEs, using EPUB and the native substrates.

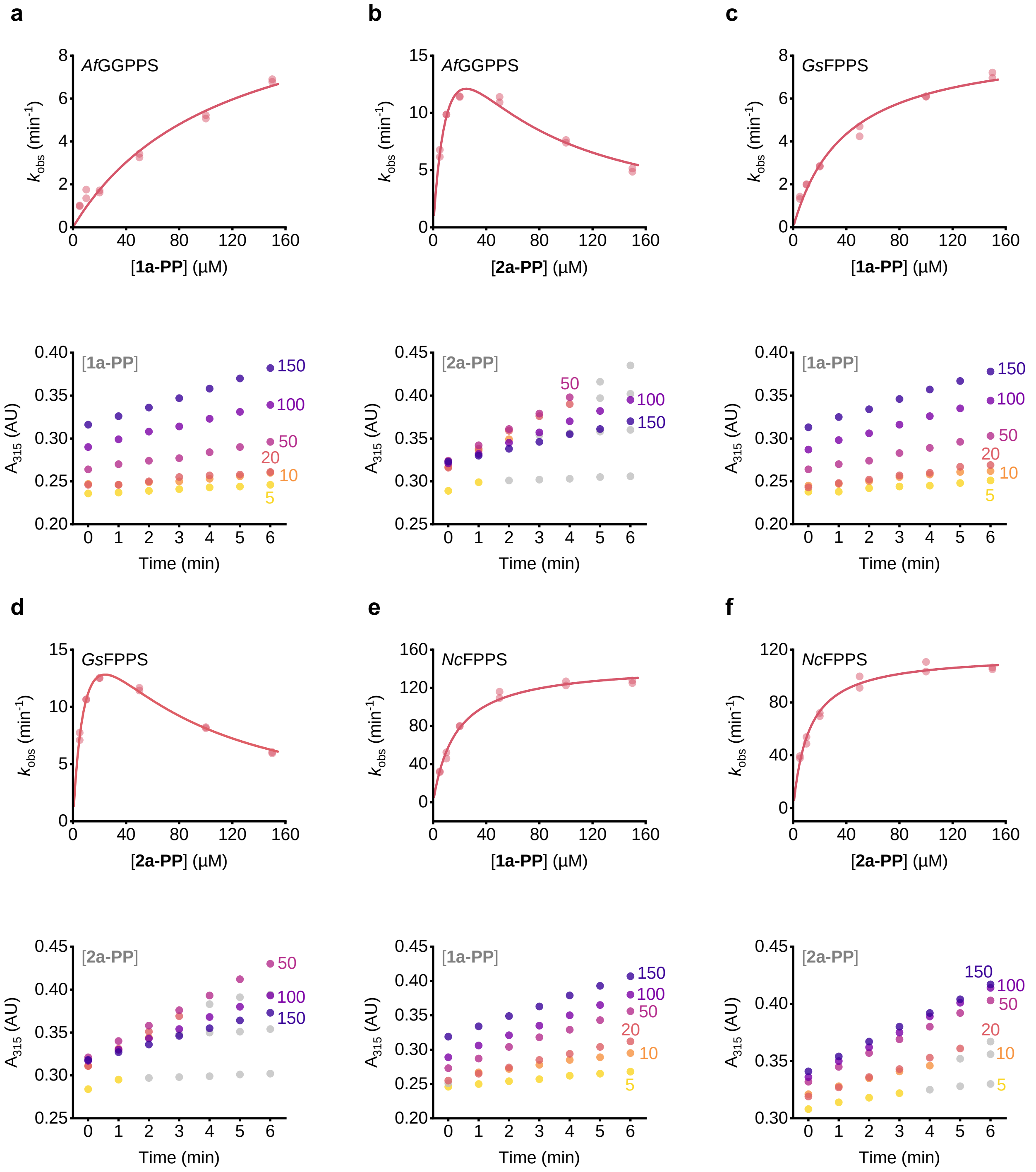

Figure S3. Representative Michaelis-Menten plots and kinetic traces.

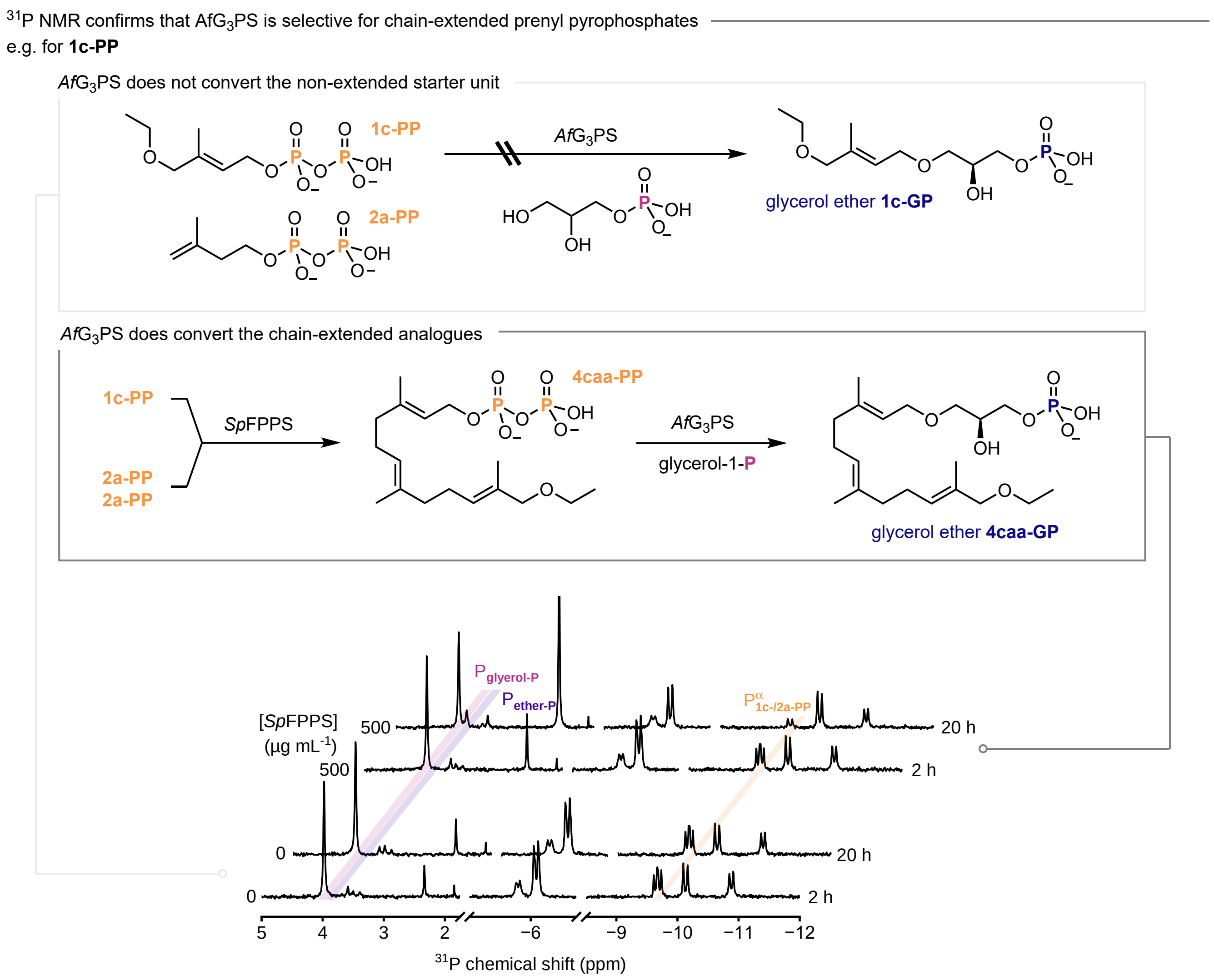

Figure S4. Exemplary scheme and ^{31}P NMR data showing that *Af*G$_3$PS is selective for chain-extended prenyl pyrophosphates.

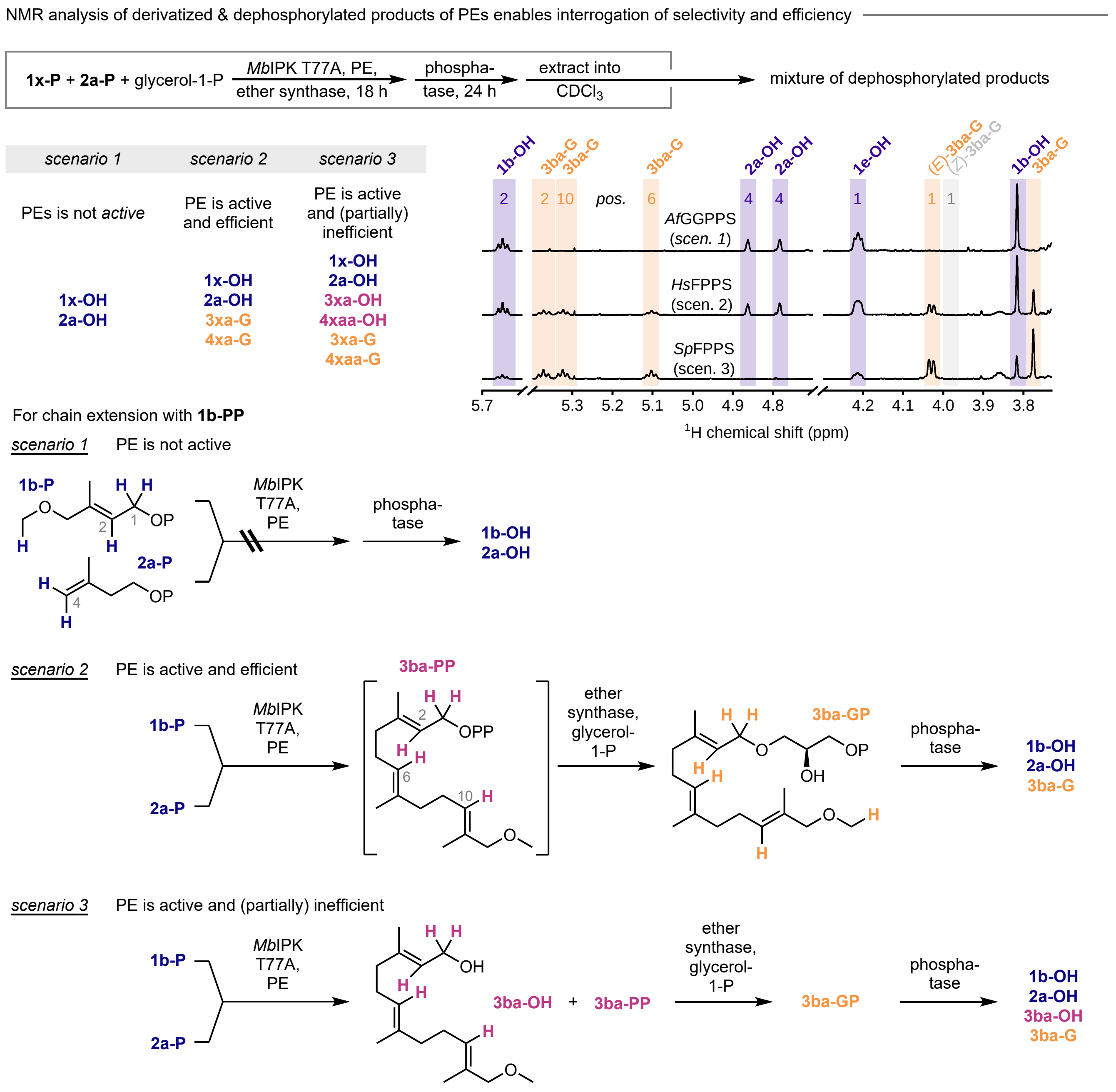

Figure S5. Schematic overview of ^{1}H NMR-based interrogation of the selectivity and efficiency of PEs. Analogous illustrative data (including a case of scenario 3) are illustrated in Fig. 4c in the main text.

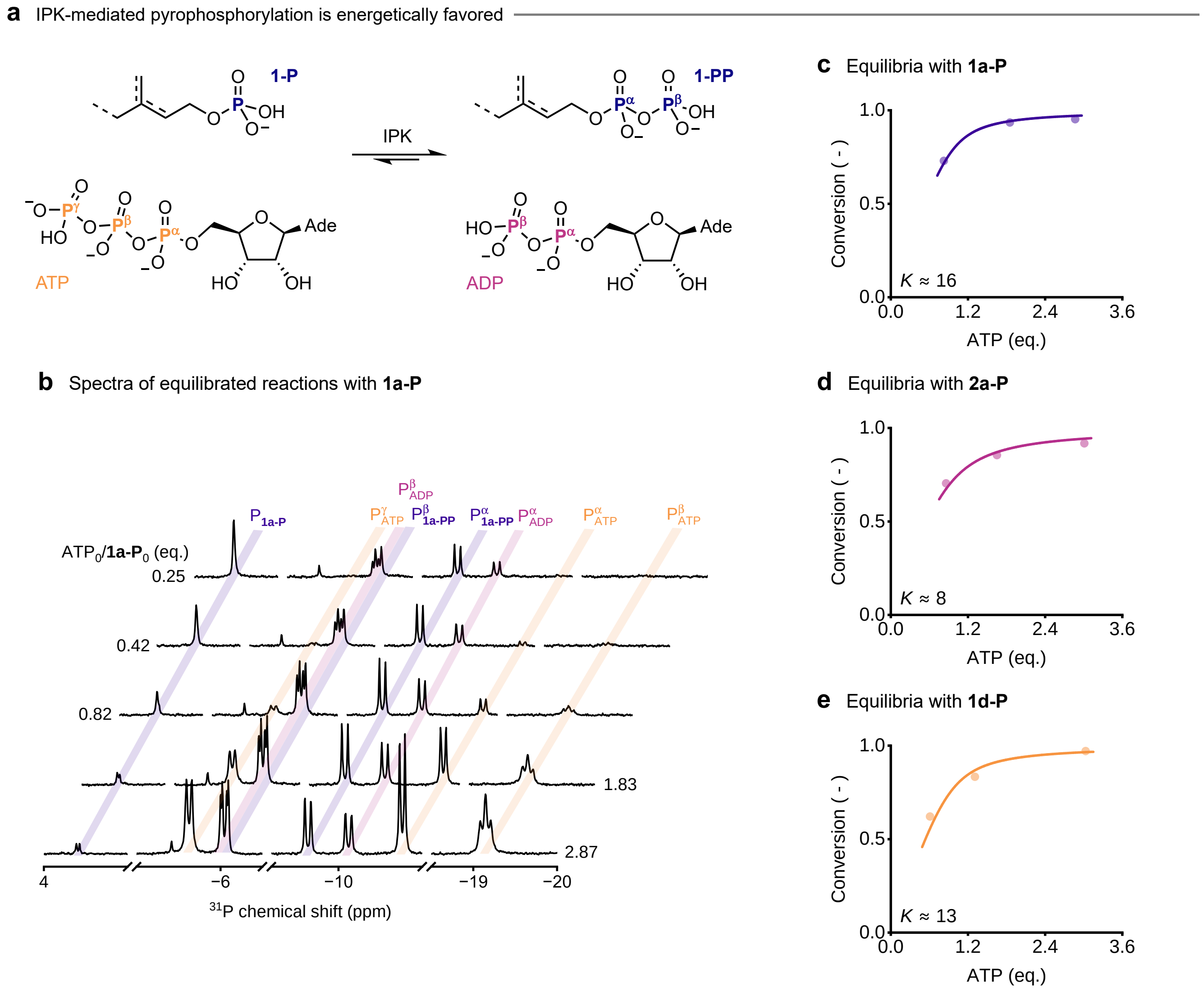

Figure S6. Equilibrium of IPK-mediated prenyl mono-to-pyrophosphorylation.

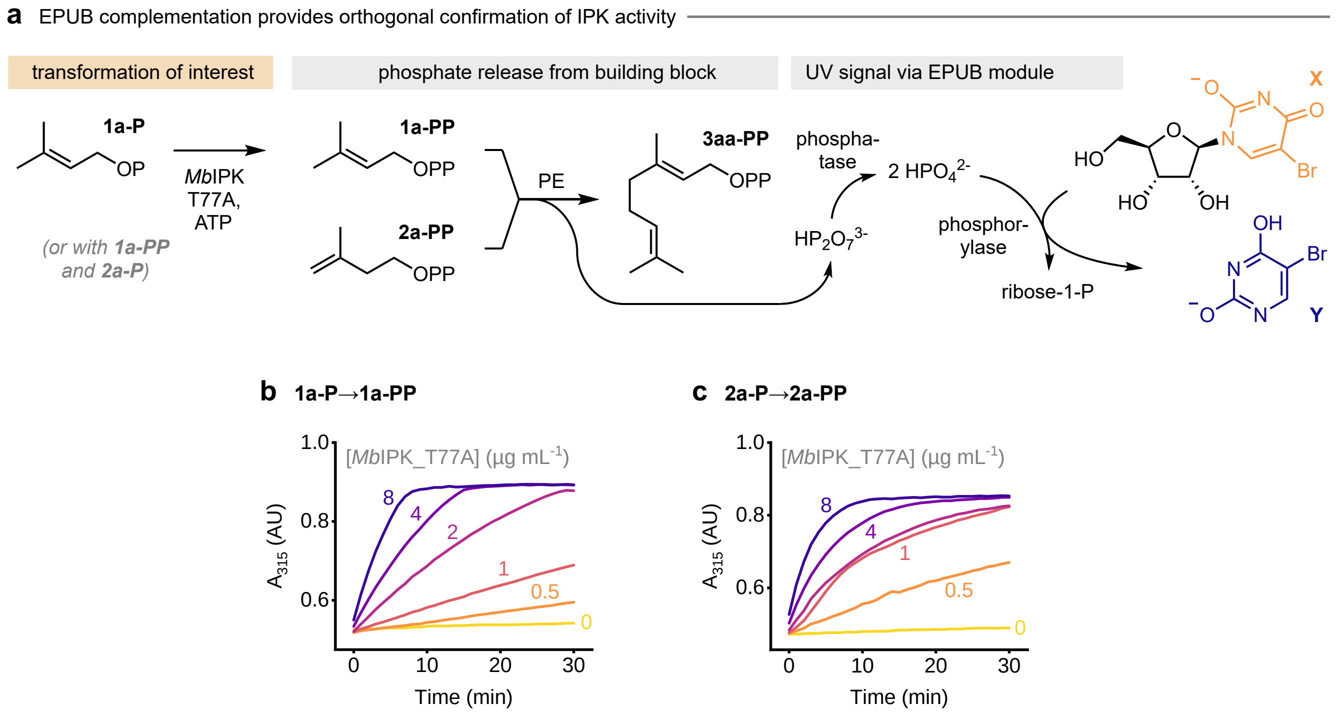

Figure S7. Orthogonal confirmation of *Mb*IPK T77A activity with a coupled EPUB assay.

Author contributions (with the CRediT definitions as recommended by Brand *et al.*[1])
Conceptualization, F.K.; Data curation, H.L., A.S., and F.K.; Formal analysis, H.L., A.S., and F.K.; Funding acquisition, C.M. and F.K.; Investigation, H.L., A.S., and F.K.; Methodology, F.K.; Project administration, C.M. and F.K.; Resources, C.M.; Software, - ; Supervision, C.M. and F.K.; Validation, - ; Visualization, F.K.; Writing—original draft, F.K.; Writing—review & editing, all authors.

Specifically regarding the experiments, H.L. cloned the genes for *Af*G$_3$PS, *Mb*IPK T77A, *Gs*PK and *Gs*LDH, established the parallelized production of PEs in multi-well plates, performed the mutagenesis of *Sp*FPPS and produced, purified, and characterized the *Sp*FPPS variants with EPUB. A.S. performed the mutagenesis of *Gs*FPPS and produced and purified the *Gs*FPPS variants and carried out preliminary characterization of the *Gs*FPPS variants. F.K. synthesized and characterized the compounds reported herein, cloned, produced, purified and characterized *Gt*PyNP, *Gt*IPP, *Ec*AP, the wt PEs, optimized the ARK module to assay *Mb*IPK T77A, produced, purified and characterized *Af*G$_3$PS, *Mb*IPK T77A, *Gs*PK and *Gs*LDH, developed the IPK-catalyzed *in situ* phosphorylation coupled to EPUB, performed the ^{31}P NMR experiments with *Mb*IPK T77A and/or *Af*G$_3$PS, performed the semi-preparative biotransformation sequences to confirm PE activity and selectivity by NMR, and characterized the *Gs*FPPS variants.

Data availability
All data depicted visually in the items in the main text (Figures 1−6) as well as in the Supplementary Information (Figures S1−S17, see below) are available as tabulated data from the externally hosted Supplementary Information at zenodo.org.[2] Similarly, all raw data, including sequencing data, UV traces and NMR spectra are available from the same zenodo entry. All enzymological data are reported following the STRENDA guidelines.[3,4]

Accessibility statement
The data presented in this manuscript are depicted using scientific color maps. Since ca. 4% of the human population are color vision-deficient, we made a conscious effort to avoid unscientific uses of color, such as color ambiguities and other biases that could lead to misrepresentation, limited accessibility, or loss of information upon reduction of the color space.[5,6] Thus, all figures herein were created using the scientific color map *plasma*, which retains color-coded information for all readers.

Safety and sustainability considerations
Many of the low molecular weight (iso-)prenoids prepared herein are volatile. In addition, THP-protected (iso-)prenols have intense and persistent olfactory properties. We recommend careful handling of these compounds.

In an effort to reduce the use of solvents which are toxic and/or harmful to the environment,[7] we made dedicated efforts to opt for renewable and non-toxic solvents (e.g. by using EtOAc instead of DCM for extractions). However, in some instances, the type of transformation and/or the substrate necessitated the use of potentially harmful solvents. For instance, DCM proved a highly effective solvent for many of the transformations reported herein and the use Et_2O as an extraction solvent during the workup of THP-protections enabled a telescoped reaction sequence. In those cases, we performed reactions at high concentrations to minimize the amount of harmful solvent used.

Biochemistry
General remarks (biochemistry)
All chemicals used in this study were of analytical grade or higher and purchased from Sigma Aldrich (Steinheim, Germany), TCI (Eschborn, Germany) or VWR (Darmstadt, Germany) and used without prior purification. Water deionized with a Sartorius water purification system was used for the preparation of all enzymatic reactions as well as purification and storage buffers. Enzymatic reactions were generally prepared from stock solutions of substrate(s), salt(s), buffer(s) and enzyme(s) and started *via* the addition of enzyme or key reagent as indicated for the respective experiment. Master mixes for EPUB were prepared

as described below and stored in aliquots of 1.8 mL at −20 °C until use. All pH values were adjusted at room temperature (20 °C), using concentrated HCl or NaOH. UV absorption spectra and UV traces were recorded in UV-transparent 96 well plates (UV-Star®, Greiner Bio-One, Frickenhausen, Germany) with a Biotek Synergy H1 microplate reader (BMG Labtech, Ortenberg, Germany). Nuclear magnetic resonance (NMR) spectra of enzymatic reactions were recorded on a Bruker AVIII 600 with the deuterated solvent acting as an internal deuterium lock. ^{31}P NMR chemical shifts are stated relative to phosphoric acid in water. Data are reported as follows: chemical shift (ppm), multiplicity (s = singlet, d = doublet, t = triplet, q = quartet, m = multiplet), coupling constant(s) (Hz), and integration. Data handling and routine calculations were carried out in Excel or LibreOffice, NMR data analysis in MestreNova (version 14.3), and analysis, fitting and data plotting in OriginPro (2021 or 2022b version).

Cloning

Expression vectors for all enzymes herein were cloned by Golden Gate Assembly from codon-optimized gene fragments with flanking BsaI sites (obtained from Genscript, Piscataway, USA) and a pACYC vector with BsaI sites (pACYC_GG).[8] Codon-optimization was performed with VectorBuilder (https://en.vectorbuilder.com/tool/codon-optimization.html). The sequences of all fragments and the original pACYC plasmid are listed below and are provided in the externally hosted supplementary information.[2] Golden Gate reactions contained 80 ng pACYC_GG, 100 ng insert with BsaI sites, 1 µL BsaI-HFv2, 0.5 µL Hi-T4 DNA ligase in T4 ligase buffer (all enzymes and buffers from New England Biolabs, Frankfurt, Germany) in a total volume of 20 µL and were incubated for 30 cycles of 5 min at 37 °C and 10 min at 16 °C, followed by final digestion step at 55 °C for 20 min and an inactivation step at 65 °C for 20 min, followed by storage at 12 °C. The resulting plasmids were transformed into *Escherichia coli* DH5α or NEB10β cell through a heat shock protocol. To this end, 2 µL of the Golden Gate mixture were placed in a sterile 1.5 mL plastic tube and 50 µL chemically competent cells (prepared as described by Inoue *et al.*[9]) of NEB10β were added. This mixture was incubated on ice for 30 min. Subsequently, the mixture was heated at 42 °C for 40 s and immediately afterwards cooled at 4 °C for 2 min. Next, 950 µL SOC medium (0.186 g L^{-1} KCl, 0.5 g L^{-1} NaCl, 5 g L^{-1} yeast extract, 20 g L^{-1} tryptone, 10 mM $MgCl_2$, 10 mM $MgSO_4$ and 20 mM glucose) were added and the resulting culture was incubated at 37 °C and 200 rpm for 1 h. Afterwards, the cells were plated on LB agar plates (10 g L^{-1} tryptone, 5 g L^{-1} yeast extract, 10 g L^{-1} NaCl and 15 g L^{-1} agar) supplemented with 34 mg L^{-1} chloramphenicol and grown overnight at 37 °C. Individual clones were then used to inoculate 5 mL liquid cultures in LB medium containing 34 mg L^{-1} chloramphenicol which were incubated overnight at 37 °C and 200 rpm. On the following day, the plasmid DNA was isolated from these cultures using the QIAprep Spin Miniprep Kits (Hilden, Germany) according to the manufacturers instruction, employing a final elution with 30 µL ddH_2O. The DNA concentration in the eluate was determined with a NanoDrop 2000 spectrophotometer (Thermo Scientific) and the isolated plasmids were stored at −20 °C. Sanger sequencing was performed by Eurofins Genomics, using 50−100 ng of isolated plasmid DNA. Plasmid DNA exhibiting the correct gene was then cloned into *E. coli* BL21(DE3) (using the same heat shock protocol) for protein expression.

Cryostocks of all cultures were prepared by freezing in aqueous glycerol. To this end, a 500 µL aliquot of an overnight culture of the respective strain in LB medium was mixed with 500 µL sterile 50% (v/v) glycerol, mixed by pipetting, and frozen at −70 °C.

pACYC_GG

AAAACCTAGGCTGCTGCCACCGCTGAGCAATAACTAGCATAACCCCTTGGGGCCTCTAAACGGGTCTTGAGGGGTTTTTT
GCTGAAACCTCAGGCATTTGAGAAGCACACGGTCACACTGCTTCCGGTAGTCAATAAACCGGTAAACCAGCAATAGACAT
AAGCGGCTATTTAACGACCCTGCCCTGAACCGACGACCGGGTCGAATTTGCTTTCGAATTTCTGCCATTCATCCGCTTATT
ATCACTTATTCAGGCGTAGCACCAGGCGTTTAAGGGCACCAATAACTGCCTTAAAAAAATTACGCCCCGCCCTGCCACTCA
TCGCAGTACTGTTGTAATTCATTAAGCATTCTGCCGACATGGAAGCCATCACAGACGGCATGATGAACCTGAATCGCCAG
CGGCATCAGCACCTTGTCGCCTTGCGTATAATATTTGCCCATAGTGAAAACGGGGGCGAAGAAGTTGTCCATATTGGCCA
CGTTTAAATCAAAACTGGTGAAACTCACCCAGGGATTGGCTGAAACGAAAAACATATTCTCAATAAACCCTTTAGGGAAATA
GGCCAGGTTTTCACCGTAACACGCCACATCTTGCGAATATATGTGTAGAAACTGCCGGAAATCGTCGTGGTATTCACTCCA
GAGCGATGAAAACGTTTCAGTTTGCTCATGGAAAACGGTGTAACAAGGGTGAACACTATCCCATATCACCAGCTCACCGT
CTTTCATTGCCATACGGAACTCCGGATGAGCATTCATCAGGCGGGCAAGAATGTGAATAAAGGCCGGATAAAACTTGTGC
TTATTTTTCTTTACGGTCTTTAAAAAGGCCGTAATATCCAGCTGAACGGTCTGGTTATAGGTACATTGAGCAACTGACTGAA
ATGCCTCAAAATGTTCTTTACGATGCCATTGGGATATATCAACGGTGGTATATCCAGTGATTTTTTTCTCCATTTTAGCTTCC
TTAGCTCCTGAAAATCTCGATAACTCAAAAAATACGCCCGGTAGTGATCTTATTTCATTATGGTGAAAGTTGGAACCTCTTA
CGTGCCGATCAACCTCTCATTTTCGCCAAAAGTTGGCCCAGGGCTTCCCGGTATCAACAGGGACACCAGGATTTATTTATT
CTGCGAAGTGATCTTCCGTCACAGGTATTTATTCGGCGCAAAGTGCGTCGGGTGATGCTGCCAACTTACTGATTTAGTGTA
TGATGGTGTTTTTGAGGTGCTCCAGTGGCTTCTGTTTCTATCAGCTGTCCCTCCTGTTCAGCTACTGACGGGGTGGTGCG
TAACGGCAAAAGCACCGCCGGACATCAGCGCTAGCGGAGTGTATACTGGCTTACTATGTTGGCACTGATGAGGGTGTCA
GTGAAGTGCTTCATGTGGCAGGAGAAAAAAGGCTGCACCGGTGCGTCAGCAGAATATGTGATACAGGATATATTCCGCTT
CCTCGCTCACTGACTCGCTACGCTCGGTCGTTCGACTGCGGCGAGCGGAAATGGCTTACGAACGGGGCGGAGATTTCCT
GGAAGATGCCAGGAAGATACTTAACAGGGAAGTGAGAGGGCCGCGGCAAAGCCGTTTTTCCATAGGCTCCGCCCCCCTG
ACAAGCATCACGAAATCTGACGCTCAAATCAGTGGTGGCGAAACCCGACAGGACTATAAAGATACCAGGCGTTTCCCCTG
GCGGCTCCCTCGTGCGCTCTCCTGTTCCTGCCTTTCGGTTTACCGGTGTCATTCCGCTGTTATGGCCGCGTTTGTCTCATT
CCACGCCTGACACTCAGTTCCGGGTAGGCAGTTCGCTCCAAGCTGGACTGTATGCACGAACCCCCCGTTCAGTCCGACC
GCTGCGCCTTATCCGGTAACTATCGTCTTGAGTCCAACCCGGAAAGACATGCAAAAGCACCACTGGCAGCAGCCACTGGT
AATTGATTTAGAGGAGTTAGTCTTGAAGTCATGCGCCGGTTAAGGCTAAACTGAAAGGACAAGTTTTGGTGACTGCGCTCC
TCCAAGCCAGTTACCTCGGTTCAAAGAGTTGGTAGCTCAGAGAACCTTCGAAAAACCGCCCTGCAAGGCGGTTTTTTCGT
TTTCAGAGCAAGAGATTACGCGCAGACCAAAACGATCTCAAGAAGATCATCTTATTAATCAGATAAAATATTTCTAGATTTC
AGTGCAATTTATCTCTTCAAATGTAGCACCTGAAGTCAGCCCCATACGATATAAGTTGTAATTCTCATGTTAGTCATGCCCC
GCGCCCACCGGAAGGAGCTGACTGGGTTGAAGGCTCTCAAGGGCATCGGTCGAGATCCCGGTGCCTAATGAGTGAGCT
AACTTACATTAATTGCGTTGCGCTCACTGCCCGCTTTCCAGTCGGGAAACCTGTCGTGCCAGCTGCATTAATGAATCGGC
CAACGCGCGGGGAGAGGCGGTTTGCGTATTGGGCGCCAGGGTGGTTTTTCTTTTCACCAGTGAAACGGGCAACAGCTGA
TTGCCCTTCACCGCCTGGCCCTGAGAGAGTTGCAGCAAGCGGTCCACGCTGGTTTGCCCCAGCAGGCGAAAATCCTGTT
TGATGGTGGTTAACGGCGGGATATAACATGAGCTGTCTTCGGTATCGTCGTATCCCACTACCGAGATGTCCGCACCAACG
CGCAGCCCGGACTCGGTAATGGCGCGCATTGCGCCCAGCGCCATCTGATCGTTGGCAACCAGCATCGCAGTGGGAACG
ATGCCCTCATTCAGCATTTGCATGGTTTGTTGAAAACCGGACATGGCACTCCAGTCGCCTTCCCGTTCCGCTATCGGCTG
AATTTGATTGCGAGTGAGATATTTATGCCAGCCAGCCAGACGCAGACGCGCCGAGACAGAACTTAATGGGCCCGCTAACA
GCGCGATTTGCTGGTGACCCAATGCGACCAGATGCTCCACGCCCAGTCGCGTACCGTCTTCATGGGAGAAAATAATACTG
TTGATGGGTGTCTGGTCAGAGACATCAAGAAATAACGCCGGAACATTAGTGCAGGCAGCTTCCACAGCAATGGCATCCTG
GTCATCCAGCGGATAGTTAATGATCAGCCCACTGACGCGTTGCGCGAGAAGATTGTGCACCGCCGCTTTACAGGCTTCGA
CGCCGCTTCGTTCTACCATCGACACCACCACGCTGGCACCCAGTTGATCGGCGCGAGATTTAATCGCCGCGACAATTTGC
GACGGCGCGTGCAGGGCCAGACTGGAGGTGGCAACGCCAATCAGCAACGACTGTTTGCCCGCCAGTTGTTGTGCCACG
CGGTTGGGAATGTAATTCAGCTCCGCCATCGCCGCTTCCACTTTTTCCCGCGTTTTCGCAGAAACGTGGCTGGCCTGGTT
CACCACGCGGGAAACGGTCTGATAAGAGACACCGGCATACTCTGCGACATCGTATAACGTTACTGGTTTCACATTCACCA
CCCTGAATTGACTCTCTTCCGGGCGCTATCATGCCATACCGCGAAAGGTTTTGCGCCATTCGATGGTGTCCGGGATCTCG
ACGCTCTCCCTTATGCGACTCCTGCATTAGGAAATTAATACGACTCACTATAGGGGAATTGTGAGCGGATAACAATTCCCC

TGTAGAAATAATTTTGTTTAACTTTAATAAGGAGATATACCATGGGCAGCAGCCATCATCATCATCATCACGGCAGCGGCC
TGGTGCCGCGCGGCAGCGCTGGTAGAGACCGGGCCTGAAGGTCTCGCTTGGGCCCGAACAAAAACTCATCTCACGAAC
AGAAAGTAATCGTATTGTACACGGCCGCATAATCGAAATTAATACGACTCACTATAGGGGAATTGTGAGCGGATAACAATT
CCCCATCTTAGTATATTAGTTAAGTATAAGAAGGAGATATACATATGTTCGGCGAGACGGAAAGTGAAACGTGATTTCATG
CGTCATTTTGAACATTTTGTAAATCTTATTTAATAATGTGTGCGGCAATTCATGCGTTTATACGTCTCTGACCGGAAAGAAA
CCGCTGCTGCGAAATTTGAACGCCAGCACATGGACTCGTCTACTAGCGCAGCTTAATTAACCTAGGAGAAAT

Enzymes

Note: Starting from their UniProt entries, three eukaryotic enzymes (AtFPPS, HsFPPS, and SfFPPS) were truncated to remove the disordered (N-terminal) leader peptide to have the protein begin at the first α-helix. AlphaFold2 (AF2) models of each full-length and truncated protein indicated that the leader peptide would likely not contribute to the structure of the core protein. The PDB entry for TcFPPS already lacked the leader peptide and, thus, we used this sequence instead of the NCBI entry.

***Archaeoglobus flugidus* geranylgeranyl pyrophosphate synthase** (*Af*GGPPS)

PDB - , UniProt Q9V305_ARCFL, accession WP_048064551.1

MLKEEIAKRAEIINKAIEELLPEREPIGLYKAARHLIKAGGKRLRPVISLLAVEALGKDYRKIIPAAVSIETIHNFTLVHDDIMDRDEMR
RGVPTVHRVYGEATAILAGDTLFAEAFKLLTKCDVESEGIRKATEMLSDVCIKICEGQYYDMSFEKKESVSEEEYLRMVELKTGV
LIAASAALPAVLFGESEEIVKALWDYGVLSGIGFQIQDDLLDLTEETGKDWGSDLLKGKKTLIVIKAFEKGVKLKTFGKEKADVSEI
RDDIEKLRECGAIDYAASMARKMAEEAKRKLEVLPESKAKETLLELTDFLVTRKK

Codon-optimized sequence (Start and stop codon, cut away by BsaI, appended sequence)

ctaaccggtctcctggtATGCTGAAAGAAGAAATTGCCAAACGCGCCGAAATTATCAACAAAGCGATCGAAGAACTGCTGCCGGAA
CGTGAACCGATCGGCCTGTATAAAGCGGCGCGTCATCTGATTAAAGCGGGCGGCAAACGCCTGCGCCCGGTTATTAGCC
TGCTGGCGGTGGAAGCGCTGGGTAAAGATTACCGCAAAATTATTCCGGCCGCGGTGAGCATTGAAACCATTCATAATTTT
ACCCTGGTGCATGATGATATTATGGACCGTGATGAAATGCGCCGTGGCGTTCCGACCGTGCATCGCGTGTATGGCGAAG
CCACTGCGATTCTGGCGGGCGACACCCTGTTTGCGGAAGCCTTTAAACTGCTGACCAAATGCGACGTCGAAAGCGAAGG
CATTCGCAAAGCGACCGAAATGCTGAGCGATGTGTGCATTAAAATTTGTGAAGGCCAGTATTACGATATGAGCTTTGAAAA
GAAAGAAAGCGTGAGCGAAGAAGAATACCTGCGCATGGTGGAACTGAAAACCGGCGTGCTGATTGCCGCGAGCGCGGC
GCTGCCGGCGGTACTGTTTGGCGAAAGCGAAGAAATCGTGAAAGCGCTGTGGGACTATGGCGTGCTGAGCGGCATTGG
CTTTCAGATTCAGGACGATCTGCTGGATCTGACCGAAGAAACCGGCAAAGATTGGGGCAGCGATCTGCTGAAAGGTAAAA
AAACCCTGATTGTGATTAAAGCGTTCGAAAAAGGCGTAAAACTGAAAACCTTTGGCAAAGAAAAAGCGGATGTGAGCGAAA
TTCGTGATGATATTGAAAAACTGCGTGAATGCGGCGCGATTGATTACGCCGCGTCGATGGCGCGCAAAATGGCGGAAGA
AGCGAAACGTAAACTGGAAGTTCTGCCGGAAAGTAAAGCAAAAGAAACCCTGCTGGAACTGACCGATTTTCTGGTGACCC
GTAAAAAATAActtgggagaccaacta

Resulting tagged protein (His$_6$-tag, linker with thrombin cut site), 338 AA, 37.6 kDa, ε_{280} = 22.9 mM^{-1} cm^{-1}

MGSSHHHHHHGSGLVPRGSAGMLKEEIAKRAEIINKAIEELLPEREPIGLYKAARHLIKAGGKRLRPVISLLAVEALGKDYRKIIPA
AVSIETIHNFTLVHDDIMDRDEMRRGVPTVHRVYGEATAILAGDTLFAEAFKLLTKCDVESEGIRKATEMLSDVCIKICEGQYYDM
SFEKKESVSEEEYLRMVELKTGVLIAASAALPAVLFGESEEIVKALWDYGVLSGIGFQIQDDLLDLTEETGKDWGSDLLKGKKTLI
VIKAFEKGVKLKTFGKEKADVSEIRDDIEKLRECGAIDYAASMARKMAEEAKRKLEVLPESKAKETLLELTDFLVTRKK*

***Arabidopsis thaliana* farnesyl pyrophosphate synthase** (*At*FPPS)

PDB - , UniProt FPPS1_ARATH, accession NP_199588.1 (~~leader peptide~~, added **M**)

~~MSVSCCCRNLGKTIKKAIPSHHLHLRSLGGSLYRRRIQSSS~~**M**ETDLKSTFLNVYSVLKSDLLHDPSFEFTNESRLWVDRMLDYN
VRGGKLNRGLSVVDSFKLLKQGNDLTEQEVFLSCALGWCIEWLQAYFLVLDDIMDNSVTRRGQPCWFRVPQVGMVAINDGILL
RNHIHRILKKHFRDKPYYVDLVDLFNEVELQTACGQMIDLITTFEGEKDLAKYSLSIHRRIVQYKTAYYSFYLPVACALLMAGENL
ENHIDVKNVLVDMGIYFQVQDDYLDCFADPETLGKIGTDIEDFKCSWLVVKALERCSEEQTKILYENYGKPDPSNVAKVKDLYKE
LDLEGVFMEYESKSYEKLTGAIEGHQSKAIQAVLKSFLAKIYKRQK

Codon-optimized sequence (Start and stop codon, cut away by BsaI, appended sequence)

ctaaccggtctcctggtATGGAAACCGATCTGAAAAGCACCTTTCTGAACGTGTATAGCGTTCTGAAAAGCGATCTGCTGCATGATC
CGAGCTTTGAATTTACCAACGAATCCCGCCTGTGGGTGGATCGCATGCTGGATTATAACGTTCGCGGTGGCAAACTGAAT
CGTGGCCTGAGCGTGGTGGATAGCTTTAAACTGCTGAAACAGGGTAATGATTTGACCGAACAAGAAGTCTTTCTGAGCTG
CGCCCTGGGCTGGTGCATTGAATGGCTGCAGGCCTATTTTCTGGTTCTGGATGATATTATGGATAACAGCGTGACCCGTC
GCGGCCAGCCGTGTTGGTTTCGCGTGCCGCAGGTGGGCATGGTTGCGATTAACGATGGCATTCTGCTGCGTAACCATAT
TCATCGTATTCTGAAAAAACATTTTCGCGATAAACCGTATTATGTGGATCTGGTGGATCTGTTTAACGAAGTAGAACTGCAG
ACCGCGTGCGGCCAGATGATTGACCTGATTACCACCTTTGAAGGCGAAAAAGATCTGGCCAAATACTCGCTGAGCATTCA
TCGCCGCATTGTGCAGTACAAAACCGCGTATTACAGCTTTTACCTGCCGGTGGCGTGCGCGCTGCTGATGGCGGGTGAA
AACCTGGAAAATCACATTGATGTCAAAAACGTGCTGGTGGATATGGGCATTTACTTCCAGGTGCAGGATGATTATCTGGAT
TGCTTTGCGGATCCGGAAACCCTGGGCAAAATTGGTACCGATATTGAAGACTTTAAATGCTCGTGGCTGGTGGTGAAAGC
GCTGGAACGTTGCAGCGAAGAACAGACCAAAATTCTGTACGAAAACTATGGTAAACCGGATCCGAGCAACGTGGCGAAAG
TTAAAGACCTGTACAAAGAACTGGATCTGGAAGGCGTGTTTATGGAATATGAAAGCAAAAGCTATGAAAAACTGACCGGTG
CGATTGAAGGCCATCAGAGCAAAGCCATTCAGGCCGTACTGAAAAGCTTTCTGGCGAAAATCTATAAACGCCAGAAATAAc
ttgggagaccaacta

Resulting tagged protein (His$_6$-tag, linker with thrombin cut site), 364 AA, 41.8 kDa, ε_{280} = 54.3 mM^{-1} cm^{-1}

MGSSHHHHHHGSGLVPRGSAGMETDLKSTFLNVYSVLKSDLLHDPSFEFTNESRLWVDRMLDYNVRGGKLNRGLSVVDSFKL
LKQGNDLTEQEVFLSCALGWCIEWLQAYFLVLDDIMDNSVTRRGQPCWFRVPQVGMVAINDGILLRNHIHRILKKHFRDKPYYV
DLVDLFNEVELQTACGQMIDLITTFEGEKDLAKYSLSIHRRIVQYKTAYYSFYLPVACALLMAGENLENHIDVKNVLVDMGIYFQV
QDDYLDCFADPETLGKIGTDIEDFKCSWLVVKALERCSEEQTKILYENYGKPDPSNVAKVKDLYKELDLEGVFMEYESKSYEKL
TGAIEGHQSKAIQAVLKSFLAKIYKRQK*

***Escherichia coli* farnesyl pyrophosphate synthase** (*Ec*FPPS)

PDB 1rqi, UniProt ISPA_ECOLI, accession number WP_137556520.1

MDFPQQLEACVKQANQALSRFIAPLPFQNTPVVETMQYGALLGGKRLRPFLVYATGHMFGVSTNTLDAPAAAVECIHAYSLIHD
DLPAMDDDDLRRGLPTCHVKFGEANAILAGDALQTLAFSILTDADMPEVSDRDRISMISELASASGIAGMCGGQALDLDAEGKH
VPLDALERIHRHKTGALIRAAVRLGALSAGDKGRRALPVLDKYAESIGLAFQVQDDILDVVGDTATLGKRQGADQQLGKSTYPAL
LGLEQARKKARDLIDDARQSLKQLAEQSLDTSALEALADYIIQRNK

Codon-optimized sequence (Start and stop codon, cut away by BsaI, appended sequence)

ctaaccggtctcctggtATGGATTTTCCGCAGCAGCTGGAAGCGTGCGTGAAACAGGCCAACCAGGCGCTGAGCCGCTTTATCGC
GCCGCTGCCGTTTCAGAACACCCCGGTTGTGGAAACCATGCAGTATGGTGCGCTGCTGGGCGGCAAACGCCTGCGCCC
GTTCCTGGTGTATGCCACCGGCCACATGTTTGGCGTTTCAACCAACACCCTGGATGCGCCGGCGGCGGCCGTCGAATGC
ATTCATGCGTATTCTCTGATTCATGATGATCTGCCGGCAATGGATGATGATGATCTGCGCCGCGGTCTGCCGACCTGTCA
CGTGAAATTCGGTGAAGCCAACGCCATTCTGGCCGGCGATGCGCTGCAGACCCTGGCCTTTAGCATTCTGACAGATGCC
GATATGCCGGAAGTGAGCGATCGCGACCGCATTAGCATGATTAGCGAACTGGCGAGCGCCAGCGGCATTGCGGGTATGT
GCGGCGGTCAGGCGCTGGATCTGGATGCCGAAGGCAAACATGTTCCGCTGGATGCGCTGGAACGCATTCATCGTCATAA
AACCGGCGCGCTGATTCGCGCCGCCGTTCGCCTGGGCGCGCTGAGCGCGGGCGATAAAGGTCGTCGCGCCCTGCCGG
TGCTGGACAAATATGCTGAAAGCATTGGCCTGGCCTTTCAGGTGCAGGATGATATTCTGGATGTTGTGGGCGATACCGCG
ACGCTGGGCAAACGCCAGGGCGCGGATCAGCAGCTGGGTAAAAGCACGTATCCGGCGCTGCTGGGCCTGGAACAGGCC
CGTAAAAAAGCGCGCGATCTGATTGATGATGCCCGCCAGAGCCTGAAACAGCTGGCCGAACAGAGCCTGGATACGAGCG
CGCTGGAAGCGCTGGCGGATTATATTATTCAGCGCAACAAATAActtgggagaccaacta

Resulting tagged protein (His$_6$-tag, linker with thrombin cut site), 320 AA, 34.3 kDa, ε_{280} = 8.9 mM^{-1} cm^{-1}

MGSSHHHHHHGSGLVPRGSAGMDFPQQLEACVKQANQALSRFIAPLPFQNTPVVETMQYGALLGGKRLRPFLVYATGHMFG
VSTNTLDAPAAAVECIHAYSLIHDDLPAMDDDDLRRGLPTCHVKFGEANAILAGDALQTLAFSILTDADMPEVSDRDRISMISELA
SASGIAGMCGGQALDLDAEGKHVPLDALERIHRHKTGALIRAAVRLGALSAGDKGRRALPVLDKYAESIGLAFQVQDDILDVVG
DTATLGKRQGADQQLGKSTYPALLGLEQARKKARDLIDDARQSLKQLAEQSLDTSALEALADYIIQRNK*

Geobacillus stearothermophilus **farnesyl pyrophosphate synthase** (*Gs*FPPS)

PDB 5ayp, UniProt ISPA_GEOSE, accession WP_033016440.1

MAQLSVEQFLNEQKQAVETALSRYIERLEGPAKLKKAMAYSLEAGGKRIRPLLLLSTVRALGKDPAVGLPVACAIEMIHTYSLIHD
DLPSMDNDDLRRGKPTNHKVFGEAMAILAGDGLLTYAFQLITEIDDERIPPSVRLRLIERLAKAAGPEGMVAGQAADMEGEGKT
LTLSELEYIHRHKTGKMLQYSVHAGALIGGADARQTRELDEFAAHLGLAFQIRDDILDIEGAEEKIGKPVGSDQSNNKATYPALLS
LAGAKEKLAFHIEAAQRHLRNADVDGAALAYICELVAARDH

Codon-optimized sequence (Start and stop codon, cut away by BsaI, appended sequence)

ctaaccggtctcctggtATGGCCCAGCTGAGCGTTGAACAGTTTCTGAACGAACAGAAACAGGCGGTGGAAACCGCGCTGAGCCG
TTATATTGAACGCCTGGAAGGCCCGGCCAAACTGAAAAAAGCCATGGCGTATAGCCTGGAAGCGGGCGGCAAACGCATC
CGTCCGCTGCTGCTGCTGAGCACCGTGCGCGCCCTGGGTAAAGATCCGGCGGTGGGCCTGCCGGTGGCATGTGCCATT
GAAATGATTCATACCTATTCGCTGATTCATGATGATCTGCCGAGCATGGATAACGATGACCTGCGTCGTGGCAAACCGAC
CAATCATAAAGTGTTTGGCGAAGCCATGGCGATTCTGGCGGGCGATGGCCTGCTGACCTACGCGTTTCAGCTGATTACCG
AAATTGATGATGAACGCATTCCGCCGAGCGTGCGTCTGCGCCTGATTGAACGTCTGGCGAAGGCGGCCGGCCCGGAAG
GCATGGTGGCGGGTCAGGCGGCGGATATGGAAGGCGAAGGCAAAACTCTGACCCTGAGCGAACTGGAATATATTCATCG
CCACAAAACCGGCAAAATGCTGCAGTATAGCGTGCATGCGGGTGCGCTGATTGGCGGTGCCGACGCGCGTCAGACCCG
TGAACTGGATGAATTCGCGGCGCATCTGGGCCTGGCGTTCCAGATTCGCGATGATATTCTGGATATTGAAGGCGCGGAA
GAAAAAATTGGCAAACCGGTCGGCAGCGACCAAAGCAACAATAAAGCGACCTACCCGGCCCTGCTGTCGCTGGCCGGCG
CGAAAGAAAAACTGGCGTTTCATATTGAAGCCGCGCAGCGTCATCTGCGCAATGCGGATGTGGATGGCGCGGCGCTGGC
ATATATTTGCGAACTGGTGGCGGCCCGCGATCACTAActtgggagaccaacta

Resulting tagged protein (His_6-tag, linker with thrombin cut site), 318 AA, 34.4 kDa, ε_{280} = 12.0 mM^{-1} cm^{-1}

MGSSHHHHHHGSGLVPRGSAGMAQLSVEQFLNEQKQAVETALSRYIERLEGPAKLKKAMAYSLEAGGKRIRPLLLLSTVRALG
KDPAVGLPVACAIEMIHTYSLIHDDLPSMDNDDLRRGKPTNHKVFGEAMAILAGDGLLTYAFQLITEIDDERIPPSVRLRLIERLAK
AAGPEGMVAGQAADMEGEGKTLTLSELEYIHRHKTGKMLQYSVHAGALIGGADARQTRELDEFAAHLGLAFQIRDDILDIEGAE
EKIGKPVGSDQSNNKATYPALLSLAGAKEKLAFHIEAAQRHLRNADVDGAALAYICELVAARDH*

***Homo sapiens* farnesyl pyrophosphate synthase** (*Hs*FPPS)

PDB 1yq7, UniProt FPPS_HUMAN, accession NP_001129293.1 (~~leader peptide~~, **added M**)

~~MPLSRWLRSVGVFLLPAPYWAPRERWLGSLRRPSLVHGYPVLAWHSARCWCQAWTEEPRALCSSLRMNGDQNSDV~~**M**YAQ
EKQDFVQHFSQIVRVLTEDEMGHPEIGDAIARLKEVLEYNAIGGKYNRGLTVVVAFRELVEPRKQDADSLQRAWTVGWCVELL
QAFFLVADDIMDSSLTRRGQICWYQKPGVGLDAINDANLLEACIYRLLKLYCREQPYYLNLIELFLQSSYQTEIGQTLDLLTAPQG
NVDLVRFTEKRYKSIVKYKTAFYSFYLPIAAAMYMAGIDGEKEHANAKKILLEMGEFFQIQDDYLDLFGDPSVTGKIGTDIQDNKC
SWLVVQCLQRATPEQYQILKENYGQKEAEKVARVKALYEELDLPAVFLQYEEDSYSHIMALIEQYAAPLPPAVFLGLARKIYKRR
K

Codon-optimized sequence (Start and stop codon, cut away by BsaI, appended sequence)

ctaaccggtctcctggtATGTACGCGCAGGAAAAACAGGATTTTGTTCAGCACTTTAGCCAGATTGTGCGCGTGCTGACCGAAGAT
GAAATGGGCCATCCGGAAATTGGCGATGCCATCGCCCGTCTGAAAGAAGTGCTGGAATACAACGCGATTGGCGGCAAAT
ACAATCGCGGCCTGACCGTGGTTGTGGCGTTCCGCGAACTGGTGGAACCGCGTAAACAGGATGCGGATAGCCTGCAGC
GCGCCTGGACCGTGGGCTGGTGCGTGGAACTGCTGCAGGCGTTTTTCCTGGTGGCCGATGACATTATGGATAGCAGCCT
GACCCGCCGTGGCCAGATTTGCTGGTACCAGAAACCGGGCGTTGGCCTGGATGCGATTAATGATGCGAACCTGCTGGAA
GCGTGCATTTACCGTCTGCTGAAACTGTATTGCCGTGAGCAGCCGTATTATCTGAACCTGATTGAACTGTTTCTGCAGAGC
AGCTATCAGACCGAAATCGGCCAGACCCTGGATCTGCTGACGGCGCCGCAGGGCAACGTGGATCTGGTGCGCTTTACTG
AGAAACGTTATAAAAGCATTGTGAAATATAAAACCGCATTTTACAGCTTTTATCTGCCGATTGCGGCGGCCATGTATATGGC
GGGCATTGATGGCGAAAAAGAACACGCGAACGCGAAAAAGATTCTGCTGGAAATGGGCGAATTTTTTCAGATTCAGGATG
ATTATCTGGATCTGTTTGGCGATCCGTCCGTGACCGGCAAAATTGGCACCGACATTCAGGATAACAAATGCTCATGGCTG
GTGGTGCAGTGCCTGCAGCGCGCGACCCCGGAACAGTACCAGATTCTGAAAGAAAATTACGGCCAGAAAGAAGCGGAAA
AAGTGGCGCGCGTGAAAGCCCTGTACGAAGAACTGGATCTGCCAGCCGTATTTCTGCAGTACGAAGAAGATAGCTACAG
CCATATTATGGCCCTGATTGAACAGTACGCCGCGCCGCTGCCGCCGGCGGTGTTCCTGGGCCTGGCGCGTAAAATTTAT
AAACGCCGCAAATAActtgggagaccaacta

Resulting tagged protein (His$_6$-tag, linker with thrombin cut site), 366 AA, 41.8 kDa, ε_{280} = 54.8 mM^{-1} cm^{-1}

MGSSHHHHHHGSGLVPRGSAGMYAQEKQDFVQHFSQIVRVLTEDEMGHPEIGDAIARLKEVLEYNAIGGKYNRGLTVVVAFRE
LVEPRKQDADSLQRAWTVGWCVELLQAFFLVADDIMDSSLTRRGQICWYQKPGVGLDAINDANLLEACIYRLLKLYCREQPYYL
NLIELFLQSSYQTEIGQTLDLLTAPQGNVDLVRFTEKRYKSIVKYKTAFYSFYLPIAAAMYMAGIDGEKEHANAKKILLEMGEFFQI
QDDYLDLFGDPSVTGKIGTDIQDNKCSWLVVQCLQRATPEQYQILKENYGQKEAEKVARVKALYEELDLPAVFLQYEEDSYSHI
MALIEQYAAPLPPAVFLGLARKIYKRRK*

***Methanothermobacter thermautotrophicus* polyprenyl pyrophosphate synthase** (*Mt*polyPPPS)

PDB - , UniProt IDSA_METTH, accession WP_010875690.1

MMEVMDILRKYSEMADERIRESISDITPETLLRASEHLITAGGKKIRPSLALLSSEAVGGDPGDAAGVAAAIELIHTFSLIHDDIMDD
DEIRRGEPAVHVLWGEPMAILAGDVLFSKAFEAVIRNGDSEMVKEALAVVVDSCVKICEGQALDMGFEERLDVTEEEYMEMIYK
KTAALIAAATKAGAIMGGGSPQEIAALEDYGRCIGLAFQIHDDYLDVVSDEESLGKPVGSDIAEGKMTLMVVKALERASEKDRER
LISILGSGDEKLVAEAIEIFERYGATEYAHAVALDHVRMAKERLEVLEESDAREALAMIADFVLEREH

Codon-optimized sequence (Start and stop codon, cut away by BsaI, appended sequence)

ctaaccggtctcctggtATGATGGAAGTTATGGATATTCTGCGCAAATATAGCGAAATGGCGGATGAACGTATTCGTGAAAGCATCA
GCGATATTACCCCGGAAACCCTGCTGCGCGCGTCAGAACATCTGATTACCGCGGGCGGCAAAAAAATCCGCCCGAGCCT
GGCGCTGCTGAGCAGCGAAGCGGTCGGCGGCGATCCGGGCGATGCGGCGGGCGTGGCGGCGGCAATCGAACTGATTC
ACACCTTTAGCCTGATTCACGATGATATTATGGATGATGATGAAATTCGCCGCGGTGAACCGGCGGTGCATGTTCTGTGG
GGCGAACCGATGGCCATTCTGGCGGGTGATGTGCTGTTTAGCAAAGCGTTTGAAGCGGTCATCCGTAACGGCGATAGCG
AAATGGTGAAAGAAGCGCTGGCCGTGGTGGTGGATAGCTGCGTGAAAATTTGCGAAGGCCAGGCGCTGGATATGGGCTT
TGAAGAACGCCTGGATGTCACCGAAGAAGAATATATGGAAATGATTTATAAAAAAACCGCCGCGCTGATTGCGGCGGCGA
CCAAAGCGGGTGCGATTATGGGCGGCGGCAGCCCGCAGGAAATTGCGGCGCTGGAAGATTATGGCCGCTGCATTGGTC
TGGCCTTTCAGATCCATGATGATTATCTGGATGTTGTCAGCGATGAAGAATCGCTGGGCAAACCGGTGGGCAGCGATATT
GCAGAAGGCAAAATGACCCTGATGGTGGTGAAAGCGCTGGAACGCGCGAGCGAAAAAGACCGTGAACGCCTGATCAGCA
TCCTGGGCAGCGGCGATGAAAAACTGGTGGCCGAAGCCATTGAAATTTTCGAACGCTATGGTGCAACCGAATATGCGCAT
GCGGTGGCGCTGGATCATGTGCGTATGGCGAAGGAACGTCTGGAAGTGCTGGAAGAAAGCGATGCGCGCGAAGCGCTG
GCGATGATTGCGGATTTTGTGTTGGAACGTGAACATTAActtgggagaccaacta

Resulting tagged protein (His6-tag, linker with thrombin cut site), 346 AA, 37.6 kDa, ε_{280} = 15.9 mM^{-1} cm^{-1}

MGSSHHHHHHGSGLVPRGSAGMMEVMDILRKYSEMADERIRESISDITPETLLRASEHLITAGGKKIRPSLALLSSEAVGGDPG
DAAGVAAAIELIHTFSLIHDDIMDDDEIRRGEPAVHVLWGEPMAILAGDVLFSKAFEAVIRNGDSEMVKEALAVVVDSCVKICEGQ
ALDMGFEERLDVTEEEYMEMIYKKTAALIAAATKAGAIMGGGSPQEIAALEDYGRCIGLAFQIHDDYLDVVSDEESLGKPVGSDI
AEGKMTLMVVKALERASEKDRERLISILGSGDEKLVAEAIEIFERYGATEYAHAVALDHVRMAKERLEVLEESDAREALAMIADF
VLEREH*

***Neurospora crassa* farnesyl pyrophosphate synthase** (*Nc*FPPS)

PDB - , UniProt FPPS_NEUCR, accession XP_009848083.1

MAKTTTLKEFESVFPKLEEALLEYAKAYKLPEQMLSWYKQSLEVNTLGGKCNRGMSVPDSASILLGRPLTEEEYFQAATLGWM
TELLQAFFLVSDDIMDSSITRRGKPCWYRQEGVGMVAINDAFMLESAIYTLLKKYFRSHPRYVDFLELFHEVTFQTEMGQLCDLL
TAPEDKVDLDNFSMDKYTFIVIYKTAYYSFYLPVALAMYMLDIATPENLKQAEDILIPLGEYFQVQDDYLDNFGLPEHIGKIGTDIQ
DNKCSWLVNKALSIVTPEQRKTLEENYGRKDKAKEAVIKQLYDDLKLEDHYKQYEEERVGEIRKMIDAIDESKGLKKQVFEAFLG
KIYKRSK

Codon-optimized sequence (Start and stop codon, cut away by BsaI, appended sequence)

ctaaccggtctcctggtATGGCGAAAACCACCACCCTGAAAGAATTTGAGAGCGTTTTTCCGAAACTGGAAGAAGCGCTGCTGGAA
TATGCCAAAGCGTATAAACTGCCGGAACAGATGCTGAGCTGGTATAAACAGTCACTGGAAGTGAATACCCTGGGTGGCAA
ATGCAACCGCGGCATGAGCGTGCCGGATAGCGCGAGCATCCTGCTGGGCCGCCCGCTGACCGAAGAAGAATATTTTCAG
GCGGCGACCCTGGGTTGGATGACCGAACTGCTGCAAGCATTTTTCCTGGTGAGCGATGATATTATGGATTCGAGCATTAC
CCGTCGCGGCAAACCGTGCTGGTATCGCCAGGAAGGCGTTGGCATGGTGGCCATTAATGATGCCTTTATGCTGGAAAGC
GCGATTTATACCCTGCTGAAAAAATATTTCCGCAGCCACCCGCGCTACGTGGACTTTCTGGAACTGTTTCACGAAGTGACC
TTTCAGACCGAAATGGGTCAGCTGTGTGATCTGCTGACCGCGCCGGAAGATAAAGTGGATCTGGATAATTTTAGCATGGA
TAAATATACCTTTATTGTGATTTACAAAACCGCGTACTATAGCTTTTATCTGCCGGTGGCGCTGGCCATGTATATGCTGGAC
ATTGCGACCCCGGAAAACCTGAAACAGGCGGAAGACATTCTGATTCCGCTGGGCGAATACTTCCAGGTTCAGGATGATTA
CCTGGATAATTTTGGCCTGCCGGAACACATTGGCAAAATTGGCACGGATATTCAGGATAACAAATGCAGCTGGCTGGTGA
ACAAAGCCCTGAGCATTGTGACCCCGGAACAACGCAAAACCCTGGAAGAGAACTATGGTCGTAAAGATAAAGCCAAAGAA
GCGGTGATTAAACAGCTGTATGATGATCTGAAGCTGGAAGACCACTATAAACAGTATGAAGAAGAACGTGTGGGCGAAAT
TCGCAAAATGATTGATGCGATCGATGAAAGCAAAGGTCTGAAAAAACAGGTGTTCGAAGCCTTTCTGGGCAAAATCTACAA
ACGCAGCAAATAActtgggagaccaacta

Resulting tagged protein (His$_6$-tag, linker with thrombin cut site), 368 AA, 42.4 kDa, ε_{280} = 53.3 mM^{-1} cm^{-1}

MGSSHHHHHHGSGLVPRGSAGMAKTTTLKEFESVFPKLEEALLEYAKAYKLPEQMLSWYKQSLEVNTLGGKCNRGMSVPDS
ASILLGRPLTEEEYFQAATLGWMTELLQAFFLVSDDIMDSSITRRGKPCWYRQEGVGMVAINDAFMLESAIYTLLKKYFRSHPRY
VDFLELFHEVTFQTEMGQLCDLLTAPEDKVDLDNFSMDKYTFIVIYKTAYYSFYLPVALAMYMLDIATPENLKQAEDILIPLGEYFQ
VQDDYLDNFGLPEHIGKIGTDIQDNKCSWLVNKALSIVTPEQRKTLEENYGRKDKAKEAVIKQLYDDLKLEDHYKQYEEERVGEI
RKMIDAIDESKGLKKQVFEAFLGKIYKRSK*

***Rhodobacter capsulatus* farnesyl pyrophosphate synthase** (*Rc*FPPS)

PDB 3lvs, UniProt Q9KWR7_RHOCA, accession WP_013069157.1

MFSERLKEIQDAVETAMAAAIGRLPAGDLRDAMAYAAQGGKRLRAFLAIESAAIHGISMAQAMPAALAVEALHAYSLVHDDMPC
MDNDDLRRGLPTVHKKWDDATAVLAGDALQTLAFELCTDPVLGSAENRVALVAALAQASGAEGMVYGQALDIAAETAAVPLTL
DEIIRLQAGKTGALISFAAQAGAILAGADRGPLTAYATALGLAFQIADDILDVEGNEEAAGKRLGKDAEAHKATFVSLLGLAGAKS
RAADLVAEAEAALAPYGEAASTLRACARYVIERDK

Codon-optimized sequence (Start and stop codon, cut away by BsaI, appended sequence)

ctaaccggtctcctggtATGTTTAGCGAACGCCTGAAAGAAATTCAGGATGCGGTGGAAACCGCGATGGCGGCGGCAATTGGCCG
TCTGCCGGCAGGCGACCTGCGCGATGCGATGGCCTATGCGGCGCAAGGCGGTAAACGCTTGCGTGCGTTTCTGGCGAT
TGAATCGGCAGCGATTCATGGCATTAGCATGGCGCAGGCCATGCCGGCGGCCCTGGCTGTGGAAGCCCTGCATGCCTAT
TCACTGGTACATGATGATATGCCGTGCATGGATAACGATGATCTGCGCCGTGGTCTGCCGACGGTTCATAAAAAATGGGA
TGATGCGACCGCGGTGCTGGCGGGCGATGCCCTGCAGACCCTGGCGTTTGAACTGTGTACCGATCCGGTCCTGGGCAG
CGCGGAAAACCGCGTGGCCTTAGTGGCGGCCCTGGCGCAGGCCAGCGGCGCCGAAGGCATGGTGTATGGCCAAGCGT
TAGATATTGCCGCCGAAACCGCGGCGGTTCCGCTGACTCTGGATGAAATTATTCGCCTGCAGGCCGGTAAAACGGGCGC
CCTGATTAGCTTTGCCGCACAGGCAGGTGCGATCCTGGCCGGTGCGGATCGTGGCCCGCTGACCGCCTATGCCACGGC
CCTGGGCCTGGCATTTCAGATTGCGGATGATATTCTGGATGTTGAAGGCAATGAAGAAGCAGCCGGCAAACGCCTGGGG
AAAGATGCCGAGGCGCACAAAGCAACCTTCGTGAGCCTGCTGGGCCTGGCCGGCGCGAAAAGCCGTGCCGCCGATCTG
GTGGCAGAAGCCGAAGCCGCACTGGCGCCGTATGGTGAAGCCGCAAGCACCCTGCGGGCATGTGCGCGCTATGTGATT
GAACGCGATAAATAActtgggagaccaacta

Resulting tagged protein (His$_6$-tag, linker with thrombin cut site), 309 AA, 31.9 kDa, ε_{280} = 14.4 mM^{-1} cm^{-1}

MGSSHHHHHHGSGLVPRGSAGMFSERLKEIQDAVETAMAAAIGRLPAGDLRDAMAYAAQGGKRLRAFLAIESAAIHGISMAQA
MPAALAVEALHAYSLVHDDMPCMDNDDLRRGLPTVHKKWDDATAVLAGDALQTLAFELCTDPVLGSAENRVALVAALAQASG
AEGMVYGQALDIAAETAAVPLTLDEIIRLQAGKTGALISFAAQAGAILAGADRGPLTAYATALGLAFQIADDILDVEGNEEAAGKRL
GKDAEAHKATFVSLLGLAGAKSRAADLVAEAEAALAPYGEAASTLRACARYVIERDK*

Spodoptera frugiperda (Fall armyworm) **farnesyl pyrophosphate synthase** (*Sf*FPPS)

PDB - , UniProt A0A9R0EV28_SPOFR, accession XP_050550682.1 (~~leader peptide~~, added **M**)

~~MNIPGKMVASSLMMKSIGTVMTRNWVESTFQRFCATTTPSAKKFD~~**M**LKEEKAEFLNALPGVIDLFSKHEKFHEVPGTEKWMRN
VLNGNLIGGKNMRGLTTVMTYKFIEKPENINEETLRLARTLGWCAEILQAYCLVLDDIADGSLTRRGLPCWYRREDVGIAHAVND
ATLIHYSLLHLLRVNFEKSPYYNDLYHNFNETLFYTCLGQYLDIMTGMKKKNYDLFTMDQYNDIVKYKSAYYTYKLPITAGLMLAN
QFNEETHKDSDEISMQLGRLFQMQDDYIDCFGDENMTGKIGSDIQEGKCSWLAVKALQHCKPNQRAVFSACYGSHEPAHVERI
KQLYVQLKIPQMYKEEENEIYNNIVKRIKSVSSKSHQELFLRVLHDTYGRKH

Codon-optimized sequence (Start and stop codon, cut away by BsaI, appended sequence)

ctaaccggtctcctggtATGCTGAAAGAGGAGAAAGCGGAATTTCTGAATGCGCTGCCGGGTGTGATTGACCTGTTCAGCAAACAT
GAAAAATTTCATGAAGTGCCGGGCACCGAAAAATGGATGCGCAATGTTCTGAACGGCAACCTGATTGGCGGTAAAAACAT
GCGCGGCCTGACCACCGTGATGACCTACAAATTTATTGAAAAACCGGAAAACATTAATGAAGAAACCCTGCGTCTGGCGC
GCACCCTGGGCTGGTGCGCGGAAATTCTGCAGGCCTATTGTCTGGTTCTGGATGATATCGCGGATGGCTCCCTGACCCG
CCGCGGCCTGCCGTGTTGGTACCGCCGTGAAGACGTGGGCATTGCGCATGCGGTGAATGATGCGACCCTGATTCATTAC
AGCCTGCTGCACCTGCTGCGTGTGAATTTTGAAAAAAGCCCGTACTACAACGATCTGTATCATAATTTTAACGAAACCCTG
TTTTACACCTGCCTGGGCCAGTACCTGGATATTATGACCGGCATGAAAAAAAAAAATTACGATCTGTTTACCATGGATCAG
TATAACGATATTGTTAAATATAAAAGCGCCTACTATACCTACAAACTGCCGATTACCGCCGGCCTGATGCTGGCCAACCAG
TTTAATGAAGAAACCCATAAAGATTCGGATGAAATTAGCATGCAGCTGGGCCGCCTGTTTCAGATGCAGGATGATTATATT
GATTGCTTTGGCGATGAAAACATGACCGGCAAAATTGGCAGCGATATTCAGGAAGGCAAATGCAGCTGGCTGGCGGTGA
AAGCGCTGCAGCACTGTAAACCGAACCAGCGCGCGGTGTTTAGCGCATGCTACGGCAGCCATGAACCGGCGCATGTGGA
ACGCATTAAACAGCTGTATGTGCAGCTCAAAATCCCGCAGATGTACAAAGAAGAAGAAAATGAAATTTACAATAACATTGT
GAAACGCATCAAAAGCGTGAGCAGCAAAAGCCACCAGGAACTGTTCCTGCGCGTGCTGCATGATACCTATGGCCGCAAA
CATTAActtgggagaccaacta

Resulting tagged protein (His$_6$-tag, linker with thrombin cut site), 365 AA, 42.2 kDa, ε_{280} = 53.3 mM^{-1} cm^{-1}

MGSSHHHHHHGSGLVPRGSAGMLKEEKAEFLNALPGVIDLFSKHEKFHEVPGTEKWMRNVLNGNLIGGKNMRGLTTVMTYKF
IEKPENINEETLRLARTLGWCAEILQAYCLVLDDIADGSLTRRGLPCWYRREDVGIAHAVNDATLIHYSLLHLLRVNFEKSPYYND
LYHNFNETLFYTCLGQYLDIMTGMKKKNYDLFTMDQYNDIVKYKSAYYTYKLPITAGLMLANQFNEETHKDSDEISMQLGRLFQ
MQDDYIDCFGDENMTGKIGSDIQEGKCSWLAVKALQHCKPNQRAVFSACYGSHEPAHVERIKQLYVQLKIPQMYKEEENEIYN
NIVKRIKSVSSKSHQELFLRVLHDTYGRKH*

***Saccharomyces cerevisiae* farnesyl pyrophosphate synthase** (*Sc*FPPS)

PDB - , UniProt ERG20_YEAST, accession KAF4004038.1

MASEKEIRRERFLNVFPKLVEELNASLLAYDMPKEARDWYAHSLNYNTPGGKLNRGLSVVDTYAILSNKTVEQLGQEEYEKVAI
LGWCIELLQAYFLVADDMMDKSITRRGQPCWYKVPEVGEIAINDAFMLEAAIYKLLKSHFRNEKYYIDITELFHEVTFQTELGQLM
DLITAPEDKVDLSKFSLKKHSFIVTFKTAYYSFYLPVALAMYVAGITDEKDLKQARDVLIPLGEYFQIQDDYLDCFGTPEQIGKIGT
DIQDNKCSWVINKALELASAEQRKTLDENYGKKDSVAEAKCKKIFNDLKIEQLYHEYEESIAKDLKAKISQVDESRGFKADVLTAF
LNKVYKRSK

Codon-optimized sequence (Start and stop codon, cut away by BsaI, appended sequence)

ctaaccggtctcctggtATGGCGAGCGAAAAAGAAATTCGCCGTGAACGCTTTCTCAACGTGTTTCCGAAACTGGTGGAAGAGCTG
AACGCGAGCCTGCTGGCGTATGATATGCCGAAAGAAGCGCGCGATTGGTATGCGCATTCGCTGAACTACAATACCCCGG
GCGGCAAACTGAACCGCGGCCTGAGCGTGGTCGATACCTATGCGATTCTGAGCAATAAAACCGTGGAACAGCTGGGCCA
GGAAGAATACGAAAAGGTGGCGATTCTGGGCTGGTGCATTGAACTGCTGCAGGCGTATTTTCTGGTGGCGGATGATATGA
TGGACAAATCAATTACCCGCCGTGGCCAGCCGTGCTGGTATAAAGTGCCGGAAGTGGGCGAAATTGCGATTAACGATGC
CTTTATGCTGGAAGCGGCCATTTACAAACTGCTGAAAAGCCATTTTCGCAACGAAAAATACTATATCGATATTACCGAACTG
TTTCATGAAGTGACCTTTCAGACCGAACTGGGCCAGCTGATGGATCTGATTACCGCGCCGGAAGATAAAGTGGATCTGAG
CAAATTTAGCCTGAAAAAACACTCGTTTATCGTTACCTTTAAAACCGCGTACTATAGCTTCTATCTGCCGGTAGCGCTGGC
GATGTACGTGGCCGGCATTACCGATGAAAAAGATCTGAAACAGGCGCGTGATGTTCTGATTCCGCTGGGCGAATATTTTC
AGATTCAGGATGATTATCTGGATTGCTTTGGCACCCCGGAACAGATTGGCAAAATTGGCACCGATATTCAGGATAATAAAT
GTAGCTGGGTGATTAATAAAGCGCTGGAACTGGCGAGCGCAGAACAGCGCAAAACCCTGGATGAAAATTATGGCAAAAAA
GATAGCGTAGCCGAAGCGAAATGCAAAAAAATTTTTAATGATCTGAAAATCGAACAGCTGTATCATGAATACGAAGAAAGC
ATCGCCAAAGATCTGAAAGCAAAAATTTCGCAGGTGGACGAAAGCCGCGGTTTTAAAGCGGATGTTCTGACCGCGTTTCT
GAATAAAGTTTATAAACGCAGCAAATAActtgggagaccaacta

Resulting tagged protein (His$_6$-tag, linker with thrombin cut site), 373 AA, 42.7 kDa, ε_{280} = 51.8 mM^{-1} cm^{-1}

MGSSHHHHHHGSGLVPRGSAGMASEKEIRRERFLNVFPKLVEELNASLLAYDMPKEARDWYAHSLNYNTPGGKLNRGLSVVD
TYAILSNKTVEQLGQEEYEKVAILGWCIELLQAYFLVADDMMDKSITRRGQPCWYKVPEVGEIAINDAFMLEAAIYKLLKSHFRNE
KYYIDITELFHEVTFQTELGQLMDLITAPEDKVDLSKFSLKKHSFIVTFKTAYYSFYLPVALAMYVAGITDEKDLKQARDVLIPLGEY
FQIQDDYLDCFGTPEQIGKIGTDIQDNKCSWVINKALELASAEQRKTLDENYGKKDSVAEAKCKKIFNDLKIEQLYHEYEESIAKD
LKAKISQVDESRGFKADVLTAFLNKVYKRSK*

***Schizosaccharomyces pombe* farnesyl pyrophosphate synthase** (*Sp*FPPS)

PDB - , UniProt ERG20_SCHPO, accession NP_593299.1

MSAVDKRAKFESALPVFVDEIVNYLKTINIPDDVTEWYKNSLFHNTLGGKYNRGLSVIDSYEILLGHPLDEAAYMKAAVLGWMVE
LLQSFFLIADDIMDASKTRRGQPCWYLMPGVGNIAINDAFMVESAIYFLLKKHFRQESCYVDLIELFHDVTFQTELGQQLDLLTAP
EDSVDLSKFSLQKHSFIVIYKTAFYSFYLPVALAMHLAGVATPENLKCAQDILIILGKYFQVQDDYLDCYGDPTVTGKIGTDILDNK
CSWIINLALAKCTPEQRVILDDNYGRKDSESEKRVKAVFEELNIRGEFENYEESEVSEIKKLIDGVDESTGLKKSIFTTFLGKIYKR
NK

Codon-optimized sequence (Start and stop codon, cut away by BsaI, appended sequence)

ctaaccggtctcctggtATGAGCGCGGTGGATAAACGTGCGAAATTTGAAAGCGCGCTGCCGGTGTTTGTGGATGAAATTGTGAAC
TACCTGAAAACCATTAATATTCCGGATGACGTGACCGAATGGTATAAAAACAGCCTGTTTCATAACACCCTGGGCGGCAAA
TACAACCGCGGCCTCAGCGTGATTGATAGCTACGAAATTCTGCTGGGCCATCCGCTGGATGAAGCGGCGTATATGAAAGC
GGCGGTGCTGGGCTGGATGGTGGAGCTGCTGCAGAGCTTCTTTCTGATCGCGGATGATATCATGGACGCGAGCAAAACC
CGTCGTGGCCAGCCGTGCTGGTACCTGATGCCGGGCGTGGGCAATATTGCGATTAACGACGCCTTCATGGTGGAAAGCG
CCATTTATTTCCTGCTGAAAAAACATTTTCGCCAGGAAAGCTGCTATGTGGATCTGATTGAACTGTTTCATGATGTGACCTT
TCAGACCGAACTGGGCCAGCAGCTGGATCTGCTGACCGCGCCGGAAGATAGCGTGGATCTGTCCAAATTTAGCCTGCAG
AAACATAGCTTTATTGTCATCTATAAAACCGCGTTCTACAGCTTTTACCTGCCGGTGGCGCTGGCCATGCATCTGGCGGGC
GTGGCGACCCCGGAAAACCTGAAATGCGCCCAGGATATTCTGATTATTCTGGGCAAATACTTTCAGGTGCAGGATGATTA
CCTGGACTGCTATGGCGACCCGACCGTGACCGGCAAAATTGGCACCGATATTCTGGATAACAAATGCAGCTGGATTATTA
ACCTGGCTCTGGCGAAATGCACCCCGGAACAGCGCGTGATTCTGGATGATAATTATGGCCGCAAAGATAGCGAAAGCGA
AAAACGCGTGAAAGCGGTGTTTGAAGAACTGAATATTCGCGGCGAATTTGAAAACTACGAAGAAAGTGAAGTGAGCGAAA
TTAAGAAACTGATTGATGGCGTGGACGAATCTACCGGCCTGAAAAAAAGCATTTTTACAACCTTTCTGGGCAAAATTTACAA
ACGTAACAAATAActtgggagaccaacta

Resulting tagged protein (His$_6$-tag, linker with thrombin cut site), 368 AA, 41.6 kDa, ε_{280} = 47.3 mM^{-1} cm^{-1}

MGSSHHHHHHGSGLVPRGSAGMSAVDKRAKFESALPVFVDEIVNYLKTINIPDDVTEWYKNSLFHNTLGGKYNRGLSVIDSYEI
LLGHPLDEAAYMKAAVLGWMVELLQSFFLIADDIMDASKTRRGQPCWYLMPGVGNIAINDAFMVESAIYFLLKKHFRQESCYVD
LIELFHDVTFQTELGQQLDLLTAPEDSVDLSKFSLQKHSFIVIYKTAFYSFYLPVALAMHLAGVATPENLKCAQDILIILGKYFQVQD
DYLDCYGDPTVTGKIGTDILDNKCSWIINLALAKCTPEQRVILDDNYGRKDSESEKRVKAVFEELNIRGEFENYEESEVSEIKKLID
GVDESTGLKKSIFTTFLGKIYKRNK*

***Trypanosoma cruzi* farnesyl pyrophosphate synthase** (*Tc*FPPS)

truncated as per PDB entry, PDB 1yhk, UniProt Q8WS26_TRYCR, accession number EKG07068.1

MASMERFLSVYDEVQAFLLDQLQSKYEIDPNRARYLRIMMDTTCLGGKYFRGMTVVNVAEGFLAVTQHDEATKERILHDACVG
GWMIEFLQAHYLVEDDIMDGSVMRRGKPCWYRFPGVTTQCAINDGIILKSWTQIMAWHYFADRPFLKDLLCLFQKVDYATAIGQ
MYDVTSMCDSNKLDPEVAQPMTTDFAEFTPAIYKRIVKYKTTFYTYLLPLVMGLLISEAAASVEMNLVERVAHLIGEYFQVQDDV
MDCFTPPEQLGKVGTDIEDAKCSWLAVTFLGKANAAQVAEFKANYGEKDPAKVAVVKRLYSEANLQADFAAYEAEVVREVESL
IEQLKVKSPTFAESVAVVWEKTHKRKK

Codon-optimized sequence (Start and stop codon, cut away by BsaI, appended sequence)

ctaaccggtctcctggtATGGCGAGCATGGAACGCTTTCTGTCGGTATACGATGAAGTGCAGGCCTTTCTGCTGGATCAGCTGCAG
AGCAAATATGAAATTGATCCGAACCGCGCGCGTTACCTGCGCATTATGATGGATACCACCTGTCTGGGCGGTAAATATTTT
CGTGGCATGACCGTGGTGAACGTGGCCGAAGGTTTTCTGGCCGTGACCCAGCACGATGAAGCCACCAAAGAACGTATTC
TGCATGATGCCTGCGTGGGTGGCTGGATGATTGAATTCCTGCAGGCACATTATCTGGTGGAAGATGATATTATGGATGGT
AGCGTGATGCGTCGCGGCAAACCGTGCTGGTATCGCTTTCCGGGCGTGACCACCCAATGCGCGATTAATGATGGCATTA
TCCTGAAAAGCTGGACGCAGATTATGGCCTGGCATTATTTCGCGGACCGCCCGTTTCTGAAAGATCTGCTGTGTCTGTTC
CAGAAAGTGGATTATGCGACCGCCATTGGCCAGATGTACGATGTGACCAGTATGTGTGATAGCAACAAACTGGATCCGGA
AGTTGCGCAGCCGATGACCACCGATTTTGCGGAATTCACCCCGGCGATTTATAAACGCATTGTAAAATACAAAACCACCTT
TTATACCTATCTGCTGCCGCTGGTTATGGGCCTGCTGATTAGCGAAGCCGCGGCCAGCGTGGAAATGAACCTGGTGGAA
CGTGTGGCCCATCTGATTGGTGAATACTTTCAGGTGCAGGATGATGTGATGGATTGTTTTACCCCGCCGGAGCAGCTGGG
CAAAGTGGGTACCGATATTGAAGATGCGAAATGCAGCTGGCTGGCCGTTACCTTTCTGGGCAAAGCGAACGCCGCCCAG
GTAGCGGAATTCAAAGCGAACTACGGCGAAAAAGATCCGGCGAAAGTGGCGGTGGTGAAACGTCTGTACAGCGAAGCGA
ACCTGCAGGCGGATTTTGCGGCGTATGAAGCCGAAGTGGTTCGCGAAGTCGAAAGCCTGATTGAACAGCTGAAAGTGAA
AAGCCCGACCTTTGCCGAAAGCGTGGCCGTGGTGTGGGAAAAAACCCATAAACGCAAAAAATAActtgggagaccaacta

Resulting tagged protein (His$_6$-tag, linker with thrombin cut site), 383 AA, 43.3 kDa, ε_{280} = 58.3 mM^{-1} cm^{-1}

MGSSHHHHHHGSGLVPRGSAGMASMERFLSVYDEVQAFLLDQLQSKYEIDPNRARYLRIMMDTTCLGGKYFRGMTVVNVAE
GFLAVTQHDEATKERILHDACVGGWMIEFLQAHYLVEDDIMDGSVMRRGKPCWYRFPGVTTQCAINDGIILKSWTQIMAWHYF
ADRPFLKDLLCLFQKVDYATAIGQMYDVTSMCDSNKLDPEVAQPMTTDFAEFTPAIYKRIVKYKTTFYTYLLPLVMGLLISEAAAS
VEMNLVERVAHLIGEYFQVQDDVMDCFTPPEQLGKVGTDIEDAKCSWLAVTFLGKANAAQVAEFKANYGEKDPAKVAVVKRLY
SEANLQADFAAYEAEVVREVESLIEQLKVKSPTFAESVAVVWEKTHKRKK*

***(Para)geobacillus thermoglucosidasius* pyrimidine nucleoside phosphorylase** (*Gt*PyNP)

PDB 7m7k, UniProt A0A1B7KX91_PARTM, accession number WP_041270053.1

MRMVDLIAKKRDGYELSKEEIDFIIRGYTNGDIPDYQMSAFAMAVFFRGMTEEETAALTMAMVRSGDVIDLSKIEGMKVDKHSTG
GVGDTTTLVLGPLVASVGVPVAKMSGRGLGHTGGTIDKLESVPGFHVEIDNEQFIELVNKNKIAIIGQTGNLTPADKKLYALRDVT
ATVDSIPLIASSIMSKKIAAGADAIVLDVKTGAGAFMKDFAGAKRLATAMVEIGKRVGRKTMAVISDMSQPLGYAVGNALEVKEAI
DTLKGKGPEDLQELCLTLGSYMVYLAEKASSLEEARALLEASIREGKALETFKVFLSAQGGDASVVDDPTKLPQAKYRWELEAP
EDGYVAEIVADEVGTAAMLLGAGRATKEATIDLSVGLVLHKKVGDAVKKGESLVTIYSNTENIEEVKQKLAKSIRLSSIPVAKPTLI
YETIS

Codon-optimized sequence (Start and stop codon, cut away by BsaI, appended sequence)

ctaaccggtctcctggtATGCGTATGGTGGATCTGATTGCAAAAAAACGCGATGGCTATGAACTGTCTAAAGAAGAAATTGATTTTA
TTATTCGCGGCTATACCAACGGCGATATTCCGGATTATCAGATGAGCGCCTTTGCAATGGCGGTGTTTTTTCGCGGCATGA
CCGAAGAAGAAACCGCCGCGCTGACCATGGCGATGGTGCGCAGCGGCGATGTGATTGATCTGAGCAAAATCGAAGGTAT
GAAAGTGGATAAACACAGCACCGGCGGTGTGGGCGACACCACCACCCTGGTGCTGGGCCCGCTGGTTGCGAGCGTGGG
CGTACCGGTCGCGAAAATGAGCGGTCGCGGCCTGGGCCATACGGGCGGCACCATTGACAAACTGGAAAGCGTGCCGGG
CTTTCATGTGGAAATTGATAACGAACAGTTTATTGAACTGGTAAACAAAAATAAAATCGCGATTATTGGCCAGACCGGTAAC
CTGACGCCGGCGGATAAAAAACTGTACGCGCTGCGCGATGTGACCGCGACCGTTGATAGCATTCCGCTGATTGCCAGCA
GCATTATGAGCAAAAAAATTGCGGCGGGCGCGGATGCGATTGTGCTGGATGTGAAAACCGGCGCGGGCGCGTTTATGAA
AGATTTTGCGGGCGCCAAACGTCTGGCGACCGCCATGGTGGAAATTGGTAAACGCGTCGGTCGCAAAACCATGGCGGTG
ATTAGCGATATGAGCCAGCCGCTGGGCTATGCCGTGGGTAATGCCCTGGAAGTTAAAGAAGCCATTGACACCCTGAAAG
GCAAAGGCCCGGAAGATCTGCAGGAACTGTGCCTGACCCTGGGCAGCTACATGGTGTATCTGGCGGAAAAAGCCAGCTC
GCTGGAAGAAGCCCGCGCGCTGCTTGAAGCCTCAATTCGCGAAGGCAAAGCGCTGGAAACCTTCAAAGTGTTTCTGAGC
GCCCAGGGCGGCGATGCGAGCGTGGTGGATGATCCAACCAAACTGCCGCAGGCGAAATATCGTTGGGAACTGGAAGCG
CCGGAAGATGGTTATGTGGCGGAAATTGTTGCCGATGAAGTGGGCACCGCAGCGATGCTGCTGGGCGCGGGCCGTGCC
ACCAAAGAAGCGACCATTGATCTGAGCGTGGGTCTGGTTCTGCATAAAAAAGTGGGCGATGCGGTTAAAAAAGGCGAATC
GCTGGTGACCATTTACTCAAACACGGAAAATATTGAAGAAGTGAAACAAAAACTGGCCAAAAGCATTCGCCTGAGCAGCAT
TCCGGTGGCGAAACCGACCCTGATTTACGAAACCATTAGCTAActtgggagaccaacta

Resulting tagged protein (His6-tag, linker with thrombin cut site), 454 AA, 48.3 kDa, ε_{280} = 21.9 mM^{-1} cm^{-1}

MGSSHHHHHHGSGLVPRGSAGMRMVDLIAKKRDGYELSKEEIDFIIRGYTNGDIPDYQMSAFAMAVFFRGMTEEETAALTMAM
VRSGDVIDLSKIEGMKVDKHSTGGVGDTTTLVLGPLVASVGVPVAKMSGRGLGHTGGTIDKLESVPGFHVEIDNEQFIELVNKN
KIAIIGQTGNLTPADKKLYALRDVTATVDSIPLIASSIMSKKIAAGADAIVLDVKTGAGAFMKDFAGAKRLATAMVEIGKRVGRKTM
AVISDMSQPLGYAVGNALEVKEAIDTLKGKGPEDLQELCLTLGSYMVYLAEKASSLEEARALLEASIREGKALETFKVFLSAQGG
DASVVDDPTKLPQAKYRWELEAPEDGYVAEIVADEVGTAAMLLGAGRATKEATIDLSVGLVLHKKVGDAVKKGESLVTIYSNTE
NIEEVKQKLAKSIRLSSIPVAKPTLIYETIS*

***(Para)geobacillus thermoglucosidasius* inorganic pyrophosphatase** (*Gt*IPP)

PDB - , UniProt A0A1B7KXJ2_PARTM, accession WP_064550122.1

MAFENKVVEAFIEIPTGSQNKYEFDKERGIFKLDRVLYSPMFYPAEYGYLQNTLALDGDPLDILVITTNPTFPGCVIDTRVIGYLNM
IDSGEEDAKLIGVPVEDPRFDEVRSIEDLPQHKLKEIAHFFERYKDLQGKRTEIGAWEGPEAAAKLIDECIARYNENKNK

Codon-optimized sequence (Start and stop codon, cut away by BsaI, appended sequence)

ctaaccggtctcctggtATGGCGTTTGAAAATAAAGTGGTGGAAGCGTTCATTGAAATTCCGACCGGCAGCCAGAATAAATATGAAT
TTGATAAAGAACGCGGCATTTTTAAACTGGATCGCGTGCTGTATAGCCCGATGTTTTATCCGGCGGAATATGGCTATCTGC
AGAACACCCTGGCGCTGGATGGCGATCCGCTGGATATTCTGGTGATTACCACCAACCCGACCTTTCCGGGTTGCGTGATC
GATACCCGCGTCATTGGCTATCTGAACATGATCGATAGCGGCGAGGAAGATGCCAAACTGATTGGCGTGCCGGTGGAAG
ATCCGCGTTTTGATGAAGTGCGCAGCATTGAAGATCTGCCGCAGCATAAACTGAAAGAAATTGCGCACTTTTTTGAACGTT
ACAAAGATCTGCAGGGCAAACGCACCGAAATTGGCGCGTGGGAAGGCCCGGAAGCGGCGGCGAAACTGATTGATGAAT
GTATTGCGCGTTACAACGAAAACAAAAATAAATAActtgggagaccaacta

Resulting tagged protein (His$_6$-tag, linker with thrombin cut site), 188 AA, 21.3 kDa, ε_{280} = 17.4 mM^{-1} cm^{-1}

MGSSHHHHHHGSGLVPRGSAGMAFENKVVEAFIEIPTGSQNKYEFDKERGIFKLDRVLYSPMFYPAEYGYLQNTLALDGDPLDI
LVITTNPTFPGCVIDTRVIGYLNMIDSGEEDAKLIGVPVEDPRFDEVRSIEDLPQHKLKEIAHFFERYKDLQGKRTEIGAWEGPEA
AAKLIDECIARYNENKNK*

***Escherichia coli* alkaline phosphatase** (*Ec*AP)

PDB 1aja, UniProt PPB_ECOLI, accession WP_000814403.1

MKQSTIALALLPLLFTPVTKARTPEMPVLENRAAQGDITAPGGARRLTGDQTAALRDSLSDKPAKNIILLIGDGMGDSEITAARNY
AEGAGGFFKGIDALPLTGQYTHYALNKKTGKPDYVTDSAASATAWSTGVKTYNGALGVDIHEKDHPTILEMAKAAGLATGNVST
AELQDATPAALVAHVTSRKCYGPSATSEKCPGNALEKGGKGSITEQLLNARADVTLGGGAKTFAETATAGEWQGKTLREQAQ
ARGYQLVSDAASLNSVTEANQQKPLLGLFADGNMPVRWLGPKATYHGNIDKPAVTCTPNPQRNDSVPTLAQMTDKAIELLSKN
EKGFFLQVEGASIDKQDHAANPCGQIGETVDLDEAVQRALEFAKKEGNTLVIVTADHAHASQIVAPDTKAPGLTQALNTKDGAV
MVMSYGNSEEDSQEHTGSQLRIAAYGPHAANVVGLTDQTDLFYTMKAALGLK

Codon-optimized sequence (Start and stop codon, cut away by BsaI, appended sequence)

ctaaccggtctcctggtATGAAACAGAGCACCATTGCGCTGGCGCTGCTGCCGCTGCTGTTTACCCCGGTTACCAAAGCGCGCAC
CCCGGAAATGCCGGTGCTGGAAAACCGCGCGGCGCAGGGCGATATTACCGCGCCGGGCGGCGCCCGTCGCCTGACCG
GCGATCAGACCGCGGCCCTGCGCGACTCGCTGAGCGATAAACCGGCCAAAAATATCATTCTGCTGATTGGCGACGGCAT
GGGCGATAGCGAGATCACCGCAGCGCGCAATTATGCCGAAGGTGCGGGCGGCTTCTTTAAAGGCATTGATGCGCTGCCG
CTGACGGGCCAGTATACCCACTACGCCCTGAACAAAAAAACCGGCAAACCGGATTATGTTACCGATAGCGCGGCGAGCG
CCACCGCCTGGAGCACCGGCGTTAAAACCTATAACGGCGCCCTGGGTGTGGATATTCATGAAAAAGATCATCCGACGATT
CTGGAAATGGCCAAAGCGGCAGGCCTGGCCACCGGCAACGTGTCGACCGCGGAGCTGCAGGATGCCACCCCGGCGGC
GCTGGTGGCGCATGTGACCAGCCGCAAATGCTACGGTCCGAGCGCGACTAGCGAAAAATGTCCGGGTAACGCCCTGGA
AAAAGGTGGCAAAGGCAGCATTACCGAGCAGCTGCTGAACGCGCGCGCGGATGTGACCCTGGGCGGCGGTGCGAAAAC
CTTTGCGGAAACCGCCACCGCGGGCGAATGGCAGGGCAAAACGCTGCGCGAACAGGCACAGGCGCGCGGCTATCAGCT
GGTTAGCGATGCGGCGAGCCTGAACAGCGTGACGGAAGCCAACCAGCAGAAACCGCTGCTTGGTCTGTTTGCGGATGGT
AATATGCCGGTGCGTTGGCTGGGCCCGAAAGCGACCTACCACGGCAACATTGATAAACCCGCGGTGACCTGCACCCCGA
ACCCGCAGCGCAATGATAGCGTGCCGACCCTGGCCCAGATGACCGATAAAGCCATTGAACTGCTGAGCAAAAATGAAAAA
GGCTTTTTCCTGCAGGTGGAAGGTGCCAGCATTGATAAACAGGATCACGCCGCCAATCCGTGTGGTCAGATCGGTGAAAC
CGTTGATCTGGATGAAGCCGTGCAGCGCGCCCTGGAATTCGCGAAAAAAGAAGGCAATACGCTGGTGATTGTGACCGCG
GATCATGCCCATGCGAGCCAGATTGTGGCGCCGGATACGAAAGCACCGGGTCTGACCCAGGCGCTGAATACCAAAGATG
GCGCGGTGATGGTGATGAGCTATGGCAACAGCGAAGAAGATAGCCAGGAACACACCGGCTCTCAGCTGCGCATTGCGG
CGTATGGCCCGCATGCGGCGAATGTGGTGGGCCTGACCGATCAGACCGATCTGTTTTACACCATGAAAGCCGCCCTGGG
CCTGAAATAActtgggagaccaacta

Resulting tagged protein (His$_6$-tag, linker with thrombin cut site), 492 AA, 51.6 kDa, ε_{280} = 32.9 mM^{-1} cm^{-1}

MGSSHHHHHHGSGLVPRGSAGMKQSTIALALLPLLFTPVTKARTPEMPVLENRAAQGDITAPGGARRLTGDQTAALRDSLSDK
PAKNIILLIGDGMGDSEITAARNYAEGAGGFFKGIDALPLTGQYTHYALNKKTGKPDYVTDSAASATAWSTGVKTYNGALGVDIH
EKDHPTILEMAKAAGLATGNVSTAELQDATPAALVAHVTSRKCYGPSATSEKCPGNALEKGGKGSITEQLLNARADVTLGGGA
KTFAETATAGEWQGKTLREQAQARGYQLVSDAASLNSVTEANQQKPLLGLFADGNMPVRWLGPKATYHGNIDKPAVTCTPNP
QRNDSVPTLAQMTDKAIELLSKNEKGFFLQVEGASIDKQDHAANPCGQIGETVDLDEAVQRALEFAKKEGNTLVIVTADHAHAS
QIVAPDTKAPGLTQALNTKDGAVMVMSYGNSEEDSQEHTGSQLRIAAYGPHAANVVGLTDQTDLFYTMKAALGLK*

***Archaeoglobus fulgidus* geranylgeranylglyceryl phosphate synthase** (*Af*G_3PS)

PDB 2f6x, 2f6u, 8ruw, UniProt GGGPS_ARCFU, accession WP_010877910.1

MRWRKWRHITKLDPDRTNTDEIIKAVADSGTDAVMISGTQNVTYEKARTLIEKVSQYGLPIVVEPSDPSNVVYDVDYLFVPTVLN
SADGDWITGKHAQWVRMHYENLQKFTEIIESEFIQIEGYIVLNPDSAVARVTKALCNIDKELAASYALVGEKLFNLPIIYIEYSGTY
GNPELVAEVKKVLDKARLFYGGGIDSREKAREMLRYADTIIVGNVIYEKGIDAFLETLP

Codon-optimized sequence (Start and stop codon, cut away by BsaI, appended sequence)

ctaaccggtctcctggtATGCGCTGGCGCAAATGGCGTCATATTACCAAACTGGATCCGGACCGCACCAATACCGATGAAATTATT
AAAGCGGTGGCGGATAGCGGTACCGATGCCGTGATGATTAGTGGCACCCAGAATGTGACCTATGAAAAAGCGCGCACCC
TGATTGAAAAAGTGAGCCAGTATGGCCTGCCGATTGTTGTGGAACCGAGCGATCCGAGCAACGTGGTGTATGATGTGGAT
TACCTGTTCGTGCCGACCGTGCTGAACAGCGCGGATGGCGATTGGATTACCGGTAAACACGCGCAGTGGGTGCGTATGC
ACTACGAAAATCTGCAGAAATTTACCGAAATTATTGAAAGCGAATTTATTCAGATTGAAGGTTACATTGTGCTGAACCCGGA
TAGCGCCGTGGCGCGCGTGACCAAAGCCCTCTGCAATATTGACAAAGAACTGGCAGCGAGCTATGCGCTGGTGGGCGAA
AAACTGTTTAATCTGCCGATTATTTATATTGAATATAGCGGTACCTATGGTAATCCGGAACTGGTGGCGGAAGTTAAAAAAG
TGCTGGATAAAGCCCGCCTGTTTTACGGCGGCGGCATCGATAGCCGCGAAAAAGCGCGCGAAATGCTGCGTTATGCGGA
TACCATTATTGTGGGCAACGTGATCTATGAAAAAGGCATTGATGCCTTCCTGGAAACCCTGCCGTAActtgggagaccaacta

Resulting tagged protein (His_6-tag, linker with thrombin cut site), 252 AA, 28.3 kDa, ε_{280} = 41.4 mM^{-1} cm^{-1}

MGSSHHHHHHGSGLVPRGSAGMRWRKWRHITKLDPDRTNTDEIIKAVADSGTDAVMISGTQNVTYEKARTLIEKVSQYGLPIV
VEPSDPSNVVYDVDYLFVPTVLNSADGDWITGKHAQWVRMHYENLQKFTEIIESEFIQIEGYIVLNPDSAVARVTKALCNIDKELA
ASYALVGEKLFNLPIIYIEYSGTYGNPELVAEVKKVLDKARLFYGGGIDSREKAREMLRYADTIIVGNVIYEKGIDAFLETLP

***Methanosarcina barkeri 3* isopentenyl kinase T77A mutant** (*Mb*IPK T77A)

PDB - , UniProt A0A0E3WXG9_METBA, accession WP_048108133.1

MNVSTEPVILKLGGSVITDKAADQGVVREDSLRRIAKEVSEYRGKMIIVHGAGSFGHTYAKKYQLGKVFDPEGAIV**A**HESVKKLA
SRVVDTLNEYGVRAIAVHPMCCTICRNGRIESMYLDNIKLMLENGLVPVLHGDVVMDLELRACVLSGDQIVPYLAKELKITRLGL
GSAEDGVLDNDGKTVPEITPKTFEDFKHYIRGSGSTDVTGGMLGKVQELLELSKTSCITSYIFNAGKDDNIYRFLNGEPMGTTIS
PDKRV

Codon-optimized sequence (Start and stop codon, cut away by BsaI, appended sequence)

ctaaccggtctcctggtATGAACGTGAGCACGGAACCGGTTATTCTGAAACTGGGCGGTAGCGTGATTACCGATAAAGCCGCCGA
CCAGGGCGTGGTGCGCGAAGATAGCCTGCGTCGCATTGCGAAAGAAGTGAGCGAATATCGCGGCAAAATGATTATTGTT
CATGGTGCGGGCAGCTTTGGCCACACCTATGCGAAAAAATATCAGCTGGGCAAAGTGTTTGATCCGGAAGGCGCGATTGT
GGCGCACGAAAGCGTGAAAAAACTGGCGAGCCGCGTGGTGGATACCCTGAATGAATATGGTGTGCGCGCCATCGCGGT
GCATCCGATGTGTTGTACCATTTGCCGCAATGGCCGCATCGAAAGCATGTATCTGGATAACATTAAACTGATGCTGGAGAA
TGGCCTGGTGCCGGTGCTGCATGGCGATGTGGTGATGGATCTGGAACTGCGCGCATGCGTTCTGAGCGGCGATCAGATT
GTGCCGTACCTGGCCAAAGAACTGAAAATTACCCGCCTGGGCCTGGGCTCGGCGGAAGATGGCGTGCTGGATAACGATG
GCAAAACCGTCCCGGAAATTACCCCGAAAACCTTCGAAGATTTCAAACATTATATTCGTGGCAGCGGCAGCACCGATGTTA
CCGGCGGCATGCTGGGCAAAGTGCAGGAACTGCTGGAACTGAGCAAAACGAGCTGCATCACCAGCTACATTTTTAACGC
GGGCAAAGATGATAATATTTATCGTTTCCTGAACGGTGAACCGATGGGCACCACCATTAGCCCGGATAAACGCGTGTGAactt
gggagaccaacta

Resulting tagged protein (His$_6$-tag, linker with thrombin cut site), 281 AA, 30.5 kDa, ε_{280} = 13.4 mM^{-1} cm^{-1}

MGSSHHHHHHGSGLVPRGSAGMNVSTEPVILKLGGSVITDKAADQGVVREDSLRRIAKEVSEYRGKMIIVHGAGSFGHTYAKK
YQLGKVFDPEGAIV**A**HESVKKLASRVVDTLNEYGVRAIAVHPMCCTICRNGRIESMYLDNIKLMLENGLVPVLHGDVVMDLELR
ACVLSGDQIVPYLAKELKITRLGLGSAEDGVLDNDGKTVPEITPKTFEDFKHYIRGSGSTDVTGGMLGKVQELLELSKTSCITSYI
FNAGKDDNIYRFLNGEPMGTTISPDKRV

Geobacillus stearothermophilus **pyruvate kinase** (*Gs*PK)

PDB 2e28, UniProt KPYK_GEOSE, accession WP_033014443.1

MKRKTKIVCTIGPASESVDKLVQLMEAGMNVARLNFSHGDHEEHGRRIANIREAAKRTGRTVAILLDTKGPEIRTHNMENGAIEL
KEGSKLVISMSEVLGTPEKISVTYPSLIDDVSVGAKILLDDGLISLEVNAVDKQAGEIVTTVLNGGVLKNKKGVNVPGVKVNLPGIT
EKDRADILFGIRQGIDFIAASFVRRASDVLEIRELLEAHDALHIQIIAKIENEEGVANIDEILEAADGLMVARGDLGVEIPAEEVPLIQK
LLIKKCNMLGKPVITATQMLDSMQRNPRPTRAEASDVANAIFDGTDAVMLSGETAAGQYPVEAVKTMHQIALRTEQALEHRDIL
SQRTKESQTTITDAIGQSVAHTALNLDVAAIVTPTVSGKTPQMVAKYRPKAPIIAVTSNEAVSRRLALVWGVYTKEAPHVNTTDE
MLDVAVDAAVRSGLVKHGDLVVITAGVPVGETGSTNLMKVHVISDLLAKGQGIGRKSAFGKAVVAKTAEEARQKMVDGGILVTV
STDADMMPAIEKAAAIITEEGGLTSHAAVVGLSLGIPVIVGVENATTLFKDGQEITVDGGFGAVYRGHASVL

Codon-optimized sequence (Start and stop codon, cut away by BsaI, appended sequence)

ctaaccggtctcctggtATGAAACGCAAAACCAAAATTGTCTGCACCATTGGCCCGGCGAGCGAAAGCGTGGATAAACTGGTGCAA
CTGATGGAAGCGGGCATGAATGTGGCACGCCTGAACTTTTCCCATGGCGATCATGAAGAACATGGCCGCCGCATCGCGA
ACATTCGCGAAGCAGCGAAACGCACCGGCCGCACCGTTGCCATCCTGCTGGATACAAAAGGTCCGGAAATTCGCACCCA
TAACATGGAAAACGGCGCCATTGAACTGAAAGAAGGCAGCAAACTGGTGATTAGCATGAGCGAAGTGCTGGGAACCCCG
GAAAAAATTAGCGTGACGTATCCGAGCCTGATTGATGATGTGTCCGTGGGCGCGAAAATCCTGCTGGATGATGGCCTGAT
TAGCCTGGAAGTGAACGCGGTGGATAAACAGGCCGGCGAAATTGTGACCACCGTCCTGAACGGCGGCGTGCTGAAAAAT
AAAAAAGGCGTTAATGTGCCGGGCGTGAAAGTGAACCTGCCGGGCATTACCGAAAAAGATCGTGCGGATATTCTGTTCGG
CATTCGCCAGGGCATTGATTTTATTGCCGCCAGCTTCGTTCGCCGTGCGAGCGATGTTCTGGAAATCCGCGAACTGCTGG
AAGCGCACGATGCGCTGCACATCCAGATTATTGCGAAAATTGAAAACGAAGAAGGCGTGGCGAACATCGATGAAATCCTG
GAAGCGGCGGATGGCCTGATGGTTGCCCGCGGCGATCTGGGCGTGGAAATCCCGGCCGAAGAAGTGCCGCTGATCCAG
AAACTGCTGATTAAAAAATGCAACATGCTGGGTAAACCGGTTATTACCGCGACCCAGATGCTGGATAGCATGCAGCGCAA
CCCGCGCCCGACCCGCGCAGAGGCGAGCGATGTTGCCAATGCCATTTTTGATGGTACCGATGCCGTGATGCTGAGCGG
CGAAACCGCGGCCGGCCAGTACCCGGTGGAAGCGGTGAAAACCATGCATCAGATTGCGCTGCGCACCGAACAGGCGCT
GGAACACCGTGATATTCTGAGTCAGCGCACGAAAGAAAGCCAGACCACTATTACCGATGCCATTGGTCAAAGCGTTGCCC
ATACCGCCCTGAATCTGGATGTGGCAGCGATTGTAACCCCGACCGTTAGCGGCAAAACCCCGCAGATGGTGGCCAAATA
CCGTCCGAAAGCCCCGATTATTGCGGTGACCAGCAACGAAGCCGTGAGCCGTCGCCTGGCGCTGGTGTGGGGCGTCTA
TACCAAAGAAGCGCCGCATGTCAACACCACCGATGAAATGCTGGATGTGGCGGTGGATGCCGCGGTGCGCAGCGGCCT
GGTGAAACATGGCGATCTGGTGGTGATCACGGCCGGCGTGCCGGTGGGCGAAACCGGCAGCACGAACCTGATGAAAGT
GCACGTGATTAGCGATCTGCTGGCGAAAGGCCAGGGCATTGGCCGCAAATCCGCGTTTGGCAAAGCGGTGGTGGCGAA
AACGGCGGAAGAAGCACGCCAGAAAATGGTGGACGGCGGCATTCTGGTTACCGTGAGCACCGATGCGGATATGATGCC
GGCGATTGAAAAAGCGGCCGCGATTATTACGGAAGAAGGTGGTCTGACCAGCCACGCGGCGGTTGTGGGCCTGAGCCT
GGGCATTCCGGTGATTGTGGGCGTGGAAAACGCGACTACCCTGTTTAAGGACGGCCAGGAAATTACCGTTGATGGCGGC
TTTGGCGCGGTGTACCGCGGCCATGCGAGCGTGCTGTAActtgggagaccaacta

Resulting tagged protein (His_6-tag, linker with thrombin cut site), 608 AA, 64.4 kDa, ε_{280} = 13.0 mM^{-1} cm^{-1}

MGSSHHHHHHGSGLVPRGSAGMKRKTKIVCTIGPASESVDKLVQLMEAGMNVARLNFSHGDHEEHGRRIANIREAAKRTGRT
VAILLDTKGPEIRTHNMENGAIELKEGSKLVISMSEVLGTPEKISVTYPSLIDDVSVGAKILLDDGLISLEVNAVDKQAGEIVTTVLN
GGVLKNKKGVNVPGVKVNLPGITEKDRADILFGIRQGIDFIAASFVRRASDVLEIRELLEAHDALHIQIIAKIENEEGVANIDEILEAA
DGLMVARGDLGVEIPAEEVPLIQKLLIKKCNMLGKPVITATQMLDSMQRNPRPTRAEASDVANAIFDGTDAVMLSGETAAGQYP
VEAVKTMHQIALRTEQALEHRDILSQRTKESQTTITDAIGQSVAHTALNLDVAAIVTPTVSGKTPQMVAKYRPKAPIIAVTSNEAVS
RRLALVWGVYTKEAPHVNTTDEMLDVAVDAAVRSGLVKHGDLVVITAGVPVGETGSTNLMKVHVISDLLAKGQGIGRKSAFGK
AVVAKTAEEARQKMVDGGILVTVSTDADMMPAIEKAAAIITEEGGLTSHAAVVGLSLGIPVIVGVENATTLFKDGQEITVDGGFGA
VYRGHASVL

***Geobacillus stearothermophilus* lactate dehydrogenase** (*Gs*LDH)

PDB 1ldn, UniProt LDH_GEOSE, accession WP_033016716.1

MKNNGGARVVVIGAGFVGASYVFALMNQGIADEIVLIDANESKAIGDAMDFNHGKVFAPKPVDIWHGDYDDCRDADLVVICAGA
NQKPGETRLDLVDKNIAIFRSIVESVMASGFQGLFLVATNPVDILTYATWKFSGLPHERVIGSGTILDTARFRFLLGEYFSVAPQN
VHAYIIGEHGDTELPVWSQAYIGVMPIRKLVESKGEEAQKDLERIFVNVRDAAYQIIEKKGATYYGIAMGLARVTRAILHNENAILT
VSAYLDGLYGERDVYIGVPAVINRNGIREVIEIELNDDEKNRFHHSAATLKSVLARAFTR

Codon-optimized sequence (Start and stop codon, cut away by BsaI, appended sequence)

ctaaccggtctcctggtATGAAAAACAATGGCGGCGCGCGCGTGGTGGTGATTGGCGCGGGCTTCGTGGGCGCGAGCTACGTGT
TCGCGCTGATGAACCAGGGCATTGCGGATGAAATTGTGCTGATTGATGCGAATGAAAGCAAAGCCATTGGCGATGCGATG
GATTTTAATCACGGCAAGGTTTTTGCGCCGAAACCGGTTGATATTTGGCATGGCGATTATGATGATTGCCGTGATGCTGAT
CTGGTGGTGATTTGCGCGGGCGCGAACCAGAAACCGGGCGAAACCCGTCTGGATCTGGTGGATAAAAATATTGCGATCT
TTCGTAGCATTGTGGAAAGCGTTATGGCGAGCGGCTTTCAGGGCCTGTTCCTGGTCGCGACCAACCCGGTGGATATTCTG
ACCTATGCGACCTGGAAATTTAGCGGTCTGCCGCATGAACGCGTGATTGGCAGCGGTACCATTCTGGATACCGCCCGCTT
CCGCTTTCTGCTGGGCGAATATTTTAGCGTTGCGCCGCAGAACGTACATGCGTATATTATTGGTGAACACGGCGATACCG
AACTGCCGGTATGGTCACAGGCCTATATTGGCGTGATGCCGATTCGCAAACTGGTGGAATCTAAAGGCGAAGAAGCCCA
GAAAGATCTGGAACGTATTTTTGTGAACGTGCGCGATGCCGCGTACCAGATTATTGAAAAAAAAGGTGCGACCTACTATG
GCATTGCCATGGGCCTGGCGCGTGTGACCCGTGCCATTCTGCATAACGAAAATGCGATTTTAACCGTCAGCGCGTACCTG
GATGGCCTGTATGGCGAACGCGATGTGTACATTGGCGTGCCGGCGGTGATTAATCGTAACGGTATTCGTGAAGTGATCGA
AATCGAACTGAACGATGATGAAAAAAACCGTTTTCATCATAGCGCCGCCACCCTGAAAAGCGTGCTGGCGCGCGCGTTTA
CCCGCTAActtgggagaccaacta

Resulting tagged protein (His$_6$-tag, linker with thrombin cut site), 338 AA, 40.0 kDa, ε_{280} = 34.5 mM^{-1} cm^{-1}

MGSSHHHHHHGSGLVPRGSAGMKNNGGARVVVIGAGFVGASYVFALMNQGIADEIVLIDANESKAIGDAMDFNHGKVFAPKPV
DIWHGDYDDCRDADLVVICAGANQKPGETRLDLVDKNIAIFRSIVESVMASGFQGLFLVATNPVDILTYATWKFSGLPHERVIGS
GTILDTARFRFLLGEYFSVAPQNVHAYIIGEHGDTELPVWSQAYIGVMPIRKLVESKGEEAQKDLERIFVNVRDAAYQIIEKKGAT
YYGIAMGLARVTRAILHNENAILTVSAYLDGLYGERDVYIGVPAVINRNGIREVIEIELNDDEKNRFHHSAATLKSVLARAFTR

Routine protein production and purification

All enzymes were heterologously produced in *E. coli* as His_6-tagged proteins through IPTG-induced overexpression, in analogy to previously described procedures.[10] To this end, a 10 mL preculture of the expression strain (*E. coli* BL21(DE3) harboring the respective plasmid) was grown in LB medium (10 g L^{-1} tryptone, 5 g L^{-1} yeast extract, 10 g L^{-1} NaCl) supplemented with 34 mg L^{-1} chloramphenicol at 37 °C and 200 rpm for 6−7 h (until OD_{600} >0.5 was reached). This preculture was then diluted with 90 mL TB medium (12 g L^{-1} tryptone, 24 g L^{-1} yeast extract, 5 g L^{-1} glycerol, 2.31 g L^{-1} KH_2PO_4, 12.54 g L^{-1} K_2HPO_4) containing 34 mg L^{-1} chloramphenicol and 0.1 mM isopropyl β-D-1-thiogalactopyranoside (IPTG). This culture was incubated at 37 °C and 200 rpm overnight (ca. 18 h). Next, cells were harvested by centrifugation (4000 g, 20 min, 4 °C) and the resulting cell pellet was either stored at −20 °C until use or immediately subjected to lysis and purification.

Lysis and purification was performed through sonication, affinity chromatography and gel filtration. To this end, pelleted cells were resuspended in binding buffer (20 mM imidazole, 100 mM NaCl, 100 mM taurine, pH 9) to a concentration of around 0.5 g_{pellet} mL^{-1}. Next, Pierce™ protease inhibitor mini tables (Thermo Fisher Scientific) and DNAse were added. Cells were then disrupted by sonication (7 min, 10 s pulse, 10 s breaks, 70% amplitude). The resulting lysate was either directly centrifuged or, for *Gt*PyNP, *Gt*IPP and *Af*G_3PS heated for 20−30 min to precipitate *E. coli* proteins (50 °C for *Gt*IPP, 60 °C for *Gt*PyNP and 70 °C for *Af*G_3PS). Cell debris and precipitated protein was then removed by centrifugation (11000 rpm, 30 min, 4 °C) and filtration (0.45 µm pore size). The cell free extract was applied to a 2 mL Ni Sepharose Histrap™ FF column (GE Healthcare) preequilibrated with binding buffer. Non-specifically bound proteins were removed by washing with 5 column volumes (CV) of binding buffer and the target protein was eluted with elution buffer (500 mM imidazole, 100 mM NaCl, 100 mM taurine, pH 9). The first 0.7 mL elution volume were discarded, and the subsequent 2.5 mL of elution volume generally contained the entirety of the tagged protein, which was collected. Afterwards, the protein was desalted into 10 mM taurine buffer (pH 9) using a PD-10 desalting column (GE Healthcare). The resulting protein preparation was either diluted with glycerol to a final concentration of 50% (v/v) glycerol and stored at −20 °C or concentrated by centrifugation (Vivaspin, Sartorius, Göttingen, Germany, molecular weight cut-off at 10 kDa) before diluting with glycerol and storing at −20 °C. For the proteins reported herein, this generally yielded purities of >90% as assessed by SDS PAGE (Fig. S1). Typical protein yields are summarized in Table S1 below (calculated with 1 AU at 280 nm being equal to a protein concentration of 1 g L^{-1}).

*Ec*AP was produced as described above but not purified because the protein lost almost all its activity during the purification process. This enzyme was therefore used as a preparation in clarified lysate. To this end, the cell-free extract obtained after lysis, centrifugation and filtration was diluted with glycerol to a final concentration of 50% (v/v) and stored at −20 °C. These preparations retained activity for at least 6 months of storage at −20 °C.

Table S1. Typical protein yields from non-optimized production in shake flasks.[a]

Protein	Typical yield (mg L^{-1})	Protein	Typical yield (mg L^{-1})
*At*FPPS	300	*Sc*FPPS	290
*Af*GGPPS	200	*Sp*FPPS	200
*Ec*FPPS	15	*Tc*FPPS	40
*Hs*FPPS	80	*Gt*PyNP	10
*Gs*FPPS	50	*Gt*IPP	70
*Mt*polyPPPS	50	*Af*G_3PS	220
*Ns*FPPS	90	*Mb*IPK T77A	140
*Rc*FPPS	130	*Gs*PK	20
*Sf*FPPS	30	*Gs*LDH	75

[a] These yields represent purified protein and are the rounded average of two or three independent production runs, except for *Gt*IPP which was only produced once. For reasons unknown to us, the yield of *Gt*PyNP generally varied between 4 and 15 mg L^{-1} even under well-controlled conditions and after optimization.

The identity of all stable PEs as well as the previously characterized auxiliary enzymes (*Gt*PyNP, *Gt*IPP, *Gs*LDH, and *Af*G_3PS) was confirmed by UPLC-MS analysis. To this end, 3 µL of a ca. 2 µM protein solution was injected into an Acquity UPLC system (Waters) coupled to a quadrupole/time-of-flight (QToF) mass spectrometer (Waters) equipped with a PDA detector, employing a reversed phase ACQUITY UPLC BEH300 C4 1.7 µm column (2.1 mm×150 mm) running 0.1% formic acid in deionized water (A) and 0.1% formic acid in acetonitrile (B) at a flow rate of 0.3 mL min^{-1}. The elution program included an isocratic elution with 90% A and 10% B for 2 minutes, a linear gradient from 10 to 50% B (v/v) from 2−10 min, 50 to 95% B from 10−11 min, and an isocatic elution at 95% B from 11−13 min before returning to 5% B from 13−13.1 min and re-equilibrating to 5% B from 13.1−20 min. Mass spectra were obtained in the ESI-positive ion mode over a mass range between 500 to 2000 Da and deconvoluted with MagTran. Table S2 lists the expected and observed masses for the PEs, considering the loss of the *N*-terminal methionine and Fig. S8 shows *Gs*FPPS, *Mt*polyPPPS and *Sp*FPPS as illustrative examples. The full set of MS results is available from the externally hosted supplementary information at zenodo.org.[2] *Mb*IPK T77A and *Gs*PK fragmented during UPLC-MS analysis and only gave much lower observed molecular weights.

Table S2. Mass spectrometry results for the stable PEs and the auxiliary enzymes

PE	Expected molecular weight (Da)[a]	Observed molecular weight (Da)	Difference (calc−obs; Da)
*Af*GGPPS	37503.4	37501.5	1.9
*Gs*FPPS	34303.1	34301.2	1.9
*Hs*FPPS	41695.7	41693.2	2.5
*Mt*polyPPPS	37478.5	37476.8	1.7
*Nc*FPPS	42271.3	42270.4	0.9
*Rc*FPPS	31749.0	31748.5	0.5
*Sf*FPPS	42100.0	42098.0	2.0
*Sp*FPPS	41509.4	41508.3	1.1
*Gt*PyNP	48205.5	48205.9	0.4
*Gt*IPP	21118.8	21118.3	0.5
*Gs*LDH	36855.9	36855.0	0.9
AfG_3PS	28137.0	28137.4	0.4

[a] calculated with ProtParam (https://web.expasy.org/protparam/) for the sequences listed above, considering the loss of the *N*-terminal methionine, which occurred for all proteins in this work.

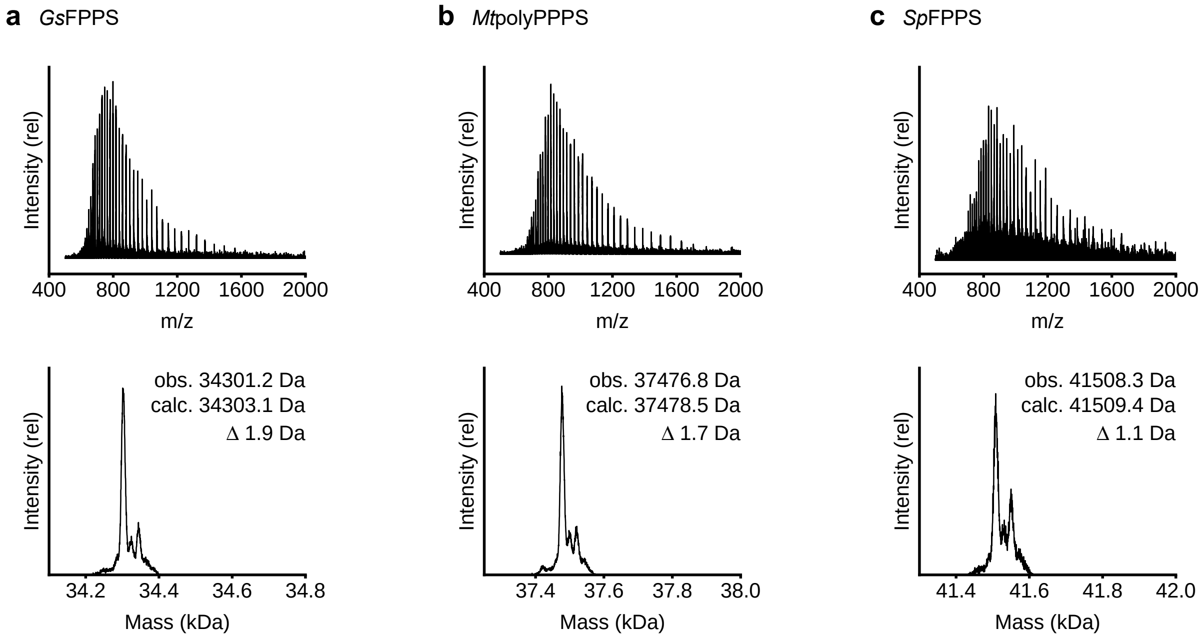

Figure S8. Illustrative MS data for PEs.

Characterization of the EPUB enzymes

As we cloned all genes for this study into a pACYC vector, the assay enzymes for the EPUB module (the inorganic pyrophosphatase *Gt*IPP and the pyrimidine nucleoside phosphorylase *Gt*PyNP, Fig. S9a) carried different linkers compared to our previous work. Therefore, we characterized these versions under conditions relevant to EPUB and found largely similar biochemical properties for both enzymes. The only notable difference was a slight substrate inhibition of *Gt*PyNP by phosphate (K_i > 5 mM), which does not represent any issue for the EPUB module as working phosphate concentrations stay well outside an inhibition regime. To the best of our knowledge, inhibition by phosphate has not been reported to date for pyrimidine nucleoside phosphorylases or uridine phosphorylases and we abstain from speculation as to how an *N*-terminal tag may influence the functional parameters of these enzymes.

To assess the stability of *Gt*PyNP, we measured its residual activity following incubation periods at incrementally higher temperatures. To this end, *Gt*PyNP was incubated at a concentration of 80 µg mL^{-1} in 10 mM taurine buffer pH 9 with 5% (v/v) glycerol in a total volume of 144 µL in a PCR tube. The tube was then placed in a PCR cycler and heated for 10 min each at 50, 55, 60, 63, 66, 69, 72, 75, 78, 80, 85, and 90 °C. After each incubation step, the tube was cooled to 20 °C and a 10 µL sample was withdrawn and pipetted into a well of a UV-transparent 96-well plate. This experiment was carried out in duplicate, yielding a total of 24 drops of 10 µL preincubated *Gt*PyNP in a multi-well plate. In sets of four, these enzyme samples were assayed for residual activity by adding 190 µL of reaction mixture to give final concentrations of 3 mM bromouridine, 1 mM phosphate, and 4 µg mL^{-1} *Gt*PyNP in 150 mM taurine buffer pH 9. The progress of these reactions at 25 °C was monitored at 315 nm for 1 min. The resulting absorption change over time was approximated by linear fitting (no forced intercept) and converted into observed rate constants with equation (S1).

$$k_{obs} = m/(\Delta\varepsilon\ d\ [E]) \qquad \text{(S1)}$$

where k_{obs} is the observed rate constant (s^{-1}), m is the linear slope of the absorption observed at a given wavelength (s^{-1}; in this case at 315 nm), $\Delta\varepsilon$ is the difference in extinction coefficient between bromouracil and bromouridine (3.1 mM^{-1} cm^{-1} at 315 nm and pH 9), d is the path length (generally 1 cm or corrected to 1 cm) and [E] is the enzyme concentration (µM; for instance, a 1 g L^{-1} working stock of *Gt*PyNP had a concentration of around 45.7 µM). This yielded residual rate constants as a function of preincubation temperature, which were fitted with the Boltzmann-type relationship (S2), yielding an apparent T_{50} value for this enzyme.

$$k_{obs}\ (T_{inc}) = k_{obs,0} + ((k_{obs,1}-k_{obs,0})/(1+\exp((T_{inc}-T_{50})/s))) \qquad \text{(S2)}$$

where $k_{obs,0}$ is the lower asymptote of the residual activity (set to 0 s^{-1}), $k_{obs,1}$ is the higher asymptote of the residual activity (s^{-1}), T_{inc} is the incubation temperature (°C or K), T_{50} is the temperature at which the residual activity is 0.5 $k_{obs,1}$ (C or K), and s is the steepness of the curve (dimensionless). This fit yielded $k_{obs,1}$ = 4.69 ± 0.11 s^{-1}, T_{50} = 73.5 ± 0.3 °C and s = 2.47 ± 0.06 (R^2 = 0.983) for $k_{obs,0}$ = 0 s^{-1} (Fig. S9b).

The stability of *Gt*IPP was assessed analogously. *Gt*IPP was incubated at a concentration of 3 µg mL^{-1} in the same buffer and setup and at the same temperatures. The reaction mixtures had final concentrations of 3 mM bromouridine, 0.5 mM pyrophosphate, 2 mM $MgCl_2$, 20 µg mL^{-1} *Gt*PyNP and 0.15 µg mL^{-1} *Gt*IPP in 150 mM taurine buffer pH 9 in a total volume of 200 µL. The progress of these reactions at 25 °C was monitored at 315 nm for 1 min. The resulting absorption change over time was approximated by linear fitting (no forced intercept) and converted into observed rate constants with equation (S3).

$$k_{obs} = m/(2\ \Delta\varepsilon\ d\ [E]) \qquad \text{(S3)}$$

with definitions from above. Equation (S3) only differs from (S1) by the factor of two in the denominator which accounts for the production of two equivalents of orthophosphate per pyrophosphate consumed. The resulting rate constant therefore reports on substrate consumption (and not product formation). Fitting of the resulting rates as a function of the preincubation temperature with equation (S2) then yielded $k_{obs,1}$ = 28.85 ± 0.63 s^{-1}, T_{50} = 72.0 ± 0.2 °C and s = 1.55 ± 0.02 (R^2 = 0.984) for $k_{obs,0}$ = 0 s^{-1} (Fig. S9e).

To assess the affinity of *Gt*PyNP and *Gt*IPP for their phosphate substrates under EPUB conditions, we obtained Michaelis-Menten kinetics for each enzyme. To examine *Gt*PyNP's affinity for bromouridine, we performed reactions with 0.05−15 mM bromouridine, 1 mM phosphate, and 2.5 µg mL^{-1} *Gt*PyNP in 1x EPUB buffer (150 mM taurine, 3 mM bromouridine, 2 mM $MgCl_2$, 3 mM (2-hydroxypropyl)-β-cyclodextrin, 1 mM TBA hydroxide, pH 9) with 4% (v/v) glycerol and 1% (v/v) Tween20 in a total volume of 200 µL. These reactions were prepared as master mixes and started by addition of the reaction mixtures (140 µL) to a suitably diluted bromouridine solution (40 µL). The progress of these reactions at 25 °C was monitored at 315 nm for 1 min. The resulting data were treated with equation (S1), which yielded observed rate constants as a function of the phosphate concentration. These data were fitted to the Michaelis-Menten equation (S4).

$$k_{obs,max} = (k_{obs}[S]) / (K_M+[S]) \quad (S4)$$

where $k_{obs,max}$ is the maximum observed rate constant (equivalent to k_{cat}; s^{-1}), k_{obs} is the rate constant (s^{-1}) observed at the substrate concentration [S] (arbitrary molar concentration) and K_M is the Michaelis-Menten constant (the same arbitrary molar concentration). This fit yielded $k_{obs,max}$ = 7.71 ± 0.15 s^{-1} and K_M = 294 ± 21 µM (R^2 = 0.983,Fig. S9c).

To examine *Gt*PyNP's affinity for phosphate, we performed reactions with 0.05−5.7 mM bromouridine, 3 mM bromouridine, and 2.5 µg mL^{-1} *Gt*PyNP in 1x EPUB buffer (150 mM taurine, 3 mM bromouridine, 2 mM $MgCl_2$, 3 mM (2-hydroxypropyl)-β-cyclodextrin, 1 mM TBA hydroxide, pH 9) with 4% (v/v) glycerol and 1% (v/v) Tween20 in a total volume of 200 µL. These reactions were prepared as master mixes and started by addition of the reaction mixtures (154.4) to a suitably diluted phosphate solution (45.6 µL). The progress of these reactions at 25 °C was monitored at 315 nm for 1 min. The resulting data were treated with equation (S1) and fitted to the modified Michaelis-Menten equation (S5) which takes substrate inhibition into account (Fig. S9d).

$$k_{obs,max} = (k_{obs}[S]) / (K_M+[S]+([S]^2/K_i)) \quad (S5)$$

where K_i is inhibition constant (arbitrary molar concentrations) and definitions from above apply. This fit yielded $k_{obs,max}$ = 13.29 ± 0.44 s^{-1}, K_M = 119 ± 11 µM, and K_M = 5.48 ± 0.70 mM (R^2 = 0.971).

To examine *Gt*IPP's affinity for phosphate, we performed reactions with 10−400 µM pyrophosphate, 0.15 µg mL^{-1} *Gt*IPP and 20 µg mL^{-1} *Gt*PyNP in 1x EPUB buffer with 4% (v/v) glycerol and 1% (v/v) Tween20 in a total volume of 200 µL. These reactions were prepared as master mixes and started by addition of the reaction mixtures (160 µL) to suitably diluted pyrophosphate (40 µL). The progress of these reactions at 25 °C was monitored at 315 nm for 1 min. The resulting data were treated with equations (S3) and (S4), which yielded $k_{obs,max}$ = 42.71 ± 0.68 s^{-1} and K_M = 16 ± 1 µM (R^2 = 0.964, Fig. S9f).

To examine *Gt*IPP's affinity for magnesium, we performed reactions with 0.1−10 mM $MgCl_2$, 500 µM pyrophosphate, 0.15 µg mL^{-1} *Gt*IPP and 20 µg mL^{-1} *Gt*PyNP in 1x EPUB buffer (without $MgCl_2$) with 4% (v/v) glycerol and 1% (v/v) Tween20 in a total volume of 200 µL. These reactions were prepared as master mixes and started by addition of the reaction mixtures (160 µL) to suitably diluted $MgCl_2$ (40 µL). The progress of these reactions at 25 °C was monitored at 315 nm for 1 min. The resulting data were treated with equation (S3) and relationship (S6) to describe the threshold concentration of necessary Mg^{2+}.

$$k_{obs}(Mg^{2+}) = k_{obs,a} + ((k_{obs,b}-k_{obs,a})/(1+\exp((\log[Mg^{2+}]-\log c_{50})/s))) \quad (S6)$$

where $k_{obs,a}$ is the lower asymptote of the observed activity (set to 0 s^{-1}), $k_{obs,b}$ is the higher asymptote of the observed activity (s^{-1}), [Mg^{2+}] is the magnesium concentration (arbitrary molar concentrations), c_{50} is the magnesium concentration at which the residual activity is 0.5 $k_{obs,b}$ (arbitrary molar concentrations), and definitions from above apply. This fit yielded $k_{obs,b}$ = 38.12 ± 0.37 s^{-1}, c_{50} = 1.0 ± 1.0 mM and s = 0.13 ± 0.01 (R^2 = 0.997) for $k_{obs,0}$ = 0 s^{-1} (Fig. S9g).

These kinetic parameters for *Gt*PyNP and *Gt*IPP compare well to those previously reported for differently tagged version of these enzymes[10] and the K_m values are significantly lower than those reported previously in glycine buffer,[11] as discussed in our prior work.[10] Collectively, the substrate affinities of *Gt*PyNP and *Gt*IPP indicate that poor affinity should not represent a kinetic bottleneck with organophosphate concentrations in the low to moderate µM range as these two enzymes should enable an efficient flow of intermediates down the reaction cascade to generate a UV signal from released pyrophosphate.

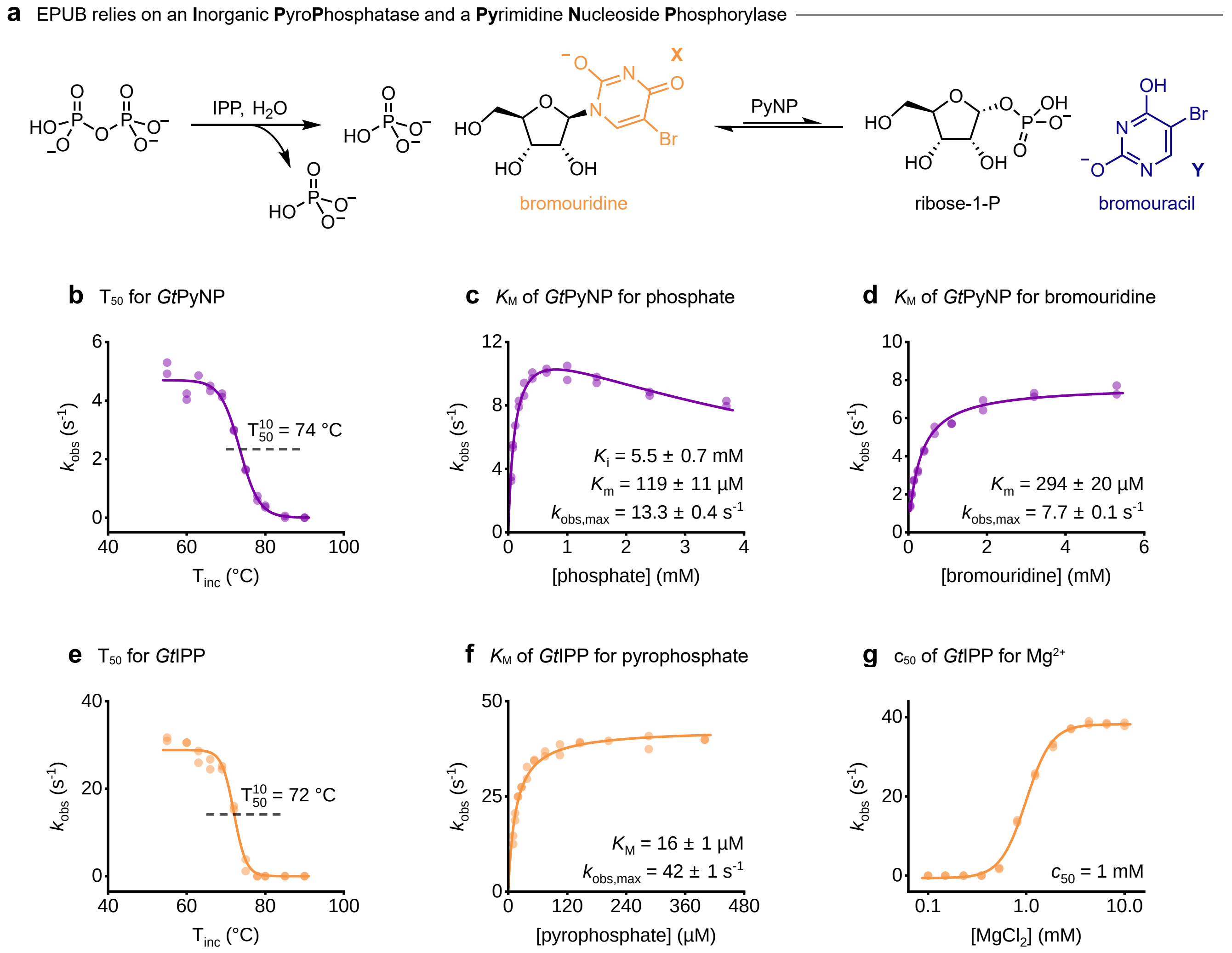

Figure S9. Characterization of *Gt*PyNP and *Gt*IPP. The differences in the maximum rate constants are due to differences in reaction conditions between the stability (**b**, **e**) and Michaelis-Menten experiments (**c**, **d**, **f**, **g**; see above for exact experimental conditions).

To ensure reproducibility of different batches of EPUB enzymes, we assayed different batches of *Gt*PyNP under standardized conditions and normalized for batch activity. To this end, we performed reactions with 100 µM phosphate and 1−4 µg mL^{-1} *Gt*PyNP in 1x EPUB buffer with 4% (v/v) glycerol and 1% (v/v) Tween20 in a total volume of 200 µL. These reactions were prepared as master mixes and started by addition of the reaction mixtures (180 µL) to suitably diluted *Gt*PyNP (20 µL). The progress of these reactions at 25 °C was monitored at 315 nm for 1 min. The resulting data were treated with equation (S7), which returns batch activities in U mg^{-1}.

$$Y = m/(\Delta\varepsilon \, d \, [E]_g) \qquad \text{(S7)}$$

where $[E]_g$ is the enzyme concentration in g L^{-1} and definitions from above apply. Linear fitting of the slopes of these reactions (only considering the first 3 min of the experiments with 1 µg mL^{-1} *Gt*PyNP) yielded batch activities of 12−14 U mg^{-1}, where one unit (U) is defined as the amount of enzyme which effects the conversion of 1 µmol of substrate per minute under EPUB conditions at 25 °C.

Characterization of the ARK enzymes

To interrogate the substrate scope and kinetics of *Mb*IPK T77A, we employed an assay system detecting ADP production through Rephosphorylation and Ketoacid reduction (ARK), using a pyruvate kinase (PK) and a lactate dehydrogenase (LDH, Fig. S10a). This coupled assay translates phosphorylation activity into a UV signal following oxidation of NADH to NAD^+ and has previously been used in variations by other groups for the interrogation of IPKs.[12–14] We opted to use the homologues from *Geobacillus stearothermophilus* because both *Gs*PK[15] and *Gs*LDH[16] have been described previously, including crystal structures. In particular, the thorough characterization of *Gs*PK by Imahori and colleagues[17] indicated that, despite its size, this enzyme should be tractable and active after production in *E. coli*. We could produce and purify both enzymes with N-terminal His_6-tags following heterologous production in *E. coli* and characterized them under conditions resembling EPUB conditions. As we were primarily interested in the performance of *Mb*IPK T77A in EPUB buffer, we carried out all biochemical characterization of this enzyme – as well as the ARK module enzymes – in taurine buffer at pH 9.

Prior to the characterization of the ARK module enzymes, we determined the extinction coefficient of NADH at 360 nm in taurine buffer at pH 9 and optimized the reaction conditions. To this end, we prepared solutions of 10−240 µM NADH in 50 mM taurine buffer at pH 9 in total volumes of 200 µL and measured their extinction at 360 nm using a platereader. The resulting absorption values were described by linear fitting (no forced intercept) where the slope reports on the extinction coefficient. This yielded ε_{360} = 4.21 mM^{-1} cm^{-1} (R^2 = 0.999, Fig. S10b). In our early characterization of *Gs*PK, we could corroborate many of the observation of Imahori and colleagues[17] about *Gs*PK's need for K^+ and the suppression of its cooperativity by adenosine monophosphate (AMP). Through a series of optimization experiments, we arrived at optimized reaction conditions which resembled EPUB conditions but featured several important differences. For instance, we found that 50 mM KCl were needed for *Gs*PK to function well (Fig. S10c, performed with 1−50 mM KCl, 0.5 mM PEP, 0.2 mM NADH, 0.2 mM ADP, 0.5 mM AMP, 10 mM $MgCl_2$, 30 µg mL^{-1} *Gs*PK, 100 µg mL^{-1} *Gs*LDH in 50 mM taurine buffer pH 9 with 4% (v/v) glycerol at 25 °C in a total volume of 200 µL, fitted with equations (S1) and (S4)) and 0.2 mM AMP effectively suppressed its cooperativity and facilitated performance at low substrate concentrations. As such, the rates of IPKs obtained with the ARK module are not directly comparable to those obtained with EPUB. In addition, as the data below illustrate, *Gs*PK's and *Gs*LDH's high K_M values for their substrates at pH 9 effected a significant lag time (Figs S4c, d and g and S5). Nonetheless, the slope after the lag time was linear over the concentration of the rate-limiting enzyme, so that this coupled assay system outputs meaningful data even though it underestimates true rate constants.

To assess the affinity of *Gs*PK and *Gs*LDH for their phosphate substrates under ARK conditions, we obtained Michaelis-Menten kinetics for each enzyme. To examine *Gs*LDH's affinity for pyruvate, we performed reactions with 0.05−1 mM pyruvate, 0.2 mM NADH, 0.2 mM ADP, 0.5 mM AMP, 10 mM $MgCl_2$, 50 mM KCl, and 15 µg mL^{-1} *Gs*LDH in 50 mM taurine buffer pH 9 with 4% (v/v) glycerol in a total volume of 200 µL. These reactions were prepared as master mixes and started by addition of the reaction mixtures (194 µL) to suitably diluted *Gs*LDH (4 µL). The progress of these reactions at 25 °C was monitored at 360 nm for 10 min. The resulting data were treated with equations (S1) and (S4), which yielded $k_{obs,max}$ > 40 min^{-1} and K_M > 5 mM as the experimental data did not approach the saturation regime (Fig. S10e).

To examine *Gs*LDH's affinity for NADH, we performed reactions with 15−240 mM NADH, 0.5 mM pyruvate, 0.2 mM ADP, 0.5 mM AMP, 10 mM $MgCl_2$, 50 mM KCl, and 15 µg mL^{-1} *Gs*LDH in 50 mM taurine buffer pH 9 with 4% (v/v) glycerol in a total volume of 200 µL. These reactions were prepared as master mixes and started by addition of the reaction mixtures (194 µL) to suitably diluted *Gs*LDH (4 µL). The progress of these reactions at 25 °C was monitored at 360 nm for 10 min. The resulting data were treated with equations (S1) and (S4), which yielded $k_{obs,max}$ = 5.07 ± 0.47 min^{-1} and K_M = 70 ± 18 µM (R^2 = 0.902, Fig. S10f).

To examine *Gs*PK's affinity for PEP, we performed reactions with 0.05−1 mM PEP, 0.2 mM NADH, 0.2 mM ADP, 0 or 0.2 mM AMP, 10 mM $MgCl_2$, 50 mM KCl, and 100 µg mL^{-1} *Gs*LDH, and 25 µg mL^{-1} *Gs*PK in 50 mM taurine buffer pH 9 with 4% (v/v) glycerol in a total volume of 200 µL. These reactions were prepared as master mixes and started by addition of the reaction mixtures (180 µL) to suitably diluted *Gs*PK (20 µL). The progress of these reactions at 25 °C was monitored at 360 nm for 30 min. The resulting data for 0.2 mM AMP were treated with equations (S1) and (S4) while excluding the initial equilibration period of 8 min, which yielded $k_{obs,max}$ = 2.89 ± 0.15 min^{-1} and K_M = 594 ± 61 µM (R^2 = 0.993, Fig. S10h). The

resulting data for 0 mM AMP were treated with equation (S1) and the Hill equation (S8) while excluding the initial equilibration period of 8 min.

$$k_{obs} = (k_{obs,max}\,[S]^n)/(K_A{}^n + [S]^n) \qquad \text{(S8)}$$

where K_A is the ligand (or substrate) concentration producing half occupation, n is the Hill coefficient, describing cooperativity, $K_A{}^n$ is equivalent to the apparent dissociation constant K_d and definitions from above apply. This fit yielded $k_{obs,max}$ = 2.30 ± 0.38 min^{-1}, K_A = 737 ± 200 µM, and n = 1.27 ± 0.13 (R^2 = 0.994, Fig. S10h).

To examine *Gs*PK's affinity for ADP, we performed reactions with 30−750 µM ADP, 0.5 mM PEP, 0.2 mM AMP, 0.2 mM NADH, 10 mM $MgCl_2$, 50 mM KCl, 100 µg mL^{-1} *Gs*LDH, and 25 µg mL^{-1} *Gs*PK in 50 mM taurine buffer pH 9 with 4% (v/v) glycerol in a total volume of 200 µL. These reactions were prepared as master mixes and started by addition of the reaction mixtures (180 µL) to suitably diluted *Gs*PK (20 µL). The progress of these reactions at 25 °C was monitored at 360 nm for 30 min. The resulting data were treated with equations (S1) and (S4) while excluding the initial equilibration period of 8 min, which yielded $k_{obs,max}$ = 2.82 ± 0.09 min^{-1} and K_M = 239 ± 20 µM (R^2 = 0.992, Fig. S10i).

To ensure reproducibility of different batches of ARK enzymes, we assayed different batches of *Gs*PK under standardized conditions and normalized for batch activity. To this end, we performed reactions with 0.4 mM ADP, 0.8 mM PEP, 0.2 mM AMP, 0.2 mM NADH, 10 mM $MgCl_2$, 50 mM KCl, 100 µg mL^{-1} *Gs*LDH, and 5−20 µg mL^{-1} *Gs*PK in 50 mM taurine buffer pH 9 with 4% (v/v) glycerol in a total volume of 200 µL. These reactions were prepared as master mixes and started by addition of the reaction mixtures (180 µL) to suitably diluted *Gs*PK (20 µL). The progress of these reactions at 25 °C was monitored at 315 nm for 1 min. The resulting data were treated with equation (S7). Linear fitting of the slopes of these reactions (excluding the first 8 min of the equilibration period) yielded batch activities of 0.14−0.19 U mg^{-1}, where one unit (U) is defined as the amount of enzyme which effects the conversion of 1 µmol of substrate per minute under ARK conditions at 25 °C.

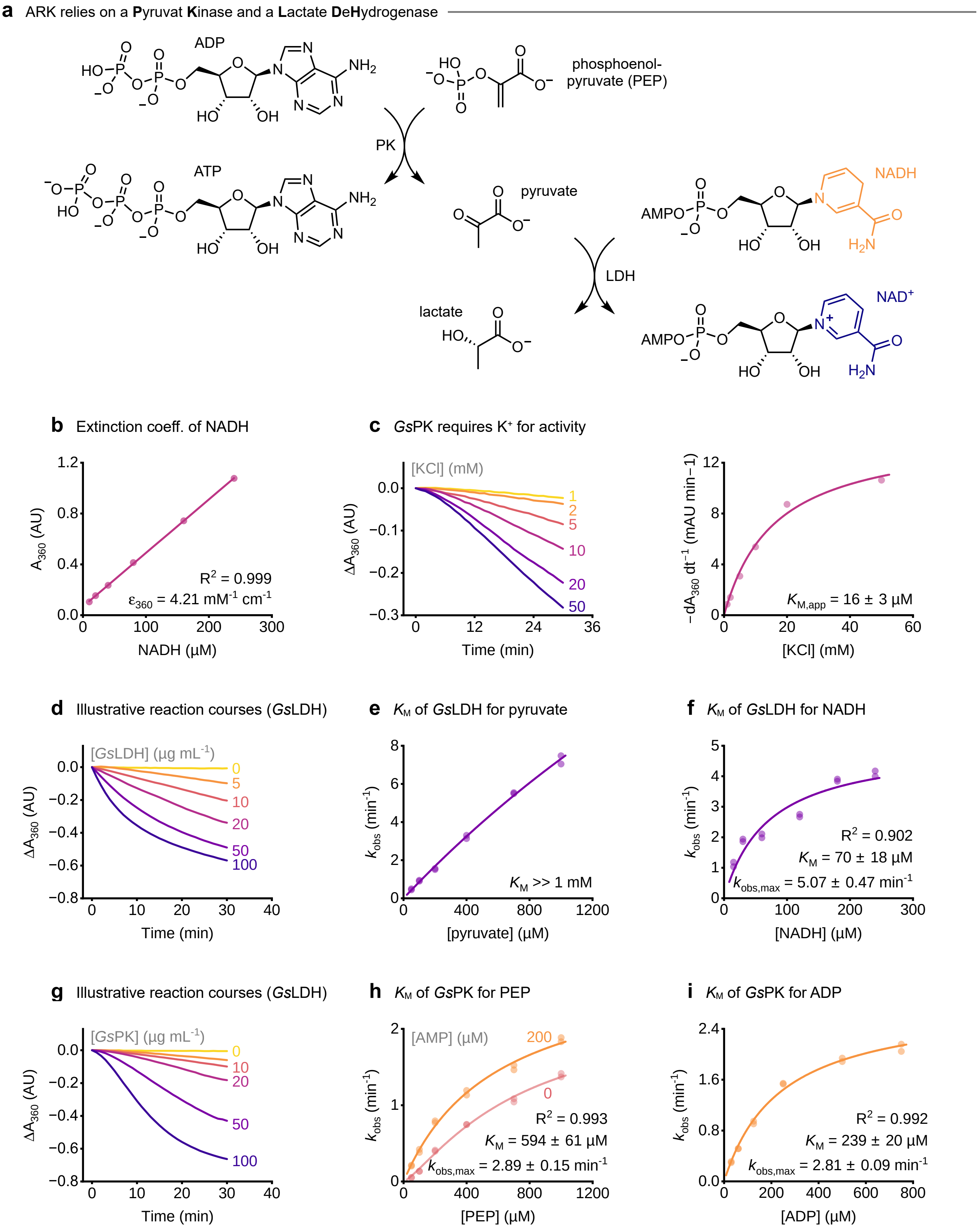

Figure S10. Characterization of *Gs*LDH and *Gs*PK as assay enzymes of the ARK module for kinase interrogation.

Characterization of *Mb*IPK T77A

We used the ARK module to interrogate the substrate scope of *Mb*IPK T77A by detecting ADP formation through rephosphorylation with PEP and reduction of pyruvate to lactate (Fig. S11a). Even under optimized conditions this version of ARK gave measurable background signal, e.g. in the absence of IPK or monophosphate substrate (Fig. S11b), likely through non-productive hydrolysis of ATP by the pyruvate kinase. These experiments were performed with 800 µM PEP, 0.2 mM NADH, 0.4 mM ATP, 0.2 mM AMP, 10 mM $MgCl_2$, 50 mM KCl, 100 µg mL^{-1} *Gs*LDH, 50 µg mL^{-1} *Gs*PK, and 5 µg mL^{-1} *Mb*IPK T77A in 50 mM taurine buffer pH 9 with 4% (v/v) glycerol in a total volume of 200 µL (or without either of these reagents). These reactions were prepared as master mixes and started by addition of the reaction mixtures (180 µL) to suitably diluted *Mb*IPK T77A (20 µL). The progress of these reactions at 25 °C was monitored at 360 nm for 55 min. However, this baseline noise proved reproducible and could easily be corrected for by subtracting the signal from negative control from each sample, converting raw (Fig. S11c) into corrected reaction courses (Fig. S11d). These experiments were performed with 800 µM PEP, 0.3 mM **2a-P**, 0.2 mM NADH, 0.4 mM ATP, 0.2 mM AMP, 10 mM $MgCl_2$, 50 mM KCl, 100 µg mL^{-1} *Gs*LDH, 50 µg mL^{-1} *Gs*PK, and 0−5 µg mL^{-1} *Mb*IPK T77A in 50 mM taurine buffer pH 9 with 4% (v/v) glycerol. These reactions were prepared as master mixes and started by addition of the reaction mixtures (180 µL) to suitably diluted *Mb*IPK T77A (20 µL). The progress of these reactions at 25 °C was monitored at 360 nm for 55 min. Despite the significant lag (or equilibration) time these reaction exhibited due to the poor affinity of *Gs*PK for ADP (Fig. S10i) and *Gs*LDH for pyruvate (Fig. S10e), this assay did output meaningful data. Fitting the pseudo-linear part of the reaction course right after the initial lag time (Fig. S11e) gave slope whose values were directly proportional to the concentration of the active enzyme (Fig. S11f). Hence, although ARK underestimates the true rate of *Mb*IPK T77A under these conditions, it does report on approximate rate constants and substrate affinity.

To examine the substrate scope of *Mb*IPK T77A, we screened the enzyme with a panel of (iso-)prenyl phosphate analogues. To this end, we performed reactions with 0.8 mM PEP, 0.2 mM NADH, 0.4 mM ATP, 0.2 mM AMP, 10 mM $MgCl_2$, 50 mM KCl, 100 µg mL^{-1} *Gs*LDH, 50 µg mL^{-1} *Gs*PK, and 0, 2, 20 or 200 µg mL^{-1} *Mb*IPK T77A in 50 mM taurine buffer pH 9 with 4% (v/v) glycerol in a total volume of 200 µL. These reactions were prepared as master mixes and started by addition of the reaction mixtures (160 µL) to suitably diluted *Mb*IPK T77A (40 µL). The progress of these reactions at 25 °C was monitored at 360 nm for 30 min. This experiment revealed that *Mb*IPK T77A converted the prenyl phosphates **1a-P**, **1b-P**, **1c-P**, **1d-P** and **1e-P** as well as the corresponding analogues of the **2x-P** series with measurable rates (Fig. S12). For most analogues, the rate with the **1x-P** and the corresponding **2x-P** analogue was essentially identical. To further examine the performance of *Mb*IPK T77A with these analogues, we assessed the affinity of the enzyme for these substrates. We first obtained a kinetic profile for the phosphate donor ATP. To this end, we performed reactions with 0.5 mM **1a-P**, 0.8 mM PEP, 0.2 mM NADH, 20−800 µM ATP, 0.2 mM AMP, 10 mM $MgCl_2$, 50 mM KCl, 100 µg mL^{-1} *Gs*LDH, 50 µg mL^{-1} *Gs*PK, and 2 µg mL^{-1} *Mb*IPK T77A in 50 mM taurine buffer pH 9 with 4% (v/v) glycerol in a total volume of 200 µL. These reactions were prepared as master mixes and started by addition of the reaction mixtures (180 µL) to suitably diluted *Mb*IPK T77A (20 µL). The progress of these reactions at 25 °C was monitored at 360 nm for 30 min. The resulting data (excluding the first 10 min of equilibration time) were treated with equations (S1) and (S4), which yielded $k_{obs,max}$ = 103 ± 5 min^{-1} and K_M = 296 ± 32 µM (R^2 = 0.989, data not shown).

Next, we examined the affinity of *Mb*IPK T77A for the (iso-)prenyl phosphate analogues. To this end, we performed reactions with 0.2−1.5 mM (iso-prenyl) phosphate, 0.8 mM PEP, 0.2 mM NADH, 0.4 mM ATP, 0.2 mM AMP, 10 mM $MgCl_2$, 50 mM KCl, 100 µg mL^{-1} *Gs*LDH, 50 µg mL^{-1} *Gs*PK, and 1−80 µg mL^{-1} *Mb*IPK T77A in 50 mM taurine buffer pH 9 with 4% (v/v) glycerol in a total volume of 200 µL. It should be noted that these conditions do not achieve [ATP] saturation ([ATP] ≈ 1.3 K_M) as high ATP concentrations produce high background signals due to unspecific hydrolysis and introduce phosphate impurities typically contained in commercial samples of ATP. As such, the following $k_{obs,max}$ further underestimate "true" *Mb*IPK T77A performance. These reactions were prepared as master mixes and started by addition of the reaction mixtures (180 µL) to suitably diluted *Mb*IPK T77A (20 µL). The progress of these reactions at 25 °C was monitored at 360 nm for 30 min. The resulting data (excluding the first 10−12 min of equilibration time) were treated with equations (S1) and (S4). For **1a-P** with 1 µg mL^{-1} *Mb*IPK T77A, this yielded $k_{obs,max}$ = 46.4 ± 1.2 min^{-1} and K_M = 102 ± 15 µM (R^2 = 0.989, Fig. S12c).

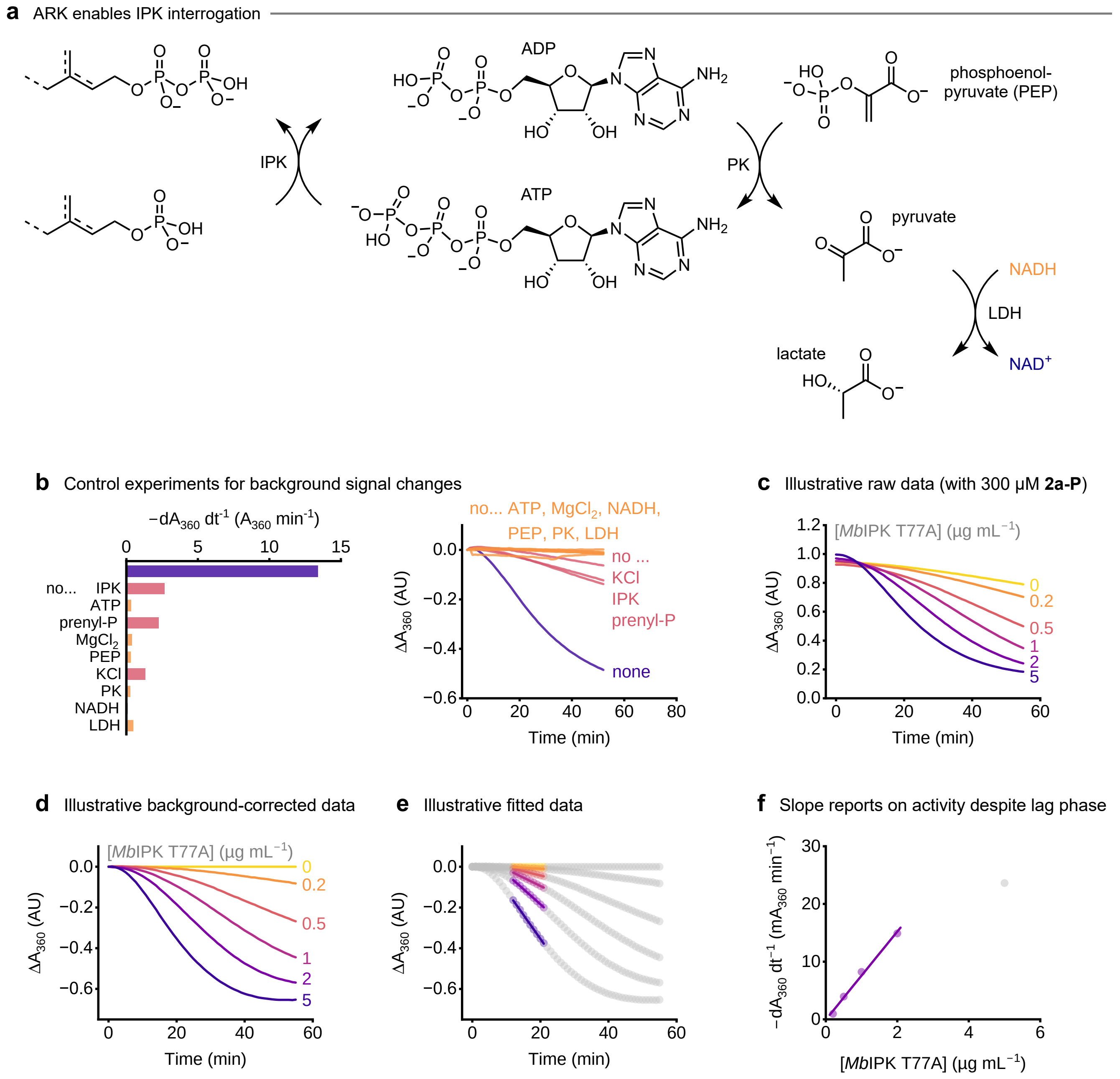

Figure S11. Control experiments for the characterization of *Mb*IPK T77A with the ARK module.

For **1b-P** with 1 μg mL^{-1} *Mb*IPK T77A, this yielded $k_{obs,max}$ = 117.6 ± 2.0 min^{-1} and K_M = 325 ± 16 μM (R^2 = 0.992, Fig. S12f). For **1c-P** with 8 μg mL^{-1} *Mb*IPK T77A, this yielded $k_{obs,max}$ = 14.1 ± 1.8 min^{-1} and K_M = 1176 ± 271 μM (R^2 = 0.964, Fig. S12i). For **1d-P** with 2.5 μg mL^{-1} *Mb*IPK T77A, this yielded $k_{obs,max}$ = 19.3 ± 1.2 min^{-1} and K_M = 345 ± 59 μM (R^2 = 0.913, Fig. S12l). For **1e-P** with 80 μg mL^{-1} *Mb*IPK T77A, this yielded $k_{obs,max}$ = 0.8 ± 0.1 min^{-1} and K_M = 503 ± 81 μM (R^2 = 0.942, Fig. S12o). However, considering that this assay does not capture initial rates perfectly and we could not saturate [ATP], these kinetic parameters need to be regarded as appoximations, as indicated in Fig. 3 in the main text.

To ensure reproducibility of different batches of *Mb*IPK T77A, we assayed different batches under standardized conditions. To this end, we performed reactions with 150 μM **1a-P**, 150 μM **2a-P**, 500 μM ATP, 80 μg mL^{-1} *Rc*FPPS and 5−20 μg mL^{-1} *Mb*IPK T77A in 1x EPUB buffer with 1x EPUB enzymes in a total volume of 200 μL. The progress of these reactions at 25 °C was monitored at 315 nm for 1 min. The resulting data were treated with equation (S7), yielding batch activities of 5−8 U mg^{-1}.

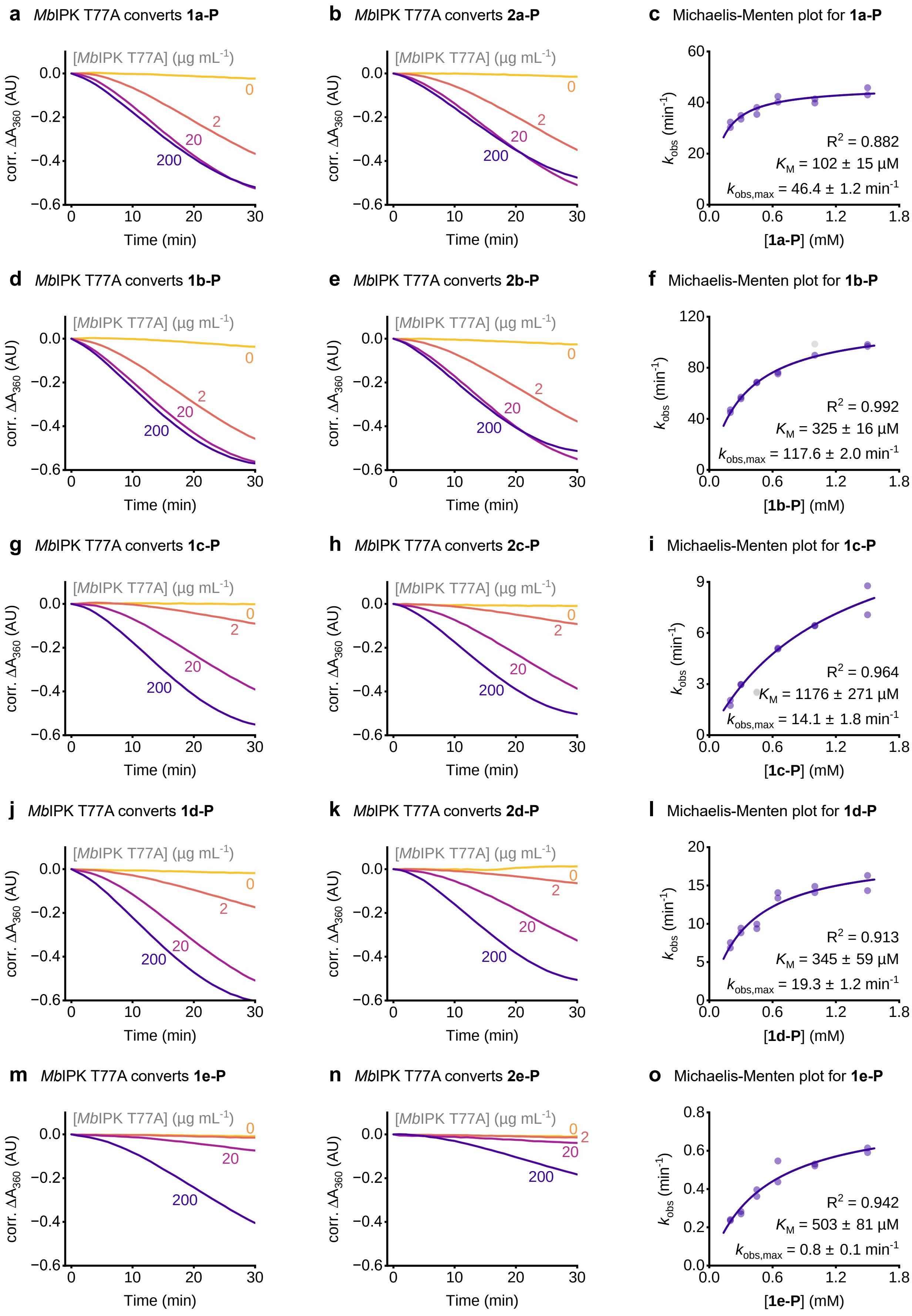

Figure S12. Activity of *Mb*IPK T77A with modified (iso-)prenyl phosphates.

Next, we obtained orthogonal data to ARK to verify that *Mb*IPK T77A selectively produces the desired organopyrohosphates. To this end, we performed i) a complementation assay with an FPPS and EPUB which reports on the presence of prenyl pyrophosphates by performing a chain extension and detecting the released inorganic pyrophosphate and ii) ^{31}P NMR experiments to interrogate the selectivity of *Mb*IPK T77A.

To assay *Mb*IPK T77A with EPUB, we performed reactions with 150 µM **1a-P**, 150 µM **2a-PP**, 200 µM ATP, 80 µg mL^{-1} *Rc*FPPS, and 0−8 µg mL^{-1} *Mb*IPK T77A in 1x EPUB buffer (150 mM taurine, 3 mM bromouridine, 2 mM $MgCl_2$, 3 mM (2-hydroxypropyl)-β-cyclodextrin, 1 mM TBA hydroxide, pH 9) with 1x EPUB enzymes (20 µg mL^{-1} GtPyNP, 0.5 µg mL^{-1} GtIPP, 4% (v/v) glycerol, 1% (v/v) Tween20) in a total volume of 200 µL. These reactions were prepared as master mixes and started by addition of the reaction mixtures (168 µL) to suitably diluted *Mb*IPK T77A (32 µL). The progress of these reactions at 25 °C was monitored at 315 nm for 30 min. These reactions probed the **1a-P→1a-PP** phosphorylation by supplying the missing substrate for an FPPS (Fig. S7a). The analogous series of reactions was also performed with **1a-PP** and **2a-P**. These reactions yielded a clear dose-response relationship (Fig. S7b and c), providing confirmation of *Mb*IPK T77A activity with an assay orthogonal to ARK.

To interrogate the selectivity of *Mb*IPK T77A by ^{31}P NMR, we performed reactions with 1 mM organophosphate, 2 mM PEP, 0.1 mM ATP, 5 mM $MgCl_2$, 50 mM KCl, 100 µg mL^{-1} GsPK, and 0−200 µg mL^{-1} *Mb*IPK T77A in 50 mM taurine buffer pH 9 with 10% D_2O and 4% (v/v) glycerol in a total volume of 650 µL in 1.5 mL plastic tubes. These mixtures were incubated at 20 °C for 18 h, filled into NMR tubes and analyzed by ^{31}P NMR (243 MHz, 128 scans). In these reactions, we employed catalytic ATP/ADP and a PEP-based recycling system as the signals for the α- and β-phosphate of ADP and (iso-)prenyl pyrophosphates overlap. This series of experiment revealed that *Mb*IPK T77A phosphorylates all tested substrate analogues with excellent selectivity. All impurities (orthophosphate from ATP, the phosphodiester from the synthesis of the monophosphates, and an unidentified impurity from PEP) remained untouched, all monophosphate was consumed, and only the desired pyrophosphate was produced (Figs. S8 and S9).

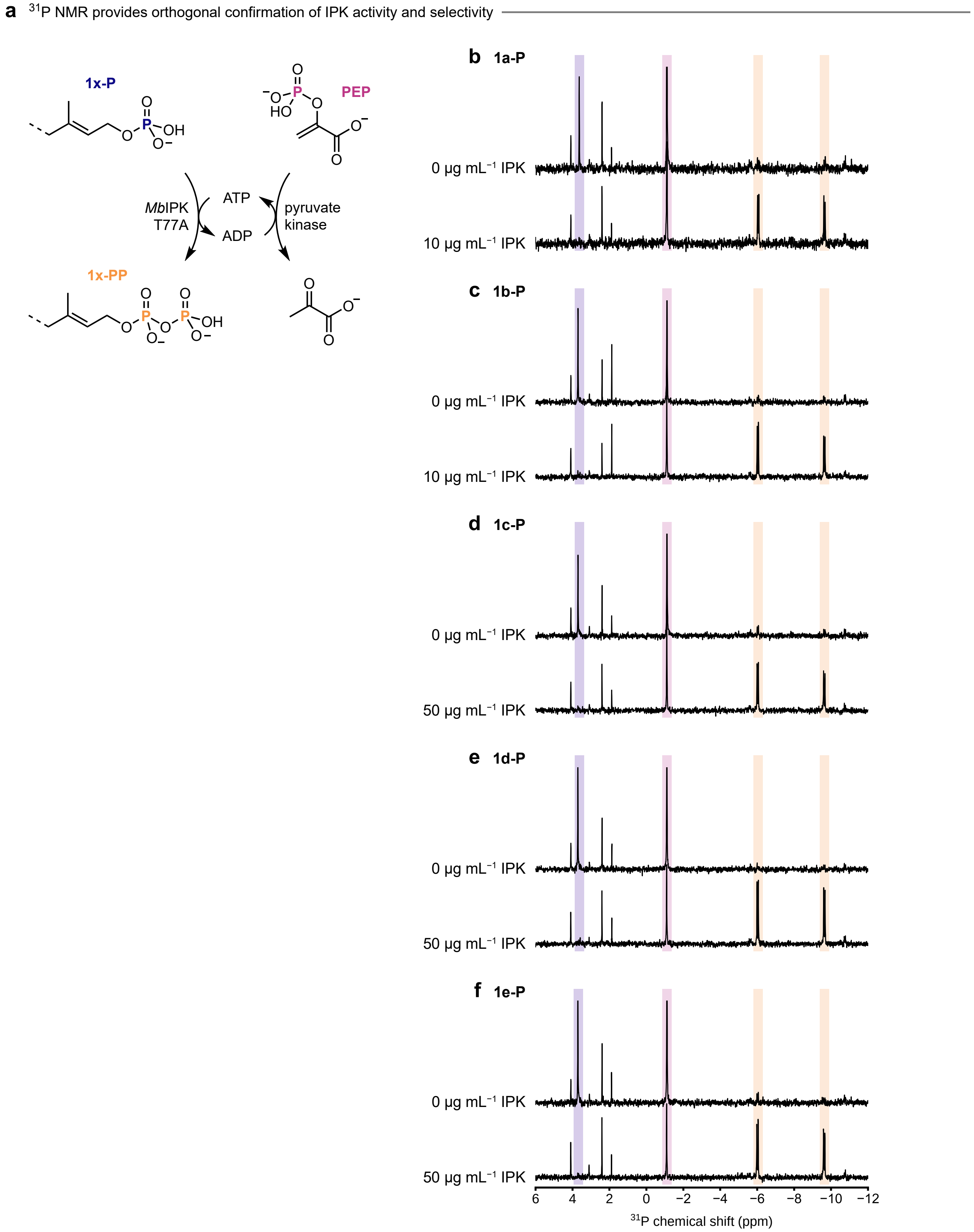

Figure S13. Confirmation of *Mb*IPK T77A's activity with prenyl pyrophosphates and selectivity by ^{31}P NMR. Note that the desired pyrophosphate is already present in the synthetic material of the monophosphate due to it being a byproduct of the condensation. The peak(s) around 3.5−4 ppm in the kinase-containing mixture do not correspond to remaining monophosphate but to non-converted impurities in the preparation of the monophosphate.

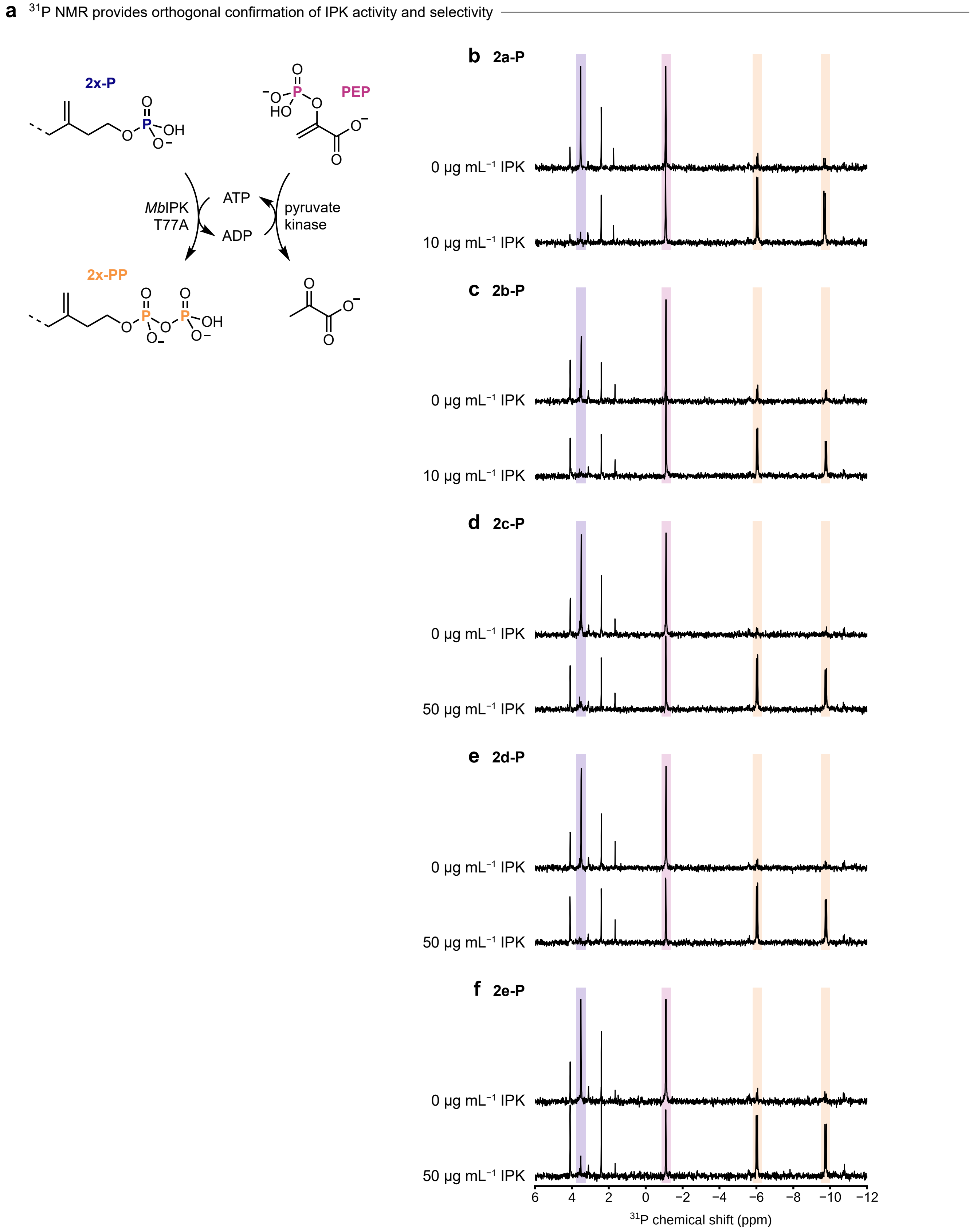

Figure S14. Confirmation of *Mb*IPK T77A's activity with isoprenyl pyrophosphates and selectivity by ^{31}P NMR. Note that the desired pyrophosphate is already present in the synthetic material of the monophosphate due to it being a byproduct of the condensation. The peak(s) around 3.5−4 ppm in the kinase-containing mixture do not correspond to remaining monophosphate but to non-converted impurities in the preparation of the monophosphate.

Equilibrium states of (iso-)prenyl mono-to-pyrophosphorylation

To assess if an ATP recycling system would be necessary when implementing IPK-mediated pyrophosphorylation into EPUB, we examined the equilibrium states of the prenyl mono-to-pyrophosphate transformation. To this end, we performed reactions with 2 mM **1a-P**, 0.4−4 mM ATP, 8 mM $MgCl_2$, and 30 µg mL^{-1} *Mb*IPK T77A in 50 mM taurine buffer pH 9 with 10% D_2O. These mixtures were incubated at 20 °C for 3 h, filled into NMR tubes and analyzed by ^{31}P NMR (243 MHz, 512 scans). Each mixture was re-analyzed after 18 h of incubation to confirm that the equilibrium had been reached. In the resulting data (Fig. S6a and b), the initial **1a-P** and ATP concentrations were calculated from peak areas (obtained via graphical fitting) and mass balances and the conversion of **1a-P** to **1a-PP** was expressed as a function of the applied ATP equivalents. The resulting conversion data as a function of excess reagent was then fitted with equation (S9), which yields an equilibrium constant.[18]

$$c = (-K-Kx+\mathrm{sqrt}((K+Kx)^2+4Kx(1-K)))/(2-2K) \qquad \text{(S9)}$$

where c is the dimensionless conversion of the limiting starting material (in fractions, not percent), K is the equilibrium constant, and x is the excess of the reagent whose concentration was varied. Fitting of the experimental data that showed $c > 0.5$ yielded $K = 16.8 \pm 3.3$ (Fig. S6c). Careful fitting of all relevant peaks with Lorentzian functions and application of (S9) gave $K = 12.3 \pm 2.5$.

Repeating the analogous experiment with **2a-P** and **1b-P** gave similar results. Repeating the experiment with **2a-P** and 30 µg mL^{-1} *Mb*IPK T77A, graphical fitting and data treatment with equation (S9) yielded $K = 7.5 \pm 1.7$ (Fig. S6d). Repeating the experiment with **1d-P** and 100 µg mL^{-1} *Mb*IPK T77A, graphical fitting and data treatment with equation (S9) yielded $K = 13.0 \pm 7.1$ (Fig. S6e). Thus, we concluded that (iso-)prenyl pyrophosphorylation is sufficiently exergonic to enable quasi-quantitative phosphorylation with a slight excess of ATP so that a recycling system would not be needed.

Characterization of PEs

Kinetic experiments with EPUB and initial activity tests

We routine employed EPUB for kinetic experiments and PE characterization. The EPUB mastermixes were prepared as described previously.[10] Briefly, To prepare 10 mL of 2x EPUB buffer (300 mM taurine, 6 mM 5-bromouridine, 4 mM $MgCl_2$, 2 mM TBA hydroxide, 6 mM (2-hydroxypropyl)-β-cyclodextrin, pH 9), 19.4 mg 5-bromouridine and 87.6 mg (2-hydroxypropyl)-β-cyclodextrin were dissolved in 5 mL 0.6 M taurine buffer (pH 9) before adding 20 µL 2 M $MgCl_2$ and 12.5 µL 1.6 M TBA hydroxide (equivalent to 40% TBA hydroxide in water) and filling up to 10 mL with deionized water. To prepare 1 mL of 10x EPUB enzymes (200 µg mL^{-1} *Gt*PyNP, and 5 µg mL^{-1} *Gt*IPP, 40% v/v glycerol, 10% v/v Tween20, 10 mM taurine, pH 9), stock solutions of *Gt*PyNP and *Gt*IPP (both in 10 mM taurine buffer with 50% v/v glycerol, pH 9) were combined to achieve final concentrations of 200 and 5 µg mL^{-1}, respectively, before adding 200 µL 50% (v/v) Tween20 and 16 µL 0.6 M taurine buffer (pH 9). Finally, the glycerol content was adjusted to 40% (v/v) (considering the glycerol added via the stock solutions of the enzymes) with 60% (v/v) glycerol (preferred) or pure glycerol (if necessary) and the volume was adjusted to 1 mL with deionized water.

Note:

"1x EPUB buffer" is equivalent to "150 mM taurine, 3 mM bromouridine, 2 mM $MgCl_2$, 3 mM (2-hydroxypropyl)-β-cyclodextrin, 1 mM TBA hydroxide, pH 9".

"1x EPUB enzymes" is equivalent to "20 µg mL^{-1} *Gt*PyNP, and 0.5 µg mL^{-1} *Gt*IPP, 4% (v/v) glycerol, 1% (v/v) Tween20, 10 mM taurine, pH 9".

PEs were generally assayed in microwell plates using a platereader (as described in the general remarks) by following the change in absorption at 315 nm. The absorption change over time was approximated by linear fitting (no forced intercept) and converted into observed rate constants with equation (S3). Under EPUB conditions, EPUB's linear range (where the observed slopes are directly proportional to the underlying activity) stretches to approximately 25 mAU min^{-1} (see [10]).

$$k_{obs} = m/(2\ \Delta\varepsilon\ d\ [E]) \qquad (S3)$$

where definitions from above apply.

Following their production and purification, we performed an initial activity test of our PEs using EPUB and the native substrates **1a-PP** and **2a-PP** (as their TBA-salts). To this end, we performed reactions with 200 µM **1a-PP**, 400 µM **2a-PP**, and 2, 10 or 50 µg mL^{-1} PE in 1x EPUB buffer with 1x EPUB enzymes in a total volume of 200 µL. The resulting reaction courses showed a clear dose-response relationship for all PEs, with the reactions with the more active PEs reaching full conversion in the 30 min experiment (Fig. S2).

Although *At*FPPS, *Ec*FPPS, *Sc*FPPS and *Tc*FPPS were active, they quickly lost activity in the taurine buffers of our assay systems. For instance, *At*FPPS was complete inactivated following a 2 h incubation in 10 mM taurine buffer at room temperature (data not shown).

Thermal stability of PEs

To assess the stability of the PEs, we measured their residual activity following incubation periods at incrementally higher temperatures. To this end, the enzymes were incubated in 10 mM taurine buffer pH 9 with 10% (v/v) glycerol in a total volume of 180 µL in a PCR tube. The tube was then placed in a PCR cycler and heated for 5 min each at different temperatures. After each incubation step, the tube was cooled to 20 °C and a 20 µL sample was withdrawn and pipetted into a well of a UV-transparent 96-well plate which was stored at 4 °C until the end of the incubation times. This experiment was carried out in duplicate, yielding a total of 16 drops of 20 µL preincubated PE in a multi-well plate. In sets of 24, these enzyme samples were assayed for residual activity by adding 180 µL of reaction mixture to give final concentrations of 120 µM **1a-PP**, 240 µM **2a-PP**, and 20 µL preincubated PE in 1x EPUB buffer with 1x EPUB enzymes in a total volume of 200 µL. The progress of these reactions at 25 °C was monitored at 315 nm for 15 min. The resulting absorption change over time was approximated by linear fitting (no forced intercept) over the initial pseudo-linear regime and converted into observed rate constants with equation (S3). This yielded residual rate constants as a function of preincubation temperature, which were fitted with the Boltzmann-type relationship (S2), yielding an apparent T_{50} value for each PE. The incubation temperature and fitted parameters for all stable PEs are listed in Table S3, with the exception of those for *Af*GGPPS which did not deactivate after incubation at 90 °C, and Fig. S15 shows the inactivation profiles.

Table S3. Thermal stability of PEs.

PE	Incubation temp. (°C)	Final [PE] in assay (µg mL^{-1})	$k_{obs,0}$ (min^{-1})	$k_{obs,1}$ (min^{-1})	T_{50} (°C)	s	R^2
*Af*GGPPS	40, 50, 60, 70, 80, 90	10	*still active after incubation at 90 °C*				
*Gs*FPPS	45, 50, 55, 60, 65, 70, 75, 80	7	0.04 ± 0.08	3.26 ± 0.06	66.7 ± 0.3	2.3 ± 0.1	0.990
*Hs*FPPS	20, 25, 30, 35, 40, 45, 50, 55	8	0.60 ± 0.15	8.45 ± 0.14	39.0 ± 0.3	1.7 ± 0.1	0.992
*Mt*polyPPPS	30, 35, 40, 45, 50, 55, 60, 65	80	0.1 ± 0	4.34 ± 0.06	70.4 ± 0.3	3.2 ± 0.3	0.990
*Nc*FPPS	25, 30, 35, 40, 45, 50, 55, 60	1.5	3.55 ± 0.84	62.02 ± 1.80	33.1 ± 0.3	2.5 ± 0.2	0.995
*Rc*FPPS	30, 35, 40, 45, 50, 55, 60, 65	1	0	44.03 ± 1.0	61.3 ± 0.4	5.6 ± 0.3	0.995
*Sf*FPPS	25, 30, 35, 40, 45, 50, 55, 60	20	0.53 ± 0.06	2.85 ± 0.04	46.5 ± 0.4	1.2 ± 0.3	0.987
*Sp*FPPS	20, 25, 30, 35, 40, 45, 50, 55	4	1.19 ± 0.12	40.70 ± 0.12	40.0 ± 0.1	2.9 ± 0.1	0.999

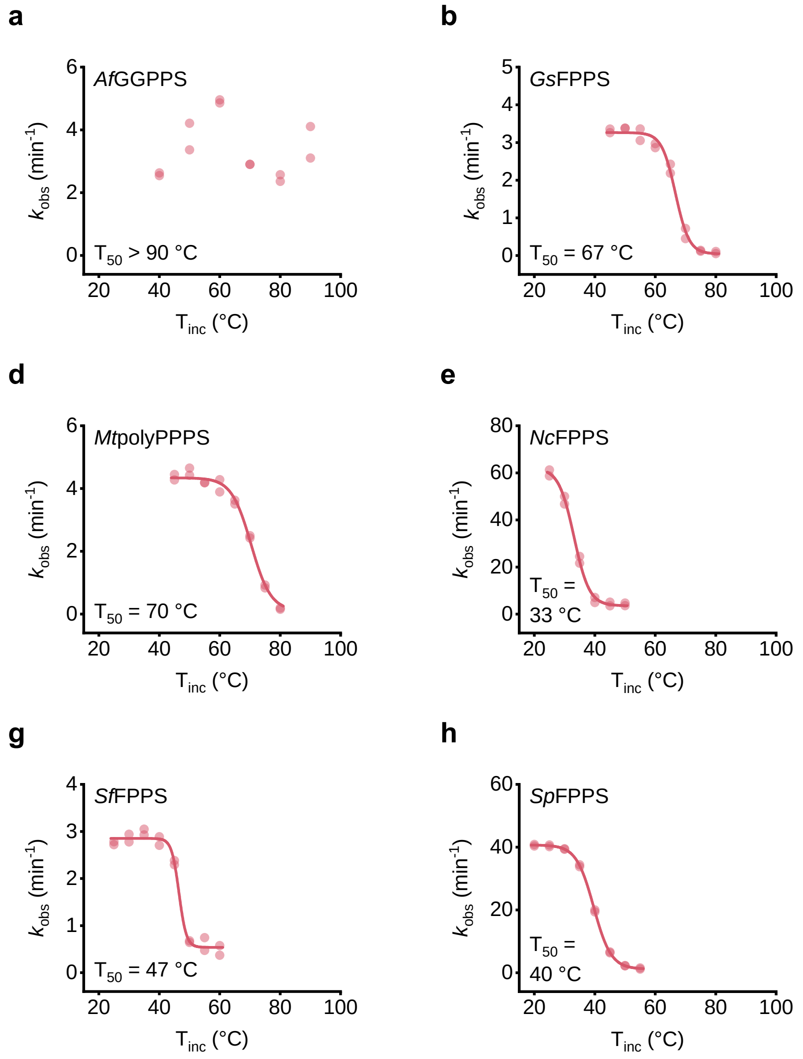

Figure S15. Thermal inactivation profiles of the PEs.

Michaelis-Menten kinetics with PEs and their native substrates

To examine the kinetic characteristics of the PEs with their native substrates, we obtained Michaelis-Menten-type kinetic profiles for each PE for the first (**1a-PP** + **2a-PP**), the second (**3aa-PP** + **2a-PP**), and, if applicable, the third chain extension (**4aaa-PP** + **2a-PP**), using EPUB. To this end, we performed reactions with 120 µM excess substrate and 5−150 µM limiting substrate in 1x EPUB buffer with 1x EPUB enzymes in a total volume of 200 µL. The progress of these reactions at 25 °C was monitored at 315 nm for 15 min. The resulting absorption change over time was approximated by linear fitting (no forced intercept) over the initial pseudo-linear regime and converted into observed rate constants with equation (S3). The resulting rate constants as a function of initial substrate concentration were fitted with either the Michaelis-Menten equation (S4) or equation (S5) if inhibition was observed. Table S4 lists enzyme concentrations and fitted kinetic parameters, while Fig. S3 illustrates representative kinetic profiles and kinetic traces. The full dataset is available from zenodo.org.[2]

Table S4. Kinetic parameters of PEs with their native substrates.

PE	[PE] in assay (µg mL^{-1})		Equation used	$k_{obs,max}$ (min^{-1})	K_M (µM)	K_i (µM)	R^2
*First chain extension (**1a-PP** + **2a-PP**)*							
*Af*GGPPS	6	for varied [**1a-PP**]	(S4)	11.51 ± 1.62	112 ± 30	-	0.964
		for varied [**2a-PP**]	(S5)	27.79 ± 3.98	16 ± 4	38 ± 8	0.961
*Gs*FPPS	3	for varied [**1a-PP**]	(S4)	8.65 ± 0.41	40 ± 5	-	0.979
		for varied [**2a-PP**]	(S5)	25.06 ± 1.32	12 ± 1	51 ± 4	0.990
*Hs*FPPS	5	for varied [**1a-PP**]	(S4)	16.33 ± 0.54	27 ± 3	-	0.984
		for varied [**2a-PP**]	(S5)	24.39 ± 1.31	7 ± 1	188 ± 29	0.951
*Mt*polyPPPS	4	for varied [**1a-PP**]	(S4)	6.10 ± 0.15	28 ± 2	-	0.991
		for varied [**2a-PP**]	(S5)	9.30 ± 0.97	24 ± 4	577 ± 312	0.983
*Nc*FPPS	1	for varied [**1a-PP**]	(S4)	145.07 ± 3.15	17 ± 1	-	0.988
		for varied [**2a-PP**]	(S4)	116.59 ± 2.58	12 ± 1	-	0.979
*Rc*FPPS	0.5	for varied [**1a-PP**]	(S4)	51.72 ± 2.87	16 ± 3	-	0.933
		for varied [**2a-PP**]	(S4)	54.15 ± 2.41	9 ± 2	-	0.898
*Sf*FPPS	10	for varied [**1a-PP**]	(S4)	6.20 ± 0.12	11 ± 1	-	0.983
		for varied [**2a-PP**]	(S5)	9.50 ± 0.53	6 ± 1	250 ± 48	0.921
*Sp*FPPS	1.5	for varied [**1a-PP**]	(S4)	74.67 ± 1.08	14 ± 1	-	0.993
		for varied [**2a-PP**]	(S5)	79.59 ± 4.79	10 ± 1	924 ± 468	0.976
*Second chain extension (**3aa-PP** + **2a-PP**)*							
*Af*GGPPS	6	for varied [**3aa-PP**]	(S4)	5.73 ± 0.23	20 ± 3	-	0.964
		for varied [**2a-PP**]	(S5)	9.73 ± 0.64	9 ± 1	174 ± 31	0.949
*Gs*FPPS	3	for varied [**3aa-PP**]	(S4)	5.89 ± 0.13	18 ± 1	-	0.988
		for varied [**2a-PP**]	(S5)	10.68 ± 0.61	6 ± 1	137 ± 20	0.937
*Hs*FPPS	5	for varied [**3aa-PP**]	(S4)	7.83 ± 0.34	11 ± 2	-	0.922
		for varied [**2a-PP**]	(S5)	8.45 ± 0.67	14 ± 2	1249 ± 1045	0.976
*Mt*polyPPPS	4	for varied [**3aa-PP**]	(S5)	4.99 ± 0.28	6 ± 1	577 ± 285	0.936
		for varied [**2a-PP**]	(S4)	5.73 ± 0.29	16 ± 3	-	0.918
*Nc*FPPS	1	for varied [**3aa-PP**]	(S5)	108.50 ± 7.02	22 ± 3	349 ± 82	0.992
		for varied [**2a-PP**]	(S4)	79.25 ± 2.18	10 ± 1	-	0.963
*Rc*FPPS	0.5	for varied [**3aa-PP**]	(S5)	48.60 ± 3.50	18 ± 3	732 ± 341	0.987
		for varied [**2a-PP**]	(S4)	40.09 ± 1.05	9 ± 1	-	0.963
*Sf*FPPS	10	for varied [**3aa-PP**]	(S5)	14.58 ± 2.08	31 ± 7	186 ± 63	0.969
		for varied [**2a-PP**]	(S5)	9.23 ± 0.58	4 ± 1	752 ± 357	0.872
*Sp*FPPS	1.5	for varied [**3aa-PP**]	(S5)	50.10 ± 1.54	18 ± 1	515 ± 78	0.987
		for varied [**2a-PP**]	(S4)	40.34 ± 0.76	6 ± 1	-	0.968
*Third chain extension (**4aaa-PP** + **2a-PP**)*							
*Af*GGPPS	18	for varied [**4aaa-PP**]	(S4)	2.91 ± 0.23	53 ± 10	-	0.961
		for varied [**2a-PP**]	(S5)	6.26 ± 0.69	15 ± 3	65 ± 12	0.956
*Mt*polyPPPS	18	for varied [**4aaa-PP**]	(S5)	1.61 ± 0.14	19 ± 3	287 ± 82	0.979
		for varied [**2a-PP**]	(S4)	1.44 ± 0.10	44 ± 8	-	0.965

Temperature-dependence of rates of PE-mediated chain extensions

As our PE panel featured meso- as well as thermophilic enzymes (which may have different temperature preferences), enzymatic rates generally follow Eyring-type or MMRT-type[10,19–22] relationships, we examined the rates of PE-catalyzed chain extensions as a function of the reaction temperature. To this end, we performed reactions with 120 µM **1a-PP** and 120 µM **2a-PP** in 1x EPUB buffer with 1x EPUB enzymes in a total volume of 200 µL. These reactions were prepared as master mixes, preheated to the reaction temperature for 5 min, and started by addition of the reaction mixtures (180 µL) to suitably diluted PE (20 µL). This analysis was performed for *Af*GGPPS, *Gs*FPPS, *Mt*polyPPPS, *Rc*FPPS, *Sf*FPPS and *Sp*FPPS, while *Hs*FPPS (T_{50} = 39 °C) *Nc*FPPS (T_{50} = 33 °C) were too thermolabile. The progress of these reactions at either 25, 28, 31 or 34 °C was monitored at 315 nm for 10 min. The resulting absorption change over time was approximated by linear fitting (no forced intercept) over the initial pseudo-linear regime and converted into observed rate constants with equation (S3). The resulting rate constants as a function of temperature (Table S5) were plotted in an Eyring fashion ($\ln(k\ T^{-1})$ over T^{-1}) and fitted with the Eyring equation (S10).

$$\ln(k_{obs}\ T^{-1}) = \ln(k_b\ h^{-1}) - (H^{\ddagger}\ R^{-1}\ T^{-1}) + (S^{\ddagger}\ R^{-1}) \qquad \text{(S10)}$$

where k_{obs} is the observed rate constant (s^{-1}) at the temperature T (K), k_b is the Boltzmann constant ($1.38\cdot10^{-23}$ J K^{-1}), h is the Planck constant ($6.626\cdot10^{-34}$ J s), R is the universal gas constant (8.314 J mol^{-1} K^{-1}), $H^{\ddagger}$ is the activation enthalpy (J mol^{-1}), and $S^{\ddagger}$ is the activation entropy (J mol^{-1} K^{-1}), assuming that the dimensionless transmission coefficient is one. Fitting of the experimental rates yielded the activation parameters listed in Table S5. As Table S5 and Fig. 2f in the main text illustrate, these PEs showed similar temperature-dependences of their rates, with the exception of *Rc*FPPS whose rate was almost temperature-independent. As such, we conclude that the observed differences in their rates at 25 °C are not due to differences in their temperature optima but rather due to differences in their overall catalytic proficiencies. The full dataset is available from zenodo.org.[2]

Table S5. Temperature-dependent rates and activation parameters of PEs.

PE	[PE] in assay (µg mL^{-1})	k_{obs} (min^{-1}) at 25 / 28 / 31 / 34 °C	$H^{\ddagger}$ (kJ mol^{-1})	$S^{\ddagger}$ (J mol^{-1} K^{-1})	R^2
*Af*GGPPS	6	6.25, 6.30 / 6.93, 6.70 / 8.09, 7.84 / 9.63, 9.32	32.8 ± 2.7	−154.0 ± 8.9	0.955
*Gs*FPPS	3	6.63, 6.70 / 7.25, 7.45 / 8.76, 8.79 / 10.27, 10.71	36.5 ± 2.5	−141.2 ± 8.2	0.968
*Mt*polyPPPS	4	6.52, 6.28 / 7.24, 7.53 / 8.47, 8.42 / 10.13, 9.93	35.1 ± 1.6	−145.9 ± 5.4	0.985
*Rc*FPPS	0.5	61.59, 61.32 / 64.20, 63.37 / 66.24, 66.29, / 67.68, 67.35	5.6 ± 0.6	−225.8 ± 1.9	0.933
*Sf*FPPS	10	5.97, 6.14 / 6.72, 6.91 / 7.84, 7.64 / 9.11, 8.77	30.4 ± 1.6	−162.1 ± 5.2	0.982
*Sp*FPPS	1.5	82.80, 84.48 / 93.38, 93.18 / 103.75, 109.60 / 115.61, 118.76	26.6 ± 1.7	−153.1 ± 5.5	0.974

Screening of PEs with modified substrates

To assess if any of our PEs accepted the modified building blocks, we used EPUB in combination with *in situ* phosphorylation by *Mb*IPK T77A (Fig. S16a). We screened the panel with the analogues of **1x-P** series using **2a-P** as the extender precursor and the analogues of the **2x-P** series using **1a-P** as the starter precursor. To this end, we performed reactions with 300 µM substrate analogue (from the **1x-P** or the **2x-P** series) and 150 µM native substrate precursor (**1a-P** or **2a-P**), 0.6 mM ATP, and 20−80 µg mL^{-1} *Mb*IPK T77A in 1x EPUB buffer with 1x EPUB enzymes in a total volume of 120 µL. The reactions for the **b**-analogues (i.e. **1b-P** and **2b-P**) contained 20 µg mL^{-1} *Mb*IPK T77A, those of the **c**- and **d**-series 40 µg mL^{-1} and those of the **e**-series 80 µg mL^{-1}.These reactions were prepared as master mixes and started by addition of the reaction mixtures (108 µL) to suitably diluted PE (12 µL). The progress of these reactions at 25 °C was monitored at 315 nm for 60 min. Each substrate-PE combination was assayed once with 200 µg mL^{-1} PE, once with 400 µg mL^{-1} PE, and (as negative control) once with 400 µg mL^{-1} PE but without the native substrate precursor. For substrate-PE combination where significant absorbance changes over background signal were observed, we repeated the experiment with different PE concentrations (0−400 µg mL^{-1}) to observe a dose-response relationship, excluding assay artefacts in the previous screening. This analysis showed that almost all PEs were inactive with modified extenders. Only *Af*GGPPS showed measurable activity with **2b-PP** (Fig. S16g), although we were unable to confirm this activity by ^{1}H NMR analysis of reaction products because this transformation did not yield extractable products (data not shown). We previously observed that geranylgeranyl ethers are intractable by extraction,[10] and suspect that a similar phenomenon might be responsible here. In contrast, several PEs accepted modified starters and were thus subjected to further kinetic experiments (see below). Fig. S16 shows some illustrative examples and the full dataset from the screening and follow-up dose-response experiment are available from the externally hosted supplementary information.[2]

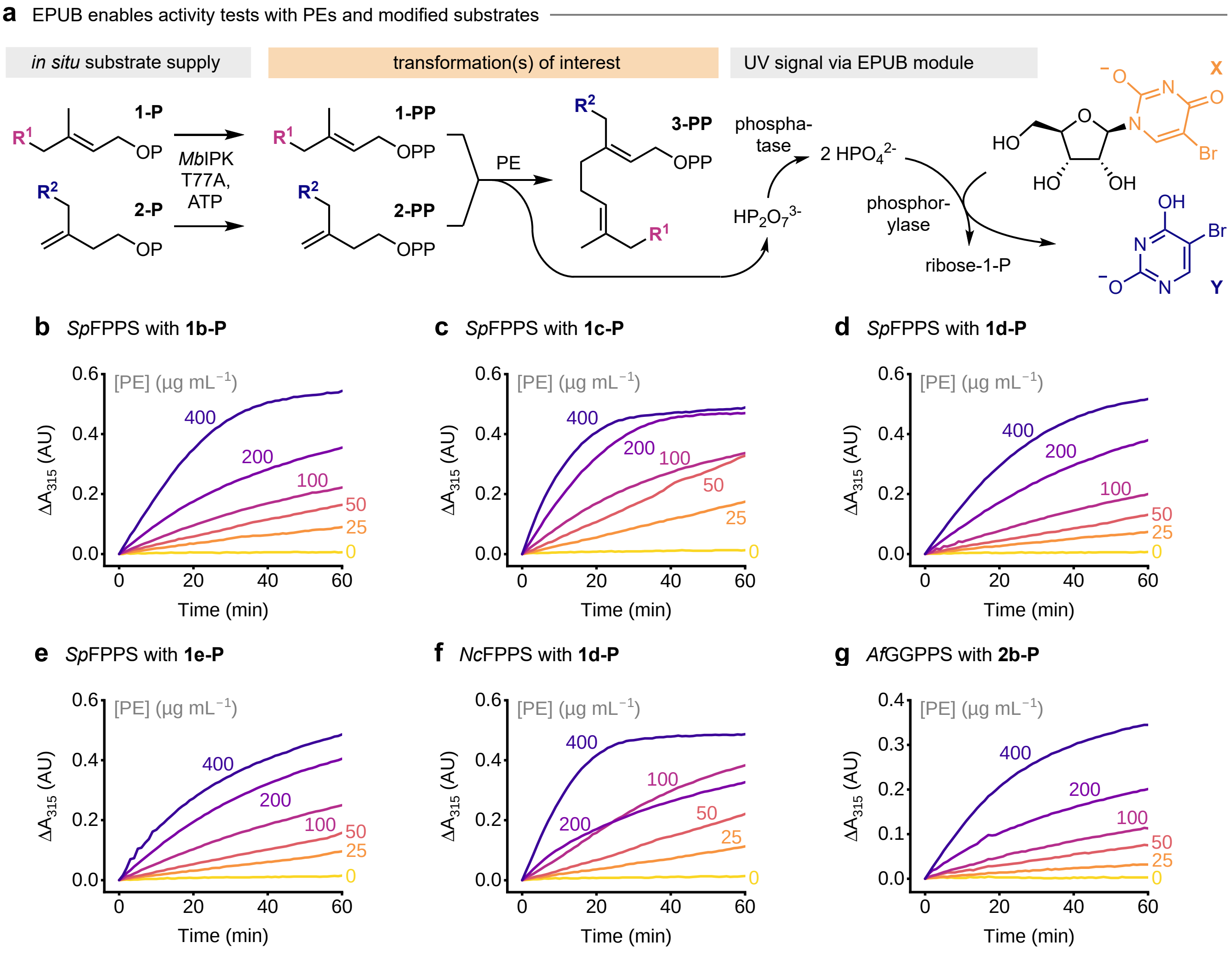

Figure S16. Illustrative reaction courses of PEs with substrate analogues. Note that this is a qualitative experiment and e.g. the rates with **1e-P** do not reflect full pyrophosphorylation to **1e-PP** as this would have required much longer incubation times (see below for the Michaelis-Menten kinetics with PEs).

Kinetic characterization of PEs with modified substrates

Following the initial screening of PEs with the panel of modified substrates, we assessed the performance with each accepted starter analogue (with k_{obs} > 0.05 min^{-1}) via Michaelis-Menten kinetics, using EPUB in combination with IPK-mediated substrate supply (Fig. S16a). To this end, we performed reactions with 40−600 µM starter precursor (13−400 µM for **1e-P**), 150 µM **2a-P**, 1 mM ATP, and 20−240 µg mL^{-1} *Mb*IPK T77A in 1x EPUB buffer with 1x EPUB enzymes in a total volume of 120 µL. These reactions were prepared as master mixes and started by addition of the reaction mixtures (108 µL) to suitably diluted PE (12 µL). Prior to reaction initiation, the master mixes were incubated at 20 °C to allow the IPK to fully pyrophosphorylate the substrate precursors. In addition, each series of reactions with different substrate concentrations was run with two different IPK concentrations to ensure that the incubation was sufficient (which is the case when both series – lower and higher [IPK] – give the same rates for the PE-mediated transformation and is not the case when the series with lower [IPK] consistently gives lower observed rates for the PE-mediated transformation). We used initial scoping runs to determine suitable IPK concentrations and preincubation times. The master mixes containing **1b-P** were preincubated with 20 and 30 µg mL^{-1} *Mb*IPK T77A for 20 min. Those with **1c-P** or **1d-P** with 40 and 60 µg mL^{-1} *Mb*IPK T77A for 20 min. Those with **1e-P** with 160 and 240 µg mL^{-1} *Mb*IPK T77A for 2 h. Following preincubation and reaction initiation, the progress of the PE-mediated transformations at 25 °C was monitored at 315 nm for 60 min. The resulting absorption change over time was approximated by linear fitting (no forced intercept) over the initial pseudo-linear regime and converted into observed rate constants with equation (S3). Correction for background signal (0.0005 AU min^{-1}) and fitting with equation (S4) then yielded kinetic parameters for each PE-starter combination. Several PE-starter combinations (e.g. *Nc*FPPS with **1b-PP** or *Sp*FPPS with **1d-PP**, Fig. S17) gave K_M values well outside the experimental parameter space. For that reason, Fig. 4b in the main text lists the observed rate constants in a standardized fashion (i.e. at 600 µM, the upper end of the experimental parameter space) and we abstain from listing high and error-prone K_M values. Table S6 lists all fitted kinetic parameters, Fig. S17 illustrates representative examples and the full dataset for this experiment is available from the externally hosted supplementary information.[2]

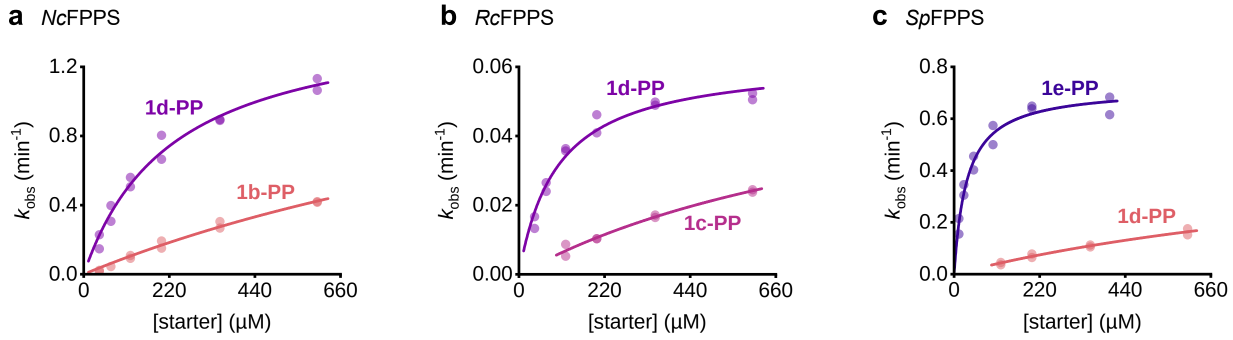

Figure S17. Illustrative Michaelis-Menten plots for PEs with modified starters.

Table S6. Kinetic parameters of PEs with modified substrates.

PE	[PE] in assay (μg mL^{-1})	Starter unit precursor	$k_{obs,max}$ (min^{-1})	K_M (μM)	R^2
*Af*GGPPS		**1b-P**	<0.02		
		**1c-P**	<0.05		
		**1d-P**	<0.05		
	50	**1e-P**	0.51 ± 0.01	32 ± 3	0.923
*Gs*FPPS		**1b-P**	<0.02		
		**1c-P**	<0.02		
		**1d-P**	<0.02		
	50	**1e-P**	0.22 ± 0.01	47 ± 9	0.923
*Hs*FPPS	200	**1b-P**	0.19 ± 0.01	492 ± 64	0.987
	200	**1c-P**	0.43 ± 0.04	331 ± 62	0.965
	200	**1d-P**	0.14 ± 0.01	142 ± 24	0.945
		**1e-P**	<0.02		
*Mt*polyPPPS		**1b-P**	<0.02		
		**1c-P**	<0.02		
		**1d-P**	<0.02		
	100	**1e-P**	0.30 ± 0.02	29 ± 6	0.938
*Nc*FPPS	100	**1b-P**	1.74 ± 0.55	1870 ± 730	0.985
	50	**1c-P**	3.50 ± 2.22	1987 ± 1549	0.943
	50	**1d-P**	1.51 ± 0.09	229 ± 31	0.975
	100	**1e-P**	0.51 ± 0.02	44 ± 6	0.960
*Rc*FPPS	100	**1b-P**	<0.02		
	100	**1c-P**	<0.05		
	100	**1d-P**	0.06 ± 0.01	99 ± 12	0.965
	50	**1e-P**	0.60 ± 0.03	31 ± 5	0.952
*Sf*FPPS	200	**1b-P**	0.64 ± 0.01	235 ± 11	0.997
	200	**1c-P**	1.13 ± 0.04	204 ± 18	0.988
	100	**1d-P**	0.32 ± 0.01	88 ± 7	0.983
	50	**1e-P**	0.49 ± 0.02	11 ± 3	0.778
*Sp*FPPS	50	**1b-P**	fit did not converge	>>800	
	50	**1c-P**	fit did not converge	>>800	
	50	**1d-P**	0.54 ± 0.17	1361 ± 565	0.968
	50	**1e-P**	0.72 ± 0.02	33 ± 4	0.963

Interrogation of the selectivity and efficiency of PEs by NMR

To obtain orthogonal confirmation of the activity of PEs and to probe their selectivity and efficiency with the native and modified substrates, we analyzed their products by NMR (Fig. S5). To this end, we employed *in situ* derivatization of the chain-extended prenyl pyrophosphates to the corresponding glycerol ethers by the archaeal ether synthase *Af*G_3PS. Using this setup, *in situ* phosphorylation by *Mb*IPK T77A, PE-catalyzed chain extension and derivatization by *Af*G_3PS can be run in parallel in one pot because all three enzymes have orthogonal selectivity. In particular, *Af*G_3PS only converts chain-extended prenyl pyrophosphates (see next section below). Subsequent global dephosphorylation with *E. coli* phosphatase (*Ec*AP) yields the free alcohols of any remaining pyrophosphates as well as the free glycerol ethers, which possess characteristic ^{1}H NMR shifts for the (*E*)- (d, 4.04−4.02 ppm) and the (*Z*)-isomer (d, 4.00−3.98 ppm in $CDCl_3$, depending on cosolvent effects, chemical shifts for all relevant analytes in this experiment are given in Table S7).[10] This experimental setup allowed a differentiation between lack of conversion (yielding prenol, **1a-OH**, or any of the respective analogues of the **1x-OH** series), productive chain extension (yielding the glycerol ethers **3xa-G** or **4xaa-G**), and futile hydrolysis by PEs following an initial chain extension (yielding **1x-OH**, **3xa-OH** and/or **4xaa-OH**, since *Af*G_3PS quantitatively converts pyrophosphates to glycerol ether phosphates, Fig. S5). Indeed, we observed extreme cases of all three scenarios. It should be noted that this experiment does not report on futile hydrolysis during the first chain extension step (**1x-PP→3xa-PP**) because the hydrolysis product is the same as non-conversion (**1x-OH**). It is also challenging to differentiate between hydrolysis during the second chain extension (**3xa-PP→4xaa-PP**, with **3xa-OH** as the hydrolysis product) or following the second chain extension (from **4xaa-PP**, with **4xaa-OH** as the hydrolysis product) but estimates can be made based on the integrals of the 6/10-protons around 5.11 ppm. Wherever possible, we indicated the primary hydrolysis product (Table S8). Curiously, we did not find futile hydrolysis products (for any PE) when running extensions from **1b-PP**, **1c-PP** or **1d-PP** but only from **1a-PP** and **1e-PP**., with the exception of *Gs*FPPS M156A which performed a single chain extension directly followed by hydrolysis with **1c-PP** (but not with **1b-PP** pr **1d-PP**). We presently have no explanation for this observation. It should also be noted that geranylgeranyl glycerol ethers (or longer analogues) are intractable for this analysis because they are not extracted into common solvents, potentially due to micelle formation.[10] For that reason, we lack selectivity data for *Af*GGPPS and *Mt*polyPPPS and cannot accurately report on the efficiency of either enzyme because the observed glycerol ethers primarily stem from accidental release of **3xa-PP** or **4xaa-PP** during otherwise processive chain extensions. Indeed, we consistently observed that our PEs are processive enzymes and primarily release their products after all favored chain extensions have been performed (i.e. the FPPSs do not release **3aa-PP** after one chain extension but only **4aaa-PP** after two chain extensions). The “chain-interrupted” analogue **3aa-G** was only accessible with the sterically congested *Gs*FPPS variants S82M and S82L (see below). In all other cases, we primarily only observed the glycerol ethers stemming from the maximum number of favored chain extensions (i.e. **4aaa-G**, **4baa-G** etc.). Chain extensions from **1d-PP** in this setup gave mixtures of **3da-G** and **4daa-G** since some PEs tended to release the product prematurely (i.e. after one extension) while extension from **1e-PP** (which is sterically similar to **3aa-PP**) only gave **3ea-G**, the product of a single chain PE-mediated extension.

We performed these one-pot cascades in 4 mL glass vials containing 0.6 mM starter precursor (**1x-P**), 0.6 mM extender precursor (**2a-P**), 3 mM glycerol-1-phosphate, 3 mM ATP, 8 mM $MgCl_2$, 1.5 µg mL^{-1} *Gt*IPP, 20−120 µg mL^{-1} *Mb*IPK, 200 µg mL^{-1} *Af*G_3PS, and 0.4 mol% (which corresponds to varying concentrations in µg mL^{-1}, see Table S8) PE in 50 mM taurine buffer pH 9 in total volumes of 2 mL. These mixtures were incubated at 20 °C for 18 h before 150 µL *Ec*AP lysate (prepared by lysing the pellet of a 500 mL culture of the expression strain in 25 mL lysis buffer and diluting the resulting lysate with glycerol to give a total of volume of 50 mL) were added and the mixtures were incubated at 20 °C for 24 h. Then, 24 µL of a 50 mM stock solution of propargyl acetate in MeCN (giving a final concentration of 0.6 mM) and 1.3 mL $CDCl_3$ were added to each mixture. Next, the mixtures were shaken for 20 s and centrifuged (200 rpm, 10 min). The organic layers were transferred into NMR tubes through a $MgSO_4$ plug (in a glass pipette), generally yielding 600−800 µL $CDCl_3$ with analytes in the NMR tube. Each sample was then analyzed by ^{1}H NMR (600 MHz, 256 scans). The resulting spectra (available from the externally hosted supplementary information)[2] were analyzed for key signals of the analytes (listed in Table S7) by graphical integration using MestreNova (version 14.2), yielding the efficiency and selectivity data given in Table S8. Figures S16 and 4c in the main text give illustrative examples for some analogues. We used propargyl acetate as an internal standard in these experiments (d,

4.66 ppm in $CDCl_3$) to confirm that the extraction worked and to calculate NMR yield. However, some substrate analogues in our panel showed poor extraction efficiencies into $CDCl_3$ (in particular all compounds derived from **1d-P**) so that NMR yield primarily reported on extraction efficiency and not on conversion. As such, we abstain from reporting NMR yields here, although they may be calculated from the raw spectra available from zenodo.org.[2]

Table S7. Key NMR signals of prenols and glycerol ethers (600 MHz, $CDCl_3$ with trace water and MeCN)

Analyte		δ_H (mult., position)[a]
prenols derived from starting materials	**1a-OH**	5.40 (t, H2), 4.12 (d, H1)
	**1b-OH**	5.65 (t, H2), 4.21 (brs, H1), 3.82 (s, H4), 3.30 (s, OMe)
	**1c-OH**	5.65 (t, H2), 4.21 (brs, H1), 3.86 (s, H4), 3.45 (q, OEt)
	**1d-OH**	5.68 (t, H2), 4.21 (d, H1), 4.11 (d, Opropargyl), 3.96 (s, H4)
	**1e-OH**	5.68 (t, H2), 4.48 (s, OBn), 4.21 (d, H1), 3.92 (s, H4)
isoprenols	**2a-OH**	3.71 (t, H1), 4.86 (s, H4), 4.78 (s, H4)
chain-extended prenols	all-(*E*)-**3aa-OH**	5.40 (t, H2), 5.10 (d, H6), 4.15 (d, H1)
	all-(*E*)-**3ba-OH**	not observed
	all-(*E*)-**3ca-OH**	5.65 (t, H2 and H6), 4.13 (d, H1), 3.98 (s, 8), 3.46 (q, OEt)
	all-(*E*)-**3da-OH**	not observed
	(*E*)-**3ea-OH**	5.40 (t, H2), 5.39 (t, H10), 4.43 (s, OBn), 4.13 (d, H1), 3.88 (s, H8)
	(*Z*)-**3ea-OH**	not observed or identical to (*E*)-**3ea-OH**
	all-(*E*)-**4aaa-OH**	not observed
	all-(*E*)-**4baa-OH**	not observed
	all-(*E*)-**4caa-OH**	not observed
	all-(*E*)-**4daa-OH**	not observed
	**4eaa-OH**	not observed
glycerol ethers	all-(*E*)-**3aa-G**	5.33 (t, H2), 5.10 (d, H6), 4.03 (d, H1)
	all-(*E*)-**3ba-G**	not observed
	all-(*E*)-**3ca-G**	not observed
	all-(*E*)-**3da-G**	not observed
	(*E*)-**3ea-G**	5.39 (t, H2), 5.33 (t, H10), 4.43 (s, OBn), 4.03 (d, H1), 3.88 (s, H8)
	(*Z*)-**3ea-G**	5.40 (t, H2), 5.35 (t, H10), 4.45 (s, OBn), 3.99 (d, H1), 3.88 (s, H8)
	all-(*E*)-**4aaa-G**	5.33 (t, H2), 5.10 (d, H6 and H10), 4.03 (d, H1)
	all-(*E*)-**4baa-G**	5.37 (t, H2), 5.32 (t, H10), 5.10 (t, H6), 4.03 (d, H1), 3.77 (s, H12), 3.26 (OMe)
	all-(*E*)-**4caa-G**	5.37 (t, H2), 5.32 (t, H10), 5.10 (t, H6), 4.03 (d, H1), 3.82 (s, H12), 3.41 (q, OEt)
	all-(*E*)-**4daa-G**	5.41 (t, H2), 5.32 (t, H10), 5.10 (t, H6), 4.06 (d, Opropargyl), 4.03 (d, H1), 3.92 (s, H12)
	**4eaa-G**	not observed

[a] the alkene protons listed as t are sometimes resolved to tq with coupling constants as reported in the synthesis section toward the end of this SI.

Table S8. Selectivity of PEs with native and modified substrates.[a]

PE	[PE] in assay (µg mL^{-1}) corres-ponding to 0.4 mol%	Starter unit precursor	**1x-OH** (%)	**3xa-OH** (%)	**3xa-G** or **4xaa-G** (%)	**2a-OH** (y or n)[b]	Efficiency[c] (%)	Selectivity
*Af*GGPPS	55	**1a-P**	n.d.[d]	n.d.[d]	n.d.[d]	n		>99% (*E*)
		**1b-P**	>99	<1	<1	y		>99% (*E*)
		**1c-P**	88	<1	12	y	>99	>99% (*E*)
		**1d-P**	91	<1	9	y	>99	>99% (*E*)
		**1e-P**	24	6	70	n	92	>99% (*E*)
*Gs*FPPS	29	**1a-P**	51	12	37	n	76	>99% (*E*)
		**1b-P**	>99	<1	<1	y		>99% (*E*)
		**1c-P**	>99	<1	<1	y		>99% (*E*)
		**1d-P**	86	<1	14	y	>99	>99% (*E*)
		**1e-P**	<1	2	98	n	98	>99% (*E*)
*Hs*FPPS	132	**1a-P**	37	17	46	n	73	>99% (*E*)
		**1b-P**	67	<1	33	y	>99	>99% (*E*)
		**1c-P**	51	<1	49	n	>99	>99% (*E*)
		**1d-P**	65	<1	35	y	>99	>99% (*E*)
		**1e-P**	38	10	52	y	84	>99% (*E*)
*Mt*polyPPPS	38	**1a-P**	n.d.[d]	n.d.[d]	n.d.[d]	n		>99% (*E*)
		**1b-P**	>99	<1	<1	y		>99% (*E*)
		**1c-P**	>99	<1	<1	y		>99% (*E*)
		**1d-P**	>99	<1	<1	y		>99% (*E*)
		**1e-P**	20	30	50	y	63	>99% (*E*)
*Nc*FPPS	128	**1a-P**	56	11	33	n	75	>99% (*E*)
		**1b-P**	30	<1	70	y	>99	>99% (*E*)
		**1c-P**	51	<1	49	n	>99	>99% (*E*)
		**1d-P**	64	<1	36	n	>99	>99% (*E*)
		**1e-P**	43	1	56	y	98	>99% (*E*)
*Rc*FPPS	35	**1a-P**	54	8	38	n	83	>99% (*E*)
		**1b-P**	>99	<1	<1	y		>99% (*E*)
		**1c-P**	81	<1	19	y	>99	>99% (*E*)
		**1d-P**	77	<1	23	y	>99	>99% (*E*)
		**1e-P**	<1	<1	>99	n	>99	>99% (*E*)
*Sf*FPPS	128	**1a-P**	64	12	24	y	67	>99% (*E*)
		**1b-P**	56	<1	44	n	>99	>99% (*E*)
		**1c-P**	56	<1	44	n	>99	>99% (*E*)
		**1d-P**	67	<1	33	n	>99	>99% (*E*)
		**1e-P**	4	44	52	n	54	>99% (*E*)
*Sp*FPPS	114	**1a-P**	54	9	37	y	80	>99% (*E*)
		**1b-P**	29	<1	71	n	>99	>99% (*E*)
		**1c-P**	51	<1	49	n	>99	>99% (*E*)
		**1d-P**	62	<1	38	n	>99	>99% (*E*)
		**1e-P**	<1	7	93	n	93	>99% (*E*)

[a] The rations of the different analytes were derived from the allylic protons in the 1-position (≈4.2−4.0 ppm). Note that equal quantities of **1x-P** and **2a-P** were applied, which means that PEs performing two chain extensions will inevitably leave unreacted **1x-P** behind even if all **2a-P** is consumed. Also note that due to large differences in extraction efficiencies, the mass balances might be skewed. [b] yes or no. [c] Calculated as **3xa-G**/(**3xa-OH**+**3xa-G**). [d] This PE makes geranylgeranyl chains or longer analogues which are not extractable as their glycerol ethers and/or may not be converted to glycerol ethers by *Af*G_3PS.

Selectivity of *Af*G_3PS for chain-extended prenyl pyrophosphates

We sought to use *Af*G_3PS to determine the (*E*)/(*Z*)-selectivity of PE-mediated chain extension. With the native substrates, *Af*G_3PS is selective for chain-extended prenyl pyrophosphates:[10] It is inactive with prenyl pyrophosphate (**1a-PP**) but active with geranyl (**3aa-PP**) and farnesyl (**4aaa-PP**) pyrophosphate. Since it also converts various chain-modified prenyl pyrophosphates (as long as the frontal alkene is present), we surmised that it might also accept (and thus report on the configuration of) ether-modified prenyl pyrophosphates such as the analogues **3xa-PP** or **4xaa-PP**. To enable a one-pot chain-extension-etherification sequence, we additionally needed to exclude *Af*G_3PS converting any of the starter units **1b-PP**, **1c-PP**, **1d-PP** or **1e-PP**. We initially probed *Af*G_3PS's activity with these analogues by EPUB and observed no significant activity (data not shown). However, at high protein concentrations, EPUB tends to give high background signals and is therefore not ideal to definitively rule out trace activities. As such, we additionally performed reactions containing high *Af*G_3PS concentrations over long incubation times to exclude activity with these analogues. To this end, we performed one-pot cascades with each analogue featuring either a) phosphorylation by *Mb*IPK T77A and potential etherification by *Af*G_3PS or b) phosphorylation by *Mb*IPK T77A, chain extension by *Sp*FPPS and potential etherification by *Af*G_3PS and analyzed these mixtures by ^{31}P NMR. *Af*G_3PS-mediated etherifications can be traced by ^{31}P NMR through consumption of the organopyrophosphate substrate (d at −6 ppm and d at −9.5 ppm in H_2O/D_2O), production of inorganic pyrophosphate (s at −5.6 ppm), consumption of glycerol-1-phosphate (4.0 ppm) and production of the glycerol phosphate ether (3.9 ppm). If *Af*G_3PS converted the non-extended starter unit, we expected consumption of the organophosphate substrate without the presence of *Sp*FPPS (in conditions a)). If *Af*G_3PS converted the chain-extended starter unit, we expected consumption of the organophosphate substrate in the presence of *Sp*FPPS (in conditions b)). If *Af*G_3PS converted none of these analogues, we expected no consumption of the organophosphate substrate under any condition a) or b). In addition, consumption of the organopyrophosphate should go hand-in-hand with appearance of the sharp peak for the glycerol phosphate ether.

Thus, to probe if *Af*G_3PS converted any of the analogues **1x-PP** or their chain extended analogues **3xa-PP** or **4xaa-PP**, we performed reactions with 1 mM starter precursor (**1x-P**), 1 mM **2a-P**, 3 mM glycerol-1-phosphate, 3 mM ATP, 10 mM $MgCl_2$, 2 µg mL^{-1} *Gt*IPP, 30−240 µg mL^{-1} *Mb*IPK T77A, 200 µg mL^{-1} *Af*G_3PS, and 0 or 500 µg mL^{-1} *Sp*FPPS in 50 mM taurine buffer pH 9 with 10% (v/v) D_2O in total volumes of 650 µL. Mixtures containing **1a-P** or **1b-P** featured 30 µg mL^{-1} *Mb*IPK T77A, those with **1c-P** 120 µg mL^{-1} *Mb*IPK T77A, those with **1d-P** 60 µg mL^{-1} *Mb*IPK T77A, and those with **1e-P** 240 µg mL^{-1} *Mb*IPK T77A. These mixture were incubated at 20 °C for 2 h (until the initial phosphorylation by *Mb*IPK T77A was complete) in plastic reaction tubes, transferred into NMR tubes and analyzed by ^{31}P NMR (243 MHz, 128 scans) to confirm that the mass balances are met and the phosphorylation proceeded. The mixtures were then incubated for an additional 18 h in their NMR tubes and analyzed by ^{31}P NMR again to check for consumption of the pyrophosphate and potential etherification. Figure S17 below illustrates representative data for this series of experiments, showing that *Af*G_3PS did not convert the non-extended starter units **1x-P** but did quantitatively convert the chain-extended analogues **3xa-PP** and/or **4xaa-PP**. Any remaining pyrophosphate species in the reactions featuring *Af*G_3PS and *Sp*FPPS correspond to ADP and remaining extender **2a-PP**. All raw spectra for this experiment are available from the externally hosted supplementary information.[2]

Orthogonal to these ^{31}P NMR experiments, we also did not observe any glycerol ethers in the one-pot transformations described above (Fig. S5 and Table S8). After dephosphorylation, mixtures with inactive or without PE consistently returned the alcohol of the unreacted starter unit and not its glycerol ether, providing further support that *Af*G_3PS does not form ethers from the analogues **1x-PP** and is therefore selective for chain-extended prenyl pyrophosphates, which it converts to their ethers quantitatively.

Mutagenesis and initial screening of *Gs*FPPS and *Sp*FPPS variants

To assess if substitutions of non-conserved active site residues (NCRs; see below for bioinformatic analysis and identification of these residues) in *Gs*FPPS and *Sp*FPPS are tolerated and lead to changes in their characteristics (biochemical, kinetic, substrate scope), we created small, defined libraries of these two PEs. To this end, we created libraries at four positions for each enzyme by saturation mutagenesis with NNK primers and sequenced 24 members of each library. All unique members of these libraries (excluding the proline variants) were then produced on a small scale, subjected to an initial screen with the native substrates and (if active) produced on a larger scale and fully characterized.

The *Gs*FPPS and *Sp*FPPS libraries were generated by site saturation mutagenesis following the NEB Phusion High-Fidelity DNA Polymerase protocol using the primers listed in Table S9. The PCR mixtures for pACYC_GsFPPS (50 µL total volume) contained 1 µL 10 ng µL^{-1} template plasmid, 1 µL 10 mM dNTPs, 2.5 µL 25 µM forward primer, 2.5 µL 25 µM reverse primer, 1.5 µL DMSO, 0.5 µL Phusion DNA polymerase (New England Biolabs), 10 µL 5xPhusion GC buffer, and 31 µL sterile water. The PCR cycling programming featured an initial denaturation at 98 °C for 1 min, 30 cycles of denaturation at 98 °C for 10 s, annealing for 30 s, and extension at 72 °C for 100 s, followed by a final extension at 72 °C for 10 min. Depending on the PCR, various annealing temperatures were used: 60 °C for GsFPPS_T80X and GsFPPS_S82X, 68 °C for GsFPPS_M156X and GsFPPS_L188X. The PCR mixture for GsFPPS_M156X contained an additional 2% DMSO and was run at a temperature gradient from 68−72 °C annealing temperature to minimize secondary structure formation of this GC-rich primer. The PCR mixtures for pACYC_SpFPPS (50 µL total volume) contained 1 µL 10 ng µL^{-1} template plasmid, 1 µL 10 mM dNTPs, 2.5 µL 10 µM forward primer, 2.5 µL 10 µM reverse primer, 1.5 µL DMSO, 0.5 µL Phusion DNA polymerase, 10 µL 5xPhusion HF buffer, and 32.5 µL sterile water. The PCR cycling programming featured an initial denaturation at 98 °C for 30 s, 30 cycles of denaturation at 98 °C for 10 s, annealing at 55 °C for 30 s, and extension at 72 °C for 160 s, followed by a final extension at 72 °C for 10 min. Following the amplification, the PCR products were analyzed by gel electrophoresis using a 0.8% agarose gel stained with ROTI-GelStain. The PCR products were then treated with 1 µL DpnI (New England Biolabs) for 2 h at 37 °C to digest parental methylated DNA and the resulting products were purified with the QIAquick PCR Purification kit (Qiagen). Next, 5 µL of the PCR products were used to transform chemically competent *E. coli* DH5α using the heat-shock protocol described on page 4 of the SI. After transformation, recovery, and overnight growth on a plate (LB medium with 34 µg mL^{-1} chloramphenicol), the clones for each library were pooled and their plasmid DNA was extracted using the QIAprep Miniprep kit (Qiagen). After Sanger sequencing confirmed successful randomization of the desired position, each library was used to transform chemically competent *E. coli* BL21(DE3) cells using the same heat shock protocol. After transformation, recovery, and overnight growth on a plate, 24 members of each library were grown in 4 mL TB medium with 34 µg mL^{-1} chloramphenicol in a 24-well plate at 37 °C and 700 rpm overnight. From these cultures, 0.5 mL were used to create a glycerol stock of each member by mixing with 0.5 mL 50% sterile glycerol and freezing at −70 °C and 3.5 mL were used to extract plasmid DNA for Sanger sequencing. From the 24 sequenced *E. coli* BL21(DE3) colonies per library, variants exhibiting high-quality sequencing results with a single, unambiguous amino acid substitution at the intended site were selected for screening. This yielded 26 unique variants for *Gs*FPPS and 32 unique variants for *Sp*FPPS (Table S9; sequencing results available from the externally hosted supplementary information).[2]

For the initial screening, all variants in the *Gs*FPPS and *Sp*FPPS libraries were produced on a small scale through IPTG-induced overexpression followed by parallelized purification. To this end, a 0.5 mL preculture was grown for each strain harboring a variant in wells of a 24-deep-well plate in LB medium supplemented with 34 µg mL^{-1} chloramphenicol at 37 °C and 900 rpm for 6 h. Then, this preculture was diluted with 4.5 mL TB medium supplemented with 34 µg mL^{-1} chloramphenicol and 0.1 mM IPTG and the culture was incubated at 37 °C and 900 rpm for 16 h. Next, the cells were harvested by centrifugation (4000 rpm, 20 min, 4 °C) and resuspended in 1 mL binding buffer (100 mM taurine, 100 mM NaCl, 20 mM imidazole, pH 9) containing 2 g L^{-1} lysozyme and DNAse (ca. 5 ng mL^{-1}). To lyse the cells, this suspension was then incubated at 20 °C and 800 rpm for 30 min and subsequently frozen overnight at −20 °C. The next morning, the frozen cell suspensions were thawed at 20 °C for 2 h, transferred to 1.5 mL reaction tubes and centrifuged (13,000 rpm, 30 min, 4 °C). Next, the supernatant was loaded onto a HisTrap™ MultiTrap Ni-NTA 96-well plate previously equilibrated with binding buffer. The plate was centrifuged (400 rpm, 2 min, 4 °C) and the flow-through was discarded. Each well was washed twice with 200 µL binding buffer and the

entire flow-through was discarded. Next, 150 µL elution buffer (100 mM taurine, 100 mM NaCl, 500 mM imidazole, pH 9) was added to each well and the flow-through was collected in a fresh 96-well plate. For the screening, 10 µL of each elution fraction were added wells of a UV-transparent 96-well plate and assayed with EPUB. The EPUB reaction mixture contained 150 µM **1a-P**, 150 µM **2a-P**, and 30 µg mL^{-1} *Mb*IPK T77A in 1x EPUB buffer with 1x EPUB enzymes in a final volume of 200 µL. Each reaction was started by adding a master mix of the reaction mixture (190 µL) to the elution fraction of each variant (10 µL). The progress of these reactions at 25 °C was monitored at 315 nm for 30 min. This qualitative screen identified which variants retained activity (Table S10) and could be advanced for a full characterization. Because most *Sp*FPPS variants were inactive, we repeated this production and screening for the SpFPPS variants and ensured continuous cooling of the samples throughout the process to avoid any undesired thermal inactivation of labile variants. This repetition yielded the same results.

Table S9. Primers used for mutagenesis of *Gs*FPPS and *Sp*FPPS and unique variants obtained.

PE	position_fw/ rev	Primer (5′→3′)	Unique variants obtained and screened
*Gs*FPPS	T80X_fw<br>T80X_rev	GAAATGATTCATNNKTATTCGCTGATTCATGATG<br>GAATCAGCGAATAMNNATGAATCATTTCAATGGC	T80… A, S, G, R, L, Q, Y
	S82_fw<br>S82X_rev	CATACCTATNNKCTGATTCATGATGATCTG<br>CATGAATCAGMNNATAGGTATGAATCATTTCAATG	S82… A, T, G, M[a], L
	M156_fw<br>M156_rev	GGAAGGCNNKGTGGCGGGTCAGGCGGCGGATATG<br>CGCCACMNNGCCTTCCGGGCCGGCCGCCTTC	M156… A, N, E, G, R, W
	L188_fw<br>L188X_rev	GGCAAAATGNNKCAGTATAGCGTGCATGCGGG<br>GCACGCTATACTGMNNCATTTTGCCGGTTTTGTGG	L188… A, T, S, E, H, M, R, G
*Sp*FPPS	S89X_fw<br>S89X_rev	CTGCTGCAGNNKTTCTTTCTGATCGCGGATGAT<br>GATCAGAAAGAAMNNCTGCAGCAGCTCCACCATCC	S89… A, V, E, G, R, H, E, W, L
	F91_fw<br>F91_rev	GCAGAGCTTCNNKCTGATCGCGGATGATATCATGG<br>TCCGCGATCAGMNNGAAGCTCTGCAGCAGCTC	F91… G, R, H, S, A, I, V, E, Q, K
	T159_fw<br>T159_rev	CCTTTCAGNNKGAACTGGGCCAGCAGCTGGATC<br>GCTGGCCCAGTTCMNNCTGAAAGGTCACATCATGAAACAG	T159… S, V, W, Q, A, E, R, K, C
	S197_fw<br>S197_rev	CGTTCTACNNKTTTTACCTGCCGGTGGCGC<br>GCAGGTAAAAMNNGTAGAACGCGGTTTTATAGATGACAATAAAGC	S197… T, R, F, V

[a] *Gs*FPPS S156M had a D29E substitution on the protein surface due to a serendipitous A→T mutation. Since this protein was produced well and had similar biochemical characteristics to the S82L variant, we conclude that the D29E substitution has no major impact on the key characteristics we examined.

Table S10. Retention of activity with the native substrates by the *Gs*FPPS and *Sp*FPPS variants.

PE	position	Active variants	Inactive variants
*Gs*FPPS	T80	G, A, S, Q, R, L, Y	
	S82	G, A, T, M, L	
	M156	G, A, N, E	R, W
	L188	A, S, T, M, H, E	G, R
*Sp*FPPS	S89	A, E, G, V	R, H, E, W, L
	F91	I	G, R, H, S, A, V, E, Q, K
	T159	S	V, W, Q, A, E, R, K, C
	S197	T	R, F, V

Production, purification, and characterization of the *Gs*FPPS and *Sp*FPPS variants

All active *Gs*FPPS and *Sp*FPPS variants (Table S10) were produced and purified on a larger scale for characterization. Following the routine protein production and purification workflow (page 26), all variants were purified from an 80 mL main culture of the expression strain, yielding good to excellent yields of the variants (generally ≈50 mg pure protein per liter of culture for *Gs*FPPS variants and ≈80 mg for *Sp*FPPS variants. The identity of all variants was confirmed by UPLC-MS (Table S11) in analogy to the methods described for the wild-type PEs (page 27), which showed the expected molecular weights for all proteins, with the exception of *Gs*FPPS L188E which exhibited a 273 Da mass increase compared to the expected mass. We re-isolated and re-sequenced the plasmid of this strain and found no off-target mutations and thus hypothesize that this variant may bind strongly to a Mg^{2+} ion and an (iso-)prenyl pyrophosphate (+267 Da) due to the increased charge in the active site.

Next, we assessed the thermal stability of the *Gs*FPPS and *Sp*FPPS variants. To this end, we measured their residual activity following incubation periods at incrementally higher temperatures, in analogy to the procedure described above for the wild-type PEs (page 45). The variants were incubated in 10 mM taurine buffer pH 9 with 10% (v/v) glycerol in a total volume of 180 µL in a PCR tube. We used initial kinetic experiments at different PE concentrations to determine suitable enzyme concentrations for the assay (and, in turn, for the incubation step). The tube was then placed in a PCR cycler and heated for 5 min each at different temperatures (35, 40, 45, 50, 55, 60, 65, 70, 75, 80, 85, 90 °C for *Gs*FPPS and 24, 28, 32, 36, 40, 44, 48, 52, 56 and 60 °C for *Sp*FFPS). After each incubation step, the tube was cooled to 20 °C and a 20 µL sample was withdrawn and pipetted into a well of a UV-transparent 96-well plate which was stored at 4 °C until the end of the incubation times. This experiment was carried out in duplicate, yielding a total of 24 drops of 20 µL preincubated PE in a multi-well plate for *Gs*FPPS and 20 for *Sp*FPPS. These enzyme samples were then assayed for residual activity by adding 180 µL of reaction mixture to give final concentrations of 150 µM **1a-P**, 150 µM **2a-P**, 0.5 mM ATP, 20 µg mL^{-1} *Mb*IPK T77A, and 20 µL preincubated PE in 1x EPUB buffer with 1x EPUB enzymes in a total volume of 200 µL. The progress of these reactions at 25 °C was monitored at 315 nm for 10 min. The resulting absorption change over time was approximated by linear fitting (no forced intercept) over the initial pseudo-linear regime and converted into observed rate constants with equation (S3). This yielded residual rate constants as a function of preincubation temperature, which were fitted with the Boltzmann-type relationship (S2), yielding an apparent T_{50} value for each PE. The incubation temperature and fitted parameters for all variants are listed in Table S12 and (for *Gs*FPPS) illustrated in Figure 5b. This analysis revealed that the variants generally retained the stability of the parent enzyme. Although some variants exhibted up to 5 °C differences in their T_{50} value, we did not observe drastic losses of stability with either PE.

Next, we obtained Michaelis-Menten kinetics for the *Gs*FPPS and *Sp*FPPS variants to assess their affinity and inhibitory profiles. Following initial scoping experiments to determine suitable enzyme concentrations, we obtained kinetic profiles for the first chain extension (**1a-PP** + **2a-PP**) in analogy to experiments for the wild-type described above (page 47). Differing from the experiments described above, we used EPUB in combination with *in situ* phosphorylation by *Mb*IPK T77A. To this end, we performed reactions with 120 µM excess substrate precursor and 10−220 µM limiting substrate precursor, 0.5 mM ATP, and 20 µg mL^{-1} *Mb*IPK T77A in 1x EPUB buffer with 1x EPUB enzymes in a total volume of 200 µL. The progress of these reactions at 25 °C was monitored at 315 nm for 12 min. The resulting absorption change over time was approximated by linear fitting (no forced intercept) over the initial pseudo-linear regime and converted into observed rate constants with equation (S3). The resulting rate constants as a function of initial substrate concentration were fitted with either the Michaelis-Menten equation (S4) or equation (S5) if inhibition was observed. Table S13 lists enzyme concentrations and fitted kinetic parameters, while Fig. 5b in the main text provides a visual summary. The illustrative kinetic profiles shown in Fig. S3 are also representative of the results of this experiment.

Next, we measured the rates of the *Gs*FPPS and *Sp*FPPS variants with substrate analogues. To this end, we assessed the performance of each variant with the starters **1a-P**, **1b-P**, **1c-P**, **1d-P**, **1e-P**, and **3aa-P** (all in combination with **2a-P**) and the extender **2b-P** (in combination with **1a-P**) at different enzyme concentrations to estimate an observed rate constant. We performed reactions with 300 µM precursor of the substrate of interest, 150 µM of the native counterpart precursor (**2a-P** for the starter analogues, **1a-P** for the extender analogue), 0.5 mM ATP, 30−150 µg mL^{-1} *Mb*IPK T77A, and various

concentrations of PE variant in 1x EPUB buffer with 1x EPUB enzymes in a total volume of 120 µL (for the *Gs*FPPS variants) or 200 µL (for the *Sp*FPPS variants). The master mixes containing **1a-P**, **3aa-P**, **1b-P**, **1d-P** or **2a-P** were preincubated with 30 µg mL^{-1} *Mb*IPK T77A for 30 min. Those with **1c-P** 80 µg mL^{-1} *Mb*IPK T77A for 30 min. Those with **1e-P** with 150 µg mL^{-1} *Mb*IPK T77A for 1.5 h. The progress of these reactions at 25 °C was monitored at 315 nm for 10 min. If the absorption change over the course of the reaction followed a pseudo-zero order rate law (i.e. was linear), we used the difference between the absorption at 0 and 10 min (corrected for background signals, see Table S14) to estimate a rate constant with equation (S3). The results are summarized in Table S14 and all kinetic traces are available from the externally hosted supplementary information.[2] For Fig. 5b, we presented the median of three experimental k_{obs} value instead of averaging multiple data points to avoid biases from background correction at different enzyme concentrations or from outliers are particularly high/low enzyme concentrations. In analogy, we also examined if *Gs*FPPS L188A, L188S, or L188T could convert the modified extenders **2c-PP**, **2d-PP** or **2e-PP** but observed no activity (data not shown).

Lastly, we examined the efficiency and selectivity of the *Gs*FPPS and *Sp*FPPS variants by subjecting the products of their chain extensions with **1a-PP** and **1e-PP** to analysis by ^{1}H NMR. In analogy to experiments described for the wild-type PEs (page 54 and following), 0.6 mM starter precursor (**1x-P**), 0.6 mM extender precursor (**2a-P**), 3 mM glycerol-1-phosphate, 3 mM ATP, 8 mM $MgCl_2$, 1.5 µg mL^{-1} *Gt*IPP, 20−120 µg mL^{-1} *Mb*IPK, 200 µg mL^{-1} *Af*G_3PS, and 0.4 mol% (14 µg mL^{-1} for *Gs*FPPS variants and 57 µg mL^{-1} for *Sp*FPPS variants) PE in 50 mM taurine buffer pH 9 in total volumes of 2 mL. These mixtures were incubated at 20 °C for 18 h before 150 µL *Ec*AP lysate (prepared by lysing the pellet of a 500 mL culture of the expression strain in 25 mL lysis buffer and diluting the resulting lysate with glycerol to give a total of volume of 50 mL) were added and the mixtures were incubated at 20 °C for 24 h. Then, 24 µL of a 50 mM stock solution of propargyl acetate in MeCN (giving a final concentration of 0.6 mM) and 1.3 mL $CDCl_3$ were added to each mixture. Next, the mixtures were shaken for 20 s and centrifuged (200 rpm, 10 min). The organic layers were transferred into NMR tubes through a $MgSO_4$ plug (in a glass pipette), generally yielding 600−800 µL $CDCl_3$ with analytes in the NMR tube. Each sample was then analyzed by ^{1}H NMR (600 MHz, 256 scans). The resulting spectra (available from the externally hosted supplementary information)[2] were analyzed for key signals of the analytes (listed in Table S7) by graphical integration using MestreNova (version 14.2), yielding the efficiency and selectivity data given in Table S15 and partially summarized in Fig. 5b in the main text.

The raw data for the experiments described above are available from the externally hosted supplementary information.[2]

Table S11. Mass spectrometry results for the *Gs*FPPS and *Sp*FPPS variants.

PE	Expected molecular weight (Da)[a]	Observed molecular weight (Da)	Difference (calc−obs; Da)
GsFPPS wt	34303.1	34302.3	0.8
*Gs*FPPS T80A	34273.1	34271.0	2.1
*Gs*FPPS T80S	34289.1	34287.4	1.7
*Gs*FPPS T80G	34259.1	34258.3	0.8
*Gs*FPPS T80R	34358.2	34357.1	1.1
*Gs*FPPS T80L	34315.2	34313.1	2.1
*Gs*FPPS T80Q	34330.2	34329.1	1.1
*Gs*FPPS T80Y	34365.2	34365.0	0.2
*Gs*FPPS S82A	34287.1	34286.9	0.2
*Gs*FPPS S82T	34317.1	34317.0	0.1
*Gs*FPPS S82G	34273.1	34273.0	0.1
*Gs*FPPS S82M[b]	34333.2	34332.1	1.1
*Gs*FPPS S82L	34329.2	34328.2	1.0
*Gs*FPPS M156A	34243.0	34241.9	1.1
*Gs*FPPS M156N	34286.0	34286.3	0.3
*Gs*FPPS M156E	34301.0	34299.5	1.5
*Gs*FPPS M156G	34229.0	34229.0	0
*Gs*FPPS L188A	34261.0	34260.6	0.4
*Gs*FPPS L188T	34291.0	34291.1	0.1
*Gs*FPPS L188S	34277.0	34276.0	1.0
*Gs*FPPS L188E	34319.1	34592.0	272.9
*Gs*FPPS L188H	34327.1	34327.2	0.1
*Gs*FPPS L188M	34321.1	34319.5	1.6
*Sp*FPPS wt	41509.4	41507.5	1.9
*Sp*FPPS S89A	41493.4	41493.5	0.1
*Sp*FPPS S89E	41551.5	41550.5	1.0
*Sp*FPPS S89G	41479.4	41478.7	0.7
*Sp*FPPS S89V	41521.5	41520.4	1.1
*Sp*FPPS F91I	41475.4	41475.5	0.1
*Sp*FPPS T159S	41495.4	41494.8	0.6
*Sp*FPPS S197T	41523.4	41521.2	2.2

[a] calculated with ProtParam (https://web.expasy.org/protparam/) for the sequences listed above, considering the loss of the *N*-terminal methionine. [b] Also featuring a D29E substitution.

Table S12. Thermal stability of *Gs*FPPS and *Sp*FPPS variants.[a]

PE	Final [PE] in assay (µg mL^{-1})	$k_{obs,0}$ (min^{-1})	$k_{obs,1}$ (min^{-1})	T_{50} (°C)	s[c]	R^2
GsFPPS wt	2.5	0.08 ± 0.08	4.87 ± 0.06	69.1 ± 0.2	1	0.989
*Gs*FPPS T80A	2.5	0.30 ± 0.11	8.29 ± 0.08	72.1 ± 0.3	1	0.994
*Gs*FPPS T80S	4.0	0.21 ± 0.08	6.91 ± 0.05	69.3 ± 0.1	1	0.997
*Gs*FPPS T80G	5.0	0.28 ± 0.05	5.22 ± 0.05	68.4 ± 0.2	1	0.995
*Gs*FPPS T80R	2.5	0.11 ± 0.13	7.38 ± 0.10	68.7 ± 0.2	1	0.988
*Gs*FPPS T80L	2.5	0.25 ± 0.08	6.38 ± 0.06	70.4 ± 0.1	1	0.994
*Gs*FPPS T80Q	2.5	0.10 ± 0.02	2.11 ± 0.01	67.9 ± 0.2	1	0.997
*Gs*FPPS T80Y	3.5	0.07 ± 0.03	1.31 ± 0.02	69.0 ± 0.2	1	0.981
*Gs*FPPS S82A	10.0	0.05 ± 0.02	1.85 ± 0.02	67.5 ± 0.2	1	0.996
*Gs*FPPS S82T	2.5	0.26 ± 0.08	7.51 ± 0.06	68.4 ± 0.1	1	0.996
*Gs*FPPS S82G	8.0	0.00 ± 0.02	1.06 ± 0.01	68.8 ± 0.2	1	0.991
*Gs*FPPS S82M[b]	8.0	0.03 ± 0.05	2.68 ± 0.04	68.1 ± 0.3	1	0.987
*Gs*FPPS S82L	10.0	0.01 ± 0.01	1.12 ± 0.01	67.8 ± 0.2	1	0.996
*Gs*FPPS M156A	2.0	0.04 ± 0.01	1.21 ± 0.01	67.3 ± 0.2	1	0.998
*Gs*FPPS M156N	12.0	0.01 ± 0.02	1.21 ± 0.02	64.3 ± 0.1	1	0.992
*Gs*FPPS M156E	5.0	0.10 ± 0.09	3.52 ± 0.08	65.9 ± 0.3	1	0.974
*Gs*FPPS M156G	2.5	0.01 ± 0.07	4.71 ± 0.06	66.4 ± 0.2	1	0.992
*Gs*FPPS L188A	1.5	0.15 ± 0.03	2.07 ± 0.03	66.0 ± 0.2	1	0.987
*Gs*FPPS L188T	1.5	2.18 ± 0.30	13.69 ± 0.22	70.0 ± 0.2	1	0.977
*Gs*FPPS L188S	1.0	0.30 ± 0.07	11.13 ± 0.07	63.7 ± 0.1	1	0.998
*Gs*FPPS L188E	8.0	0.03 ± 0.01	0.50 ± 0.01	63.5 ± 0.3	1	0.986
*Gs*FPPS L188H	2.5	1.54 ± 0.15	7.87 ± 0.14	65.2 ± 0.2	1	0.977
*Gs*FPPS L188M	2.5	0.45 ± 0.12	4.40 ± 0.09	69.4 ± 0.3	1	0.966
*Sp*FPPS wt	2.0	6.67 ± 0.10	38.00 ± 0.44	40.8 ± 0.2	1.5	0.994
*Sp*FPPS S89A	2.0	6.25 ± 0.55	39.72 ± 0.48	44.1 ± 0.2	1.5	0.992
*Sp*FPPS S89E	20.0	1.26 ± 0.13	3.27 ± 0.08	45.6 ± 0.9	1.5	0.958
*Sp*FPPS S89G	2.0	5.56 ± 0.55	32.11 ± 0.61	40.3 ± 0.3	1.5	0.982
*Sp*FPPS S89V	2.0	5.93 ± 0.45	33.67 ± 0.52	39.6 ± 0.2	1.5	0.989
*Sp*FPPS F91I	2.0	3.66 ± 0.27	27.24 ± 0.32	38.8 ± 0.2	1.5	0.994
*Sp*FPPS T159S	5.0	4.35 ± 0.38	21[d]	35.9 ± 0.5	3.8 ± 0.4	0.979
*Sp*FPPS S197T	2.0	5.30 ± 0.52	26.17 ± 0.61	39.0 ± 0.4	1.5	0.974

[a] Note that the rate constants here may not be directly comparable to other kinetic experiments reported in this manuscript because *Gs*FPPS is highly substrate-inhibited. [b] Also featuring a D29E substitution. [c] fixed at 1 for the *Gs*FPPS variants and at 1.5 for the *Sp*FPPs variants (with the exception of the T159S variant which showed an atypical deactivation profile) to reducing fitting biases and artefacts since many variants lacked datapoints in the narrow transition regime. [d] Fixed at 21 since no clear upper asymptote was reached as the protein was too labile.

Table S13. Kinetic parameters of the *Gs*FPPS and *Sp*FPPS variants with their native substrates in the first chain extension.[a]

PE	[PE] in assay (µg mL^{-1})		Equation used	$k_{obs,max}$ (min^{-1})	K_M (µM)	K_i (µM)	R^2
*Gs*FPPS wt	3.0	for varied [**1a-P**]	(S4)	15.63 ± 0.59	150 ± 11	-	0.997
		for varied [**2a-P**]	(S5)	132.74 ± 176.26	173 ± 248	6 ± 9	
*Gs*FPPS T80A	1.8	for varied [**1a-P**]	(S4)	11.23 ± 0.23	48 ± 3	-	0.995
		for varied [**2a-P**]	(S5)	19.57 ± 1.81	18 ± 3	98 ± 16	0.958
*Gs*FPPS T80S	3.0	for varied [**1a-P**]	(S4)	10.15 ± 0.31	74 ± 5	-	0.994
		for varied [**2a-P**]	(S5)	19.36 ± 2.28	22 ± 4	55 ± 10	0.972
*Gs*FPPS T80G	3.5	for varied [**1a-P**]	(S4)	8.82 ± 0.38	119 ± 11	-	0.994
		for varied [**2a-P**]	(S5)	37.08 ± 20.72	63 ± 43	15 ± 10	0.949
*Gs*FPPS T80R	2.5	for varied [**1a-P**]	(S4)	8.08 ± 0.13	15 ± 1	-	0.983
		for varied [**2a-P**]	(S5)	10.35 ± 0.61	29 ± 3	595 ± 144	0.989

*Gs*FPPS T80L	3.0	for varied [**1a-P**]	(S4)	6.02 ± 0.09	13 ± 1	-	0.982
		for varied [**2a-P**]	(S4)	8.89 ± 0.34	84 ± 7	-	0.992
*Gs*FPPS T80Q	8.0	for varied [**1a-P**]	(S4)	4.98 ± 0.12	15 ± 2	-	0.967
		for varied [**2a-P**]	(S5)	8.05 ± 1.16	39 ± 10	303 ± 114	0.958
*Gs*FPPS T80Y	20.0	for varied [**1a-P**]	(S4)	2.96 ± 0.06	18 ± 1	-	0.982
		for varied [**2a-P**]	(S4)	4.67 ± 0.16	93 ± 7	-	0.994
*Gs*FPPS S82A	15.0	for varied [**1a-P**]	(S4)	2.91 ± 0.10	115 ± 8	-	0.996
		for varied [**2a-P**]	(S5)	8.60 ± 1.70	51 ± 13	25 ± 6	0.984
*Gs*FPPS S82T	2.0	for varied [**1a-P**]	(S4)	10.20 ± 0.19	65 ± 3	-	0.997
		for varied [**2a-P**]	(S5)	15.75 ± 1.17	19 ± 3	86 ± 11	0.977
*Gs*FPPS S82G	10.0	for varied [**1a-P**]	(S4)	1.82 ± 0.14	209 ± 27	-	0.993
		for varied [**2a-P**]	(S5)	9.94 ± 3.73	51 ± 23	8 ± 3	0.994
*Gs*FPPS S82M[b]	14.0	for varied [**1a-P**]	(S4)	5.84 ± 0.31	235 ± 20	-	0.997
		for varied [**2a-P**]	(S5)	9.13 ± 0.89	57 ± 7	36 ± 4	0.994
*Gs*FPPS S82L	10.0	for varied [**1a-P**]	(S4)	2.34 ± 0.14	195 ± 21	-	0.995
		for varied [**2a-P**]	(S4)	1.86 ± 0.13	120 ± 17	-	0.984
*Gs*FPPS M156A	18.0	for varied [**1a-P**]	(S4)	4.62 ± 0.14	55 ± 5	-	0.992
		for varied [**2a-P**]	(S5)	57.11 ± 48.29	664 ± 590	11 ± 10	0.990
*Gs*FPPS M156N	13.0	for varied [**1a-P**]	(S4)	2.57 ± 0.17	280 ± 29	-	0.997
		for varied [**2a-P**]	(S5)	≈2	>200	<20	n.d.[c]
*Gs*FPPS M156E	5.0	for varied [**1a-P**]	(S4)	5.07 ± 0.29	152 ± 16	-	0.993
		for varied [**2a-P**]	(S5)	16.72 ± 15.63	578 ± 591	75 ± 90	0.993
*Gs*FPPS M156G	1.5	for varied [**1a-P**]	(S4)	7.46 ± 0.29	52 ± 5	-	0.985
		for varied [**2a-P**]	(S5)	9.81 ± 1.31	38 ± 8	139 ± 34	0.965
*Gs*FPPS L188A	8.0	for varied [**1a-P**]	(S4)	11.50 ± 0.34	45 ± 4	-	0.988
		for varied [**2a-P**]	(S5)	24.57 ± 7.31	71 ± 28	57 ± 23	0.944
*Gs*FPPS L188T	0.8	for varied [**1a-P**]	(S4)	15.05 ± 0.28	26 ± 2	-	0.990
		for varied [**2a-P**]	(S5)	14.80 ± 1.26	14 ± 3	380 ± 110	0.903
*Gs*FPPS L188S	1.0	for varied [**1a-P**]	(S4)	16.55 ± 0.73	41 ± 5	-	0.978
		for varied [**2a-P**]	(S5)	13.90 ± 0.52	9 ± 1	425 ± 60	0.953
*Gs*FPPS L188E	30.0	for varied [**1a-P**]	(S4)	1.11 ± 0.03	22 ± 2	-	0.971
		for varied [**2a-P**]	(S4)	2.09 ± 0.15	152 ± 20	-	0.988
*Gs*FPPS L188H	2.0	for varied [**1a-P**]	(S4)	6.76 ± 0.20	48 ± 4	-	0.990
		for varied [**2a-P**]	(S5)	8.18 ± 0.66	8 ± 2	171 ± 33	0.898
*Gs*FPPS L188M	3.5	for varied [**1a-P**]	(S4)	5.55 ± 0.19	94 ± 5	-	0.998
		for varied [**2a-P**]	(S5)	23.75 ± 3.34	30 ± 6	23 ± 4	0.992
*Sp*FPPS wt	4.0	for varied [**1a-P**]	(S4)	16.16 ± 0.24	13 ± 1	-	0.983
		for varied [**2a-P**]	(S5)	15.73 ± 0.99	6 ± 1	966 ± 430	0.768
*Sp*FPPS S89A	4.0	for varied [**1a-P**]	(S4)	19.64 ± 0.19	24 ± 1	-	0.997
		for varied [**2a-P**]	(S5)	21.83 ± 1.07	9 ± 1	394 ± 70	0.908
*Sp*FPPS S89E	40.0	for varied [**1a-P**]	(S4)	1.36 ± 0.02	24 ± 2	-	0.989
		for varied [**2a-P**]	(S4)	1.23 ± 0.02	6 ± 1	-	0.917
*Sp*FPPS S89G	4.0	for varied [**1a-P**]	(S4)	16.18 ± 0.16	26 ± 1	-	0.997
		for varied [**2a-P**]	(S5)	18.30 ± 0.87	10 ± 1	307 ± 45	0.935
*Sp*FPPS S89V	4.0	for varied [**1a-P**]	(S4)	15.60 ± 0.28	6 ± 1	-	0.925
		for varied [**2a-P**]	(S4)	15.38 ± 0.42	16 ± 2	-	0.956
*Sp*FPPS F91I	4.0	for varied [**1a-P**]	(S4)	12.01 ± 0.32	27 ± 2	-	0.978
		for varied [**2a-P**]	(S5)	12.04 ± 0.45	5 ± 1	354 ± 46	0.928
*Sp*FPPS T159S	8.0	for varied [**1a-P**]	(S4)	11.30 ± 0.22	31 ± 2	-	0.990
		for varied [**2a-P**]	(S5)	10.71 ± 0.40	8 ± 1	700 ± 137	0.935
*Sp*FPPS S197T	4.0	for varied [**1a-P**]	(S4)	11.14 ± 0.22	4 ± 1	-	0.957
		for varied [**2a-P**]	(S4)	13.20 ± 0.37	33 ± 3	-	0.983

[a] Note that the kinetic parameters of wt *Gs*FPPS and wt *Sp*FPPS differ slightly from those reported above (Table S4) using pure **1a-PP** and **2a-PP** (instead of *in situ* synthesis from the monophosphate precursors) because slightly different reaction conditions were used. In addition, a strong codependency of K_M, K_i and $k_{obs,max}$ introduced large errors for the kinetic parameter of some enzymes, typically in the experiment varying [**2a-P**]. [b] Also featuring a D29E substitution. [c] This fit featured codependency of parameters and did not converge.

Table S14. Observed rate constants of the *Gs*FPPS and *Sp*FPPS variants with various substrates.[a]

PE	[PE] / $k_{obs,max}$ (min^{-1}) at 300 µM						
	**1a-PP**	**3aa-PP**	**1b-PP**	**1c-PP**	**1d-PP**	**1e-PP**	**2b-PP**
*Gs*FPPS wt	1.3 / 12.2<br>2.5 / 12.1<br>5.0 / 6.9	2.5 / 10.3<br>5.0 / 9.1<br>10 / 5.4	62 / 0.07<br>125 / 0.03<br>250 / <0.02	62 / 0.04<br>125 / <0.02<br>250 / <0.02	62 / 0.08<br>125 / 0.04<br>250 / 0.03	62 / 0.19<br>125 / 0.22<br>250 / 0.22	62 / 0.08<br>125 / 0.03<br>250 / <0.02
*Gs*FPPS T80A	1.3 / 12.2<br>2.5 / 10.0<br>5.0 / 9.6	2.5 / 5.3<br>5.0 / 5.1<br>10 / 4.4	62 / 0.03<br>125 / <0.02<br>250 / <0.02	62 / 0.04<br>125 / <0.02<br>250 / <0.02	62 / 0.03<br>125 / 0.02<br>250 / <0.02	62 / 0.16<br>125 / 0.13<br>250 / 0.13	62 / 0.03<br>125 / <0.02<br>250 / <0.02
*Gs*FPPS T80S	1.3 / 9.4<br>2.5 / 8.8<br>5.0 / 9.3	2.5 / 5.0<br>5.0 / 5.5<br>10 / 4.1	62 / 0.04<br>125 / <0.02<br>250 / <0.02	62 / 0.05<br>125 / <0.02<br>250 / <0.02	62 / 0.04<br>125 / <0.02<br>250 / <0.02	62 / 0.18<br>125 / 0.11<br>250 / 0.15	62 / 0.02<br>125 / 0.03<br>250 / 0.02
*Gs*FPPS T80G	1.3 / 7.9<br>2.5 / 8.7<br>5.0 / 8.6	2.5 / 6.2<br>5.0 / 5.0<br>10 / 4.1	62 / 0.03<br>125 / <0.02<br>250 / <0.02	62 / 0.05<br>125 / <0.02<br>250 / <0.02	62 / 0.05<br>125 / <0.02<br>250 / <0.02	62 / 0.19<br>125 / 0.16<br>250 / 0.15	62 / 0.02<br>125 / 0.02<br>250 / <0.02
*Gs*FPPS T80R	1.3 / 10.8<br>2.5 / 6.9<br>5.0 / 5.3	2.5 / 2.7<br>5.0 / 2.8<br>10 / 2.5	62 / 0.02<br>125 / <0.02<br>250 / <0.02	62 / <0.02<br>125 / <0.02<br>250 / <0.02	62 / 0.03<br>125 / <0.02<br>250 / <0.02	62 / 0.09<br>125 / 0.07<br>250 / 0.07	62 / 0.03<br>125 / <0.02<br>250 / <0.02
*Gs*FPPS T80L	1.3 / 7.9<br>2.5 / 6.9<br>5 / 4.4	2.5 / 3.0<br>5.0 / 1.6<br>10 / 2.8	62 / 0.03<br>125 / <0.02<br>250 / <0.02	62 / 0.04<br>125 / <0.02<br>250 / <0.02	62 / 0.03<br>125 / <0.02<br>250 / <0.02	62 / <0.02<br>125 / 0.04<br>250 / 0.04	62 / 0.02<br>125 / <0.02<br>250 / <0.02
*Gs*FPPS T80Q	1.3 / 7.3<br>2.5 / 5.8<br>5.0 / 4.4	2.5 / 2.1<br>5.0 / 2.9<br>10 / 2.3	62 / 0.02<br>125 / <0.02<br>250 / <0.02	62 / 0.03<br>125 / <0.02<br>250 / <0.02	62 / 0.04<br>125 / <0.02<br>250 / <0.02	62 / 0.08<br>125 / 0.09<br>250 / 0.09	62 / 0.02<br>125 / <0.02<br>250 / <0.02
*Gs*FPPS T80Y	1.3 / 4.8<br>2.5 / 3.8<br>5.0 / 3.1	2.5 / 1.7<br>5.0 / 1.7<br>10 / 1.9	62 / 0.02<br>125 / <0.02<br>250 / <0.02	62 / 0.03<br>125 / <0.02<br>250 / <0.02	62 / <0.02<br>125 / <0.02<br>250 / <0.02	62 / 0.06<br>125 / <0.02<br>250 / 0.03	62 / <0.02<br>125 / <0.02<br>250 / <0.02
*Gs*FPPS S82A	1.3 / 3.2<br>2.5 / 2.6<br>5.0 / 2.7	2.5 / 1.4<br>5.0 / 1.8<br>10 / 1.6	62 / 0.04<br>125 / <0.02<br>250 / <0.02	62 / 0.04<br>125 / <0.02<br>250 / <0.02	62 / 0.03<br>125 / <0.02<br>250 / <0.02	62 / 0.09<br>125 / 0.07<br>250 / 0.09	62 / <0.02<br>125 / <0.02<br>250 / <0.02
*Gs*FPPS S82T	1.3 / 11.7<br>2.5 / 9.4<br>5.0 / 9.2	2.5 / 7.0<br>5.0 / 6.1<br>10 / 3.3	62 / 0.03<br>125 / <0.02<br>250 / <0.02	62 / 0.04<br>125 / <0.02<br>250 / <0.02	62 / 0.05<br>125 / 0.04<br>250 / 0.06	62 / 0.30<br>125 / 0.24<br>250 / 0.16	62 / 0.03<br>125 / <0.02<br>250 / <0.02
*Gs*FPPS S82G	1.3 / 3.0<br>2.5 / 5.7<br>5.0 / 3.2	2.5 / 2.7<br>5.0 / 2.7<br>10 / 2.3	62 / 0.08<br>125 / 0.05<br>250 / <0.02	62 / 0.03<br>125 / <0.02<br>250 / <0.02	62 / 0.05<br>125 / <0.02<br>250 / <0.02	62 / 0.04<br>125 / 0.03<br>250 / 0.05	62 / 0.02<br>125 / <0.02<br>250 / <0.02
*Gs*FPPS S82M[b]	1.3 / 4.6<br>2.5 / 5.3<br>5.0 / 3.9	5.0 / 0.1<br>10 / 0.2<br>20 / 0.2	62 / 0.05<br>125 / <0.02<br>250 / <0.02	62 / 0.03<br>125 / <0.02<br>250 / <0.02	62 / 0.04<br>125 / <0.02<br>250 / <0.02	62 / <0.02<br>125 / <0.02<br>250 / <0.02	62 / 0.02<br>125 / <0.02<br>250 / <0.02
*Gs*FPPS S82L	1.3 / 2.6<br>2.5 / 2.1<br>5.0 / 2.1	2.5 / 0.1<br>5.0 / <0.1<br>10 / 0.2	62 / 0.04<br>125 / <0.02<br>250 / <0.02	62 / 0.03<br>125 / <0.02<br>250 / <0.02	62 / <0.02<br>125 / <0.02<br>250 / <0.02	62 / 0.02<br>125 / <0.02<br>250 / <0.02	62 / <0.02<br>125 / <0.02<br>250 / <0.02
*Gs*FPPS M156A	1.3 / 6.2<br>2.5 / 6.4<br>5.0 / 6.1	2.5 / 5.0<br>5.0 / 4.5<br>10 / 4.1	62 / 0.16<br>125 / 0.11<br>250 / 0.12	62 / 0.06<br>125 / 0.03<br>250 / 0.02	62 / 0.23<br>125 / 0.20<br>250 / 0.21	62 / 0.44<br>125 / 0.41<br>250 / 0.15	62 / 0.05<br>125 / 0.02<br>250 / <0.02
*Gs*FPPS M156N	2.5 / 2.3<br>5.0 / 3.6<br>10.0 / 4.0	2.5 / 4.3<br>5.0 / 3.7<br>10 / 3.2	62 / 0.03<br>125 / <0.02<br>250 / <0.02	62 / 0.04<br>125 / <0.02<br>250 / <0.02	62 / 0.03<br>125 / <0.02<br>250 / <0.02	62 / 0.10<br>125 / 0.08<br>250 / 0.10	62 / <0.02<br>125 / <0.02<br>250 / <0.02
*Gs*FPPS M156E	1.3 / 0.3<br>2.5 / 2.5<br>5.0 / 3.3	2.5 / 3.4<br>5.0 / 2.6<br>10 / 3.5	62 / 0.04<br>125 / <0.02<br>250 / <0.02	62 / 0.04<br>125 / <0.02<br>250 / <0.02	62 / 0.02<br>125 / <0.02<br>250 / <0.02	62 / 0.05<br>125 / 0.06<br>250 / 0.08	62 / 0.02<br>125 / <0.02<br>250 / <0.02
*Gs*FPPS M156G	1.3 / 8.7<br>2.5 / 7.5<br>5.0 / 5.1	2.5 / 4.9<br>5.0 / 4.0<br>10 / 3.4	62 / 0.17<br>125 / 0.15<br>250 / 0.14	62 / 0.10<br>125 / 0.07<br>250 / 0.09	62 / 0.08<br>125 / 0.08<br>250 / 0.10	62 / 0.36<br>125 / 0.34<br>250 / 0.12	62 / <0.02<br>125 / 0.03<br>250 / <0.02
*Gs*FPPS L188A	1.3 / 11.3<br>2.5 / 8.4<br>5.0 / 9.0	2.5 / 11.1<br>5.0 / 9.7<br>10 / 6.2	62 / 0.06<br>125 / <0.02<br>250 / <0.02	62 / <0.02<br>125 / <0.02<br>250 / <0.02	62 / 0.07<br>125 / 0.04<br>250 / 0.04	62 / 0.24<br>125 / 0.27<br>250 / 0.22	62 / 0.25<br>125 / 0.18<br>250 / 0.13

	1.3 / 18.1	2.5 / 12.0	62 / 0.05	62 / <0.02	62 / 0.07	62 / 0.16	62 / 0.17
*Gs*FPPS L188T	2.5 / 14.6	5.0 / 10.2	125 / <0.02	125 / <0.02	125 / 0.03	125 / 0.18	125 / 0.13
	5.0 / 10.3	10 / 5.9	250 / <0.02	250 / <0.02	250 / 0.02	250 / 0.18	250 / 0.11
	1.3 / 16.5	2.5 / 12.8	62 / 0.06	62 / <0.02	62 / 0.06	62 / 0.25	62 / 0.19
*Gs*FPPS L188S	2.5 / 9.8	5.0 / 7.7	125 / 0.02	125 / <0.02	125 / 0.04	125 / 0.21	125 / 0.16
	5.0 / 9.4	10 / 6.2	250 / <0.02	250 / <0.02	250 / 0.03	250 / 0.20	250 / 0.12
	1.3 / 2.9	2.5 / 1.1	62 / 0.07	62 / <0.02	62 / 0.09	62 / <0.02	62 / 0.06
*Gs*FPPS L188E	2.5 / 1.4	5.0 / 1.1	125 / 0.03	125 / <0.02	125 / 0.03	125 / <0.02	125 / 0.03
	5.0 / 1.8	10 / 0.8	250 / <0.02	250 / <0.02	250 / 0.02	250 / <0.02	250 / <0.02
	1.3 / 7.9	2.5 / 6.9	62 / 0.06	62 / <0.02	62 / 0.08	62 / 0.09	62 / 0.07
*Gs*FPPS L188H	2.5 / 8.3	5.0 / 6.6	125 / 0.03	125 / <0.02	125 / 0.03	125 / 0.11	125 / 0.03
	5.0 / 7.5	10 / 5.3	250 / <0.02	250 / <0.02	250 / <0.02	250 / 0.11	250 / <0.02
	1.3 / 7.5	2.5 / 6.4	62 / 0.06	62 / <0.02	62 / 0.05	62 / 0.19	62 / 0.12
*Gs*FPPS L188M	2.5 / 7.0	5.0 / 7.0	125 / 0.03	125 / <0.02	125 / 0.04	125 / 0.21	125 / 0.07
	5.0 / 7.3	10 / 5.4	250 / <0.02	250 / <0.02	250 / 0.02	250 / 0.20	250 / 0.06
	1.3 / 29.1	2.5 / 9.8	62 / 0.22	62 / 0.12	62 / 0.06	62 / 0.33	62 / <0.02
*Sp*FPPS wt	2.5 / 30.1	5.0 / 11.8	125 / 0.08	125 / 0.09	125 / 0.06	125 / 0.36	125 / <0.02
	5.0 / 28.0	10 / 12.3	250 / 0.16	250 / 0.12	250 / 0.06	250 / 0.27	250 / <0.02
	1.3 / 43.8	1.3 / 7.9	62 / 0.18	62 / 0.08	62 / <0.02	62 / 0.35	62 / <0.02
*Sp*FPPS S89A	2.5 / 45.4	2.5 / 5.8	125 / 0.21	125 / 0.10	125 / 0.04	125 / 0.41	125 / <0.02
	5.0 / 33.0	5 / 15.6	250 / 0.19	250 / 0.10	250 / 0.07	250 / 0.30	250 / <0.02
	1.3 / 1.6	1.3 / <0.1	62 / 0.28	62 / 0.13	62 / 0.09	62 / <0.02	62 / <0.02
*Sp*FPPS S89E	2.5 / 3.2	2.5 / <0.1	125 / 0.08	125 / 0.06	125 / 0.13	125 / 0.05	125 / <0.02
	10 / 3.3	5 / <0.1	250 / 0.04	250 / 0.02	250 / <0.02	250 / 0.03	250 / <0.02
	1.3 / 29.6	1.3 / 4.8	62 / 0.17	62 / 0.15	62 / 0.11	62 / 0.32	62 / <0.02
*Sp*FPPS S89G	2.5 / 33.3	2.5 / 16.4	125 / 0.22	125 / 0.10	125 / 0.04	125 / 0.39	125 / <0.02
	5.0 / 31.0	5 / 12.9	250 / 0.17	250 / 0.13	250 / 0.08	250 / 0.29	250 / <0.02
	1.3 / 29.6	1.3 / 11.6	62 / 0.07	62 / <0.02	62 / <0.02	62 / 0.05	62 / <0.02
*Sp*FPPS S89V	2.5 / 32.2	2.5 / 14.0	125 / 0.06	125 / <0.02	125 / <0.02	125 / 0.12	125 / <0.02
	5.0 / 25.8	5 / 10.7	250 / 0.11	250 / 0.07	250 / <0.02	250 / 0.14	250 / <0.02
	1.3 / 34.9	1.3 / 0.5	62 / 0.14	62 / 0.11	62 / <0.02	62 / 0.02	62 / <0.02
*Sp*FPPS F91I	2.5 / 33.0	2.5 / 4.5	125 / 0.02	125 / 0.03	125 / <0.02	125 / <0.02	125 / <0.02
	5.0 / 25.2	5 / 4.0	250 / 0.03	250 / <0.02	250 / <0.02	250 / 0.02	250 / <0.02
	1.3 / 19.5	1.3 / 7.9	62 / 0.46	62 / 0.18	62 / 0.20	62 / 0.51	62 / <0.02
*Sp*FPPS T159S	2.5 / 15.1	2.5 / 9.0	125 / 0.39	125 / 0.15	125 / 0.17	125 / 0.51	125 / <0.02
	5.0 / 16.9	5 / 11.4	250 / 0.42	250 / 0.18	250 / 0.21	250 / 0.39	250 / <0.02
	1.3 / 26.9	1.3 / 4.8	62 / 0.11	62 / <0.02	62 / <0.02	62 / 0.03	62 / <0.02
*Sp*FPPS S197T	2.5 / 23.8	2.5 / 6.6	125 / 0.02	125 / <0.02	125 / <0.02	125 / 0.12	125 / <0.02
	5.0 / 19.0	5 / 8.7	250 / 0.07	250 / <0.02	250 / <0.02	250 / 0.14	250 / <0.02

[a] The following background absorption corrections were used: 0.006 AU min^{-1} for [PE] ≤ 20 μg mL^{-1}, 0.015 AU min^{-1} for [PE] = 20−100 μg mL^{-1}, 0.025 AU min^{-1} for [PE] = 125 μg mL^{-1}, 0.035 AU min^{-1} for [PE] = 250 μg mL^{-1} [b] Also featuring a D29E substitution.

Table S15. Selectivity of PEs with native and modified substrates.[a]

PE	Starter unit precursor	**1x-OH** (%)	**3xa-OH** (%)	**3xa-G** or **4xaa-G** (%)	**2a-OH** (y or n)[b]	Efficiency[c] (%)	Selectivity
*Gs*FPPS wt	**1a-P**	51	12	37	n	76	>99% (*E*)
	**1e-P**	<1	2	98	n	98	>99% (*E*)
*Gs*FPPS T80A	**1a-P**	50	14	36	n	72	>99% (*E*)
	**1e-P**	<1	<1	>99	y	99	>99% (*E*)
*Gs*FPPS T80S	**1a-P**	51	13	36	n	73	>99% (*E*)
	**1e-P**	<1	<1	>99	y	99	>99% (*E*)
*Gs*FPPS T80G	**1a-P**	49	12	39	n	76	>99% (*E*)
	**1e-P**	<1	<1	>99	y	99	>99% (*E*)
*Gs*FPPS T80R	**1a-P**	45	10	45	n	82	>99% (*E*)
	**1e-P**	7	<1	93	y	99	>99% (*E*)
*Gs*FPPS T80L	**1a-P**	46	10	44	n	81	>99% (*E*)
	**1e-P**	25	6	69	n	92	58:42 (*E*)/(Z)
*Gs*FPPS T80Q	**1a-P**	49	10	41	n	80	>99% (*E*)
	**1e-P**	3	3	94	y	97	>99% (*E*)
*Gs*FPPS T80Y	**1a-P**	44	12	44	n	79	>99% (*E*)
	**1e-P**	46	8	46	y	85	66:34 (*E*)/(Z)
*Gs*FPPS S82A	**1a-P**	48	11	37	n	77	>99% (*E*)
	**1e-P**	6	2	92	y	98	83:17 (*E*)/(Z)
*Gs*FPPS S82T	**1a-P**	53	11	36	n	77	>99% (*E*)
	**1e-P**	<1	2	98	y	98	>99% (*E*)
*Gs*FPPS S82G	**1a-P**	47	7	46	n	87	>99% (*E*)
	**1e-P**	27	7	66	n	90	61:39 (*E*)/(Z)
*Gs*FPPS S82M[b]	**1a-P**	<1	49	51	n	51	>99% (*E*)
	**1e-P**	79	21	<1	y	n.a.	n.a.
*Gs*FPPS S82L	**1a-P**	<1	48	52	n	52	>99% (*E*)
	**1e-P**	79	21	<1	n	n.a.	n.a.
*Gs*FPPS M156A	**1a-P**	n.d.[d]	n.d.[d]	n.d.[d]	n	n.a.	n.a.
	**1b-P**	67	<1	33	n	99	>99% (*E*)
	**1c-P**	82	18	<1	n	n.a.	n.a.
	**1d-P**	45	10	45	n	82	>99% (*E*)
	**1e-P**	<1	3	97	y	97	>99% (*E*)
*Gs*FPPS M156N	**1a-P**	53	13	34	n	72	>99% (*E*)
	**1e-P**	17	2	81	y	98	>99% (*E*)
*Gs*FPPS M156E	**1a-P**	48	15	36	n	71	>99% (*E*)
	**1e-P**	15	2	83	y	98	>99% (*E*)
*Gs*FPPS M156G	**1a-P**	n.d.[d]	n.d.[d]	n.d.[d]	n	n.a.	n.a.
	**1b-P**	57	10	33	n	77	>99% (*E*)
	**1c-P**	72	5	23	n	82	>99% (*E*)
	**1d-P**	72	<1	28	n	99	>99% (*E*)
	**1e-P**	3	3	93	n	97	>99% (*E*)
*Gs*FPPS L188A	**1a-P**	46	11	43	n	80	>99% (*E*)
	**1e-P**	<1	3	97	y	97	>99% (*E*)
*Gs*FPPS L188T	**1a-P**	53	12	35	n	74	>99% (*E*)
	**1e-P**	<1	<1	>99	n	99	72:28 (*E*)/(Z)
*Gs*FPPS L188S	**1a-P**	51	10	39	n	80	>99% (*E*)
	**1e-P**	<1	3	97	y	97	>99% (*E*)
*Gs*FPPS L188E	**1a-P**	47	6	47	n	87	>99% (*E*)

	**1e-P**	79	21	<1	n	n.a.	n.a.
*Gs*FPPS L188H	**1a-P**	52	11	37	n	77	>99% (*E*)
	**1e-P**	<1	4	96	y	96	>99% (*E*)
*Gs*FPPS L188M	**1a-P**	52	9	39	n	81	>99% (*E*)
	**1e-P**	2	<1	98	n	99	61:39 (*E*)/(Z)
*Sp*FPPS wt	**1a-P**	58	13	29	n	93	>99% (*E*)
	**1e-P**	<1	7	93	y	93	>99% (*E*)
*Sp*FPPS S89A	**1a-P**	53	15	32	n	68	>99% (*E*)
	**1e-P**	<1	3	97	y	97	>99% (*E*)
*Sp*FPPS S89E	**1a-P**	53	16	31	n	66	>99% (*E*)
	**1e-P**	20	4	76	y	95	>99% (*E*)
*Sp*FPPS S89G	**1a-P**	58	15	27	n	64	>99% (*E*)
	**1e-P**	<1	3	97	y	97	>99% (*E*)
*Sp*FPPS S89V	**1a-P**	51	13	36	n	73	>99% (*E*)
	**1e-P**	<1	3	97	y	97	>99% (*E*)
*Sp*FPPS F91I	**1a-P**	38	35	27	n	43	>99% (*E*)
	**1e-P**	13	4	83	y	95	>99% (*E*)
*Sp*FPPS T159S	**1a-P**	74	13	13	n	50	>99% (*E*)
	**1e-P**	<1	2	98	y	98	>99% (*E*)
*Sp*FPPS S197T	**1a-P**	51	13	36	n	73	>99% (*E*)
	**1e-P**	<1	3	97	y	97	>99% (*E*)

[a] The rations of the different analytes were derived from the allylic protons in the 1-position (≈4.2−4.0 ppm). Note that equal quantities of **1x-P** and **2a-P** were applied, which means that PEs performing two chain extensions will inevitably leave unreacted **1x-P** behind even if all **2a-P** is consumed. Also note that due to large differences in extraction efficiencies, the mass balances might be skewed. [b] yes or no. [c] Calculated as **3xa-G**/(**3xa-OH**+**3xa-G**). [d] This PE likely makes geranylgeranyl chains or longer analogues which are not extractable as their glycerol ethers and/or may not be converted to glycerol ethers by *Af*G_3PS.

Bioinformatic analyses

Protein structures of were predicted with ColabFold,[23] using the AlphaFold2 (AF2) default settings (model_type=auto, num_recycles=3, recycle_early_stop_tolerance=auto, relax_max_iterations=200, pairing_strategy=greedy). All predicted structures are available from the externally hosted supplementary information.[2]

Overall conservation and the distribution of the non-conserved residues (NCRs) were calculated from a representative set of head-to-tail PEs assembled from InterPro. To this end, all reviewed entries of head-to-tail PEs were assembled from the isoprenoid synthase domain superfamily (IPR008949), which included the families listed in Table S16. These 400 entries were reduced to 166 unique sequences (listed in the externally hosted supplementary information)[2] by deleting duplications, head-to-head PEs, terpene synthases/cyclases, and dual-function proteins. Alignment with Clustal Omega[24] using the EMBL-EMI webserver (https://www.ebi.ac.uk/jdispatcher/msa/clustalo) and manual inspection of active site residues not directly involved in substrate binding or catalysis then revealed four NCRs. The overall conservation (Fig. 1d) was calculated with ChimeraX[25–27] (https://www.rbvi.ucsf.edu/chimerax/) and mapped onto the AF2 model of *Gs*FPPS.

Table S16. Reviewed head-to-tail PEs from IPR008949.

Name	Number	Reviewed members
Polyprenyl synthetase-like	IPR000092	223
Heptaprenyl diphosphate synthase component II	IPR014119	2
Farnesyl pyrophosphate synthase-like	IPR039702	40
Trans-isoprenyl diphosphate synthases, bacterial-type	IPR044843	60
Trans-isoprenyl diphosphate synthases, eukaryotic-type	IPR044844	39
Prenyl diphosphate synthase	IPR053378	24
Short-chain Isoprenyl Diphosphate Synthase	IPR053491	2
Geranylgeranyl diphosphate synthase	IPR053504	2
Polyprenyl diphosphate synthase	IPR053541	1
Hexaprenyl pyrophosphate synthase	IPR053655	1
Geranylfarnesyl diphosphate synthase-like	IPR053662	1
Geranylgeranyl pyrophosphate synthase CRTE-like	IPR054848	3
(2E,6E)-farnesyl diphosphate synthase	IPR054885	1
Hexaprenyl-diphosphate synthase large subunit	IPR054985	1

Table S17. Amino acid distribuition of NCRs in representative PE set.

Amino acid[a]	NCR**I**	NCR**II**	NCR**III**	NCR**IV**
G	4	0	0	1
P	0	0	0	0
A	41	1	4	0
L	10	9	79	26
I	1	0	25	24
V	7	0	6	8
M	3	1	10	5
F	1	34	7	63
Y	0	1	0	2
W	0	0	0	0
S	8	104	0	32
T	57	16	35	5
N	30	0	0	0
Q	1	0	0	0
C	2	0	0	0
D	0	0	0	0
E	1	0	0	0
K	0	0	0	0
R	0	0	0	0
H	0	0	0	0

[a] Color code: non-polar, polar, charged

Synthetic procedures

General remarks (synthetic chemistry)

Unless noted otherwise, all syntheses were carried out at room temperature, in non-dried, non-degassed commercially available solvents. All reagents and starting materials were used without prior purification. Reactions under inert conditions were carried out under a nitrogen atmosphere with commercially available dry solvents as noted, using standard Schlenk techniques. Reactions in larger volumes were performed in septum-capped round bottom flasks of appropriate size, while smaller-scale reactions (<2 mL) were carried out in vials with a screw-cap. Reaction progress was generally monitored by thin layer chromatography (Polygram SIL G/UV254, Macherey & Nagel), using CAM stain (1 g $Ce(IV)(SO_4)_2$ and 5 g $(NH_4)_6Mo_4O_7$ in 90 mL water and 10 mL concentrated H_2SO_4) to visualize spots. Chromatographic purifications on normal phase were carried out employing flash chromatography on silica gel (silica gel 60, particle size 0.040−0.063 mm, mesh 230−440 ASTM, Fluka). Chromatographic purifications on reverse phase were carried out on C18-functionalized silica gel (C18-RP, 23%C, 1 mmol g^{-1}, particle size 0.040−0.063 mm, ThermoFisher). Unless stated otherwise, crude products were wet-loaded onto silica columns preequilibrated with the eluent. High-resolution mass spectrometry (HRMS) data was obtained on a Thermo Scientific Orbitrap Exploris 480 using electrospray ionization (ESI) in positive or negative mode. Nuclear magnetic resonance (NMR) spectra were recorded on a Bruker AVIII 600 or a Varian INOVA 400 NMR with the deuterated solvent acting as an internal deuterium lock. ^{1}H and ^{13}C NMR spectra are referenced to the residual solvent signal. ^{31}P NMR chemical shifts are stated relative to phosphoric acid in water. Data are reported as follows: chemical shift (ppm), multiplicity (s = singlet, d = doublet, t = triplet, q = quartet, m = multiplet, brs = broad singlet), coupling constant(s) (Hz), and integration. NMR data analysis was carried out in MestreNova (version 14.2) and in OriginPro (2024b version). NMR data are listed for all synthetized compounds and all raw NMR data are available for download from the externally hosted supplementary information at zenodo.org.[2]

Synthesis of substrates

The organopyrophosphates serving as substrates for the PEs were prepared either by nucleophilic substitution from the corresponding bromides or mesylates, following procedures reported by Dickschat and colleagues (e.g. and prior work) with some modifications reported previously by us[10] or by *in situ* IPK-catalyzed phosphorylation starting from the corresponding monophosphates which were obtained by condensation. It should be noted that some of the compounds reported below are volatile and/or highly retentive of solvent. As such, for some compounds it proved practically impossible to remove all solvent from a given material so that some intermediates were intentionally characterized and processed as solvates. However, unless stated otherwise, isolated yields correspond to >95% pure, solvent-free material.

Numbering of compounds in this manuscript

The numbering of compounds in this manuscript follows a retrosynthetic and functional group-centric perspective (Scheme S1): The pyrophosphate **Z-PP** was derived from the monophosphate **Z-P** or the electrophile (bromide or mesylate) **Z-Br** or **Z-OMs**, either of which was potentially obtained from the alcohol **Z-OH**. This alcohol was potentially a deprotection product of **Z-OTHP** which was derived from alkylation of **S1** or **S2**. The **1** series of compounds includes prenols and the **2** series isoprenols. The glycerol ether phosphate resulting from *Af*G_3PS-catalyzed etherification of **Z-PP** is **Z-GP** and the corresponding non-phosphorylated glycerol ether **Z-G**. Chain-extended compounds resulting from combinations of **1-PP**-type and **2-PP**-type building blocks either fall into the **3** series (one chain extension, geranyl-type) or **4** series (two chain extensions, farnesyl-type) with the lower-case letter indicating the position and order of building blocks. For instance, if the building block **1b-PP** was extended twice with **2a-PP** and the resulting product was dephosphorylated, this gives the alcohol **4baa-OH**. A single extension of **1d-PP** with **2a-PP**, etherification and dephosphorylation gives **3da-G**.

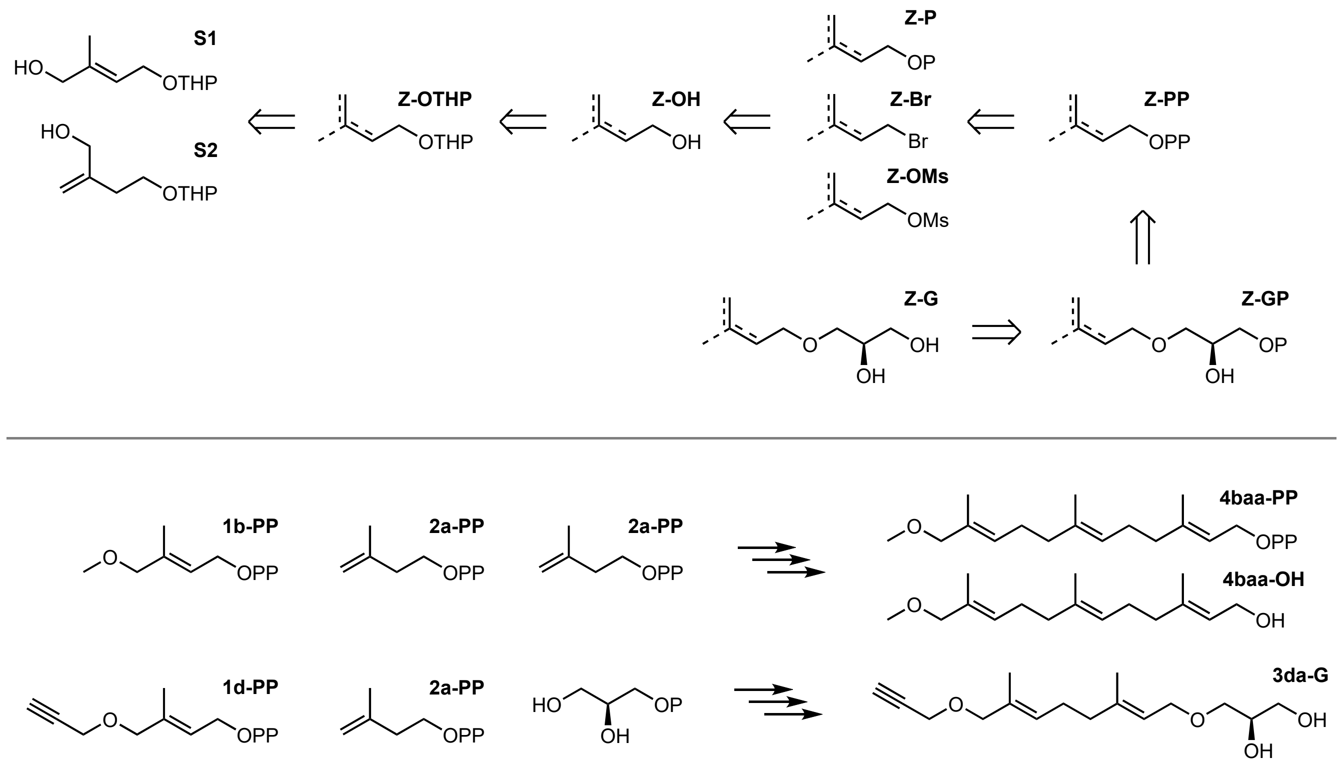

Scheme S1. Retrosynthetic analysis, compound numbering, and illustrative examples.

Notes on failed routes

We initially attempted to access the substrate analogues disclosed here (in addition to others) through a route involving the key electrophile **SX-2**. Although Riley oxidation of prenyl acetate proceeded smoothly, our attempts to activate the resulting allylic alcohol consistently resulted in decomposition. We found that mesylate **SX-2** is extremely labile as a pure compound or in solution and typically undergoes rapid isomerization, elimination and/or polymerization processes. Although the mesylate **SX-2** can be intercepted with strong nucleophiles such as azide *in situ*, the corresponding allylic azide (as well as the corresponding deprotected alcohol) is labile in solution or when purified and readily decomposes to diols of the type **SX-5**. Given the instability of these intermediates, we opted to avoid this electrophile and instead used a route that employs a branchpoint nucleophile.

We re-encountered analogous stability issues as with **SX-7** when trying to process heteroatom-bearing prenols to their pyrophosphates. When trying to activate **1b-OH** or **1c-OH** for nucleophilic pyrophosphorylation, we found that the corresponding halides or mesylates were prohibitively labile. Although activation generally proceeded in good to quantitative conversion, the resulting electrophiles readily decomposed during standard workups or concentration. We did find the corresponding chlorides (e.g. **SX-8**) to be stable enough for isolation and characterization (synthesis and partial characterization of **SX-8** are reported below) but they ultimately proved too labile to hydrolysis to be of consistent synthetic value. Pyrophosphorylation with the chloride generally yielded only 50−75% of the desired pyrophosphate (with the rest of the material being hydrolysis byproduct(s)) which was prohibitively challenging to purify and did not yield material suitable for enzyme assays. A particular challenge in this purification was the large excess of tetrabutylammonium counterion present in the mixture and the comparably high polarily of the organopyrophosphate product. The desired product generally did not retain on chromatography material and ion exchanges proved cumbersome and low-yielding. As such, we abandoned this route and instead opted for electrophilic monophosphorylation followed by enzymatic pyrophosphorylation. This route bypassed all stability issues encountered upon activation of heteroatom-bearing prenols.

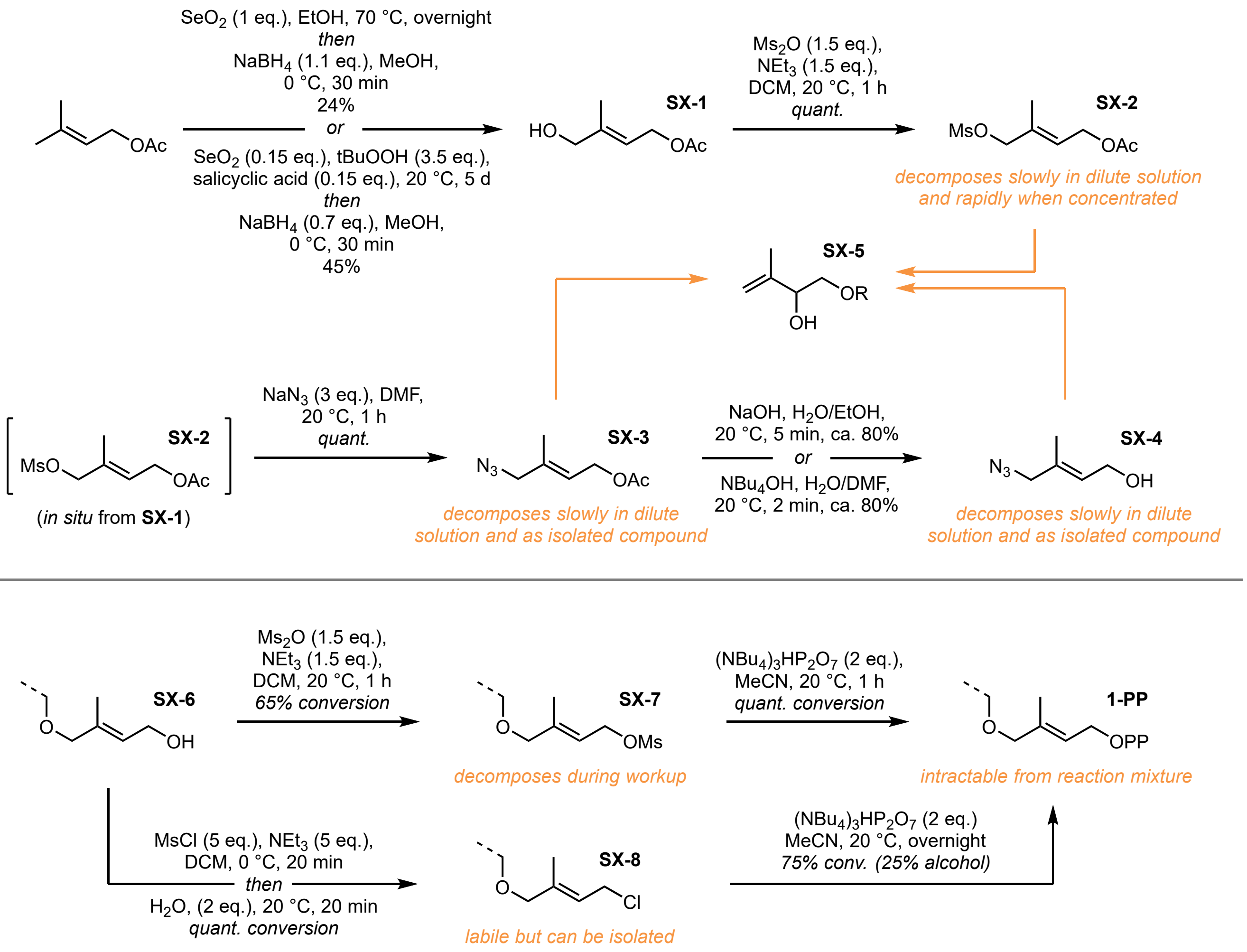

Scheme S2. Failed synthetic approaches toward heteroatom-bearing prenyl pyrophosphates.

(*E*)-1-butoxy-4-chloro-2-methylbut-2-ene (**SX-8a**)

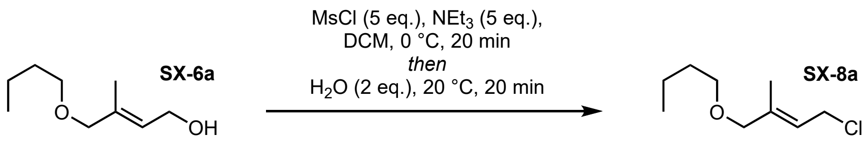

To a flame-dried 1 mL vial with a stir bar under nitrogen atmosphere, 4-butoxyprenol (**SX-6a**, prepared in analogy to the other analogues in this series, 10 mg, 12 µL, 0.063 mmol, 1.0 eq.) was added, dissolved in 200 µL DCM, and cooled to 0 °C while stirring in an ice bath. To this colorless solution were added triethylamine (28 mg, 39 µL, 0.316 mmol, 5.0 eq.) and methanesulfonylchloride (36 mg, 24 µL, 0.316 mmol, 5.0 eq.). This gave a palely yellowish suspension which was stirred while slowly warming to room temperature over 20 min. Then, water (2 mg, 2 µL, 0.127 mmol, 2.0 eq.) was added which caused rapid dissolution of the precipitate and yielded a palely yellow solution which was stirred at room temperature for 30 min. TLC analysis at this point (1:2 EtOAc/heptane, CAM) showed quantitative conversion of the starting alcohol (R_f = 0.45) and indicated the formation of a single product (R_f = 0.84). The reaction mixture was then diluted with 500 µL DCM and extracted with 200 µL sat. aq. $NaHCO_3$. The organic phase was filtered through a Na_2SO_4 plug and concentrated *in vacuo* to yield the desired chloride **SX-8a** as a yellowish oil. *Note: Although **SX-8a** is sufficiently stable for isolation and characterization, we consistently observed decomposition of similar compounds during follow-up reactions or storage.*

Analytical data for **SX-8a**:
^{1}H NMR (400 MHz, $CDCl_3$) δ 5.72−5.66 (m, 1 H), 4.12 (d, *J* = 8.3 Hz, 2 H), 3.87 (s, 2 H, m), 3.38 (t, *J* = 6.5 Hz, 2 H), 1.73 (s, 3 H), 1.60−1.52 (m, 2 H), 0.92 (t, *J* = 6.5 Hz, 3 H)

Synthesis of the native pyrophosphate substrates

Tris(tetrabutylammonium) hydrogen pyrophosphate

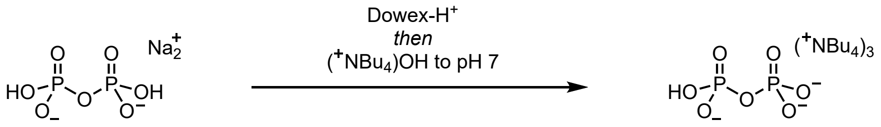

Dowex-H+ resin (ca. 15 mL, ca. 2 meq mL^{-1}, equivalent to 2 mmol protons per mL, giving 30 mmol protons total capacity) was swollen in water and loaded into a glas column. The resin was then washed with 50 mL of water until the eluent reached a neutral pH (checked with pH paper). Then, disodium pyrophosphate (2 g, 9.009 mmol, 1.0 eq.) was dissolved in 20 mL of water and passed through the Dowex column. The highly acidic eluate was collected directly into an aqueous 1.6 M (40 wt%) solution of tetrabutylammonium hydroxide (16.9 mL, 27.027 mmol, 3.0 eq.) and elution was continued until the eluate again reached a near-neutral pH (pH paper), which took ca. 1.5 CV. The resulting colorless aqueous solution was lyophilized over the weekend, yielding a brittle foamy/waxy solid. This solid was crushed into smaller pieces (ca. 2−10 mm diameter) and lypophilized overnight again. This yielded the desired salt as a crispy white solid which was stored at −20 °C. Since this compound is highly hydroscopic, it was re-freeze-dried after every other use. Following this procedure, we consistently obtained material with <3% orthophosphate impurity.

Analytical data for $(NBu_4)_3P_2O_7H$:
^{1}H NMR (400 MHz, D_2O) δ 3.06−2.97 (m, 8 H), 1.52−1.42 (m, 8 H), 1.18 (hex, *J* = 7.5 Hz, 8 H), 0.77 (t, *J* = 7.5 Hz, 12 H)
^{31}P NMR (162 MHz, D_2O) δ −8.3 (s)

Prenyl pyrophosphate (**1a-PP**) via **1a-Br → 1a-PP**

3-Methylbut-2-en-1-yl diphosphate (**1a-PP**)

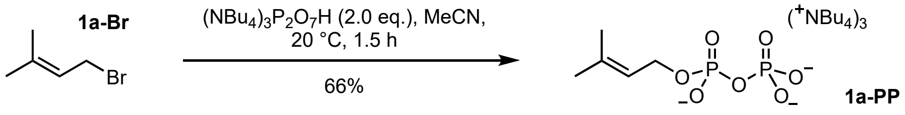

Prenyl bromide (**1a-Br**, 95%) was obtained commercially and used without purification.

To a flame-dried 4 mL vial with a stir bar under a nitrogen atmosphere, tris(tetrabutylammonium) hydrogen pyrophosphate (1.2 g, 1.342 mmol, 2.0 eq.) was added and dissolved in 1.2 mL dry MeCN. To this viscous solution, prenyl bromide (**1a-Br**, 100 mg, 78 µL, 0.671 mmol, 1.0 eq.) was added and the mixture was stirred at room temperature for 1.5 h. At this point, NMR analysis of the palely brown reaction mixture (50 µL of the reaction mixture were diluted in 600 µL CD_3CN and subjected to ^1H NMR analysis) showed complete conversion of the bromide (t, 4.53 pm for the α-protons) to the pyrophosphate (t, 4.31 ppm for the α-protons). The reaction mixture was then concentrated *in vacuo* to yield a brownish syrup. Next, this crude product was diluted with 1 mL of water and applied to a 15 g C18 column equilibrated with water. The column was washed with 40 mL of water and the desired product was eluted with 50 mL of MeCN. Concentration of the MeCN fraction *in vacuo* yielded a palely brown aq. residue which was freeze-dried overnight to provide the desired pyrophosphate **1a-PP** was a highly viscous brown oil (390 mg, 0.444 mmol and 66% yield, with an average molecular weight of 879 g mol^{-1} as the 2.6·TBA salt).

Analytical data for **1a-PP** (in agreement with our previous report[10]):
^{1}H NMR (400 MHz, CD_3CN) δ 5.38−5.33 (m, 1 H), 4.34−4.28 (m, 2 H), 3.16−3.08* (m, 21 H), 1.72−1.69 (m, 3 H), 1.71 (brs, 3 H), 1.66−1.56* (m, 22 H), 1.36* (h, *J* = 7.3 Hz, 20 H), 0.97* (t, *J* = 7.3 Hz, 29 H)
^{31}P NMR (162 MHz, CD_3CN) δ −7.9 (d, *J* = 18.8 Hz), −8.7 (d, *J* = 18.7 Hz)
*TBA

Isoprenyl pyrophosphate (**2a-PP**) via **2a-OH → 2a-OMs → 2a-PP**

3-methylbut-3-en-1-yl methanesulfonate (**2a-OMs**)

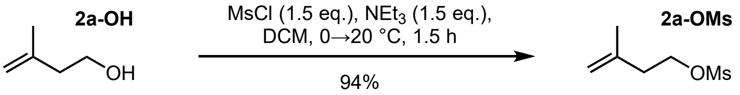

Isoprenol (**2a-OH**, 98%) was obtained commercially and used without purification. The following procedure was adapoted from our previous report.[10]

In a nitrogen-purged 10 mL round-bottom flask with a stir bar, isoprenol (**2a-OH**, 500 mg, 586 µL, 5.814 mmol, 1.0 eq.) was added, dissolved in 2 mL DCM, and cooled to 0 °C while stirring in an ice bath. To this colorless solution were added triethylamine (776 mg, 1069 µL, 8.712 mmol, 1.5 eq.) and methanesulfonylchloride (994 mg, 672 µL, 8.712 mmol, 1.5 eq.). This gave a palely yellowish suspension which was stirred while slowly warming to room temperature over 1.5 h. TLC analysis at this point (4:5 EtOAc/heptane, CAM) showed complete consumption of the starting material (R_f = 0.61) and indicated the formation of a single product (R_f = 0.69). The reaction mixture was then concentrated *in vacuo* and the resulting yellowish oil was partitioned in a mixture of 10 mL water, 10 mL sat. aq. $NaHCO_3$ and 20 mL EtOAc. The organic phase was collected, and the aqueous phase was extracted with 20 mL EtOAc again. The combined organic layers were dried over $MgSO_4$ and concentrated *in vacuo* to provide the desired mesylate **2a-OMs** as a yellowish oil (900 mg, 5.490 mmol, 94% yield) which was used without further purification or characterization.

Analytical data for **2a-OMs** (in agreement with the data reported by Fernández-Mateos *et al.*[28]):
^{1}H NMR (400 MHz, $CDCl_3$) δ 4.83 (brs, 1 H), 4.75 (brs, 1 H), 4.28 (t, *J* = 6.8 Hz, 2 H), 2.97 (s, 3 H), 2.42 (t, *J* = 6.8 Hz, 2 H), 1.73 (brs, 3 H)

3-Methylbut-3-en-1-yl diphosphate (**2a-PP**)

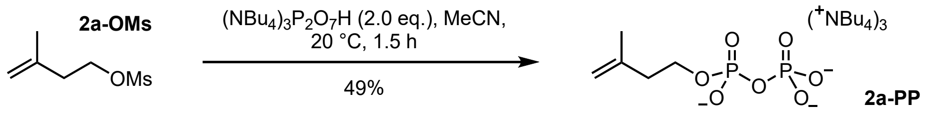

To a flame-dried 4 mL vial stir a stir bar under a nitrogen atmosphere, tris(tetrabutylammonium) hydrogen pyrophosphate (1.1 g, 1.220 mmol, 2.0 eq.) was added and dissolved in 1.2 mL dry MeCN. To this viscous solution, isoprenyl mesylate (**2a-OMs**, 100 mg, 0.610 mmol, 1.0 eq.) was added and the mixture was stirred at room temperature for 1.5 h. At this point, NMR analysis of the palely brown reaction mixture (50 µL of the reaction mixture were diluted in 600 µL CD_3CN and subjected to ^{1}H NMR analysis) showed >90% conversion of the mesylate (t, 4.29 pm for the α-protons) to the pyrophosphate (q, 3.87 ppm for the α-protons). The reaction mixture was then concentrated *in vacuo* to yield a yellowish syrup. This crude product was then diluted with 1 mL of water and applied to a 15 g C18 column equilibrated with water. The column was washed with 40 mL of water and the desired product was eluted with 50 mL of MeCN. Concentration of the MeCN fraction *in vacuo* yielded a palely yellow aqueous residue which was freeze-dried overnight to provide the desired pyrophosphate **2a-PP** was a brown oil (236 mg, 0.299 mmol and 49% yield, with an average molecular weight of 788 g mol^{-1} as the 2.3·TBA salt).

Analytical data for **2a-PP**:
^{1}H NMR (400 MHz, CD_3CN) δ 4.74 (brs, 2 H), 3.88 (q, *J* = 6.9 Hz, 2 H), 3.15−3.07* (m, 18 H), 2.28 (t, *J* = 6.9 Hz, 2 H), 1.74 (brs, 3 H), 1.66−1.56* (m, 17 H), 1.35* (h, *J* = 7.3 Hz, 17 H), 0.97* (t, *J* = 7.3 Hz, 26 H)
^{13}C NMR (101 MHz, CD_3CN) δ 144.7, 111.6, 63.7 (d), 59.3*, 39.6 (d), 24.3*, 22.9, 20.3*, 13.8*
^{31}P NMR (162 MHz, CD_3CN) δ −8.1 (d, *J* = 18.6 Hz), −8.7 (d, *J* = 18.6 Hz)
*TBA

Geranyl pyrophosphate (**3aa-PP**) via **3aa-Br → 3aa-PP**

(*E*)-3,7-dimethylocta-2,6-dien-1-yl diphosphate (**3aa-PP**)

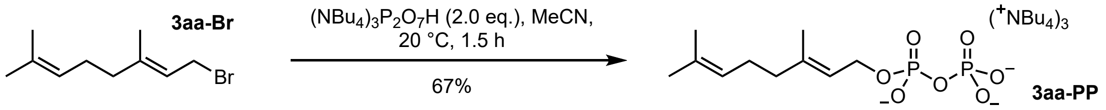

Geranyl bromide (**3aa-Br**, 95%) was obtained commercially and used without purification.

To a flame-dried 4 mL vial with a stir bar under a nitrogen atmosphere, tris(tetrabutylammonium) hydrogen pyrophosphate (831 mg, 0.922 mmol, 2.0 eq.) was added and dissolved in 1.2 mL dry MeCN. To this viscous solution, geranyl bromide (**3aa-Br**, 100 mg, 91 µL, 0.461 mmol, 1.0 eq.) was added and the mixture was stirred at room temperature for 1.5 h. At this point, NMR analysis of the yellowish reaction mixture (50 µL of the reaction mixture were diluted in 600 µL CD_3CN and subjected to ^1H NMR analysis) showed complete conversion of the bromide (t, 4.53 pm for the α-protons) to the pyrophosphate (t, 4.33 ppm for the α-protons). The reaction mixture was then concentrated *in vacuo* to yield a yellowish syrup. Next, this crude product was diluted with 1 mL of water and applied to a 15 g C18 column equilibrated with water. The column was washed with 40 mL of water and the desired product was eluted with 50 mL of MeCN. Concentration of the MeCN fraction *in vacuo* yielded a palely brown aq. residue which was freeze-dried overnight to provide the desired pyrophosphate **3aa-PP** was a highly viscous brown oil (320 mg, 0.307 mmol and 67% yield, with an average molecular weight of 1044 g mol^{-1} as the 2.9·TBA salt), containing 17% inorganic pyrophosphate as an impurity.

Analytical data for **3aa-PP** (in agreement with our previous report[10]):
^{1}H NMR (400 MHz, CD_3CN) δ 5.38−5.33 (m, 1 H), 5.15−5.09 (m, 1 H), 4.32 (t, *J* = 6.3 Hz, 2 H), 3.16−3.08* (m, 27 H), 2.14−2.06 (m, 2 H), 2.03−1.98 (m, 2 H), 1.67 (s, 3 H), 1.65 (s, 3 H), 1.64−1.56* (m, 26 H), 1.36* (h, *J* = 7.3 Hz, 23 H), 0.97* (t, *J* = 7.3 Hz, 34 H)
^{31}P NMR (162 MHz, CD_3CN) δ −7.9 (d, *J* = 18.8 Hz), −8.7 (d, *J* = 18.7 Hz)
*TBA

Farnesyl pyrophosphate (**4aaa-PP**) via **4aaa-Br → 4aaa-PP**

(2*E*,6*E*)-3,7,11-trimethyldodeca-2,6,10-trien-1-yl diphosphate (**4aaa-PP**)

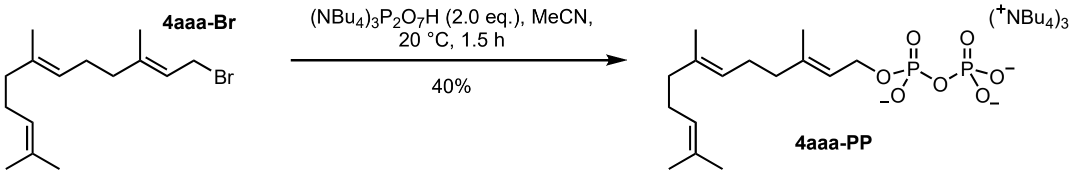

Farnesyl bromide (**4aaa-Br**, 95%) was obtained commercially and used without purification.

To a flame-dried 4 mL vial with a stir bar under a nitrogen atmosphere, tris(tetrabutylammonium) hydrogen pyrophosphate (633 mg, 0.702 mmol, 2.0 eq.) was added and dissolved in 900 µL dry MeCN. To this viscous solution, farnesyl bromide (**4aaa-Br**, 100 mg, 95 µL, 0.351 mmol, 1.0 eq.) was added and the mixture was stirred at room temperature for 1.5 h. The reaction mixture was then concentrated *in vacuo* to yield a palely yellowish syrup. Next, this crude product was diluted with 1 mL of water and applied to a 15 g C18 column equilibrated with water. The column was washed with 40 mL of water and the desired product was eluted with 50 mL of MeCN. Concentration of the MeCN fraction *in vacuo* yielded a brownish aq. residue which was freeze-dried overnight to provide the desired pyrophosphate **4aaa-PP** was a highly viscous brown oil (160 mg, 0.140 mmol and 40% yield, with an average molecular weight of 1139 g mol^{-1} as the 2.9·TBA salt), containing 17% inorganic pyrophosphate as an impurity.

Analytical data for **4aaa-PP** (in agreement with our previous report[10]):
^{1}H NMR (400 MHz, CD_3CN) δ 5.38−5.33 (m, 1 H), 5.18−5.07 (m, 2 H), 4.32 (t, *J* = 6.4 Hz, 2 H), 3.16−3.08* (m, 23 H), 2.14−1.96 (m, 8 H), 1.66 (brs, 6 H), 1.64−1.56* (m, 28 H), 1.36* (h, *J* = 7.3 Hz, 23 H), 0.97* (t, *J* = 7.3 Hz, 34 H)
^{31}P NMR (162 MHz, CD_3CN) δ −7.9 (d, *J* = 18.8 Hz), −8.7 (d, *J* = 18.6 Hz)
*TBA

Synthesis of modified monophosphate building blocks

Key branchpoint intermediates

(*E*)-2-methyl-4-((tetrahydro-2H-pyran-2-yl)oxy)but-2-en-1-ol (**S1**)

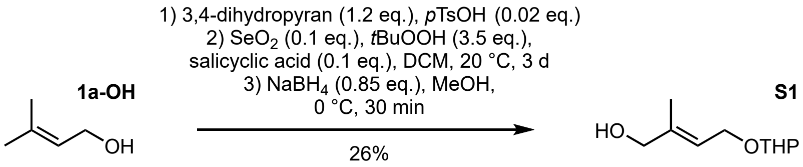

Prenol (**1a-OH**, 98%) was obtained commercially and used without purification. The following procedure was adapted from Das *et al.*[29] with modifications to enable a telescoped procedure.

A 100 mL round-bottom flask with a stir bar was charged with prenol (**1a-OH**, 11 g, 12.8 mL, 127.579 mmol, 1.0 eq.), *p*-toluenesulfonic acid (486 mg, 2.552 mmol, 0.02 eq.), and 15 mL DCM. This solution was cooled to 0 °C in an ice bath. Then, 3,4-dihydropyran (12.8 g, 14.0 mL, 153.095 mmol, 1.2 eq.) was added in 1 mL portions over 20 min. *Note: This addition is exothermic and can cause the DCM to boil if it is performed too rapidly.* The resulting brownish/greenish mixture was stirred overnight, reduced *in vacuo*, diluted in 40 mL Et_2O and washed twice with 40 mL sat. aq. $NaHCO_3$. The combined organic layers were dried over $MgSO_4$ and concentrated to yield the desired crude THP-protected prenol **1a-OTHP** as a dark brown oil. *Note: This compound has an intense and persistent citrus-like scent and we strongly recommend careful handling during the workup to avoid any spillage or contamination.* ^{1}H NMR analysis of this crude product showed it to be ca. 90% pure so that it was directly used in the subsequent Riley oxidation without further purification or characterization. *Note: This intermediate is extremely retentive of solvent. Even prolonged exposure to vacuum did not yield to Et_2O contents below 40%. However, we found that this cosolvent did not impede the subsequent oxidation. As exact quantification of this intermediate was impossible, we assumed 95% recovery during the workup and concentration as the basis for the calculation of the reagent amounts of the next transformations.* Thus, the crude **1a-OTHP** (ca. 40 mL, including the residual solvent) was added to a 250 mL round-bottom flask and dissolved in 60 mL DCM. Then, selenium dioxide (1.4 g, 12.120 mmol, 0.1 eq.), salicyclic acid (1.7 g, 12.120 mmol, 0.1 eq.), and *tert*-butylhydroperoxide (424.5 mmol, 3.5 eq., 59 mL of a 70 wt% solution in water) were added, yielding an orange biphasic mixture which was stirred at room temperature for 3 d. At this point, ^{1}H NMR analysis of the organic phase (ca. 20 µL of the reaction mixture were diluted in 700 µL $CDCl_3$ and subjected to ^{1}H NMR analysis) indicated ca. 87% conversion of the starting material (m, 5.36 ppm for the alkene proton) to a mixture of the corresponding allylic alcohol (m, 5.64 ppm for the alkene proton) and aldehyde (m, 6.61 ppm for the alkene proton and s, 9.45 ppm for the aldehyde proton). *Note: In our experience with Riley oxidations of (iso-)prenols, we found that exact reaction times, alcohol/aldehyde selectivity and conversions are unreliable. We generally recommend monitoring these transformations by ^{1}H NMR and working up once >70% conversion have been reached. Through the course of this project, we never observed any of these transformations to reach >90% conversion, even after several additional days of reaction time.* Next, Na_2SO_4 was added to the mixture under vigorous stirring and the resulting dried organic phase was filtered and concentrated *in vacuo.* The resulting orange oil was dissolved in 70 mL MeOH in a 250 mL round-bottom flask and cooled to 0 °C in an ice bath. Then, sodium borohydride (3.8 g, ca. 102 mmol, 0.85 eq.) was added in small portions over 30 min. *Note: This reduction is extremely exothermic! We strongly recommend very careful addition in a well-cooled ice bath. During this addition, the appearance of the reaction mixture typically fluctuates between a black/red suspension and a palely yellow/orange solution.* After the addition of the reductant, the mixture was stirred for an additional 3 min while warming to room temperature, giving a yellowish solution which was slowly poured into 100 mL brine. The resulting dark red and milky brine was then extracted twice with EtOAc and the combined organic layers were dried over Na_2SO_4 and concentrated *in vacuo*. ^{1}H NMR analysis indicated that the resulting crude product consisted of ca. 70% desired allylic alcohol with the rest of the material being residual starting material, isomerization product (to isoprenoids) and deprotected side products. Flash chromatography on silica gel (1:2 EtOAc/pentane → EtOAc over ca.

7 CV) provided the desired allylic alcohol **S1** (R_f = 0.26 in 1:2 EtOAc/heptane) as a palely yellowish oil as the EtOAc solvate. A subsequent solvent exchange provided **S1** as a palely yellowish oil as a 1:0.7 mixture with MeCN (6.75 g, ca. 31.5 mmol, 26% yield over the full sequence, considering the solvent content). *Note: It proved practically impossible to remove all solvent from the bulk material of **S1** (although solvent-free NMR-scale samples could be obtained). As such, we opted to process **S1** as a MeCN solvate to ensure downstream solvent compatibility. We assume that the low yield of the chromatographic purification is caused by slow deprotection of the desired product on silica gel as we consistently observed the elution of material with R_f < 0.1 from silica gel, which was absent in the crude mixture. Neutralization of silica with NEt_3 did not prevent this issue. Although partially THP-protected diols are prone to protecting group transpositions, the orientation of the double bond of **S1** prevented intramolecular transposition. We also did not observe intermolecular transposition of the purified material in MeCN over the course of this project, nor did we observe transpositions in (slightly acidic) $CDCl_3$ over several hours. In contrast, the analogous isoprenoid **S2** is much more labile (see below).*

Analytical data for **1a-OTHP** (in agreement with the data reported by Das *et al.*[3]):
^{1}H NMR (400 MHz, $CDCl_3$) δ 5.39−5.32 (m, 1 H), 4.63−4.59 (m, 1 H), 4.21 (dd, *J* = 6.7, 11.6 Hz, 1 H), 3.97 (dd, *J* = 7.6, 11.6 Hz, 1 H), 3.88 (tt, *J* = 3.1, 7.7 Hz, 1 H), 3.53−3.47 (m, 1 H), 1.87−1.78 (m, 1 H), 1.74 (s, 3 H), 1.67 (s, 3 H), 1.62−1.47 (m, 5 H)

Analytical data for **S1** (in agreement with the data reported by Das *et al.*[29]):
^{1}H NMR (400 MHz, $CDCl_3$) δ 5.67−5.61 (m, 1 H), 4.65−4.62 (m, 1 H), 4.30 (dd, *J* = 6.4, 12.0 Hz, 1 H), 4.06 (dd, *J* = 7.4, 12.2 Hz, 1 H), 4.04 (brs, 2 H), 3.88 (tt, *J* = 2.2, 8.7 Hz, 1 H), 3.56−3.48 (m, 1 H), 1.87−1.78 (m, 1 H), 1.71 (s, 3 H), 1.64−1.49 (m, 5 H)

*Note: As previously discussed in detail,[30,31] Riley oxidations are (E)-selective. Consistent with prior reports, we found our material to be isomerically pure and isomerically stable. We additionally corroborated the configuration of the double bond by NOESY analysis of the derived ethylated analogue **1c-OH** (see below).*

2-methylene-4-((tetrahydro-2H-pyran-2-yl)oxy)butan-1-ol (**S2**)

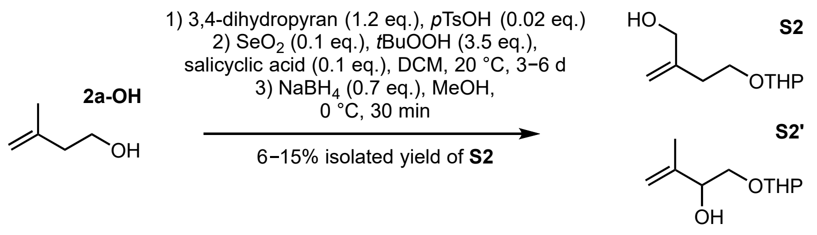

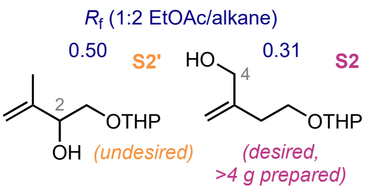

run # (scale, time)	SM	S2'	S2
1 (1.5 g, 3 d)	23%	45%	32%
2 (6 g, 4 d)	18%	49%	33%
3 (20 g, 6 d)	33%	38%	29%
4 (10 g, 4 d)	38%	40%	22%

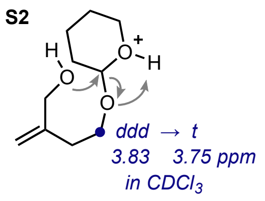

Isoprenol (**2a-OH**, 98%) was obtained commercially and used without purification. The following procedure follows in analogy to the procedure described above for **S1**. The same notes about precautions and the physical states and olfactory properties of **S1** apply to **S2**. The following synthesis was repeated in analogy several times, giving comparable yields and identical physical states and analytical data.

A 100 mL round-bottom flask with a stir bar was charged with isoprenol (**2a-OH**, 5 g, 5.89 mL, 58.14 mmol, 1.0 eq.), *p*-toluenesulfonic acid (221 mg, 1.163 mmol, 0.02 eq.), and 15 mL DCM. This solution was cooled to 0 °C in an ice bath. Then, 3,4-dihydropyran (5.86 g, 6.37 mL, 69.767 mmol, 1.2 eq.) was added in 1 mL portions over 20 min. The resulting brownish/greenish mixture was stirred overnight, reduced *in vacuo*, diluted in 20 mL Et_2O and washed twice with 20 mL sat. aq. $NaHCO_3$. The combined organic layers were dried over $MgSO_4$ and concentrated to yield the desired crude THP-protected prenol **2a-OTHP** as a dark brown oil. Next, the crude **2a-OTHP** (ca. 20 mL, including the residual Et_2O) was added to a 100 mL round-bottom flask and dissolved in 30 mL DCM. Then, selenium dioxide (644 mg, 5.8 mmol, 0.1 eq.), salicyclic acid (813 mg, 5.8 mmol, 0.1 eq.), and *tert*-butylhydroperoxide (203 mmol, 3.5 eq., 28 mL of a 70 wt% solution in water) were added, yielding an orange biphasic mixture which was stirred at room temperature for 4 d. Then, Na_2SO_4 was added to the mixture under vigorous stirring and the resulting dried organic phase was filtered and concentrated *in vacuo.* The resulting orange oil was dissolved in 30 mL MeOH in a 250 mL round-bottom flask and cooled to 0 °C in an ice bath. Then, sodium borohydride (1.5 g, ca. 40 mmol, 0.85 eq.) was added in small portions over 30 min. After the addition of the reductant, the mixture was stirred for an additional 10 min while warming to room temperature, giving a yellowish solution which was slowly poured into 50 mL brine. The resulting dark red and milky brine was then extracted twice with EtOAc and the combined organic layers were dried over Na_2SO_4 and concentrated *in vacuo*. ^{1}H NMR analysis indicated that the resulting crude product consisted of ca. 22% desired allylic primary alcohol S2 (characteristic t, 2.41 ppm in $CDCl_3$ for the allylic protons in 2-position), 38% remaining unoxidized 2a-OTHP (t, 2.32 ppm, same position) and 40% undesired allylic secondary alcohol **S2′** (d, 4.23 ppm, same position) as main components. Flash chromatography on silica gel (1:2 EtOAc/pentane → EtOAc over ca. 7 CV) provided the desired allylic alcohol **S2** (R_f = 0.31 in 1:2 EtOAc/heptane) as a palely yellowish oil as the EtOAc solvate. A subsequent solvent exchange provided **S2** as a palely yellowish oil as a 1:0.05 mixture with MeCN (872 mg, ca. 4.7 mmol, 8% yield over the full sequence, considering the solvent content). Similarly, the alcohol **S2′** (R_f = 0.50 in 1:2 EtOAc/heptane) was obtained as a palely yellow oil as a 1:0.56 mixture with MeCN (1 g, ca. 5.4 mmol, 9% yield).

*Note: We consistently found this reaction to proceed slower than the oxidation of the prenyl analogue **1a-OTHP**, although again the reaction time needed to reach sufficient conversion as well as the exact C2-/C4-oxidation selectivity proved unreliable. Both **S2** and **S2′** are extremely prone to protecting group transposition. The THP group in **S2** transpositions to the*

*1-position relatively quickly when exposed to (dilute) acid (ca. 20% transposition after 2 h in $CDCl_3$). For this reason, characterization in $CDCl_3$ (in analogy to the other synthetic intermediates) proved impractical. However, compounds **S2** and **S2'** remained isomerically stable in DMSO-d_6 and could be stored for several months in wet MeCN without any measurable protecting group transposition occuring. As both compounds also retained solvent and could not be completely dried in bulk, we opted to exchange the residual EtOAc from the workup to MeCN to ensure downstream compatibility and sufficient stability during storage.*

Analytical data for **2a-OTHP** (in agreement with the data reported by Dixon *et al.*[32]):
^{1}H NMR (400 MHz, $CDCl_3$) δ 4.76 (s, 1 H), 4.72 (s, 1 H), 4.61−4.57 (m, 1 H), 3.90−3.80 (m, 2 H), 3.53−3.45 (m, 2 H), 2.31 (t, *J* = 7.2 Hz, 2 H), 1.85−1.77 (m, 1 H), 1.75 (s, 3 H), 1.72−1.65 (m, 1 H), 1.60−1.47 (m, 4 H)

Analytical data for **S2** (Chang *et al.*[6] previously reported a synthesis of **S2** but without analytical data):
<u>In $CDCl_3$</u>
^{1}H NMR (400 MHz, $CDCl_3$) δ 5.06 (brs, 1 H), 4.93 (brs, 1 H), 4.62−4.60 (m, 1 H), 4.09 (s, 2 H), 3.88 (dt, *J* = 9.6, 6.4 Hz, 1 H), 3.83 (ddd, *J* = 11.2, 8.2, 3.2 Hz, 1 H), 3.54−3.48 (m, 2 H), 2.31 (t, *J* = 6.4 Hz, 2 H), 1.81−1.48 (m, 6 H)
<u>In DMSO-d_6</u>
^{1}H NMR (600 MHz, DMSO-d_6) δ 5.00−4.98 (m, 1 H), 4.81 (dq, *J* = 2.6, 1.6 Hz 1 H), 4.78 (t, *J* = 5.6, 1 H), 4.55 (dd, *J* = 4.4, 2.8 Hz, 1 H), 3.87 (d, *J* = 5.7 Hz, 1 H), 3.75−3.69 (m, 2 H), 3.47−3.40 (m, 2 H), 2.23 (dt, *J* = 6.6, 1.2 Hz, 2 H), 1.74−1.66 (m, 1 H), 1.62−1.56 (m, 1 H), 1.51−1.39 (m, 4 H)
^{13}C NMR (151 MHz, DMSO-d_6) δ 147.2, 109.2, 97.9, 65.5, 63.9, 61.3, 32.8, 30.3, 25.0, 19.2
HRMS (ESI, m/z) 209.11486, calc 209.11482 for $[C_{10}H_{18}O_3Na]^+$ as $[M+Na]^+$, 395.24037, calc 395.24096 for $[C_{20}H_{36}O_6Na]^+$ as $[2M+Na]^+$

Analytical data for **S2′** (racemic, both diasteromers treated as one compound):
<u>In $CDCl_3$</u>
^{1}H NMR (400 MHz, $CDCl_3$) δ 5.08−5.05 (m, 1 H), 4.91 (brs, 1 H), 4.58 (dt, *J* = 5.4, 2.8 Hz, 1 H), 4.23 (dd, *J* = 7.4, 2.5 Hz, 1 H), 3.96−3.85 (m, 1 H), 3.82 (dd, *J* = 10.5, 3.0 Hz, 0.5 H) and 3.74 (dd, *J* = 11.0, 2.7 Hz, 0.5 H), 3.56 (dd, *J* = 11.0, 8.4 Hz, 0.5 H) and 3.46 (dd, *J* = 10.4, 7.9 Hz, 0.5 H), 3.58−3.49 (m, 1 H), 1.87−1.76 (m, 2 H), 1.75 (brs, 3 H), 1.64−1.49 (m, 4 H)
<u>In DMSO-d_6</u>
^{1}H NMR (600 MHz, DMSO-d_6) δ 4.94 (brs, 1 H), 4.89 (dd, *J* = 12.5, 4.6 Hz, 1 H), 4.80 (brs, 1 H), 4.58 (t, *J* = 3.7, 0.5 H) and 4.56 (t, *J* = 3.7, 0.5 H), 3.76 (ddd, *J* = 14.5, 7.4, 3.3 Hz, 0.5 H), 3.72 (ddd, *J* = 14.5, 7.4, 3.3 Hz, 0.5 H), 3.56 (dd, *J* = 10.2, 4.9 Hz, 0.5 H) and 3.52 (dd, *J* = 10.3, 6.6 Hz, 0.5 H), 3.44−3.39 (m, 1 H), 3.36 (dd, *J* = 10.3, 4.8 Hz, 0.5 H) and 3.28 (dd, *J* = 10.2, 7.1 Hz, 0.5 H), 1.73−1.68 (m, 1 H), 1.67 (brs, 3 H), 1.63−1.56 (m, 1 H), 1.50−1.38 (m, 4 H)
^{13}C NMR (151 MHz, DMSO-d_6) δ 146.0, 145.9, 111.1, 111.1, 98.3, 97.9, 73.1, 72.7, 70.3, 70.2, 61.3, 61.2, 30.2 (x2), 25.1 (x2), 19.1, 19.1, 18.5, 18.4
HRMS (ESI, m/z) 209.11478, calc 209.11482 for $[C_{10}H_{18}O_3Na]^+$ as $[M+Na]^+$

The identity of the regioisomers **S2** and **S2′** was corroborated by 2D NMR analysis of both compounds. The data for **S2** (used in subsequent steps) are illustrated below. Both datasets are available from the externally hosted supplementary information.[2]

S2

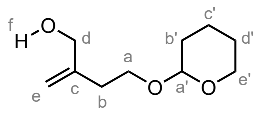

Position	δ_H (DMSO-d_6)	δ_C (DMSO-d_6)
a	ca. 3.73 / 3.45 (m)	65.5
b	2.23 (dt)	32.8
c		147.2
d	3.87 (d)	63.9
e	ca. 4.99 / 4.81 (m)	109.2
f	4.78 (t)	
a'	4.55 (dd)	97.9
b'	ca. 1.61 / 1.43 (m)	30.3
c'	ca. 1.70 / 1.48 (m)	19.2
d'	ca. 1.50 / 1.42 (m)	25.0
e'	ca. 3.74 / 3.43 (m)	61.3

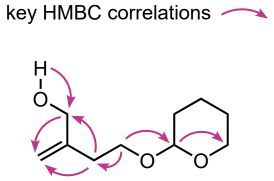

Synthesis of monophosphate substrates

Starting from **S1** or **S2**, we accessed the analogues **1b-P**−**1e-P** and **2b-P**−**2e-P** through a telescoped alkylation-deprotection sequence (*general procedure A*), followed by condensation to an orthophosphate salt (*general procedure B*). We also used *general procedure B* to make the monophosphates prenol and isoprenol.

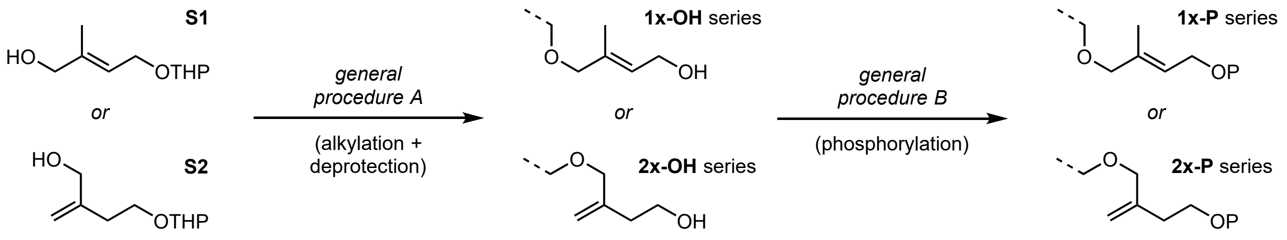

General procedure A: Alkylation & deprotection (loosely following a procedure from Wollack *et al.*[33] who reported a synthesis of **1d-PP** via **1d-OTHP**)

<u>*A1* Akylation</u>

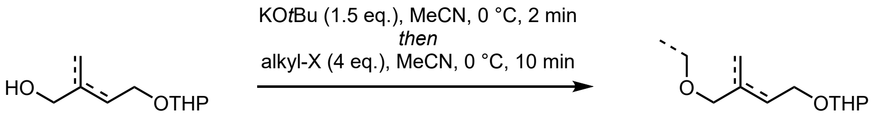

The respective alcohol **S1** or **S2** (1.0 eq., generally 0.6−1.0 g) was added to a 50 mL flask with a stir bar through a pipette filled with Na_2SO_4 and the flask was capped with a septum and flushed with nitrogen. Then, dry MeCN (to give ca. 0.2 M of the alcohol) was added. The resulting solution was cooled to 0 °C in an ice bath and potassium *tert*-butoxide (as a 1 M solution in THF) was added dropwise over 1 min. This generally yielded an orange or dark yellowish suspension, which was stirred at 0 °C for 2 min. Next, an alkyl halide (neat) was added dropwise over 1 min and the resulting dark orange flaky suspension was stirred at 0 °C for 10 min. TLC analysis at this point (1:2 heptane/EtOAc) indicated complete conversion of the starting material ($R_f \approx 0.2$−0.3) to a single product ($R_f \approx 0.7$−0.8). The reaction was then quenched by slow addition of ca. 2 mL water under stirring. The resulting orange or brownish solution was subsequently poured into 40 mL brine which was extracted two or three times with EtOAc. The combined organic layers were dried over Na_2SO_4 and concentrated *in vacuo*, generally the giving a brownish or dark yellow oil. This crude product was either purified by flash chromatography or directly subjected to THP-deprotection via *general procedure A2*. In the latter case, a small analytical sample (ca. 20 mg) of the alkylated intermediate was analyzed by NMR, either (if sufficiently pure) as a crude product or (if needed) after a pipet column. *Note: We initially purified these protected intermediates but later found that a telescoped procedure generally delivers higher yields with essentially the same purity. For telescoping, the quenched reaction mixture of general procedure A1 was directly diluted with the reagents and solvents of general procedure A2 and the deprotection was carried out as described below.*

<u>*A2* Deprotection</u>

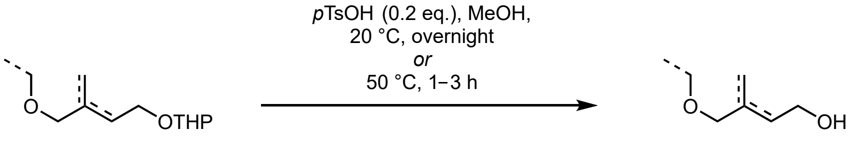

The intermediary alkylated (iso-)prenol (1.0 eq., generally 0.7−1.2 g) was added to a 50 mL flask or 20 mL vial with a stir bar and dissolved in MeOH (to give ca. 0.2 M of the alcohol). Next, *para*-toluenesulfonic acid monohydrate (0.2 eq.) was added and the resulting palely yellow solution was stirred, either at 20 °C overnight or at 50 °C for 1−3 h. TLC analysis (1:2 heptane/EtOAc or 1:1:9 EtOAc/MeOH/heptane) of the reaction mixture at this point indicated complete consumption of the starting material ($R_f \approx 0.7-0.8$) to give two product spots ($R_f \approx 0.8$ and $R_f \approx 0.2-0.3$). The reaction mixture was then poured into 40 mL sat. aq. $NaHCO_3$ which was extracted twice with EtOAc. The combined organic layers were dried over Na_2SO_4 and concentrated *in vacuo*, generally the giving a yellowish oil. The desired product ($R_f \approx 0.2-0.3$) was obtained after purification via flash chromatography (typically 1:3 EtOAc/heptane → 3:1 EtOAc/heptane), generally as a palely yellowish or palely brownish product. *Note: All these analogues tend to retain solvent and require extensive exposure to a controlled vaccum. For the more labile analogues (e.g. the methoxy analogues* ***1b-OH*** *and* ***2b-OH****) this did result in losses in yield.*

General procedure B: Monophosphorylation (loosely following a procedure from Lira *et al.*,[34] modified by us[10])

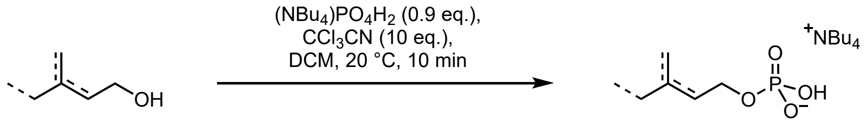

Tetrabutylammonium dihydrogen phosphate (0.9 eq.) was added to a 20 mL vial with a stir bar and dissolved in DCM (to give ca. 0.4 M of the alcohol). *Note: When dry, this salt is physically hard and essentially intractable but it can be melted easily with a heatgun to give a viscous syrup which can be scooped and smeared.* Next, the alcohol (1.0 eq., generally 80−150 mg) was added, followed by trichloroacetonitrile (10 eq.) and the resulting yellowish solution was stirred at 20 °C for 10 min. *Note: It is important to stick to this short reaction time, although it only gives 40−55% of the desired pyrophosphate. Longer reaction times under these conditions lead to an accumulation of undesired and inseparable condensation products, including diprenylphosphate or prenyltriphosphate.* The solvent was then removed *in vacuo*, yielding a yellowish syrup which was diluted with 1 mL 1:1 EtOAc/heptane and applied to a dry silica column. The column was then washed with 2 CV 1:1 EtOAc/heptane and the desired product was eluted with 2 CV MeOH. The MeOH fraction was concentrated *in vacuo* and the residue was dissolved in 1 mL MeCN, filtered and concentrated again. The residue was dissolved in MeCN once more, filtered, and concentrated again. This generally yielded the desired product as a yellowish syrup in 70−80% purity with the main impurities being diprenylphosphate (which does not impede any of the subsequent enzymatic transformations) and the diphosphate (which is already the desired product of the subsequent enzymatic phosphorylation). *Note: The desired product and these impurities can be differentiated by their charactistic NMR signals. In ^{31}P NMR (^{1}H-decoupled, in CD_3CN), the desired prenyl monophosphates generally exhibit a singlet at ca. 0.8 ppm, the diprenyl phosphate a singlet ca. 0.7 ppm and the pyrophosphate doublets around −8 ppm. In ^{1}H NMR, these species generally exhibit a multiplett (sometimes recognizable as a dt); the desired monophosphates at ca. 4.2−4.3 ppm, the diprenyl phosphate ca. 0.05 ppm lower than the monophosphate and the pyrophosphate ca. 0.1 ppm higher than the monophosphate. The isoprenyl analogues give slightly lower shifts in ^{31}P NMR (monophosphate at ca. 0.5 ppm) and ^{1}H NMR (monophosphate at ca. 3.8 ppm as a multiplett or dq) but follow the same shift trends for the byproducts. In addition, these syrups all retain solvent. To enable downstream compatibility, we opted to exchange the residual solvent to MeCN. Hence, most of our monophosphates generally contained around 1 eq. of residual MeCN. Tetrabutylammonium dihydrogen phosphate can easily be obtained by mixing phosphoric acid and tetrabutylammonium hydroxide in equal molar quantities and drying the resulting mixture in vacuo (either by lyophilization or using a rotary evaporator with a hot water bath).*

Prenyl monophosphate (**1a-P**) via **1a-OH → 1a-P**

3-Methylbut-2-en-1-yl phosphate (**1a-P**)

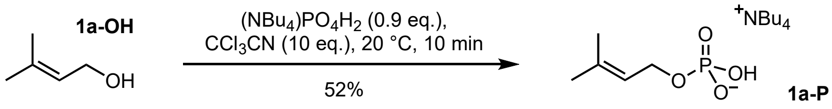

Prenol (**1a-OH**, 98%) was obtained commercially and used without purification.

Following *general procedure B*, prenol (**1a-OH**, 100 mg, 1.163 mmol, 111 µL, 1.0 eq.) was reacted with tetrabutylammonium dihydrogen phosphate (355 mg, 1.047 mmol, 0.9 eq.) and trichloroacetonitrile (1326 mg, 11.628 mmol, 921 µL, 10 eq.) in 2.9 mL DCM. Workup and purification according to the general procedure yielded the monophosphate **1a-P** as a yellow syrup (295 mg, 0.606 mmol, 52% yield as the 1.1·TBA salt with 1.2 eq. residual MeCN, 65% monophosphate content and a mean molecular weight of 487 g mol^{-1} assuming that the different phosphorylated product have the same molecular weight as the monophosphate).

Analytical data for **1a-P** (only for the monophosphate):

^{1}H NMR (600 MHz, CD_3CN) δ 5.37−5.31 (m, 1 H), 4.24−4.20 (m, 2 H), 3.16−3.06* (m, 9 H), 1.72−1.69 (m, 3 H), 1.70 (brs, 3 H), 1.65−1.56* (m, 11 H), 1.43−1.30* (m, 9 H), 1.01−0.93* (m, 13 H), *Note: Residual silica caused broadening of all signals.*

^{13}C NMR (151 MHz, CD_3CN) δ 134.2, 124.9 (d), 61.6 (d), 59.3*, 25.8, 24.4*, 20.4*, 18.1, 13.8*

^{31}P NMR (243 MHz, CD_3CN) δ 0.8 (s)

*TBA

HRMS (ESI, m/z) 165.03224, calc 165.03222 for $[C_5H_{10}O_4P]^-$ as $[M_{anion}]^-$

4-Methoxyprenyl monophosphate (**1b-P**) via **S1 → 1b-OTHP → 1b-OH → 1b-P**

(*E*)-4-Methoxy-3-methylbut-2-en-1-ol (**1b-OH**)

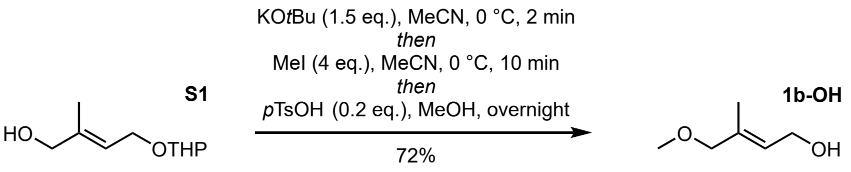

Following *general procedure A1*, intermediate **S1** (ca. 85% pure considering cosolvent content, 800 mg, 3.656 mmol, ca. 940 µL, 1.0 eq.) was reacted with potassium *tert*-butoxide (5.484 mmol, 1.5 eq., 5.48 mL of a 1 M solution in THF) and methyl iodide (2.07 g, 14.624 mmol, 911 µL, 4.0 eq.) in 18.3 mL dry MeCN. Analysis of the resulting mixture by TLC (1:2 EtOAc/heptane) showed complete consumption of **S1** (R_f = 0.30) and formation of one product spot, the intermediate **1b-OTHP** (R_f = 0.65). After quenching with 4 mL water, a small sample of the reaction mixture was worked up in analogy to the general procedure for characterization. The rest of the reaction mixture was diluted with the reagents for *general procedure A2*. To this end, **1b-OTHP** was reacted with *para*-toluenesulfonic acid monohydrate (139 mg, 0.731 mmol, 0.2 eq.) in 18.3 mL MeOH at 20 °C overnight. Analysis of the resulting mixture (1:2 EtOAc/heptane) showed complete consumption of **1b-OTHP** (R_f = 0.65) and formation of two product spots, the desired product **1b-OH** (R_f = 0.13) and the cleaved protecting group (R_f = 0.72). Workup and purification according to the general procedure yielded the alcohol **1b-OH** as a yellowish oil (305 mg, 2.629 mmol, 72% yield).

Analytical data for **1b-OTHP**:
^{1}H NMR (600 MHz, $CDCl_3$) δ 5.63−5.60 (m, 1 H), 4.63 (dd, *J* = 4.3, 3.1 Hz, 1 H), 4.29 (dd, *J* = 12.4, 6.2 Hz, 1 H), 4.08 (dd, *J* = 12.3, 7.1 Hz, 1 H), 3.88 (ddd, *J* = 11.2, 8.0, 3.0 Hz, 1 H), 3.83 (s, 2 H), 3.53−3.49 (m, 1 H), 3.30 (s, 3 H), 1.86−1.79 (m, 1 H), 1.74−1.70 (m, 1 H), 1.69 (s, 3 H), 1.62−1.49 (m, 4 H)
^{13}C NMR (151 MHz, $CDCl_3$) δ 136.3, 123.8, 98.1, 78.0, 63.4, 62.4, 57.9, 30.8, 25.6, 19.7, 14.1
HRMS (ESI, m/z) 223.13043, calc 223.13047 for $[C_{11}H_{20}O_3Na]^+$ as $[M+Na]^+$

Analytical data for **1b-OH**:
^{1}H NMR (600 MHz, $CDCl_3$) δ 5.65 (tq, *J* = 6.8, 1.4 Hz, 1 H), 4.21 (dq, *J* = 6.8, 0.8 Hz, 2 H), 3.82 (s, 2 H), 3.31 (s, 3 H), 1.69 (s, 3 H)
^{13}C NMR (151 MHz, $CDCl_3$) δ 135.8, 126.2, 77.9, 59.22, 58.0, 14.0
HRMS (ESI, m/z) compound did not ionize sufficiently well for characterization

(*E*)-4-Methoxy-3-methylbut-2-en-1-yl phosphate (**1b-P**)

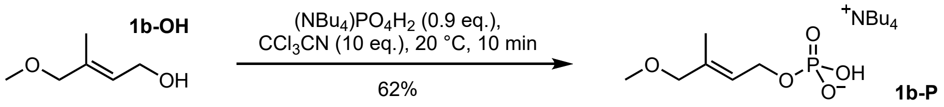

Following *general procedure B*, alcohol **1b-OH** (80 mg, 0.655 mmol, ca. 89 µL, 1.0 eq.) was reacted with tetrabutylammonium dihydrogen phosphate (200 mg, 0.590 mmol, 0.9 eq.) and trichloroacetonitrile (747 mg, 6.552 mmol, 519 µL, 10 eq.) in 1.6 mL DCM. Workup and purification according to the general procedure yielded the monophosphate **1b-P** as a yellow syrup (212 mg, 0.405 mmol, 62% yield as the 1.3·TBA salt with 0.8 eq. residual MeCN, 67% monophosphate content and a mean molecular weight of 523 g mol^{-1} assuming that the different phosphorylated product have the same molecular weight as the monophosphate).

Analytical data for **1b-P** (only for the monophosphate):

^{1}H NMR (600 MHz, CD_3CN) δ 5.60−5.56 (m, 1 H), 4.30 (t, *J* = 6.4 Hz, 2 H), 3.77 (brs, 2 H), 3.22 (s, 3 H), 3.15−3.08* (m, 11 H), 1.65−1.57* (m, 14 H), 1.35* (hex, *J* = 7.4 Hz, 11 H), 0.96* (dd, *J* = 7.4 Hz, 16 H)

^{13}C NMR (151 MHz, CD_3CN) δ 134.3, 127.7 (d), 78.6, 61.3 (d), 59.3*, 57.6, 24.4*, 20.4*, 14.1, 13.8*

^{31}P NMR (243 MHz, CD_3CN) δ 0.8 (s)

*TBA

HRMS (ESI, m/z) 195.04297, calc 195.04279 for $[C_6H_{12}O_5P]^-$ as $[M_{anion}]^-$

4-Ethoxyprenyl monophosphate (**1c-P**) via **S1 → 1c-OTHP → 1c-OH → 1c-P**

(*E*)-4-Ethoxy-3-methylbut-2-en-1-ol (**1c-OH**)

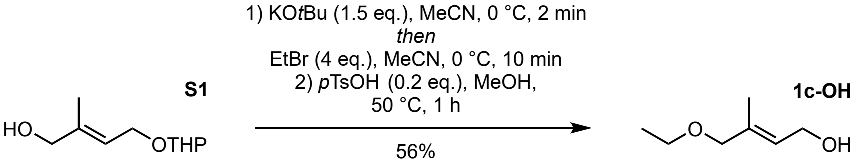

Following *general procedure A1*, intermediate **S1** (ca. 85% pure considering cosolvent content, 800 mg, 3.656 mmol, ca. 940 µL, 1.0 eq.) was reacted with potassium *tert*-butoxide (5.484 mmol, 1.5 eq., 5.48 mL of a 1 M solution in THF) and ethyl bromide (1.59 g, 14.624 mmol, 1.09 mL, 4.0 eq.) in 18.3 mL dry MeCN. Analysis of the resulting mixture by TLC (1:2 EtOAc/heptane) showed complete consumption of **S1** (R_f = 0.30) and formation of one product spot, the intermediate **1c-OTHP** (R_f = 0.70). After quenching with 2 mL water and workup according to the general procedure, a small sample of the crude product was characterized and the rest of the crude product was subjected to deprotection following *general procedure A2*. To this end, **1c-OTHP** was reacted with *para*-toluenesulfonic acid monohydrate (139 mg, 0.731 mmol, 0.2 eq.) in 18.3 mL MeOH at 50 °C for 1 h. Analysis of the resulting mixture (1:2 EtOAc/heptane) showed complete consumption of **1c-OTHP** (R_f = 0.70) and formation of two product spots, the desired product **1c-OH** (R_f = 0.27) and the cleaved protecting group (R_f = 0.72). Workup and purification according to the general procedure yielded the alcohol **1c-OH** as a palely yellowish oil (265 mg, 2.038 mmol, 56% yield).

Analytical data for **1c-OTHP**:
^{1}H NMR (600 MHz, $CDCl_3$) δ 5.62 (dddd, *J* = 7.5, 6.2, 2.7, 1.4 Hz, 1 H), 4.63 (dd, *J* = 4.3, 3.0 Hz, 1 H), 4.29 (dq, *J* = 12.2, 6.2, 1.0 Hz, 1 H), 4.07 (ddq, *J* = 12.3, 7.1, 0.7 Hz, 1 H), 3.90−3.88 (m, 1 H), 3.87 (s, 2 H), 3.53−3.49 (m, 1 H), 3.44 (q, *J* = 7.0 Hz, 2 H), 1.87−1.79 (m, 1 H), 1.74−1.70 (m, 1 H), 1.69 (s, 3 H), 1.62−1.49 (m, 4 H)
^{13}C NMR (151 MHz, $CDCl_3$) δ 136.7, 123.4, 98.1, 76.1, 65.5, 63.4, 62.4, 30.8, 25.6, 19.7, 15.3, 14.2
HRMS (ESI, m/z) 237.14596, calc 237.14612 for $[C_{12}H_{22}O_3Na]^+$ as $[M+Na]^+$

Analytical data for **1c-OH**:
^{1}H NMR (600 MHz, $CDCl_3$) δ 5.66 (tq, *J* = 6.8, 1.4 Hz, 1 H), 4.21 (d, *J* = 6.7 Hz, 2 H), 3.86 (s, 2 H), 3.46 (q, *J* = 7.0 Hz, 2 H), 1.70 (s, 3 H), 1.21 (t, *J* = 7.0 Hz, 3 H)
^{13}C NMR (151 MHz, $CDCl_3$) δ 136.1, 125.6, 75.8, 65.5, 59.1, 15.2, 14.0
HRMS (ESI, m/z) compound did not ionize sufficiently well for characterization
The (*E*)-configuration of **1c-OH** was additionally confirmed by NOESY analysis. Key NOESY signals included correlations between the free methyl group (1.70 ppm) and the allylic protons neighboring the free alcohol (4.21 ppm) as well as between the alkene proton (5.66 ppm) and the allylic protons neighboring the ether (3.86 ppm)

(*E*)-4-Ethoxy-3-methylbut-2-en-1-yl phosphate (**1c-P**)

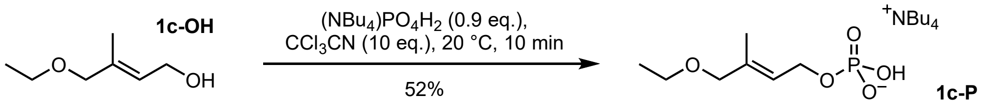

Following *general procedure B*, alcohol **1c-OH** (90 mg, 0.658 mmol, ca. 100 µL, 1.0 eq.) was reacted with tetrabutylammonium dihydrogen phosphate (201 mg, 0.592 mmol, 0.9 eq.) and trichloroacetonitrile (750 mg, 6.577 mmol, 521 µL, 10 eq.) in 1.6 mL DCM. Workup and purification according to the general procedure yielded the monophosphate **1c-P** as a yellowish syrup (206 mg, 0.342 mmol, 52% yield as the 1.4·TBA salt with 0.9 eq. residual MeCN, 74% monophosphate content and a mean molecular weight of 602 g mol^{-1} assuming that the different phosphorylated product have the same molecular weight as the monophosphate).

Analytical data for **1c-P** (only for the monophosphate):

^{1}H NMR (600 MHz, CD_3CN) δ 5.60−5.55 (m, 1 H), 4.29 (t, *J* = 6.4 Hz, 2 H), 3.81 (brs, 2 H), 3.39 (q, *J* = 6.6 Hz, 2 H), 3.15−3.08* (m, 12 H), 1.65−1.57* (m, 15 H), 1.35* (hex, *J* = 7.4 Hz, 12 H), 1.13 (t, *J* = 7.0 Hz, 2 H), 0.96* (dd, *J* = 7.4 Hz, 16 H)

^{13}C NMR (151 MHz, CD_3CN) δ 134.7, 127.1 (d), 76.5, 65.5, 61.2 (d), 59.3*, 57.6, 24.4*, 20.3*, 15.5, 14.2, 13.8*

^{31}P NMR (243 MHz, CD_3CN) δ 0.8 (s)

*TBA

HRMS (ESI, m/z) 209.05846, calc 209.05844 for $[C_7H_{14}O_5P]^-$ as $[M_{anion}]^-$

4-Propargoxyprenyl monophosphate (**1d-P**) via **S1 → 1d-OTHP → 1d-OH → 1d-P**

(*E*)-3-methyl-4-(prop-2-yn-1-yloxy)but-2-en-1-ol (**1d-OH**)

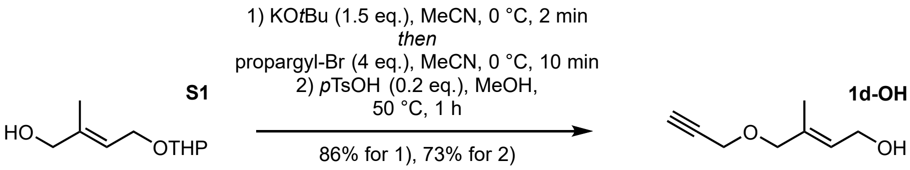

Following *general procedure A1*, intermediate **S1** (ca. 60% pure considering cosolvent content, 1.0 g, 3.226 mmol, ca. 1.18 mL, 1.0 eq.) was reacted with potassium *tert*-butoxide (4.839 mmol, 1.5 eq., 4.84 mL of a 1 M solution in THF) and propargyl bromide (12.903 mmol, 4.0 eq., 1.4 mL of an 80% solution in toluene stabilized with MgO) in 16.1 mL dry MeCN. *Note: Due to the MgO stabilizer, this reaction mixture was black.* Analysis of the resulting mixture by TLC (1:2 EtOAc/heptane) showed complete consumption of **S1** (R_f = 0.30) and formation of one product spot, the intermediate **1d-OTHP** (R_f = 0.70). After quenching with 2 mL water and workup according to the general procedure, **1d-OTHP** was purified by flash chromatography, yielding the intermediate as a golden oil (773 mg, 2.761 mmol, 86% corrected yield, retaining 20% EtOAc). Next, following *general procedure A2*, **1d-OTHP** (80% pure, 700 mg, 2.500 mmol) was reacted with *para*-toluenesulfonic acid monohydrate (95 mg, 0.500 mmol, 0.2 eq.) in 12.5 mL MeOH at 50 °C for 1 h. Analysis of the resulting mixture (1:2 EtOAc/heptane) showed complete consumption of **1d-OTHP** (R_f = 0.70) and formation of two product spots, the desired product **1d-OH** (R_f = 0.30) and the cleaved protecting group (R_f = 0.72). Workup and purification according to the general procedure yielded the alcohol **1d-OH** as a palely yellowish oil (342 mg, 1.830 mmol, 73% corrected yield, retaining 30% EtOAc).

Analytical data for **1d-OTHP** (in agreement with the data reported by Wollack *et al.*[33]):
^{1}H NMR (400 MHz, $CDCl_3$) δ 5.69−5.63 (m, 1 H), 4.64−4.62 (m, 1 H), 4.29 (dq, *J* = 12.4, 6.1, 1.0 Hz, 1 H), 4.11 (s, 3 H), 4.07 (dd, *J* = 12.3, 7.1, Hz, 1 H), 3.98 (s, 2 H), 3.88 (ddd, *J* = 11.1, 7.8, 3.3 Hz, 1 H), 3.55−3.48 (m, 1 H), 2.42 (t, *J* = 2.5 Hz, 1 H), 1.88−1.78 (m, 1 H), 1.76−1.72 (m, 1 H), 1.71 (s, 3 H), 1.63−1.49 (m, 4 H)

Analytical data for **1d-OH** (in agreement with the data reported by Wollack *et al.*[33]):
^{1}H NMR (400 MHz, $CDCl_3$) δ 5.70 (tq, *J* = 6.7, 1.4 Hz, 1 H), 4.22 (dq, *J* = 6.6, 0.8 Hz, 2 H), 4.12 (d, *J* = 2.4 Hz, 2 H), 3.97 (s, 1 H), 2.43 (t, *J* = 2.4 Hz, 1 H), 1.71 (s, 3 H)

(*E*)-3-methyl-4-(prop-2-yn-1-yloxy)but-2-en-1-yl phosphate (**1d-P**)

1d-OH

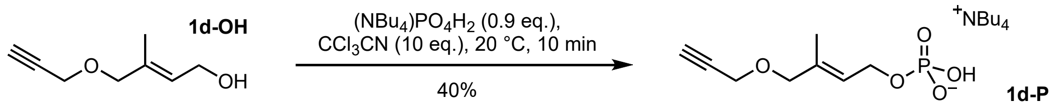

1d-P

Following *general procedure B*, alcohol **1d-OH** (75% pure, 122 mg, 0.654 mmol, ca. 136 µL, 1.0 eq.) was reacted with tetrabutylammonium dihydrogen phosphate (199 mg, 0.588 mmol, 0.9 eq.) and trichloroacetonitrile (745 mg, 6.536 mmol, 517 µL, 10 eq.) in 1.6 mL DCM. Workup and purification according to the general procedure yielded the monophosphate **1d-P** as a yellowish syrup (168 mg, 0.261 mmol, 40% yield as the 1.3·TBA salt with 2.4 eq. residual MeCN, 88% monophosphate content and a mean molecular weight of 644 g mol^{-1} assuming that the different phosphorylated product have the same molecular weight as the monophosphate).

Analytical data for **1d-P** (only for the monophosphate):

^{1}H NMR (600 MHz, CD_3CN) δ 5.63−5.59 (m, 1 H), 4.29 (dd, *J* = 6.4, 6.4 Hz, 2 H), 4.06 (d, *J* = 2.4 Hz, 2 H), 3.90 (brs, 2 H), 3.14−3.08* (m, 11 H), 2.69 (t, *J* = 2.4, 2 H), 1.64−1.57* (m, 14 H), 1.35* (hex, *J* = 7.4 Hz, 11 H), 0.96* (t, *J* = 7.4 Hz, 15 H)

^{13}C NMR (151 MHz, CD_3CN) δ 133.5, 128.6 (d), 81.1, 75.8, 75.4, 61.2 (d), 59.3*, 57.1, 24.3*, 20.3*, 14.3, 13.8*

^{31}P NMR (243 MHz, CD_3CN) δ 0.8 (s)

*TBA

HRMS (ESI, m/z) 219.04269, calc 219.04279 for $[C_8H_{12}O_5P]^-$ as $[M_{anion}]^-$

4-Benzoxyprenyl monophosphate (**1e-P**) via **S1 → 1e-OTHP → 1e-OH → 1e-P**

(*E*)-4-(benzyloxy)-3-methylbut-2-en-1-ol (**1e-OH**)

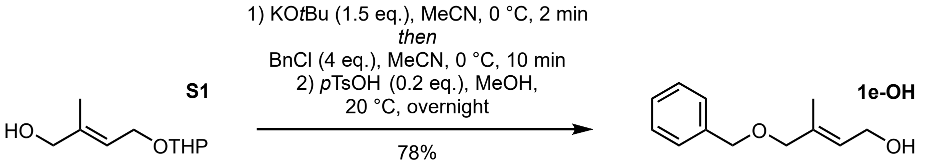

Following *general procedure A1*, intermediate **S1** (ca. 85% pure considering cosolvent content, 800 mg, 3.656 mmol, ca. 889 µL, 1.0 eq.) was reacted with potassium *tert*-butoxide (5.484 mmol, 1.5 eq., 5.48 mL of a 1 M solution in THF) and benzyl chloride (1.74 g, 14.626 mmol, 4.0 eq., 1.58 mL) in 18.2 mL dry MeCN. Analysis of the resulting mixture by TLC (1:2 EtOAc/heptane) showed complete consumption of **S1** (R_f = 0.30) and formation of one product spot, the intermediate **1e-OTHP** (R_f = 0.79). After quenching with 2 mL water and workup according to the general procedure, a small amount of **1e-OTHP** (ca. 15 mg) was purified through a pipet column (1:10 EtOAc/heptane, R_f = 0.39) yielding the intermediate as a yellow oil. The rest of the crude material was subjected to deprotection following *general procedure A2*. To this end, crude **1e-OTHP** was reacted with *para*-toluenesulfonic acid monohydrate (139 mg, 0.731 mmol, 0.2 eq.) in 18.2 mL MeOH at 20 °C overnight. Analysis of the resulting mixture (1:1:9 EtOAc/MeOH/heptane) showed complete consumption of **1e-OTHP** (R_f = 0.57) and formation of two product spots, the desired product **1e-OH** (R_f = 0.24) and the cleaved protecting group (R_f = 0.85). Workup and purification according to the general procedure yielded the alcohol **1e-OH** as a palely yellowish oil (546 mg, 2.844 mmol, 78% yield).

Analytical data for **1e-OTHP**:

^{1}H NMR (600 MHz, $CDCl_3$) δ 7.35−7.27 (m, 5 H), 5.67 (ddq, *J* = 7.4, 6.1, 1.3 Hz, 1 H), 4.64 (dd, *J* = 4.4, 3.0 Hz, 1 H), 4.48 (s, 2 H), 4.31 (ddq, *J* = 12.4, 6.3, 1.0 Hz, 1 H), 4.10 (ddq, *J* = 12.3, 7.1, 0.5 Hz, 1 H), 3.94 (s, 2 H), 3.89 (ddd, *J* = 11.2, 8.0, 3.0 Hz, 1 H), 3.54−3.50 (m, 1 H), 1.87−1.79 (m, 1 H), 1.74−1.70 (m, 4 H), 1.62−1.50 (m, 4 H)

^{13}C NMR (151 MHz, CD_3CN) δ 138.6, 136.4, 128.5 (x2), 127.9 (x2), 127.7, 124.0, 98.2, 75.7, 72.0, 63.5, 62.4, 30.8, 25.6, 19.7, 14.3

HRMS (ESI, m/z) 299.16161, calc 299.16177 for $[C_{17}H_{24}O_3Na]^+$ as $[M+Na]^+$

Analytical data for **1e-OH**:

^{1}H NMR (600 MHz, $CDCl_3$) δ 7.35−7.27 (m, 5 H), 5.66 (tq, *J* = 6.7, 1.4 Hz, 1 H), 4.49 (s, 2 H), 4.23 (d, *J* = 6.7, 1 H), 3.93 (s, 2 H), 1.73 (s, 3 H),

^{13}C NMR (151 MHz, CD_3CN) δ 138.5, 135.9, 128.5 (x2), 127.9 (x2), 126.2, 75.5, 72.2, 59.3, 14.2

HRMS (ESI, m/z) compound did not ionize sufficiently well for characterization

(*E*)-4-(benzyloxy)-3-methylbut-2-en-1-yl phosphate (**1e-P**)

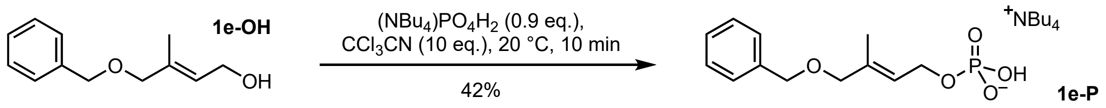

Following *general procedure B*, alcohol **1e-OH** (132 mg, 0.653 mmol, ca. 147 µL, 1.0 eq.) was reacted with tetrabutylammonium dihydrogen phosphate (199 mg, 0.588 mmol, 0.9 eq.) and trichloroacetonitrile (745 mg, 6.536 mmol, 517 µL, 10 eq.) in 1.6 mL DCM. Workup and purification according to the general procedure yielded the monophosphate **1e-P** as a yellowish syrup (187 mg, 0.276 mmol, 42% yield as the 1.5·TBA salt with 1.1 eq. residual MeCN, 85% monophosphate content and a mean molecular weight of 677 g mol^{-1} assuming that the different phosphorylated product have the same molecular weight as the monophosphate).

Analytical data for **1e-P** (only for the monophosphate):

^{1}H NMR (600 MHz, CD_3CN) δ 7.35−7.25 (m, 5 H), 5.63 (tq, *J* = 6.3, 1.3 Hz, 2 H), 4.42 (s, 2 H), 4.32 (tq, *J* = 6.3, 1.0 Hz, 2 H), 3.89 (brs, 2 H), 3.14−3.08* (m, 11 H), 1.66 (brs, 3 H), 1.64−1.57* (m, 12 H), 1.35* (hex, *J* = 7.4 Hz, 12 H), 0.96* (t, *J* = 7.4 Hz, 17 H)

^{13}C NMR (151 MHz, CD_3CN) δ 140.0, 134.3, 129.2 (x2), 128.6 (x2), 128.4, 127.8 (d), 76.4, 72.1, 61.2 (d), 59.3*, 24.3*, 20.3*, 14.3, 13.8*

^{31}P NMR (243 MHz, CD_3CN) δ 0.8 (s)

*TBA

HRMS (ESI, m/z) 271.07386, calc 271.07408 for $[C_{12}H_{16}O_5P]^-$ as $[M_{anion}]^-$

Isoprenyl monophosphate (**2a-P**) via **2a-OH → 2a-P**

3-Methylbut-3-en-1-yl phosphate (**2a-P**)

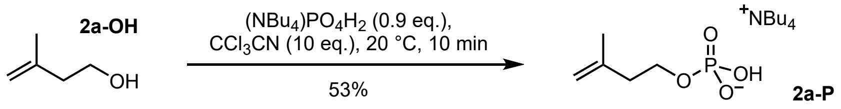

Isoprenol (**2a-OH**, 98%) was obtained commercially and used without purification.

Following *general procedure B*, isoprenol (**2a-OH**, 100 mg, 1.163 mmol, 111 µL, 1.0 eq.) was reacted with tetrabutylammonium dihydrogen phosphate (355 mg, 1.047 mmol, 0.9 eq.) and trichloroacetonitrile (1326 mg, 11.628 mmol, 921 µL, 10 eq.) in 2.9 mL DCM. Workup and purification according to the general procedure yielded the monophosphate **2a-P** as a yellowish syrup (312 mg, 0.612 mmol, 53% yield as the 1.2·TBA salt with 1.2 eq. residual MeCN, 85% monophosphate content and a mean molecular weight of 510 g mol^{-1} assuming that the different phosphorylated product have the same molecular weight as the monophosphate).

Analytical data for **2a-P** (only for the monophosphate):
^{1}H NMR (600 MHz, CD_3CN) δ 4.73 (s, 2 H), 3.79 (q, *J* = 6.8 Hz, 2 H), 3.15−3.08* (m, 10 H), 2.26 (t, *J* = 7.1 Hz, 2 H), 1.74 (s, 3 H), 1.64−1.56* (m, 10), 1.35* (hex, *J* = 7.4 Hz, 10 H), 0.96* (t, *J* = 7.3 Hz, 15 H)
^{13}C NMR (151 MHz, CD_3CN) δ 144.9, 111.42, 63.0 (d), 59.22, 59.2*, 40.0 (d), 24.3*, 22.9, 20.3*, 13.7*
^{31}P NMR (243 MHz, CD_3CN) δ 0.5 (s)
*TBA

4-Methoxyisoprenyl monophosphate (**2b-P**) via **S2 → 2b-OTHP → 2b-OH → 2b-P**

3-(Methoxymethyl)but-3-en-1-ol (**2b-OH**)

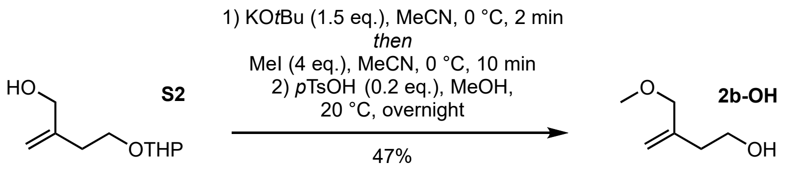

Following *general procedure A1*, intermediate **S2** (ca. 83% pure considering cosolvent content, 800 mg, 3.570 mmol, ca. 889 µL, 1.0 eq.) was reacted with potassium *tert*-butoxide (5.355, 1.5 eq., 5.36 mL of a 1 M solution in THF) and methyl iodide (2.03 g, 14.280 mmol, 889 µL, 4.0 eq.) in 17.8 mL dry MeCN. Analysis of the resulting mixture by TLC (1:2 EtOAc/heptane) showed complete consumption of **S2** (R_f = 0.27) and formation of one product spot, the intermediate **2b-OTHP** (R_f = 0.63). After quenching with 2 mL water and workup according to the general procedure, a small sample of the crude product (ca. 10 mg) was characterized and the rest of the crude product was subjected to deprotection following *general procedure A2*. To this end, crude **2b-OTHP** was reacted with *para*-toluenesulfonic acid monohydrate (136 mg, 0.714 mmol, 0.2 eq.) in 17.8 mL MeOH at 20 °C overnight. Analysis of the resulting mixture (1:1:9 EtOAc/MeOH/heptane) showed complete consumption of **2b-OTHP** (R_f = 0.59) and formation of two product spots, the desired product **2b-OH** (R_f = 0.24) and the cleaved protecting group (R_f = 0.85). Workup and purification according to the general procedure yielded the alcohol **1b-OH** as a yellowish oil (194 mg, 1.672 mmol, 47% yield). *Note: Alcohol **2b-OH** is volatile. This procedure provided **2b-OH** along with ca. 8% of an (E)/(Z)-mixture of the internal alkene isomer **1b-OH** as an inseparable byproduct.*

Analytical data for **1b-OTHP**:

^{1}H NMR (600 MHz, $CDCl_3$) δ 5.60 (s, 1 H), 4.98 (s, 1 H), 4.60 (dd, *J* = 4.4, 3.0 Hz, 1 H), 4.29 (dd, *J* = 12.4, 6.2 Hz, 1 H), 3.89 (s, 2 H), 3.88−3.84 (m, 2 H), 3.55−3.48 (m, 2 H), 3.32 (s, 3 H), 2.36 (td, *J* = 7.1, 1.3 Hz, 2 H), 1.85−1.77 (m, 1 H), 1.73−1.67 (m, 1 H), 1.60−1.49 (m, 4 H)

^{13}C NMR (151 MHz, $CDCl_3$) δ 143.4, 113.7, 98.9, 75.9, 66.3, 62.5, 58.0, 33.4, 30.9, 25.6, 19.7

HRMS (ESI, m/z) 223.13052, calc 223.13047 for $[C_{11}H_{20}O_3Na]^+$ as $[M+Na]^+$

Analytical data for **1b-OH**:

^{1}H NMR (600 MHz, $CDCl_3$) δ 5.12 (s, 1 H), 5.04 (s, 1 H), 3.89 (s, 2 H), 3.72 (t, *J* = 6.1 Hz, 1 H), 3.35 (s, 3 H), 2.36 (t, *J* = 6.0 Hz, 2 H)

^{13}C NMR (151 MHz, $CDCl_3$) δ 143.3, 115.8, 76.1, 61.6, 58.1, 37.6

HRMS (ESI, m/z) 139.07281, calc 139.07295 for $[C_6H_{12}O_2Na]^+$ as $[M+Na]^+$

3-(Methoxymethyl)but-3-en-1-yl phosphate (**2b-P**)

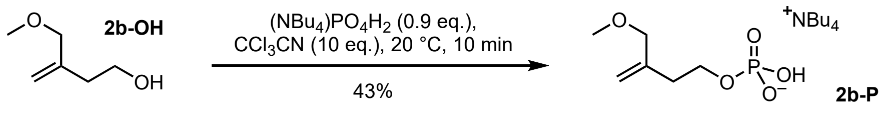

Following *general procedure B*, alcohol **2b-OH** (84 mg, 0.652 mmol, ca. 93 µL, 1.0 eq.) was reacted with tetrabutylammonium dihydrogen phosphate (199 mg, 0.587 mmol, 0.9 eq.) and trichloroacetonitrile (743 mg, 6.517 mmol, 516 µL, 10 eq.) in 1.6 mL DCM. Workup and purification according to the general procedure yielded the monophosphate **2b-P** as a brownish syrup (172 mg, 0.281 mmol, 43% yield as the 1.5·TBA salt with 1.2 eq. residual MeCN, 75% monophosphate content and a mean molecular weight of 613 g mol^{-1} assuming that the different phosphorylated product have the same molecular weight as the monophosphate). *Note: Since the precursor **2b-OH** contained ca. 8% (E)- and (Z)-**1b-OH** as inseparable impurities, this procedure also yielded the corresponding phosphates (E)/(Z)-**1b-P** (ca. 7%).*

Analytical data for **2b-P** (only for the monophosphate):

^{1}H NMR (600 MHz, CD_3CN) δ 4.99 (brs, 1 H), 4.96 (brs, 1 H), 3.85 (brs, 2 H), 3.81 (q, *J* = 6.9 Hz, 2 H), 3.26 (s, 3 H), 3.13−3.09* (m, 12 H), 2.28 (t, *J* = 7.1 Hz, 2 H), 1.64−1.57* (m, 12 H), 1.35* (hex, *J* = 7.4 Hz, 12 H), 0.96* (t, *J* = 7.4 Hz, 18 H)

^{13}C NMR (151 MHz, CD_3CN) δ 145.2, 112.4, 76.0, 63.3 (d), 59.3*, 58.1, 35.5 (d), 24.3*, 20.3*, 13.8*

^{31}P NMR (243 MHz, CD_3CN) δ 0.4 (s)

*TBA

HRMS (ESI, m/z) 195.04277, calc 195.04279 for $[C_6H_{12}O_5P]^-$ as $[M_{anion}]^-$

4-Ethoxyisoprenyl monophosphate (**2c-P**) via **S2 → 2c-OTHP → 2c-OH → 2c-P**

3-(Ethoxymethyl)but-3-en-1-ol (**2c-OH**)

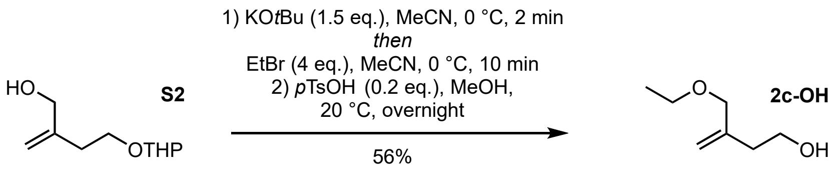

Following *general procedure A1*, intermediate **S2** (ca. 83% pure considering cosolvent content, 800 mg, 3.570 mmol, ca. 889 µL, 1.0 eq.) was reacted with potassium *tert*-butoxide (5.355 mmol, 1.5 eq., 5.36 mL of a 1 M solution in THF) and ethyl bromide (1.56 g, 14.624 mmol, 1.07 mL, 4.0 eq.) in 17.8 mL dry MeCN. Analysis of the resulting mixture by TLC (1:2 EtOAc/heptane) showed complete consumption of **S2** (R_f = 0.27) and formation of one product spot, the intermediate **2c-OTHP** (R_f = 0.67). After quenching with 2 mL water and workup according to the general procedure, a small sample of the crude product (ca. 20 mg) was characterized and the rest of the crude product was subjected to deprotection following *general procedure A2*. To this end, **2c-OTHP** was reacted with *para*-toluenesulfonic acid monohydrate (136 mg, 0.714 mmol, 0.2 eq.) in 17.9 mL MeOH at 20 °C overnight. Analysis of the resulting mixture (1:2 EtOAc/heptane) showed complete consumption of **2c-OTHP** (R_f = 0.67) and formation of two product spots, the desired product **2c-OH** (R_f = 0.27) and the cleaved protecting group (R_f = 0.72). Workup and purification according to the general procedure yielded the alcohol **2c-OH** as a palely yellowish oil (265 mg, 2.038 mmol, 56% yield). *This procedure provided **2c-OH** along with ca. 6% of an (E)/(Z)-mixture of the internal alkene isomer **1c-OH** as an inseparable byproduct.*

Analytical data for **2c-OTHP**:

^{1}H NMR (600 MHz, $CDCl_3$) δ 5.07 (s, 1 H), 4.96 (s, 1 H), 4.60 (dd, *J* = 4.4, 2.9 Hz, 1 H), 3.93 (s, 2 H), 3.89−3.84 (m, 2 H), 3.55−3.48 (m, 2 H), 3.46 (q, *J* = 7.0 Hz, 2 H), 2.37 (td, *J* = 7.0, 1.2 Hz, 2 H), 1.84−1.78 (m, 1 H), 1.73−1.67 (m, 1 H), 1.60−1.49 (m, 4 H), 1.21 (t, *J* = 7.0 Hz, 3 H)

^{13}C NMR (151 MHz, $CDCl_3$) δ 143.7, 112.7, 98.9, 73.9, 66.3, 65.7, 62.5, 33.5, 30.9, 25.6, 19.7, 15.3

HRMS (ESI, m/z) 237.14590, calc 237.14612 for $[C_{12}H_{22}O_3Na]^+$ as $[M+Na]^+$

Analytical data for **2c-OH**:

^{1}H NMR (600 MHz, $CDCl_3$) δ 5.12 (brs, 1 H), 5.02 (brs, 1 H), 3.93 (d, *J* = 1.1 Hz, 2 H), 3.72 (t, *J* = 5.9 Hz, 2 H), 3.51 (q, *J* = 7.0 Hz, 2 H), 2.37 (td, *J* = 5.9, 1.2 Hz, 2 H), 1.22 (t, *J* = 7.0 Hz, 3 H)

^{13}C NMR (151 MHz, $CDCl_3$) δ 143.7, 115.8, 74.2, 66.0, 61.8, 37.9, 15.2

HRMS (ESI, m/z) 153.08878, calc 153.08860 for $[C_7H_{14}O_2Na]^+$ as $[M+Na]^+$

3-(Ethoxymethyl)but-3-en-1-yl phosphate (**2c-P**)

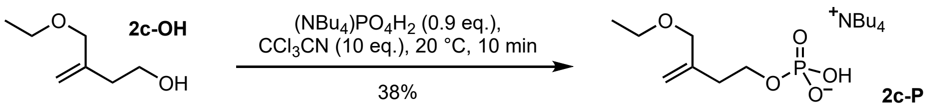

Following *general procedure B*, alcohol **2c-OH** (97 mg, 0.657 mmol, ca. 108 µL, 1.0 eq.) was reacted with tetrabutylammonium dihydrogen phosphate (200 mg, 0.591 mmol, 0.9 eq.) and trichloroacetonitrile (749 mg, 6.566 mmol, 520 µL, 10 eq.) in 1.6 mL DCM. Workup and purification according to the general procedure yielded the monophosphate **2c-P** as a brownish syrup (183 mg, 0.251 mmol, 38% yield as the 1.7·TBA salt with 1.7 eq. residual MeCN, 88% monophosphate content and a mean molecular weight of 613 g mol^{-1} assuming that the different phosphorylated product have the same molecular weight as the monophosphate). *Note: Since the precursor **2c-OH** contained ca. 6% (E)- and (Z)-**1c-OH** as inseparable impurities, this procedure also yielded the corresponding phosphates (E)/(Z)-**1c-P** (ca. 6%).*

Analytical data for **2c-P** (only for the monophosphate):

^{1}H NMR (600 MHz, CD_3CN) δ 5.01 (brs, 1 H), 4.94 (brs, 1 H), 3.85 (brs, 2 H), 3.89 (s, 2 H), 3.83 (q, *J* = 6.8 Hz, 2 H), 3.43 (dq, *J* = 7.0, 0.7 Hz, 2 H), 3.14−3.08* (m, 14 H), 2.29 (t, *J* = 7.1 Hz, 2 H), 1.64−1.57* (m, 14 H), 1.35* (hex, *J* = 7.4 Hz, 14 H), 1.15 (dd, *J* = 7.0 Hz, 3 H), 0.96* (dd, *J* = 7.4 Hz, 20 H)

^{13}C NMR (151 MHz, CD_3CN) δ 145.4, 112.2, 73.9, 66.0, 63.5 (d), 59.3*, 35.4 (d), 24.3*, 20.3*, 15.5 13.8*

^{31}P NMR (243 MHz, CD_3CN) δ 0.3 (s)

*TBA

HRMS (ESI, m/z) 209.05847, calc 209.05844 for $[C_7H_{14}O_5P]^-$ as $[M_{anion}]^-$

4-Propargoxyisoprenyl monophosphate (**2d-P**) via **S2 → 2d-OTHP → 2d-OH → 2d-P**

3-((prop-2-yn-1-yloxy)methyl)but-3-en-1-ol (**2d-OH**)

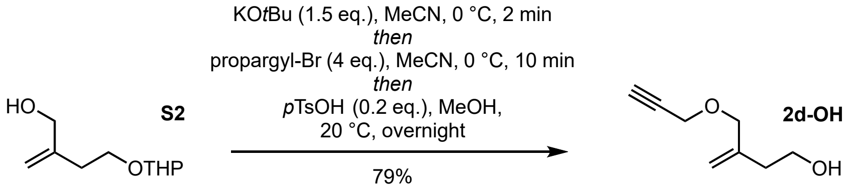

Following *general procedure A1*, intermediate **S2** (ca. 90% pure considering cosolvent content, 780 mg, 3.774 mmol, ca. 867 µL, 1.0 eq.) was reacted with potassium *tert*-butoxide (5.661 mmol, 1.5 eq., 5.66 mL of a 1 M solution in THF) and propargyl bromide (15.097 mmol, 4.0 eq., 1.64 mL of an 80% solution in toluene stabilized with MgO) in 18.9 mL dry MeCN. *Note: Due to the MgO stabilizer, this reaction mixture was black.* Analysis of the resulting mixture by TLC (1:2 EtOAc/heptane) showed complete consumption of **S2** (R_f = 0.27) and formation of one product spot, the intermediate **2d-OTHP** (R_f = 0.71). After quenching with 2 mL water, a small amount of the reaction mixture (ca. 1.5 mL) was withdrawn and worked up according to the general procedure and purified through a pipet column (1:2 EtOAc/heptane) yielding ca. 15 mg of the intermediate **2d-OTHP** as a palely yellowish oil. The rest of the crude material was subjected to deprotection by diluting the rest of the reaction mixture with the reagents for *general procedure A2*. To this end, **2d-OTHP** was reacted with *para*-toluenesulfonic acid monohydrate (143 mg, 0.755 mmol, 0.2 eq.) in 18.9 mL MeOH at 20 °C overnight. Analysis of the resulting mixture (1:1:9 EtOAc/MeOH/heptane) showed complete consumption of **2d-OTHP** (R_f = 0.60) and formation of two product spots, the desired product **2d-OH** (R_f = 0.17) and the cleaved protecting group (R_f = 0.72). Workup and purification according to the general procedure yielded the alcohol **2d-OH** as a brown oil (419 mg, 2.993 mmol, 79% yield). *This procedure provided **2d-OH** along with ca. 5% of an (E)/(Z)-mixture of the internal alkene isomer **1d-OH** as an inseparable byproduct.*

Analytical data for **2d-OTHP**:

^{1}H NMR (600 MHz, $CDCl_3$) δ 5.11 (s, 1 H), 5.02 (s, 1 H), 4.61−4.59 (m, 1 H), 4.13 (brs, 2 H), 4.04 (brs, 2 H), 3.89−3.83 (m, 2 H), 3.56−3.47 (m, 2 H), 2.42 (brs, 1 H), 2.37 (t, *J* = 7.0 Hz, 2 H), 1.84−1.78 (m, 1 H), 1.73−1.67 (m, 1 H), 1.60−1.48 (m, 4 H)

^{13}C NMR (151 MHz, $CDCl_3$) δ 142.7, 114.1, 98.9, 79.9, 74.5, 72.9, 66.2, 62.5, 57.1, 33.5, 30.9, 25.6, 19.7

HRMS (ESI, m/z) 247.13050, calc 247.13047 for $[C_{13}H_{20}O_3Na]^+$ as $[M+Na]^+$

Analytical data for **2d-OH**:

^{1}H NMR (600 MHz, $CDCl_3$) δ 5.17 (s, 1 H), 5.07 (s, 1 H), 4.16 (s, 2 H), 4.04 (s, 2 H), 3.74 (t, *J* = 6.2 Hz, 2 H), 2.44 (brs, 1 H), 2.37 (t, *J* = 6.2 Hz, 2 H)

^{13}C NMR (151 MHz, $CDCl_3$) δ 142.4, 116.3, 79.5, 74.9, 72.9, 61.2, 57.4, 37.3

HRMS (ESI, m/z) 163.07321, calc 163.07295 for $[C_8H_{11}O_2Na]^+$ as $[M+Na]^+$

3-((prop-2-yn-1-yloxy)methyl)but-3-en-1-yl phosphate (**2d-P**)

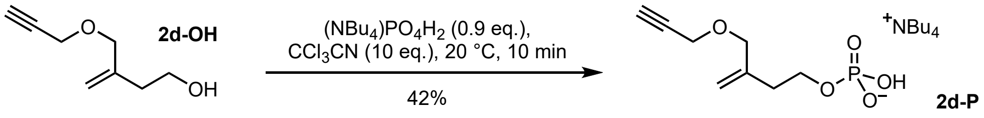

Following *general procedure B*, alcohol **2d-OH** (100 mg, 0.657 mmol, ca. 111 µL, 1.0 eq.) was reacted with tetrabutylammonium dihydrogen phosphate (200 mg, 0.591 mmol, 0.9 eq.) and trichloroacetonitrile (749 mg, 6.566 mmol, 520 µL, 10 eq.) in 1.6 mL DCM. Workup and purification according to the general procedure yielded the monophosphate **2d-P** as a brownish syrup (181 mg, 0.278 mmol, 42% yield as the 1.5·TBA salt with 1.5 eq. residual MeCN, 88% monophosphate content and a mean molecular weight of 652 g mol^{-1} assuming that the different phosphorylated product have the same molecular weight as the monophosphate). *Note: Since the precursor **2d-OH** contained ca. 6% (E)- and (Z)-**1d-OH** as inseparable impurities, this procedure also yielded the corresponding phosphates (E)/(Z)-**1d-P** (ca. 2%).*

Analytical data for **2d-P** (only for the monophosphate):

^{1}H NMR (600 MHz, CD_3CN) δ 5.02 (brs, 1 H), 5.00 (brs, 1 H), 4.12 (d, *J* = 2.4 Hz, 2 H), 3.99 (brs, 2 H), 3.80 (q, *J* = 6.8 Hz, 2 H), 3.14−3.09* (m, 12 H), 2.73 (t, *J* = 2.4 Hz, 2 H), 2.28 (t, *J* = 7.0 Hz, 2 H), 1.64−1.57* (m, 12 H), 1.35* (hex, *J* = 7.4 Hz, 12 H), 0.96* (dd, *J* = 7.4 Hz, 18 H)

^{13}C NMR (151 MHz, CD_3CN) δ 144.6, 113.3, 81.0, 75.6, 73.3, 63.1 (d), 59.3*, 35.5 (d), 24.3*, 20.3*, 15.5 13.8*

^{31}P NMR (243 MHz, CD_3CN) δ 0.5 (s)

*TBA

HRMS (ESI, m/z) 219.04259, calc 219.04279 for $[C_8H_{12}O_5P]^-$ as $[M_{anion}]^-$

4-Benzyloxyisoprenyl monophosphate (**2e-P**) via **S2 → 2d-OTHP → 2d-OH → 2d-P**

3-(benzyloxymethyl)but-3-en-1-ol (**2e-OH**)

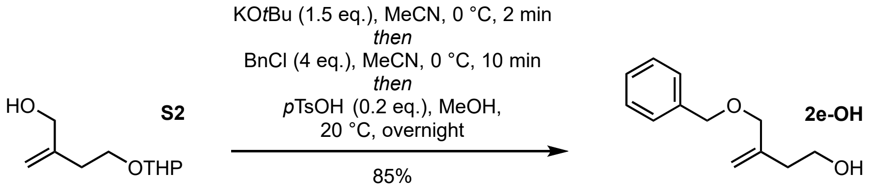

Following *general procedure A1*, intermediate **S2** (ca. 90% pure considering cosolvent content, 800 mg, 3.871 mmol, ca. 889 µL, 1.0 eq.) was reacted with potassium *tert*-butoxide (5.806 mmol, 1.5 eq., 5.81 mL of a 1 M solution in THF) and benzyl chloride (1.84 g, 15.484 mmol, 4.0 eq., 1.67 mL) in 19.4 mL dry MeCN. Analysis of the resulting mixture by TLC (1:2 EtOAc/heptane) showed complete consumption of **S2** (R_f = 0.27) and formation of one product spot, the intermediate **2e-OTHP** (R_f = 0.74). After quenching with 4 mL water, a small amount of the reaction mixture (ca. 1.5 mL) was withdrawn and worked up according to the general procedure and purified through a pipet column (1:10 EtOAc/heptane) yielding ca. 20 mg of the intermediate **2e-OTHP** as a yellowish oil. The rest of the crude material was subjected to deprotection by diluting the rest of the reaction mixture with the reagents for *general procedure A2*. To this end, **2e-OTHP** was reacted with *para*-toluenesulfonic acid monohydrate (147 mg, 0.774 mmol, 0.2 eq.) in 19.3 mL MeOH at 20 °C overnight. Analysis of the resulting mixture (1:1:9 EtOAc/MeOH/heptane) showed complete consumption of **2e-OTHP** (R_f = 0.56) and formation of two product spots, the desired product **2e-OH** (R_f = 0.35) and the cleaved protecting group (R_f = 0.72). Workup and purification according to the general procedure yielded the alcohol **2e-OH** as a yellow oil (634 mg, 3.302 mmol, 85% yield). *This procedure provided **2e-OH** along with ca. 2% of an (E)/(Z)-mixture of the internal alkene isomer **1e-OH** as an inseparable byproduct.*

Analytical data for **2e-OTHP**:

^{1}H NMR (600 MHz, $CDCl_3$) δ 7.36−7.27 (m, 5 H), 5.12 (s, 1 H), 5.01 (s, 1 H), 4.59 (brs, 1 H), 4.50 (s, 2 H), 4.00 (s, 2 H), 3.90−3.82 (m, 2 H), 3.56−3.47 (m, 2 H), 2.40 (t, *J* = 7.1 Hz, 2 H), 1.84−1.77 (m, 1 H), 1.72−1.66 (m, 1 H), 1.60−1.48 (m, 4 H)

^{13}C NMR (151 MHz, $CDCl_3$) δ 143.5, 138.5, 128.5 (x2), 127.8 (x2), 127.7, 113.3, 98.9, 62.5, 33.5, 30.9, 25.6, 19.7

HRMS (ESI, m/z) 299.16166, calc 299.16177 for $[C_{17}H_{24}O_3Na]^+$ as $[M+Na]^+$

Analytical data for **2e-OH**:

^{1}H NMR (600 MHz, $CDCl_3$) δ 7.39−7.27 (m, 5 H), 5.17 (s, 1 H), 5.06 (s, 1 H), 4.53 (s, 2 H), 3.99 (s, 2 H), 3.73 (t, *J* = 6.0 Hz, 2 H), 2.39 (t, *J* = 6.0 Hz, 2 H)

^{13}C NMR (151 MHz, $CDCl_3$) δ 143.2, 138.0, 128.6 (x2), 128.0 (x2), 115.9, 73.5, 72.4, 61.5, 37.5

HRMS (ESI, m/z) 215.10420, calc 215.10425 for $[C_{12}H_{16}O_2Na]^+$ as $[M+Na]^+$

3-(benzyloxymethyl)but-3-en-1-yl phosphate (**2e-P**)

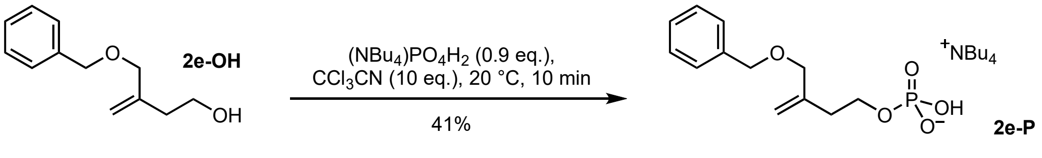

Following *general procedure B*, alcohol **2e-OH** (133 mg, 0.658 mmol, ca. 148 µL, 1.0 eq.) was reacted with tetrabutylammonium dihydrogen phosphate (201 mg, 0.592 mmol, 0.9 eq.) and trichloroacetonitrile (750 mg, 6.581 mmol, 521 µL, 10 eq.) in 1.6 mL DCM. Workup and purification according to the general procedure yielded the monophosphate **2e-P** as an orange syrup (198 mg, 0.272 mmol, 41% yield as the 1.5·TBA salt with 2.0 eq. residual MeCN, 85% monophosphate content and a mean molecular weight of 727 g mol^{-1} assuming that the different phosphorylated product have the same molecular weight as the monophosphate). *Note: Since the precursor* ***2e-OH*** *contained ca. 2% (E)- and (Z)-**1e-OH** as inseparable impurities, this procedure also yielded the corresponding phosphates (E)/(Z)-**1e-P** (ca. 1%).*

Analytical data for **2e-P** (only for the monophosphate):

^{1}H NMR (600 MHz, CD_3CN) δ 7.36−7.32 (m, 4 H), 7.29−7.26 (m, 1 H), 5.04 (brs, 1 H), 4.99 (brs, 1 H), 4.47 (s, 2 H), 3.99 (s, 2 H), 3.83 (q, *J* = 6.9 Hz, 2 H), 3.14−3.08* (m, 12 H), 2.32 (t, *J* = 7.0 Hz, 2 H), 1.64−1.57* (m, 12 H), 1.35* (hex, *J* = 7.4 Hz, 12 H), 0.96* (dd, *J* = 7.4 Hz, 18 H)

^{13}C NMR (151 MHz, CD_3CN) δ 145.3, 140.0, 129.2 (x2), 128.6 (x2), 128.3, 112.5, 73.9, 72.5, 63.2 (d), 59.2*, 35.6 (d), 24.3*, 20.3*, 13.8*

^{31}P NMR (243 MHz, CD_3CN) δ 0.5 (s)

*TBA

HRMS (ESI, m/z) 271.07417, calc 271.07408 for $[C_{12}H_{16}O_5P]^-$ as $[M_{anion}]^-$

Semi-preparative biotransformations

To obtain reference material to confirm the selectivity of PEs with modified building blocks, we performed semi-preparative biotransformations to make the glycerol ethers of the chain-extended prenoids derived from the modified starters. To this end, we performed one-pot reactions starting from the monophosphates of the starter and the extender as well as glycerol phosphate as a prenyl acceptor. In these cascade, *Mb*IPK T77A phosphorylates the (iso)prenyl phosphates, a prenylelongase performs one or two chain extentions and *Af*G_3PS (which is inactive with non-extended building blocks, Fig. S4) connects the extended chain to a glycerol unit. Subsequent hydrolysis by a phosphatase then yields the free glycerol ether. These reactions were performed in 50 mL cylindrical tubes with 0.6 mM starter unit, 0.6 mM **2a-P** as extender, 2 mM glycerol-1-phosphate (as its sodium salt), 3 mM ATP (as its sodium salt), 8 mM $MgCl_2$, 0.15 µg mL^{-1} *Gt*IPP (0.0014 mol%), 10−60 µg mL^{-1} *Mb*IPK T77A (0.12−0.72 mol%), 0.1 g L^{-1} *Sp*FPPS (0.35 mol%), and 0.1 µg mL^{-1} *Af*G_3PS (0.31 mol%), in 50 mM taurine buffer in a total volume of 20 mL at 20 °C for two days. At this point, analysis of the reaction mixture by ^{31}P NMR (50 µL of the reaction mixture were diluted with 700 µL D_2O, filtered, and analyzed by ^{31}P NMR, 243 MHz, 128 scans) indicated full consumption of **2a-P** (s, 3.39 ppm in D_2O, pH 9), starter-P (s, 3.53 ppm), **2a-PP** (d, −9.74 ppm), and starter-PP (d, −9.62 ppm, the signals near −6.08 ppm for the starter and extender overlap with those of ADP) as well as formation of a glycerol monophosphate (s, 3.89 ppm) from the glycerol-1-P starting material (s, 4.04 ppm). Then, 300 µL *Ec*AP lysate (prepared by lysing the pellet of a 500 mL culture of the expression strain in 25 mL lysis buffer and diluting the resulting lysate with glycerol to give a total of volume of 50 mL) were added and the mixture was incubated at 20 °C for 1 d. At this point, analysis of the cloudy reaction mixture by ^{31}P NMR (performed as described above) typically showed complete dephosphorylation of all species except for the phosphodiester and some remaining glycerol-1-P. Next, the reaction mixture was saturated with NaCl and extracted twice with 20 mL EtOAc. The combined organic phases were dried over Na_2SO_4, concentrated *in vacuo*, redissolved in a minimal amount of 1:1 EtOAc/heptane and purified through a pipet column (1:1 EtOAc/heptane, R_f = 0.25 for all these glycerol ethers). *Note: We used SpFPPS for these cascades because it is promiscuous, well produced and reasonably stable. However, other enzymes from our panel (e.g. SfFPPS or NcFPPS) could have filled this role as well. We abstain from reporting isolated yields for these transformations because the amounts of material are too small to report meaningful yields. The theoretical yields for these transformations are in the range of 2−3 mg and each of them returned 1−2 mg of product. AfG$_3$PS is highly stereoselective[10] and only yields the (S)-isomer, which – after dephosphorylation and changes in priority – gives the (R)-enantiomer of the free glycerol ether.*

12-Methoxyfarnesylglycerol (**4baa-G**)

(*R*)-3-(((2*E*,6*E*,10*E*)-12-Methoxy-3,7,11-trimethyldodeca-2,6,10-trien-1-yl)oxy)propane-1,2-diol (**4baa-G**)

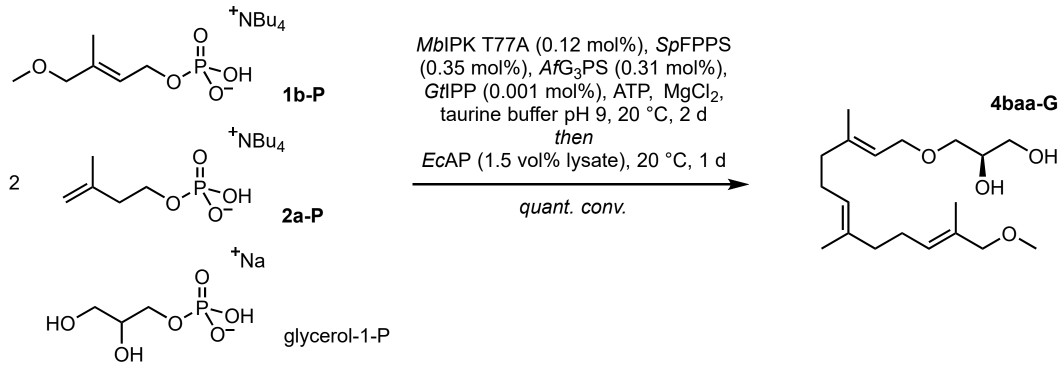

Following the general procedure with **1b-P**, **2a-P**, and 10 µg mL^{-1} *Mb*IPK T77 yielded the farnesol **4baa-G** as a colorless oil.

Analytical data for **4baa-G**:

^{1}H NMR (600 MHz, CDCl$_3$) δ 5.38 (qt, *J* = 7.2, 1.1 Hz, 1 H), 5.33 (qt, *J* = 6.8, 1.2 Hz, 1 H), 5.11 (qt, *J* = 6.8, 1.1 Hz, 1 H), 4.04 (dd, *J* = 6.9, 1.2 Hz, 2 H), 3.87 (ddt, *J* = 6.3, 5.4, 3.9 Hz, 1 H), 3.78 (s, 2 H), 3.72 (dd, *J* = 11.4, 3.9 Hz, 1 H), 3.64 (dd, *J* = 11.4, 5.2 Hz, 1 H), 3.54 (dd, *J* = 9.7, 3.8 Hz, 1 H), 3.49 (dd, *J* = 9.6, 6.2 Hz, 1 H), 3.27 (s, 3 H), 2.16−2.09 (m, 4 H), 2.07−2.00 (m, 4 H), 1.67 (s, 3 H), 1.64 (s, 3 H), 1.60 (s, 3 H)

^{13}C NMR (151 MHz, CDCl$_3$) δ 141.0, 135.2, 132.1, 128.3, 124.1, 120.5, 78.9, 71.8, 70.6, 68.1, 64.4, 57.5, 39.7, 39.4, 26.4, 26.4, 16.7, 16.1, 14.0

HRMS (ESI, m/z) compound did not ionize sufficiently well for characterization

12-Ethoxyfarnesylglycerol (**4caa-G**)

(*R*)-3-(((2*E*,6*E*,10*E*)-12-Ethoxy-3,7,11-trimethyldodeca-2,6,10-trien-1-yl)oxy)propane-1,2-diol (**4caa-G**)

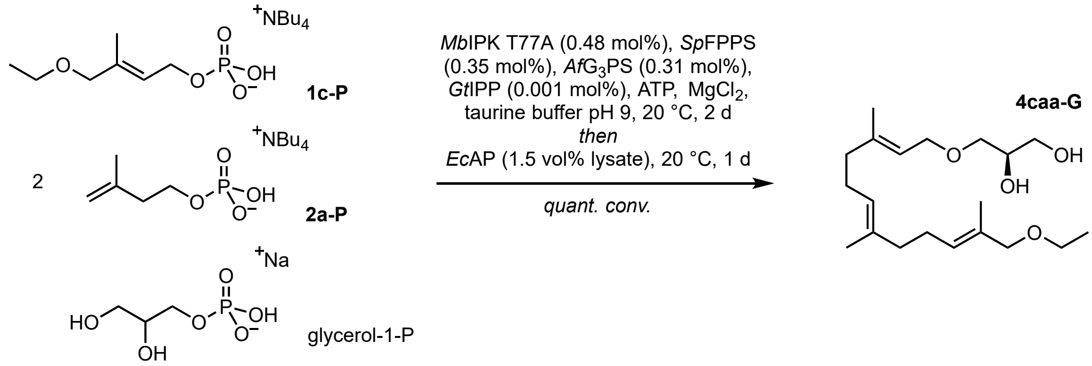

Following the general procedure with **1c-P**, **2a-P**, and 40 µg mL^{-1} *Mb*IPK T77 yielded the farnesol **4caa-G** as a colorless oil.

Analytical data for **4caa-G**:

^{1}H NMR (600 MHz, CDCl$_3$) δ 5.37 (qt, *J* = 7.1, 1.1 Hz, 1 H), 5.33 (qt, *J* = 6.8, 1.2 Hz, 1 H), 5.11 (qt, *J* = 6.9, 1.1 Hz, 1 H), 4.04 (dd, *J* = 6.8, 1.3 Hz, 2 H), 3.87 (ddt, *J* = 6.3, 5.4, 3.9 Hz, 1 H), 3.83 (s, 2 H), 3.72 (dd, *J* = 11.4, 3.9 Hz, 1 H), 3.64 (dd, *J* = 11.4, 5.2 Hz, 1 H), 3.54 (dd, *J* = 9.6, 3.8 Hz, 1 H), 3.49 (dd, *J* = 9.6, 6.2 Hz, 1 H), 3.42 (q, *J* = 7.0 Hz, 2 H), 2.15−2.09 (m, 4 H), 2.07−2.00 (m, 4 H), 1.67 (s, 3 H), 1.65 (s, 3 H), 1.60 (s, 3 H), 1.20 (t, *J* = 7.0 Hz, 3 H)

^{13}C NMR (151 MHz, CDCl$_3$) δ 141.0, 135.3, 132.5, 127.8, 124.1, 120.5, 76.9, 71.8, 70.6, 68.1, 65.1, 64.4, 39.7, 39.4, 26.5, 26.4, 16.7, 16.1, 15.4, 14.0

HRMS (ESI, m/z) compound did not ionize sufficiently well for characterization

12-Propargoxyfarnesylglycerol (**4daa-G**)

(*R*)-3-(((2*E*,6*E*,10*E*)-3,7,11-trimethyl-12-(prop-2-yn-1-yloxy)dodeca-2,6,10-trien-1-yl)oxy)propane-1,2-diol (**4daa-G**)

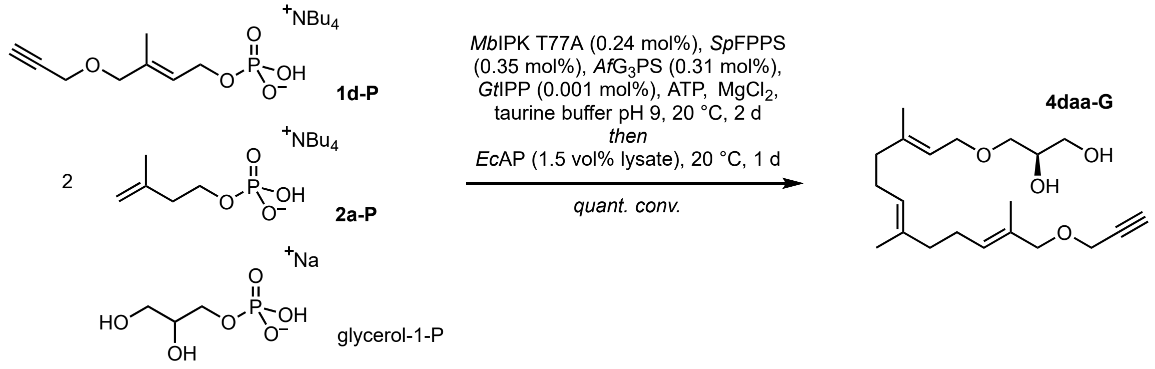

Following the general procedure with **1d-P**, **2a-P**, and 20 µg mL^{-1} *Mb*IPK T77A yielded the farnesol **4daa-G** as a colorless oil.

Analytical data for **4daa-G**:

^{1}H NMR (600 MHz, CDCl$_3$) δ 5.42 (t, *J* = 7.1 Hz, 1 H), 5.33 (t, *J* = 6.9 Hz, 1 H), 5.11 (t, *J* = 7.0 Hz, 1 H), 4.08 (d, *J* = 2.3 Hz, 2 H), 4.04 (d, *J* = 6.9 Hz, 2 H), 3.93 (s, 2 H), 3.89−3.85 (m, 1 H), 3.74−3.70 (m, 1 H), 3.64 (dd, *J* = 11.4, 5.2 Hz, 1 H), 3.54 (dd, *J* = 9.7, 3.8 Hz, 1 H), 3.40 (dd, *J* = 9.7, 6.3 Hz, 1 H), 2.41 (t, *J* = 2.3 Hz, 1 H), 2.16−2.09 (m, 4 H), 2.07−2.00 (m, 4 H), 1.67 (s, 3 H), 1.65 (s, 3 H), 1.60 (s, 3 H)

^{13}C NMR (151 MHz, CDCl$_3$) δ 141.0, 135.3, 131.4, 129.5, 124.2, 120.5, 80.2, 76.0, 74.2, 71.8, 70.6, 68.1, 64.4, 56.5, 39.7, 39.3, 26.5, 26.4, 16.7, 16.1, 14.0

HRMS (ESI, m/z) 373.23467, calc 373.23493 for $[C_{21}H_{34}O_4Na]^+$ as $[M+Na]^+$

Analysis by 2D NMR confirmed the (2*E*,6*E*,10*E*)-configuration of the prenyl chain of **4daa-G**. The configuration of the other modified farnesyl chains is assigned by analogy since they possess identical chemical shifts for the alkene protons in 6-position and 10-position.

4daa-G

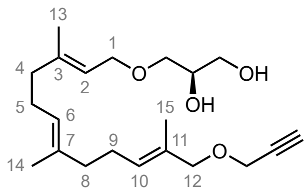

Position	δ_H (CDCl$_3$)	δ_C (CDCl$_3$)
1	4.04 (d)	68.1
2	5.33 (t)	120.4
3		141.0
4	ca. 2.03 (m)	39.3/39.7
5	ca. 2.14 (m)	26.4/26.5
6	5.11 (t)	124.2
7		135.3
8	ca. 2.03 (m)	39.3/39.7
9	ca. 2.14 (m)	26.4/26.5
10	5.42 (t)	129.5
11		131.4
12	3.93 (s)	76.0
13	1.67 (s)	16.7
14	1.60 (s)	16.1
15	1.65 (s)	14.0

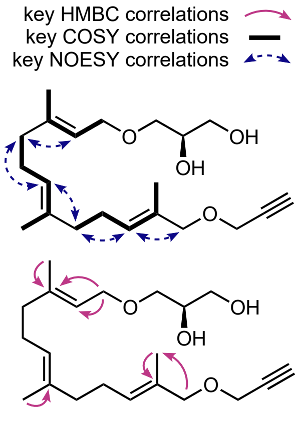

8-Benzyloxygeranylglycerol (**3ea-G**)

(*R*)-3-(((2*E*,6*E*)-8-(benzyloxy)-3,7-dimethylocta-2,6-dien-1-yl)oxy)propane-1,2-diol (**3ea-G**)

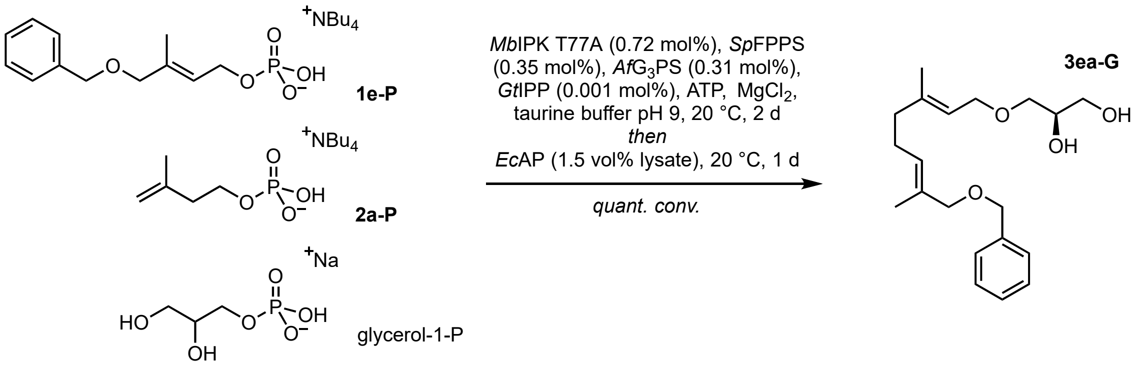

Following the general procedure with **1e-P**, **2a-P**, and 60 µg mL^{-1} *Mb*IPK T77A yielded the geraniol **3ea-G** as a colorless oil.

Analytical data for **3ea-G**:

^{1}H NMR (600 MHz, $CDCl_3$) δ 7.35−7.33 (m, 4 H), 7.30−7.27 (m, 1 H), 5.40 (t, *J* = 7.0 Hz, 1 H), 5.34 (t, *J* = 6.9 Hz, 1 H), 4.04 (dd, *J* = 6.7, 0.9 Hz, 2 H), 3.98 (s, 2 H), 3.87−3.83 (m, 1 H), 3.70 (dd, *J* = 11.5, 3.7 Hz, 1 H), 3.62 (dd, *J* = 11.4, 5.2 Hz, 1 H), 3.52 (dd, *J* = 9.7, 3.9 Hz, 1 H), 3.48 (dd, *J* = 9.7, 6.2 Hz, 1 H), 2.22−2.17 (m, 2 H), 2.12−2.08 (m, 2 H), 1.68 (s, 6 H)

^{13}C NMR (151 MHz, $CDCl_3$) δ 141.5, 138.6, 132.5, 128.4 (x2), 127.8 (x2), 127.6, 127.5, 120.6, 76.2, 71.6, 71.5, 70.5, 67.9, 64.3, 39.2, 25.9, 16.5, 14.0

HRMS (ESI, m/z) 357.20328, calc 357.20363 for $[C_{20}H_{30}O_4Na]^+$ as $[M+Na]^+$

This synthesis was also performed at a larger scale up to examine the scalability of this transformation. To this end, a solution containing 1 mM **1e-P** (47 mg, 0.07 mmol, 14 mL of a 5 mM solution), 1.3 mM **2a-P** (18.2 mM of a 5 mM solution), 3 mM glycerol-1-phosphate, 2.6 mM ATP, 10 mM $MgCl_2$, 0.15 µg mL^{-1} *Gt*IPP, 80 µg mL^{-1} *Mb*IPK T77A, 0.1 g L^{-1} *Sp*FPPS, and 0.1 µg mL^{-1} *Af*G$_3$PS, in 50 mM taurine buffer in a total volume of 70 mL was incubated at 20 °C for two days. At this point, analysis of the reaction mixture by ^{31}P NMR (50 µL of the reaction mixture were diluted with 600 µL D_2O, filtered, and analyzed by ^{31}P NMR) indicated full consumption of **2a-P** (s, 3.39 ppm in D_2O, pH 9), **1e-P** (s, 3.53 ppm), **2a-PP** (d, −9.74 ppm), and **1e-PP** (d, −9.62 ppm) as well as formation of a glycerol monophosphate (s, 3.89 ppm) from the glycerol-1-P starting material (s, 4.04 ppm). The ^{31}P NMR spectrum of this intermediary stage is provided in the externally hosted supplementary information at zenodo.org.[2] Then, 300 µL *Ec*AP lysate (prepared as described above) were added and the mixture was incubated at 20 °C for 2 d. At this point, analysis of the cloudy reaction mixture by ^{31}P NMR (performed as described above) showed complete dephosphorylation of all species except for the phosphodiester and some remaining glycerol-1-P. This ^{31}P NMR spectrum is also provided at zenodo.org.[2] Next, the reaction mixture was saturated with NaCl and extracted twice with 70 mL EtOAc. The combined organic phases were filtered through a silica plug, dried over Na_2SO_4, and concentrated *in vacuo*, to yield the glycerol ether **3ea-G** (13 mg, 0.039 mmol, 56% yield). This material matched the purified sample of **3ea-G** from the small scale by ^{1}H NMR (spectra provided at zenodo.org.[2]).

Methoxygeraniol (**3ab-OH**)

(*Z*)-3-(methoxymethyl)-7-methylocta-2,6-dien-1-ol (**3ab-OH**)

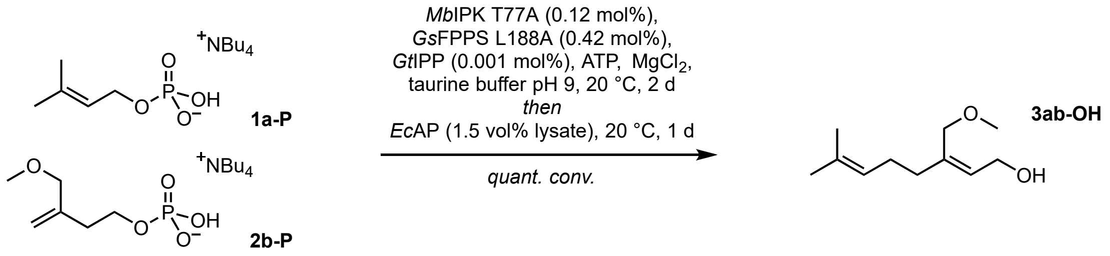

In analogy to the glycerol ethers described above, the methoxygeraniol **3ab-OH** was prepared by biocatalytic assembly in semi-preparative scale. To this end, a reaction mixture with 0.6 mM **1a-P**, 0.6 mM **2b-P**, 3 mM ATP (as its sodium salt), 8 mM $MgCl_2$, 0.15 µg mL^{-1} *Gt*IPP (0.0014 mol%), 10 µg mL^{-1} *Mb*IPK T77A (0.12 mol%), and 30 µg mL^{-1} *Gs*FPPS variant L188A (0.41 mol%), in 50 mM taurine buffer in a total volume of 20 mL was incubated in a 50 mL cylindrical tube at 20 °C for two days. Then, 300 µL *Ec*AP lysate (prepared by lysing the pellet of a 500 mL culture of the expression strain in 25 mL lysis buffer and diluting the resulting lysate with glycerol to give a total of volume of 50 mL) were added and the mixture was incubated at 20 °C for a 1 d. Next, the reaction mixture was saturated with NaCl and extracted twice with 20 mL EtOAc. The combined organic phases were dried over Na_2SO_4 and concentrated *in vacuo*, yielding **3ab-OH** as a colorless oil. *Note: This compound is labile on silica gel. It does not run cleanly on TLC and a previous attempt to purify this compound through a small silica plug resulted in a complete loss of the material. Hence we obtained the following analytical data from the crude material.*

Analytical data for **3ab-OH**:
^{1}H NMR (600 MHz, $CDCl_3$) δ 5.68 (t, *J* = 7.0 Hz, 1 H), 5.12−5.08 (m, 1 H), 4.17 (d, *J* = 6.9 Hz, 2 H), 3.95 (s, 2 H), 3.33 (s, 3 H), 2.13−2.11 (m, 4 H), 1.69 (s, 3 H), 1.61 (s, 3 H)
^{13}C NMR (151 MHz, $CDCl_3$) δ 139.9, 131.7, 127.9, 123.6, 70.4, 58.8, 58.1, 35.7, 26.4, 25.6, 17.5
HRMS (ESI, m/z) compound did not ionize sufficiently well for characterization

Analysis by 2D NMR confirmed the (*Z*)-configuration of **3ab-OH** and allowed a full assignment of the unusual ^{1}H NMR signals of this prenyl chain.

3ab-OH

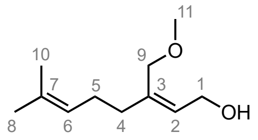

Position	δ_H ($CDCl_3$)	δ_C ($CDCl_3$)
1	4.17 (d)	58.8
2	5.68 (t)	127.9
3		139.9
4	ca. 2.12 (m)	35.7
5	ca. 2.12 (m)	26.4
6	ca. 5.10 (m)	123.6
7		131.7
8	1.61 (s)	17.5
9	3.95 (s)	70.4
10	1.69 (s)	25.6
11	3.33 (s)	58.1

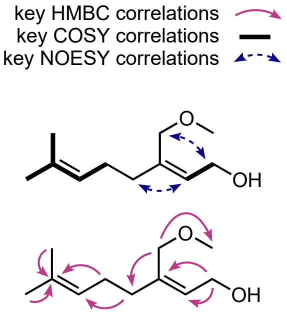

Additional discussion

Synthesis of prenyl pyrophosphates

Thanks to their privileged role in the biosynthesis of natural products, allylic pyrophosphates have a long history in bioorganic chemistry. The two common ways to prepare these compounds is either i) by nucleophilic substitution using a tetralkylammonium pyrophosphate salt and a prenyl electrophile or ii) by condensation of a phosphate salt with a prenyl alcohol. Both strategies have numerous historic precedents (as cited above, page 70, and in the main text) and neither is ideal. Strategy i) generally affords the desired pyrophosphate cleanly, although hydrolysis byproducts are common and a synthesis generally takes two days per analogue due to a lengthy purification with freeze-drying steps. Strategy ii) generally affords the desired pyrophosphate as a mixture with the free alcohol, the monophosphate and the triphosphate, requiring a laborious and typically low-yielding purification. In addition, neither strategy is well-suited to the synthesis of more polar, heteroatom-bearing analogues as these analogues often feature functional groups which either interfere with the synthetic strategy or complicate the purification of the product. For example, we encountered this issue with the ether-bearing analogues in our substrate panel which were prone to hydrolysis and did not retain sufficiently well on chromatography material to permit their purification (also see the Notes on failed routes, page 74). As such, the chemoenzymatic strategy we advanced in our work is a valuable addition to the toolbox since it allows rapid access to mono- and pyrophosphates with minimal purification effort and affords greater functional group tolerance than established methods. We also showed how *in situ* activation can readily be interfaced with the assay providing an activity read-out, foregoing the potentially challenging and laborious purification of polar prenyl pyrophosphates.

Assaying prenylelongases

Previous continuous biochemical assays for PEs (and prenyltransferases in general) either relied on the detection of the released inorganic pyrophosphate or on the use of radioactive substrate analogues, as cited in the main text. The latter strategy is inherently not suited to high-throughput experimentation and requires specialized equipment and laboratory space. In contrast, the former strategy is readily compatible with parallelized experimentation and a variety of different assay systems have been described previously. We extensively compiled and reviewed these in the Supplementary Information of our original PUB paper.[11] Common ways to generate a UV signal from (pyro-)phosphate include coupled assays involving a nucleoside phosphorylase or an oxireductase (e.g. glucose-6-phosphate dehydrogenase or glyceraldehyde-3-phosphate dehydrogenase). However, these assay systems generally require either expensive reagents (e.g. methylated purine nucleosides or NADP) or three to four enzymes of which some are not or poorly commercially available. As such, we originally developed (E)PUB to bypass these inherent drawbacks of existing methods. EPUB features only two enzymes, which are thermostable and can readily be produced in *E. coli* (homologues are also commercially available) as well as 5-bromouridine as a chromophore which is cheaply available on the gram scale.

Discontinuous phosphate detection methods (e.g. via Malachite green) have previously been applied to PEs and prenyltransferases (see the main text). However, these methods are generally much more labor-intensive, provide a smaller quantity of data and are poorly suited to kinetic analyses as each datapoint requires individual quenching and analysis.

Interrogating PEs by EPUB using *E. coli* lysates

During the early phases of this project, we experimented with the use of cleared cell lysates with EPUB. Lysate screening is generally advantageous over the interrogation of purified enzymes since it greatly improves throughput. Although EPUB generally works in lysates, we encountered two key issues with PEs. Neither precludes the application of EPUB for the interrogation of enzyme libraries as lysates, but they did prompt us to work with purified protein for the present study.

First, *E. coli*'s inherent phosphatases generate significant background signal due to hydrolysis of the phosphorylated substrates in the reaction mixture (**1a-P**, **1a-PP**, ATP, etc.). This requires a reaction of interest, PE-mediated chain extension, to significantly outpace background hydrolysis to achieve meaningful signal/noise ratios. Several enzymes from our PE panel consistently achieved that (e.g. *Rc*FPPS or *Sp*FPPS) while others were either too slow (e.g. *Mt*polyPPPS) or too labile (e.g. *Sf*FPPS) to consistently give measurable activity over the back-ground signal. Given that our PEs had low rate constants with

the substrate analogues (**1b-PP**, **1c-PP** etc.), lysate screening of variant libraries would have been unlikely to generate any significant signal/noise ratio.

Secondly, we found that PEs are produced with unreliable titers by *E. coli* when the cultivation conditions are not well controlled (as is the case in multiwell plates). We found that PEs are produced relatively quickly after induction and degraded quickly as well and that not all PEs follow the same production/degradation kinetics. For example, *Sp*FPPS activity was often detectable 6−16 h after induction, while *Gs*FPPS activity was detectable 8−24 h after induction. This made it extremely difficult to obtain reproducible results when using multiwell plates and lysates.

Although lysate screening did not prove feasible in our study, we expect that more stable enzymes with more predictable production kinetics will be tractable by EPUB in lysates. For example, *E. coli* phosphatases could be inactivated by thermal treatment of the lysate which would allow the interrogation of thermostable prenyltransferases. One could also imagine that the issue of non-reproducible production kinetics could be addressed by normalizing a screened library for protein concentration, e.g. by producing the prenyltransferase variants as split-GFP fusion proteins.

Supplementary references

(1) Brand, A.; Allen, L.; Altman, M.; Hlava, M.; Scott, J. Beyond Authorship: Attribution, Contribution, Collaboration, and Credit. *Learn. Publ.* **2015**, *28* (2), 151–155. https://doi.org/10.1087/20150211.

(2) Kaspar, F. Supplementary Material PEs. **2025**. https://doi.org/10.5281/zenodo.17425289.

(3) Swainston, N.; Baici, A.; Bakker, B. M.; Cornish-Bowden, A.; Fitzpatrick, P. F.; Halling, P.; Leyh, T. S.; O'Donovan, C.; Raushel, F. M.; Reschel, U.; Rohwer, J. M.; Schnell, S.; Schomburg, D.; Tipton, K. F.; Tsai, M.; Westerhoff, H. V.; Wittig, U.; Wohlgemuth, R.; Kettner, C. STRENDA DB: Enabling the Validation and Sharing of Enzyme Kinetics Data. *FEBS J.* **2018**, *285* (12), 2193–2204. https://doi.org/10.1111/febs.14427.

(4) Tipton, K. F.; Armstrong, R. N.; Bakker, B. M.; Bairoch, A.; Cornish-Bowden, A.; Halling, P. J.; Hofmeyr, J.-H.; Leyh, T. S.; Kettner, C.; Raushel, F. M.; Rohwer, J.; Schomburg, D.; Steinbeck, C. Standards for Reporting Enzyme Data: The STRENDA Consortium: What It Aims to Do and Why It Should Be Helpful. *Sci. Perspect.* **2014**, *1* (1–6), 131–137. https://doi.org/10.1016/j.pisc.2014.02.012.

(5) Crameri, F.; Shephard, G. E.; Heron, P. J. The Misuse of Colour in Science Communication. *Nat. Commun.* **2020**, *11* (1), 5444–5444. https://doi.org/10.1038/s41467-020-19160-7.

(6) Kaspar, F.; Crameri, F. Coloring Chemistry—How Mindful Color Choices Improve Chemical Communication. *Angew. Chem. Int. Ed.* **2022**, *61* (16), e202114910. https://doi.org/10.1002/anie.202114910.

(7) Alder, C. M.; Hayler, J. D.; Henderson, R. K.; Redman, A. M.; Shukla, L.; Shuster, L. E.; Sneddon, H. F. Updating and Further Expanding GSK's Solvent Sustainability Guide. *Green Chem.* **2016**, *18* (13), 3879–3890. https://doi.org/10.1039/C6GC00611F.

(8) Rubini, R.; Jansen, S. C.; Beekhuis, H.; Rozeboom, H. J.; Mayer, C. Selecting Better Biocatalysts by Complementing Recoded Bacteria**. *Angew. Chem. Int. Ed.* **2023**, *62* (2), e202213942. https://doi.org/10.1002/anie.202213942.

(9) Inoue, H.; Nojima, H.; Okayama, H. High Efficiency Transformation of Escherichia Coli with Plasmids. *Gene* **1990**, *96* (1), 23–28. https://doi.org/10.1016/0378-1119(90)90336-P.

(10) Kaspar, F.; Eilert, L.; Staar, S.; Oung, S. W.; Wolter, M.; Ganskow, C. S. G.; Kemper, S.; Klahn, P.; Jacob, C. R.; Blankenfeldt, W.; Schallmey, A. Biocatalytic Ether Lipid Synthesis by an Archaeal Glycerolprenylase. *Angew. Chem. Int. Ed.* **2024**, *63* (46), e202412597. https://doi.org/10.1002/anie.202412597.

(11) Eilert, L.; Schallmey, A.; Kaspar, F. UV-Spectroscopic Detection of (Pyro-)Phosphate with the PUB Module. *Anal. Chem.* **2022**, *94* (8), 3432–3435. https://doi.org/10.1021/acs.analchem.1c05356.

(12) Johnson, B. P.; Kumar, V.; Scull, E. M.; Thomas, L. M.; Bourne, C. R.; Singh, S. Molecular Basis for the Substrate Promiscuity of Isopentenyl Phosphate Kinase from *Candidatus Methanomethylophilus Alvus*. *ACS Chem. Biol.* **2022**, *17* (1), 85–102. https://doi.org/10.1021/acschembio.1c00655.

(13) Kumar, V.; Johnson, B. P.; Dimas, D. A.; Singh, S. Novel Homologs of Isopentenyl Phosphate Kinase Reveal Class-Wide Substrate Flexibility. *ChemCatChem* **2021**, *13* (17), 3781–3788. https://doi.org/10.1002/cctc.202100595.

(14) Lund, S.; Courtney, T.; Williams, G. J. Probing the Substrate Promiscuity of Isopentenyl Phosphate Kinase as a Platform for Hemiterpene Analogue Production. *ChemBioChem* **2019**, *20* (17), 2217–2221. https://doi.org/10.1002/cbic.201900135.

(15) Suzuki, K.; Ito, S.; Shimizu-Ibuka, A.; Sakai, H. Crystal Structure of Pyruvate Kinase from Geobacillus Stearothermophilus. *Journal of Biochemistry* **2008**, *144* (3), 305–312. https://doi.org/10.1093/jb/mvn069.

(16) Wigley, D. B.; Gamblin, S. J.; Turkenburg, J. P.; Dodson, E. J.; Piontek, K.; Muirhead, H.; Holbrook, J. J. Structure of a Ternary Complex of an Allosteric Lactate Dehydrogenase from *Bacillus Stearothermophilus* at 2·5 Å Resolution. *J. Mol. Biol.* **1992**, *223* (1), 317–335. https://doi.org/10.1016/0022-2836(92)90733-Z.

(17) Sakai, H.; Suzuki, K.; Imahori, K. Purification and Properties of Pyruvate Kinase from *Bacillus Stearothermophilus*. *J. Biochem.* **1986**, *99* (4), 1157–1167. https://doi.org/10.1093/oxfordjournals.jbchem.a135579.

(18) Kaspar, F.; Seeger, M.; Westarp, S.; Köllmann, C.; Lehmann, A.; Pausch, P.; Kemper, S.; Neubauer, P.; Bange, G.; Schallmey, A.; Werz, D. B.; Kurreck, A. Diversification of 4'-Methylated Nucleosides by Nucleoside Phosphorylases. *ACS Catal.* **2021**, *11* (17), 10830–10835. https://doi.org/10.1021/acscatal.1c02589.

(19) Arcus, V. L.; van der Kamp, M. W.; Pudney, C. R.; Mulholland, A. J. Enzyme Evolution and the Temperature Dependence of Enzyme Catalysis. *Curr. Opin. Struct. Biol.* **2020**, *65*, 96–101. https://doi.org/10.1016/j.sbi.2020.06.001.

(20) Arcus, V. L.; Prentice, E. J.; Hobbs, J. K.; Mulholland, A. J.; Van der Kamp, M. W.; Pudney, C. R.; Parker, E. J.; Schipper, L. A. On the Temperature Dependence of Enzyme-Catalyzed Rates. *Biochemistry* **2016**, *55* (12), 1681–1688. https://doi.org/10.1021/acs.biochem.5b01094.

(21) Arcus, V. L.; Mulholland, A. J. Temperature, Dynamics, and Enzyme-Catalyzed Reaction Rates. *Ann. Rev. Biophys.* **2020**, *49* (1), 163–180. https://doi.org/10.1146/annurev-biophys-121219-081520.

(22) Kaspar, F.; Wolff, D. S.; Neubauer, P.; Kurreck, A.; Arcus, V. L. pH-Independent Heat Capacity Changes during Phosphorolysis Catalyzed by the Pyrimidine Nucleoside Phosphorylase from *Geobacillus Thermoglucosidasius*. *Biochemistry* **2021**, *60* (20), 1573–1577. https://doi.org/10.1021/acs.biochem.1c00156.

(23) Mirdita, M.; Schütze, K.; Moriwaki, Y.; Heo, L.; Ovchinnikov, S.; Steinegger, M. ColabFold: Making Protein Folding Accessible to All. *Nat. Methods* **2022**, *19* (6), 679–682. https://doi.org/10.1038/s41592-022-01488-1.

(24) Sievers, F.; Wilm, A.; Dineen, D.; Gibson, T. J.; Karplus, K.; Li, W.; Lopez, R.; McWilliam, H.; Remmert, M.; Söding, J.; Thompson, J. D.; Higgins, D. G. Fast, Scalable Generation of High‐quality Protein Multiple Sequence Alignments Using Clustal Omega. *Mol. Syst. Biol.* **2011**, *7* (1), 539. https://doi.org/10.1038/msb.2011.75.

(25) Goddard, T. D.; Huang, C. C.; Meng, E. C.; Pettersen, E. F.; Couch, G. S.; Morris, J. H.; Ferrin, T. E. UCSF ChimeraX: Meeting Modern Challenges in Visualization and Analysis. *Prot. Sci.* **2018**, *27* (1), 14–25. https://doi.org/10.1002/pro.3235.

(26) Pettersen, E. F.; Goddard, T. D.; Huang, C. C.; Meng, E. C.; Couch, G. S.; Croll, T. I.; Morris, J. H.; Ferrin, T. E. UCSF ChimeraX: Structure Visualization for Researchers, Educators, and Developers. *Prot. Sci.* **2021**, *30* (1), 70–82. https://doi.org/10.1002/pro.3943.

(27) Meng, E. C.; Goddard, T. D.; Pettersen, E. F.; Couch, G. S.; Pearson, Z. J.; Morris, J. H.; Ferrin, T. E. UCSF ChimeraX : Tools for Structure Building and Analysis. *Prot. Sci.* **2023**, *32* (11), e4792. https://doi.org/10.1002/pro.4792.

(28) Fernández-Mateos, A.; Madrazo, S. E.; Teijón, P. H.; González, R. R. Radical Cyclization of Epoxy Vinyl- and Allylsulfones Promoted by Titanocene Chloride. *J. Org. Chem.* **2015**, *80* (9), 4378–4391. https://doi.org/10.1021/acs.joc.5b00206.

(29) Das, D.; Tnimov, Z.; Nguyen, U. T. T.; Thimmaiah, G.; Lo, H.; Abankwa, D.; Wu, Y.; Goody, R. S.; Waldmann, H.; Alexandrov, K. Flexible and General Synthesis of Functionalized Phosphoisoprenoids for the Study of Prenylation in Vivo and in Vitro. *ChemBioChem* **2012**, *13* (5), 674–683. https://doi.org/10.1002/cbic.201100733.

(30) Stephenson, L. M.; Speth, D. R. Mechanism of Allylic Hydroxylation by Selenium Dioxide. *J. Org. Chem.* **1979**, *44* (25), 4683–4689. https://doi.org/10.1021/jo00393a045.

(31) Trachtenberg, E. N.; Nelson, C. H.; Carver, J. R. Mechanism of Selenium Dioxide Oxidation of Olefins. *J. Org. Chem.* **1970**, *35* (5), 1653–1658. https://doi.org/10.1021/jo00830a083.

(32) Dixon, D. J.; Ley, S. V.; Tate, E. W. The Synthesis of Mono- and Bicyclic Ethers via Acid Catalysed Ring-Opening Cyclisation of Tetrahydropyranyl Ether Derivatives. *J. Chem. Soc., Perkin Trans. 1* **2000**, No. 12, 1829–1836. https://doi.org/10.1039/a909302h.

(33) Wollack, J. W.; Silverman, J. M.; Petzold, C. J.; Mougous, J. D.; Distefano, M. D. A Minimalist Substrate for Enzymatic Peptide and Protein Conjugation. *ChemBioChem* **2009**, *10* (18), 2934–2943. https://doi.org/10.1002/cbic.200900566.

(34) Lira, L. M.; Vasilev, D.; Pilli, R. A.; Wessjohann, L. A. One-Pot Synthesis of Organophosphate Monoesters from Alcohols. *Tet. Lett.* **2013**, *54* (13), 1690–1692. https://doi.org/10.1016/j.tetlet.2013.01.059.
